# On the strong difference in reactivity of acyclic and cyclic diazodiketones with thioketones: experimental results and quantum-chemical interpretation

**DOI:** 10.3762/bjoc.11.57

**Published:** 2015-04-20

**Authors:** Andrey S Mereshchenko, Alexey V Ivanov, Viktor I Baranovskii, Grzegorz Mloston, Ludmila L Rodina, Valerij A Nikolaev

**Affiliations:** 1 Institute of Chemistry, Saint Petersburg State University, University prosp., 26, 198504, Saint Petersburg, Russia; 2 Faculty of Chemistry, University of Łódź, Tamka 12, 91-403 Łódź, Poland

**Keywords:** diazocarbonyl compounds, 1,3-dipolar cycloaddition, 1,3-oxathioles, thiiranes, thiocarbonyl ylides, thioketones

## Abstract

The 1,3-dipolar cycloaddition of acyclic 2-diazo-1,3-dicarbonyl compounds (DDC) and thioketones preferably occurs with *Z*,*E*-conformers and leads to the formation of transient thiocarbonyl ylides in two stages. The thermodynamically favorable further transformation of C=S ylides bearing at least one acyl group is identified as the 1,5-electrocyclization into 1,3-oxathioles. However, in the case of diazomalonates, the dominating process is 1,3-cyclization into thiiranes followed by their spontaneous desulfurization yielding the corresponding alkenes. Finally, carbocyclic diazodiketones are much less reactive under similar conditions due to the locked cyclic structure and are unfavorable for the 1,3-dipolar cycloaddition due to the *Z*,*Z*-conformation of the diazo molecule. This structure results in high, positive values of the Gibbs free energy change for the first stage of the cycloaddition process.

## Introduction

Dipolar cycloadditions of diazo compounds have been of great interest for a long time as they provide a means for the preparation of a wide variety of nitrogen containing heterocyclic compounds [1–5]. These reactions are generally known for diazoalkanes and 2-diazocarbonyl compounds, whereas similar processes with 2-diazo-1,3-dicarbonyl compounds (DDC) are far less common [6–10], and reported literature data on this matter are somewhat contradictory. For example, it was established that diazomalonate and diazodimedone do not react under standard conditions with the С=S bond of thiobenzophenone [11–12], which is one of the most reactive “superdipolarophiles” known [13]. On the other hand, it was recently demonstrated that diazoacetylacetone, even at room temperature, easily reacts with Ph_2_C=S, giving rise to the formation of 1,3-oxathiole derivatives [14–15], which are considered to be the typical final products of the 1,3-dipolar cycloaddition of diazoketones to the C=S bond [16–18].

**Scheme 1:**
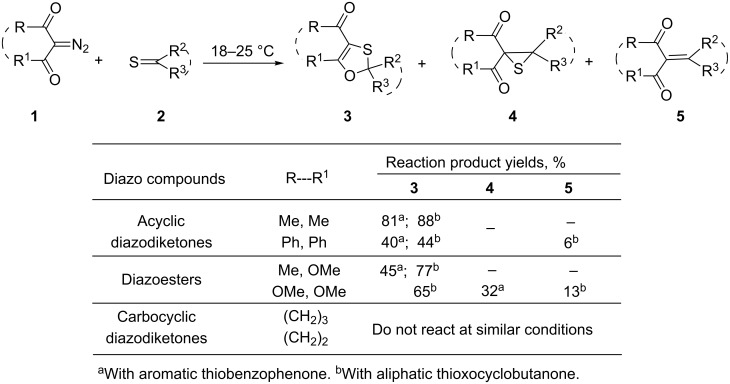
The key experimental results on the DDC **1** reactions with thioketones **2** [19–21].

In this regard, we performed a detailed experimental study of reactions of a series of 2-diazo-1,3-dicarbonyl compounds **1** with aromatic and aliphatic thioketones **2**, and it was established that they occurred in a varied manner: acyclic diazodicarbonyl compounds **1** readily reacted with thioketones **2**, whereas carbocyclic diazodiketones were essentially indifferent to aromatic and aliphatic thioketones under similar reaction conditions (Scheme 1) [14–15,19–21].

Generally, the main reaction products formed in these reactions were 1,3-oxathioles **3**. However, in some cases, thiiranes **4** and alkenes **5** were isolated from the reaction mixture as well [19–20] (Scheme 1). In addition, it was established that in the reaction of diazomalonate with aliphatic 2,2,4,4-tetramethyl-3-thioxocyclobutanone, the ratio of the reaction products **3** and **5** strongly depends on the reaction conditions: at room temperature, a mixture of 1,3-oxathiole **3** and alkene **5** (4:1) was formed, whereas at 80 °C, product **5** was formed exclusively (81%) [20].

In order to explain the obtained results and to elucidate the reaction pathway (which governs reactions of DDC **1** with aromatic and aliphatic thioketones **2**), detailed quantum-chemical calculations of the relative energy of the reagents, reaction intermediates, transition states, and reaction products on the potential energy surfaces were carried out. The results of the performed study are summarized and discussed in the present publication.

## Results and Discussion

**Figure 1:**
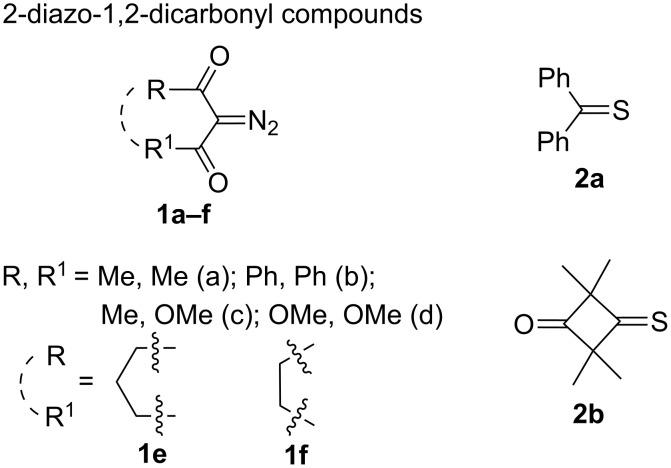
Diazo compounds **1** and thioketones **2** used in the study.

The subjects of the quantum-chemical calculations were the same diazodicarbonyl compounds and thioketones that were applied in the earlier experimental studies, namely: acyclic diazodiketones **1a**,**b**, diazoketoester **1c**, diazomalonate **1d**, and carbocyclic diazodiketones **1e**,**f**, along with thiobenzophenone (**2a**) and 2,2,4,4-tetramethyl-3-thioxocyclobutanone (**2b**) as dipolarophiles (Figure 1).

The calculations were performed at the density functional theory (DFT) level using the 6-31G(d) basis set. For reactions of diazo compounds **1a–f** with thiobenzophenone (**2a**), the solvent was simulated using the polarizable continuum model (PCM) [22], and for the case of thioketone **2b**, all calculations were performed for the gas phase.

### Reactions with thiobenzophenone (**2a**)

**Scheme 2:**
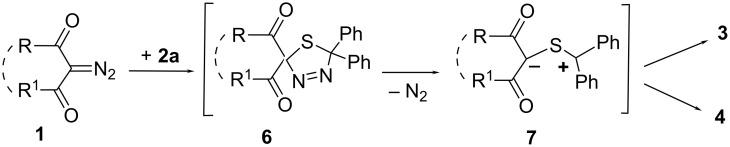
General scheme for reactions of DDC **1** with thiobenzophenone (**2a)**.

By comparison with the literature data [16–18], one can assume that the multistep reactions of DDC **1a–d** and thione **2a** are initiated by 1,3-cycloaddition of the diazogroup with the C=S bond (step 1), followed by decomposition of thiadiazoline **6** formed (step 2) and competitive electrocyclization of the intermediate thiocarbonyl ylide **7** either into 1,3-oxathiole **3** or thiirane **4** (step 3) (Scheme 2).

According to the latest molecular orbital (MO) theory, reactions of diazodicarbonyl compounds with a dipolarophile are cycloaddition processes of type II (HOMO, LUMO controlled) [23–25]. In order to confirm the proposed reaction mechanism and to explain the experimental results, geometries of the stationary points (i.e., reagents, products, intermediates, and the appropriate transition states) on the potential energy surface of the reaction of the diazo compounds **1a–f** with thiobenzophenone (**2a**) were located. The optimized structures of reagents, intermediates, and products were found to be characterized by the absence of the imaginary part of the frequency, and transition states contained only one imaginary frequency component. The calculated intrinsic reaction coordinate (IRC) paths demonstrated that the stationary points are effectively connected to each other.

**Figure 2:**
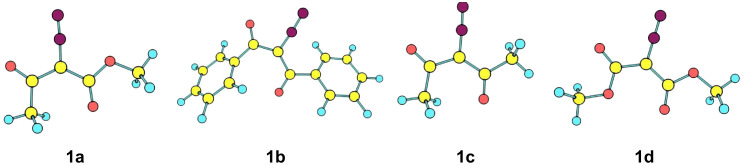
Optimized structures of the lowest energy *Z*,*E*-conformers of diazo compounds **1a–d**.

It was found that the *E*,*Z-*conformations of acyclic DDC **1a–d** have the lowest energy as compared to their *Z*,*E*-, *Z*,*Z-* and *E*,*E-*counterparts [26–28] (up to 4.7–5.0 kcal/mol). Because of this, calculations were carried out for the *E*,*Z-*conformers of acyclic **1a–d** and the *Z*,*Z*-locked conformation of carbocyclic DDC **1e**,**f**. The optimized structures of the *E*,*Z*-conformers for diazo compounds **1a–d** are shown in Figure 2.

The structural formulas and the corresponding, optimized geometry energy for the first and the second reaction steps (cycloaddition of **1** with **2a** and decomposition of 1,3,4-thiadiazolines **6**) are given in Table 1. The positive values of the Gibbs free energy change for the first stage of the process (Table 1; Δ*G*_1_) demonstrate that formation of thiadiazolines **6** from **1a–d** and **2a** is thermodynamically unfavorable. However, the total value of the Gibbs free energy change for the formation of molecular nitrogen and thiocarbonyl ylide **7** from **1a–d** and **2a**, Δ*G*_1–7 _*=* Δ*G*_1 _*+* Δ*G*_2_, is negative.

**Table 1:** Calculated relative energies (in kcal·mol^−1^) and relative reaction rates for cycloaddition reactions of diazo compounds **1a**–**g** with thiobenzophenone **2a** using the PBE1PBE/6-31G*//PCM (benzene) method. The energies calculated via B3LYP/6-31G*//PCM (benzene) are given in parenthesis.

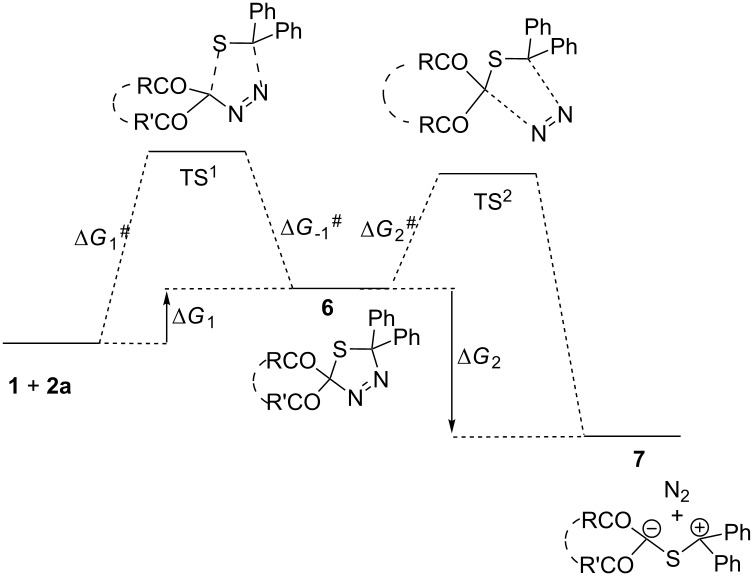							

Entry	DDC **1**; R, R^1^	Δ*G*_1_^#^	Δ*G*_1_	Δ*G*_2_^#^	Δ*G*_2_	Δ*G*_1–7 _*=* Δ*G*_1 _*+* Δ*G*_2_	*k***_1a_**/*k*_x_ at 25 °C^a^

1	**1a**; Me, Me	30.5 (36.6)	8.4 (20.3)	21.2 (16.4)	−25.1 (−34.9)	−16.7 (−14.6)	1 (1)
2	**1b**; Ph, Ph	31.3 (38.1)	7.5 (19.8)	18.3 (13.6)	−25.8 (−34.9)	−18.3 (−15.1)	3.8 (12)
3	**1c**; Me, OMe	31.2 (37.2)	8.4 (19.9)	19.6 (14.6)	−26.0 (−35.6)	−17.6 (−15.7)	3.3 (2.9)
4	**1d**; OMe, OMe	30.8 (36.4)	6.1 (17.4)	18.6 13.9)	−24.0 (−33.2)	−17.9 (−15.8)	1.7 (0.7)
5	**1e**; (CH_2_)_3_	32.7 (38.8)	12.4 (23.7)	14.9 (10.3)	−33.9 (−43.6)	−21.5 (−19.9)	43 (38)
6	**1f**; (CH_2_)_2_	35.8 (41.5)	12.4 (23.6)	12.9 (8.0)	−34.2 (−44.1)	−21.8 (−20.5)	8300 (3700)

^a^The details of *k***_1a_**/*k*_x_ calculations are given in the Computational Details section.

Therefore, the formation of the intermediate thiocarbonyl ylides of type **7** from **1a–d** and **2a** is thermodynamically favorable. The activation energy of the 1,3-dipolar cycloaddition of diazo compounds **1** to thiobenzophenone (**2a**, Δ*G*_1_^#^) is significantly larger than the decomposition energy of 1,3,4-thiadiazolines **6** (Δ*G*_2_^#^*).* In line with these data, the first step of the process (cycloaddition) must be a rate-determining step, and therefore, the larger value of Δ*G*_1_^#^ corresponds to the slower formation of thiocarbonyl ylide **7**.

IRC scans have demonstrated that diazo compounds **1** and thiobenzophenone (**2a**) are smoothly converted to thiadiazolines **6** through the single minimum energy transition state, TS^1^. This observation implies that the cycloaddition reaction proceeds via a concerted mechanism. In the case of an alternative, stepwise mechanism, one would expect the appearance of at least two transition states [29], which was not observed. In addition, the cyclic geometry of the TS^1^ also confirms the concerted cycloaddition process.

The activation energies of cycloadditions of DDC **1** with thiobenzophenone (**2a**, Table 1) are in good agreement with the reported experimental results [14–15,19]. Thus, the smallest value of Δ*G*_1_^#^ (30.5 kcal·mol^−1^, PBE1PBE, Table 1, entry 1) corresponds to the reaction of the most reactive diazoacetylacetone (**1a**) with thioketone **2a**, which was completed at room temperature over several days (and in good yields of oxathiole **3a** of up to 80%). The intermediate values of Δ*G*_1_^#^ (30.8–31.3 kcal·mol^−1^, PBE1PBE, Table 1, entries 2–4) are related to diazodicarbonyl compounds **1b–d** with reaction times of 1–3 months. The carbocyclic diazodiketone **1e** with Δ*G*_1_^#^ = 32.7 kcal·mol^−1^ (PBE1PBE, Table 1, entry 5) does not react at room temperature with **2a** (Scheme 1), while diazocyclopentanedione **1f** with the largest value of Δ*G*_1_^#^ = 35.8 kcal·mol^−1^ (PBE1PBE, Table 1, entry 6) was unreactive under all conditions.

The calculated values of the relative reaction rates (Table 1, last right column) also correlate well with the above considered experimental data. The acyclic DDC **1b–d** have much slower reaction rates as compared to diazoacetylacetone **1a**, whereas the cycloaddition of carbocyclic diazodiketones **1e**,**f** with **2a** occurs even at elevated temperatures at an insignificant rate. Apparently, these differences result from the fixed cyclic structure *Z*,*Z*-conformation of DDCs **1e**,**f**, which is unfavorable for the 1,3-dipolar cycloaddition by stereochemical and/or the energetic parameters of the process.

### Reactions with 2,2,4,4-tetramethyl-3-thioxocyclobutanone (**2b**)

The mechanism of the 1,3-dipolar cycloaddition of diazo compounds **1** with aliphatic thioketone **2b** was assumed to be similar to their reaction with aromatic thione **2a** (Scheme 2). Thus, the reaction of **1** with the C=S bond of thioketone **2b** (step 1) is followed by the decomposition of the intermediate 1,3,4-thiadiazoline **6'** (step 2) giving rise to thiocarbonyl ylide **7'**. The latter undergoes competitive 1,5- or 1,3-electrocylizations (step 3).

Considering that thiadiazolines with bulky substituents are rather stable compounds (in particular, those derived from thioketone **2b** [18,30]), it seems difficult to predict in advance whether step 1 or step 2 determines the reaction rate. In this respect, the relative energy of the stationary points on the potential energy surface for cycloaddition of diazo compounds **1a–f** with cycloaliphatic thioketone **2b** were calculated (Table 2). All calculations refer to the gas phase, since all reactions of DDC **1** with the thioxocyclobutanedione **2b** were carried out under solvent-free conditions [20].

**Table 2:** Calculated relative energies for the 1,3-dipolar cycloadditions of diazodicarbonyl compounds **1a–f** with thioketone **2b** on the potential energy surface (in kcal·mol^−1^).

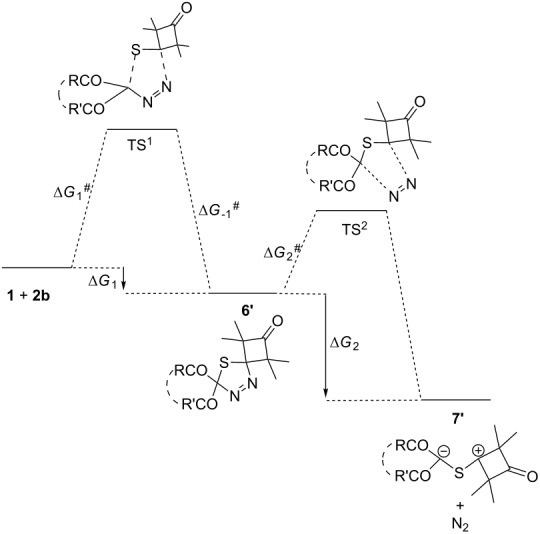							

Entry	Diazocompound	Δ*G*_1_^#^	Δ*G*_1_	Δ*G*_2_^#^	Δ*G*_2_	Δ*G*_1–7’_ = Δ*G*_1_ + Δ*G*_2_	*k***_1a_**/*k*_x_ at 25 °C^a^

1	**1a**; Me, Me	30.7 (36.2)	0.6 (12.9)	26.0 (20.5)	−12.8 (−22.6)	−12.2 (−9.7)	1 (1)
2	**1b**; Ph, Ph	29.3 (34.7)	-4.2 (8.1)	25.8 (20.7)	−8.3 (−17.7)	−12.5 (−9.6)	0.1 (0.08)
3	**1c**; Me, OMe	31.2 (36.8)	2.1 (13.9)	22.6 (17.3)	−14.8 (−24.4)	−12.7 (−10.5)	2.3 (2.5)
4	**1d**; OMe, OMe	30.8 (36.2)	-1.1 (10.5)	23.6 (18.4)	−13.3 (−22.7)	−14.4 (−12.2)	1.4 (0.9)
5	**1e**; (CH_2_)_3_	31.9 (37.3)	5.6 (17.5)	16.0 (10.7)	−22.9 (−33.2)	−17.3 (−15.7)	7.7 (6.2)
6	**1f**; (CH_2_)_2_	33.8 (39.4)	4.6 (15.9)	14.8 (10.0)	−24.2 (−33.6)	−19.6 (−17.7)	210 (210)

^a^The details of *k***_1a_**/*k*_x_ calculations are given in the Computational Details section.

The driving force behind the DDC **1a–d** reactions with thiocyclobutanedione **2b**, similar to thiobenzophenone (**2a**), are the significant negative values of the Gibbs free energy change of the 1,3,4-thiodiazoline **6’** decomposition process to produce thiocarbonyl ylide **7’** (Δ*G*_2_). This results in the overall negative total value of Gibbs free energy calculated according to the equation: Δ*G*_1–7’_ = Δ*G*_1_ + Δ*G*_2_.

The lower relative energy of the 1,3,4-thiadiazoline **6'** decomposition products, as well as the smaller activation energy of the second step of the process (Δ*G*_2_^#^ < Δ*G*_1_^#^), result in the formation of thiocarbonyl ylide **7'**. Since the activation energy of the cycloaddition step is larger than that of step 2 (the formation of C=S ylide), it is evident that step 1 determines the reaction rate. Furthermore, based on the character of the minimum energy transition states TS^1^, and by analogy with reactions of thiobenzophenone (**2a**), it is plausible to suggest that cycloadditions of DDC **1** with **2b** also proceed via a concerted pathway.

The calculated activation energy of DDC **1a–f** cycloadditions with thioketone **2b** correlate well with the experimental results, where the lowest values of Δ*G*_1_^#^ (30.7–31.2 kcal·mol^−1^; Table 2) are related to the most reactive of acyclic diazoadicarbonyl compounds **1a–d** [20] in this process. Carbocyclic diazodiketone **1e** with Δ*G*_1_^#^ = 31.9 kcal·mol^−1^ (PBE1PBE, Table 2, entry 5), in a similar way as the reaction with thiobenzophenone (**2a**), would not easily react at room temperature with thioketone **2b**. The largest Δ*G*_1_^#^ = 33.8 kcal·mol^−1^ (PBE1PBE, Table 2, entry 6) corresponds to the lowest reaction rate of the carbocyclic diazodiketone **1f**, which produces neither related oxathiole **3’f** nor any other product in this reaction [20]. Apparently, the predicted reaction pathway stems from the unfavorable 1,3-cycloaddition process of the *Z*,*Z*-locked conformation of carbocyclic diazodiketones **1e**,**f**.

### Transformations of thiocarbonyl ylides **7** and **7'**

In order to evaluate the correlation between the structure of thiocarbonyl ylides **7**, **7'** and the direction of their electrocyclizations (1,5- or 1,3-dipolar electrocyclization), the relative energies of the stationary points on the potential energy surface of both reaction pathways were calculated (Table 3) for the electrocyclizations of transient species **7** and **7'**.

**Table 3:** Calculated relative energies for competitive 1,5- and 1,3-electrocyclizations of thiocarbonyl ylides **7** and **7'** on the potential energy surface (in kcal·mol^−1^).^a^

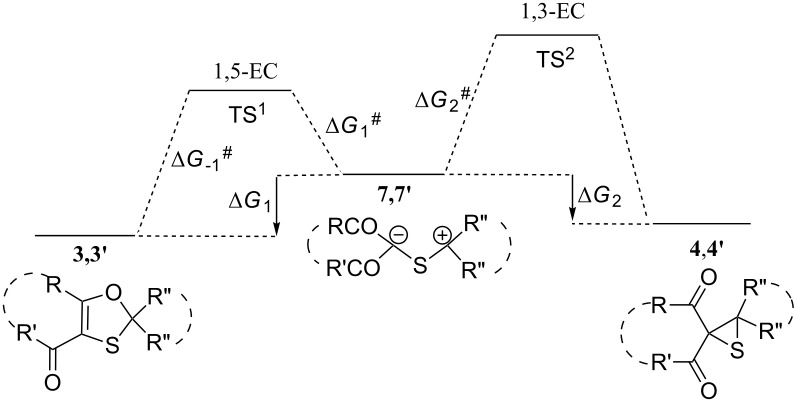						

Entry	Ylide **7**, **7'** (R, R')	Δ*G*_1_^#^ 1,5-EC	Δ*G*_1_ 1,5-EC	Δ*G*_-1_^#^ retro-1,5-EC	Δ*G*_2_^#^ 1,3-EC	Δ*G*_2_ 1,3-EC

1	**7a** (Me, Me)	2.8	−19.5	22.4	15.6	−15.0
2	**7b** (Ph, Ph)	6.1	−22.7	28.8	13.6	−14.2
3	**7c** (Me, OMe)	2.7	−19.9	22.6	14.3	−13.1
4	**7d** (2OMe)	8.9	−6.8	15.6	13.5	−16.4
7	**7'a** (Me, Me)	2.8	−29.7	32.5	17.5	−22.1
8	**7'b** (Ph, Ph)	3.3	−33.0	36.4	14.6	−29.0
9	**7'c** (Me, OMe)	2.4	−30.8	33.2	16.0	−23.0
10	**7'd** (2OMe)	8.7	−15.2	23.8	15.1	−26.6

^a^PBE1PBE functional was solely used for calculations.

The negative values of the Gibbs free energy change for 1,5- and 1,3-electrocyclizations of the ylides **7** and **7'** (Δ*G*_1_ and Δ*G*_2_, respectively), indicates that the reaction is thermodynamically allowed in both directions. However, based on the relative values of Δ*G*_1_ and Δ*G*_2_ for thiocarbonyl ylides **7a–c** and **7'a**–**c** (Table 3, entries 1–3 and 7–9), the 1,5-electrocyclization leading to 1,3-oxathioles **3a**–**c** and **3'a**–**c** is a thermodynamically more favorable process. However, in the case of the diazomalonate derivatives **7d** and **7’d** (Table 3, entries 4 and 10), the 1,3-electrocyclization process is thermodynamically preferable, resulting in the formation of thiiranes **4d** and **4'd**. At the same time, from the kinetic point of view, the 1,5-electrocyclization process is more efficient because the activation energy for the 1,5-electrocyclization (Δ*G*_1_^#^) is usually smaller than for the 1,3-electrocyclization (Δ*G*_2_^#^).

The obtained computation results are in good agreement with the experimental observations, which demonstrate that at 20 °C, due to a kinetic reaction control, 1,3-oxathioles **3** and **3’** are in most cases the major products of the multistep reactions of diazo compounds **1** with thioketones **2** [14–15,19–20]. At the same time, at elevated temperatures (80 °C), where the reaction is thermodynamically controlled, the lowest energy reaction products are expected to be formed. Indeed, the reaction of diazo compounds **1a–c** with thioketones **2a** and **2b** results in the formation of oxathioles **3a–c** and **3’a–c**, while with diazomalonate **1d** the thiiranes **4d**, **4’d** and alkenes **5d**, **5’d**, the desulfurization products of thiiranes are the principal reaction products [20].

**Scheme 3:**
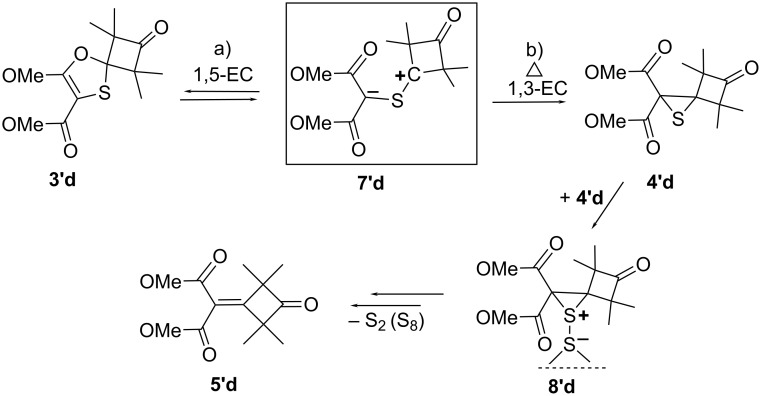
Reactions of the intermediate thiocarbonyl ylide **7'd** via competative 1,5-EC (a) or 1,3-EC (b) followed by desulfurization of thiirane **4'd** into alkene **5'd**.

To understand the mechanism of alkene **5’d** formation and the effect of temperature on this process, the relative energy of the stationary points on the potential energy surface for the transformation of ylide **7'd** into 1,3-oxathiole **3’d** (path a), and further to alkene **5’d** (path b) via the intermediate thiirane **4’d,** were calculated (Scheme 3).

It was assumed that the mechanism includes the following steps [12,31–32]: 1,5-electrocyclization of C=S ylide **7’d** (step a), 1,3-electrocyclization of C=S ylide **7’d** (step b), disproportionation of the thiirane **4’d** dimer into thiirane S-sulfide **8’d** and alkene **5’d** (step c). The subsequent decomposition of thiirane-S-sulfide **8’d** (step d) and the tetramerization of the extruded S_2_ molecule results in the formation of the most thermodynamically stable, rhombic modification of sulfur S_8_ (step e). The obtained computational results for this process are summarized in Table 4 (the data for the Gibbs free energy change for the tetramerization of the S_2_ molecule into S_8_ were taken from the literature [33]).

**Table 4:** Computed relative energies for conversion of thiocarbonyl ylide **7'd** into 1,3-oxathiole **3’d** and alkene **5'd** on the potential energy surface (in kcal·mol^−1^).

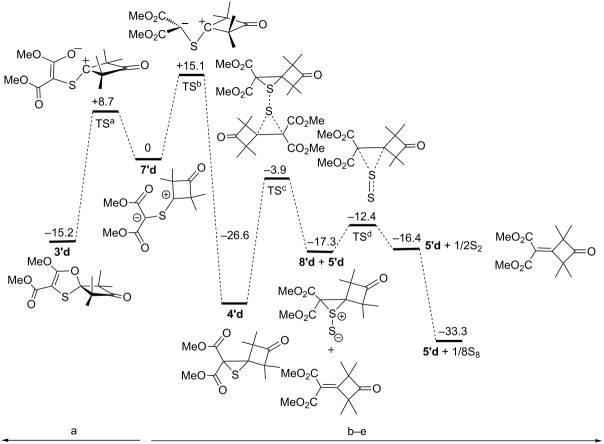					

Step	a	b	c	d	e

	**7’d** → **3’d**	**7’e** → **4’d**	**4’d** → **8’d + 5’d**	**8’d** → **5’d + S_2_**	**S_2_** → **S_8_**
Δ*G*_i_	−15.2	−26.6	9.3	0.9	−16.9
Δ*G*_i_^#^	8.7	15.1	22.7	4.9	–
Δ*G*_-i_^#^	23.8	41.7	13.4	4.0	–

According to the calculations, the Gibbs free energy changes for the 1,5-electrocyclization of ylide **7’d** to 1,3-oxathiole **3’d** (Δ*G*_1_), and/or 1,3-electrocyclization of ylide **7’d** to thiirane **4’d**, Δ*G*_b_, are equal to −15.2 and −26.6 kcal·mol^−1^, respectively, whereas the relevant values of the activation energy for the steps a (Δ*G*_a_^#^) and b (Δ*G*_b_^#^) are equal to 8.7 and 15.1 kcal·mol^−1^, respectively. As a result, the thiirane derivative **4’d** is thermodynamically more stable than 1,3-oxathiole **3’d**, and therefore, **4’d** and the corresponding alkene **5’d** resulting from desulfurization are the main products formed at elevated temperatures when the reaction is thermodynamically controlled.

At the same time, the lower activation barrier of 1,5-electrocyclization (Δ*G*_a_^#^ = 8.7 kcal·mol^−1^) as compared to 1,3-electrocyclization (Δ*G*_b_^#^ = 15.1 kcal·mol^−1^) results in the dominance of oxathiole **3’d**, which is preferentially formed in the reaction mixture at room temperature when the reaction is kinetically controlled. Due to the relatively low activation barrier of the 1,3-oxathiole **3’d** ring opening back into ylide **7’d** (Δ*G***_-_**_a_^#^ = 23.8 kcal·mol^−1^), the thermodynamically more stable alkene **5’d** is accumulated in the reaction mixture with time even at room temperature.

As for a disproportionation mechanism of two thiirane molecules **4’d** (step c) into thiirane S-sulfide **8’d** and alkene **5’d**, followed by the decomposition of the transient thiirane S-sulfide **8’d** (step d), it is most likely that it follows the pathway proposed for the spontaneous desulfurization of matrix-isolated oxathiiranes [32].

## Conclusion

The quantum-chemical calculations show that the initial step of the 1,3-dipolar cycloaddition of acyclic 2-diazo-1,3-dicarbonyl compounds with the C=S bonds of “superdipolarophilic” thioketones proceeds with *Z*,*E*-conformers of DDC via a concerted mechanism and adequately agree with experimental data. At room temperature, the 1,5-electrocyclization of the intermediate ylides into the 1,3-oxathiole derivatives is a thermodynamically and kinetically favorable process for DDC with at least one electron-withdrawing acyl group. At higher temperatures, however, due to the entropy contribution, the dominating process becomes the 1,3-cyclization followed by desulfurization of the obtained thiiranes, which leads to the corresponding alkenes. In the case of the C=S ylides bearing two alkoxycarbonyl groups, the latter pathway clearly dominates. The reversibility of the 1,5-electrocyclization of the transient C=S ylide, generated from diazomalonate **1d** and thione **2b**, is attributed to a relatively low activation barrier (Δ*G*^#^ = 23.8 kcal·mol^−1^) for the ring opening of the 1,3-oxathiole back to thiocarbonyl ylide. Carbocyclic diazodiketones are practically unreactive at room temperature due to the locked *Z*,*Z*-configuration of the diazo molecule, which is unfavorable for initiating the cycloaddition step.

## Computational Details

The geometry optimization of reagents, intermediates, products, and transition states, and the calculations of the molecular orbital energies, vibrational frequencies, and ground-state IRC scans, were all performed at the B3LYP and PBE1PBE theory level employing the 6-31G(d) basis set using the GAUSSIAN 09 program package [34]. The calculated energies for the B3LYP and PBE1PBE functionals were found to be consistent. The thermochemical parameters were calculated for 298.15 K and all are given in kcal·mol^−1^. For the reactions of diazo compounds **1a–f** with thiobenzophenone (**2a**), the solvent was simulated using the polarizable continuum model (PCM) [22]. All calculations for the reactions of diazo compounds **1a**,**c**,**d** with thiocyclobutanedione **2b** were performed in the gas phase. To characterize the nature of the stationary points, the corresponding vibrational frequencies were calculated. The optimized structure of the reagents, intermediates, and products was characterized by the absence of an imaginary frequency, and transition states containing only one imaginary frequency component. The calculated IRC paths demonstrated that the stationary points are effectively connected to each other. The relative reaction rates were estimated using the Arrenius equation (Equation 1), where *k* is the reaction rate, *A* is the prefactor, Δ*G*^#^ is the activation energy, *R* is the universal gas constant, and *T* is the temperature.

[1]
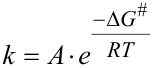


[2]
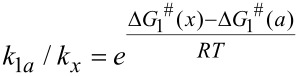


The relative reaction rates of DDC **1b–f**, as compared to the most reactive diazoacetylacetone **1a**, were estimated by Equation 2 (Table 1 and Table 2).

## Supporting Information

File 1 Details of computational studies: cartesian coordinates, computed geometries of compounds, transition states, and computed total energies.
