# Supplementary material for: On the strong difference in reactivity of acyclic and cyclic diazodiketones with thioketones: experimental results and quantum-chemical interpretation
Source: Beilstein J Org Chem. 2015 Apr 20;11:504–13. doi: 10.3762/bjoc.11.57 (PMC4419510; doi:10.3762/bjoc.11.57)
Supplement: File 1 — Details of computational studies: cartesian coordinates, computed geometries of compounds, transition states, and computed total energies. [file Beilstein_J_Org_Chem-11-504-s001.pdf]

Supporting Information

for

On the strong difference in reactivity of acyclic and cyclic diazodiketones with thioketones: experimental results and quantum-chemical interpretation

Andrey S. Mereshchenko[1], Alexey V. Ivanov[1], Viktor I. Baranovskii[1], Grzegorz Mloston[2,*], Ludmila L. Rodina[1] and Valerij A. Nikolaev [1,*]

Address: [1]Department of Chemistry, Saint-Petersburg State University, University prosp., 26, 198504, Saint Petersburg, Russia and [2]Faculty of Chemistry, University of Łódź, Tamka 12, 91-403 Łódź, Poland

Email: Grzegorz Mloston - gmloston@uni.lodz.pl; Valerij A. Nikolaev - valerij.nikolaev@gmail.com

*Corresponding author

Details of computational studies: Cartesian coordinates, computed geometries of compounds, transition states, and computed total energies.

Table of Contents

Cartesian coordinates (in Å) and energies (in Hartree/particle) for:

Calculated at the PBE1PBE/6-31G(d) level of theory

1. 2-diazo-1,3-dicarbonyl compounds....S3
2. Thioketones....S9
3. Transition states for cycloadditions of diazo compounds **1** to thiobenzophenone (**2a**)....S10
4. Thiodiazolines **6** obtained from diazo compounds **1** and thiobenzophenone (**2a**)....S16
5. Transition states for decompositions of thiadiazolines **6**....S22
6. Thiodiazolines **6** decomposition products....S28
7. Transition states for 1,5-electrocyclizations of C=S-ylides **7** to oxathioles **3**....S34
8. Oxathioles **3**....S38
9. Transition states for 1,3-electrocyclizations of C=S-ylides **7** to thiiranes **4**....S42
10. Thiiranes **4**....S46
11. Transition states for cycloadditions of diazo compounds **1** to aliphatic thioketone **2b**....S50
12. Thiodiazolines **6'** obtained from diazo compounds **1** and aliphatic thioketone **2b**....S56
13. Transition states for decompositions of thiadiazolines **6'**....S62
14. Thiodiazolines **6'** decomposition products....S68
15. Transition states for 1,5-electrocyclizations of C=S-ylides **7'** to oxathioles **3'**....S74
16. Oxathioles **3'**....S78
17. Transition states for 1,3-electrocyclizations of C=S-ylides **7'** to thiiranes **4'**....S82
18. Thiiranes **4'**....S86
19. Products and transition states for conversion of oxathiole **3'd** to alkene **5'd**....S90

Calculated at the B3LYP/6-31G(d) level of theory

20. 2-diazo-1,3-dicarbonyl compounds....S95
21. Thioketones....S101
22. Transition states for cycloadditions of diazo compounds **1** to thiobenzophenone (**2a**)....S102
23. Thiodiazolines **6** obtained from diazo compounds **1** and thiobenzophenone (**2a**)....S108
24. Transition states for decompositions of thiadiazolines **6**....S114
25. Thiodiazolines **6** decomposition products....S120
26. Transition states for cycloadditions of diazo compounds **1** to aliphatic thioketone **2b**....S126
27. Thiodiazolines **6'** obtained from diazo compounds **1** and aliphatic thioketone **2b**....S132
28. Transition states for decompositions of thiadiazolines **6'**....S138
29. Thiodiazolines **6'** decomposition products....S144

Calculated at the PBE1PBE/6-31G(d) level of theory

1. **2-diazo-1,3-dicarbonyl compounds**

Gas phase, 6-31G(d), PBE1PBE

3-diazopentane-2,4-dione (1a)

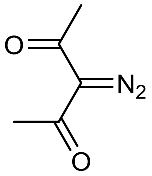

Sum of electronic and zero-point Energies= -453.428168
Sum of electronic and thermal Energies= -453.418491
Sum of electronic and thermal Enthalpies= -453.417547
Sum of electronic and thermal Free Energies= -453.463367

Standard orientation. Coordinates (Angstroms):

C	1.943066000	-1.440417000	-0.000038000
H	1.570793000	-1.983991000	-0.873328000
H	1.570896000	-1.984019000	0.873278000
H	3.034307000	-1.414224000	-0.000102000
C	1.449513000	-0.021593000	0.000009000
C	-0.023838000	0.193969000	0.000124000
C	-1.110568000	-0.798308000	0.000125000
C	-2.530627000	-0.276925000	0.000028000
H	-2.727487000	0.340200000	-0.884669000
H	-3.210235000	-1.130138000	-0.000123000
H	-2.727656000	0.340022000	0.884811000
O	2.196857000	0.939043000	0.000011000
O	-0.865735000	-1.991105000	-0.000110000
N	-0.354295000	1.469633000	0.000161000
N	-0.577829000	2.574409000	-0.000239000

2-diazo-1,3-diphenylpropane -1,3-dione (1b)

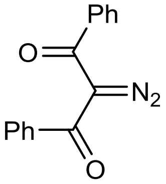

Sum of electronic and zero-point Energies= -836.339790
Sum of electronic and thermal Energies= -836.324251
Sum of electronic and thermal Enthalpies= -836.323307
Sum of electronic and thermal Free Energies= -836.385151

Standard orientation. Coordinates (Angstroms):

C	-0.728465000	-0.486104000	-0.033826000
C	-0.043110000	0.787418000	-0.332483000
C	1.362884000	1.232868000	-0.079768000
O	-0.111527000	-1.530364000	0.094089000
O	1.573787000	2.431002000	0.036228000
N	-0.782453000	1.796254000	-0.759488000
N	-1.338401000	2.687549000	-1.166243000
C	-2.219070000	-0.478499000	0.108301000
C	-2.914825000	-1.591415000	-0.375207000
C	-2.918326000	0.536661000	0.768079000
C	-4.294620000	-1.667208000	-0.240164000
H	-2.354245000	-2.389646000	-0.852276000
C	-4.298605000	0.449062000	0.919589000
H	-2.386829000	1.381721000	1.197920000
C	-4.988793000	-0.645426000	0.405946000
H	-4.830652000	-2.527143000	-0.631944000
H	-4.834108000	1.234573000	1.445218000
H	-6.067839000	-0.707603000	0.517771000
C	2.474148000	0.253262000	0.025359000
C	3.557273000	0.616128000	0.835429000
C	2.537349000	-0.921337000	-0.728452000
C	4.672915000	-0.204664000	0.920078000

H	3.504486000	1.549147000	1.387966000
C	3.668385000	-1.727580000	-0.661288000
H	1.708594000	-1.203521000	-1.365917000
C	4.729800000	-1.377958000	0.168941000
H	5.502906000	0.072852000	1.563876000
H	3.717979000	-2.634939000	-1.256506000
H	5.605956000	-2.018414000	0.228271000

Methyl 2-diazo-3-oxobutanoate (1c)

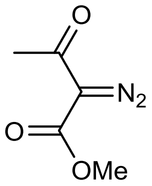

Sum of electronic and zero-point Energies= -528.577275
Sum of electronic and thermal Energies= -528.566646
Sum of electronic and thermal Enthalpies= -528.565702
Sum of electronic and thermal Free Energies= -528.614048

Standard orientation. Coordinates (Angstroms):

C	0.873603000	-0.449967000	-0.000315000
C	-0.416706000	0.238929000	-0.000110000
C	-1.778037000	-0.346911000	-0.000101000
O	1.012352000	-1.652710000	-0.000226000
O	-2.749135000	0.387529000	0.000352000
N	-0.401059000	1.557650000	0.000079000
N	-0.457722000	2.680974000	-0.000277000
O	1.894482000	0.427091000	0.000074000
C	3.192604000	-0.164442000	0.000279000
H	3.892978000	0.670503000	0.000604000
H	3.332727000	-0.783432000	0.889995000
H	3.333153000	-0.783100000	-0.889601000
C	-1.886141000	-1.846548000	0.000023000
H	-1.387105000	-2.275076000	0.874272000
H	-2.946216000	-2.105767000	0.000331000
H	-1.387599000	-2.275143000	-0.874474000

Dimethyl diazomalonate (1d)

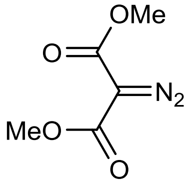

Sum of electronic and zero-point Energies= -603.719501
Sum of electronic and thermal Energies= -603.707835
Sum of electronic and thermal Enthalpies= -603.706891
Sum of electronic and thermal Free Energies= -603.758204

Standard orientation. Coordinates (Angstroms):

C	-1.447610000	0.278594000	-0.000218000
C	0.014245000	0.446222000	-0.000271000
C	1.068004000	-0.577444000	-0.000469000
O	-2.214397000	1.218872000	0.000215000
O	0.886279000	-1.769227000	-0.000267000
N	0.394369000	1.707923000	-0.000069000
N	0.708494000	2.788626000	0.000035000
O	-1.807207000	-1.002468000	-0.000099000
C	-3.217007000	-1.220666000	0.000281000
H	-3.340272000	-2.303302000	0.000329000
H	-3.674449000	-0.780137000	0.889944000
H	-3.674919000	-0.780158000	-0.889149000
O	2.284785000	0.006377000	0.000056000
C	3.380808000	-0.905816000	0.000408000
H	3.353501000	-1.540272000	-0.889197000

H	4.276963000	-0.285336000	0.000803000
H	3.352822000	-1.540417000	0.889889000

__2-Diazocyclohexane-1,3-dione (1e)__

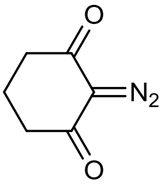

Sum of electronic and zero-point Energies= -491.494100
Sum of electronic and thermal Energies= -491.485445
Sum of electronic and thermal Enthalpies= -491.484501
Sum of electronic and thermal Free Energies= -491.528133

Standard orientation. Coordinates (Angstroms):

C	0.085108000	1.318906000	-0.068629000
C	-0.562547000	-0.000001000	-0.026676000
C	0.085109000	-1.318906000	-0.068625000
N	-1.880541000	-0.000001000	0.052293000
N	-3.001575000	0.000000000	0.116953000
O	-0.546256000	-2.358374000	-0.033336000
O	-0.546258000	2.358374000	-0.033331000
C	1.594542000	-1.263124000	-0.178491000
H	1.842236000	-1.316768000	-1.249307000
H	1.986360000	-2.175088000	0.281712000
C	2.195552000	0.000002000	0.433589000
H	3.281111000	0.000002000	0.290164000
H	2.022787000	0.000004000	1.517635000
C	1.594541000	1.263124000	-0.178498000
H	1.986360000	2.175091000	0.281699000
H	1.842233000	1.316760000	-1.249315000

__2-Diazocyclopentane1,3-dione (1f)__

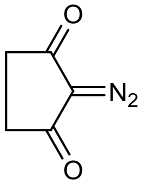

Sum of electronic and zero-point Energies= -452.255438
Sum of electronic and thermal Energies= -452.247702
Sum of electronic and thermal Enthalpies= -452.246758
Sum of electronic and thermal Free Energies= -452.288814

Standard orientation. Coordinates (Angstroms):

C	-0.362974000	1.225054000	-0.000265000
C	0.442114000	0.000076000	0.000108000
C	-0.362662000	-1.225114000	0.000376000
N	1.748120000	0.000177000	0.000022000
N	2.874556000	0.000291000	-0.000130000
O	0.027168000	-2.370162000	-0.000056000
O	0.026547000	2.370207000	0.000154000
C	-1.820192000	-0.765075000	-0.000087000
H	-2.311093000	-1.196274000	0.878488000
H	-2.310516000	-1.196179000	-0.879031000
C	-1.820389000	0.764639000	-0.000039000
H	-2.311143000	1.195659000	-0.878782000
H	-2.311081000	1.195673000	0.878737000

Benzene (PCM), 6-31G(d), PBE1PBE

3-diazopentane-2,4-dione (1a)

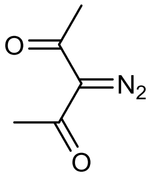

Sum of electronic and zero-point Energies= -453.432002
Sum of electronic and thermal Energies= -453.422383
Sum of electronic and thermal Enthalpies= -453.421439
Sum of electronic and thermal Free Energies= -453.467055

Standard orientation. Coordinates (Angstroms):

C	1.942430000	-1.439213000	-0.000028000
H	1.571851000	-1.982201000	-0.874343000
H	1.571889000	-1.982255000	0.874270000
H	3.033663000	-1.413337000	-0.000050000
C	1.446099000	-0.022070000	0.000023000
C	-0.023595000	0.192938000	0.000083000
C	-1.111175000	-0.798088000	0.000025000
C	-2.529203000	-0.276745000	0.000035000
H	-2.724392000	0.340460000	-0.884828000
H	-3.211336000	-1.127932000	-0.000050000
H	-2.724431000	0.340314000	0.884990000
O	2.196274000	0.939451000	0.000063000
O	-0.863992000	-1.991917000	-0.000089000
N	-0.354136000	1.469775000	0.000125000
N	-0.577698000	2.573619000	-0.000212000

2-diazo-1,3-diphenylpropane -1,3-dione (1b)

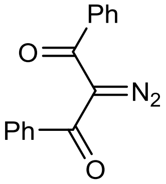

Sum of electronic and zero-point Energies= -836.344528
Sum of electronic and thermal Energies= -836.328995
Sum of electronic and thermal Enthalpies= -836.328051
Sum of electronic and thermal Free Energies= -836.389684

Standard orientation. Coordinates (Angstroms):

C	-0.717926000	-0.467621000	0.015488000
C	-0.042115000	0.804093000	-0.306201000
C	1.363654000	1.247172000	-0.068234000
O	-0.086847000	-1.497921000	0.190188000
O	1.585375000	2.445472000	0.040359000
N	-0.784995000	1.805833000	-0.744832000
N	-1.342764000	2.690800000	-1.161128000
C	-2.210243000	-0.476988000	0.122815000
C	-2.883992000	-1.599361000	-0.371021000
C	-2.933387000	0.534944000	0.762168000
C	-4.265899000	-1.688256000	-0.265355000
H	-2.308374000	-2.393887000	-0.836308000
C	-4.315840000	0.433603000	0.884677000
H	-2.420373000	1.387227000	1.199900000
C	-4.983883000	-0.670587000	0.361620000
H	-4.784746000	-2.554765000	-0.665432000
H	-4.870167000	1.215974000	1.395034000
H	-6.064278000	-0.743322000	0.450789000
C	2.468442000	0.259734000	0.024748000
C	3.551531000	0.591504000	0.847756000
C	2.522349000	-0.896274000	-0.758646000
C	4.659341000	-0.242531000	0.914765000
H	3.506604000	1.507923000	1.428276000
C	3.645944000	-1.714149000	-0.710676000
H	1.693959000	-1.150944000	-1.409052000
C	4.708030000	-1.395871000	0.132417000
H	5.489122000	0.009323000	1.569137000

H	3.690144000	-2.605350000	-1.330179000
H	5.578147000	-2.045486000	0.177260000

Methyl 2-diazo-3-oxobutanoate (1c)

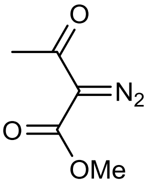

Sum of electronic and zero-point Energies= -528.580797
Sum of electronic and thermal Energies= -528.570194
Sum of electronic and thermal Enthalpies= -528.569250
Sum of electronic and thermal Free Energies= -528.617502

Standard orientation. Coordinates (Angstroms):

C	0.873143000	-0.452997000	0.000308000
C	-0.417348000	0.237436000	-0.000194000
C	-1.777109000	-0.343549000	-0.000608000
O	1.007840000	-1.657293000	0.000070000
O	-2.748633000	0.394364000	-0.000280000
N	-0.397865000	1.556825000	-0.000211000
N	-0.448061000	2.679898000	0.000693000
O	1.892225000	0.421608000	0.000136000
C	3.195243000	-0.164962000	0.000068000
H	3.891140000	0.673290000	-0.000090000
H	3.337862000	-0.781055000	0.891030000
H	3.337692000	-0.781242000	-0.890791000
C	-1.892331000	-1.841866000	-0.000082000
H	-1.396523000	-2.271381000	0.875536000
H	-2.953072000	-2.098539000	0.000081000
H	-1.396658000	-2.271939000	-0.875500000

Dimethyl diazomalonate (1d)

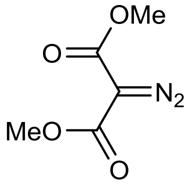

Sum of electronic and zero-point Energies= -603.723901
Sum of electronic and thermal Energies= -603.712187
Sum of electronic and thermal Enthalpies= -603.711243
Sum of electronic and thermal Free Energies= -603.762743

Standard orientation. Coordinates (Angstroms):

C	-1.446659000	0.279649000	-0.000613000
C	0.014376000	0.444864000	-0.000142000
C	1.068226000	-0.578629000	0.000131000
O	-2.212086000	1.222945000	-0.000157000
O	0.882382000	-1.771747000	-0.000181000
N	0.397028000	1.706345000	0.000079000
N	0.713326000	2.785879000	0.000219000
O	-1.810376000	-0.998988000	-0.000161000
C	-3.222299000	-1.218614000	0.000177000
H	-3.344807000	-2.301081000	0.000521000
H	-3.678623000	-0.779601000	0.890718000
H	-3.678955000	-0.780125000	-0.890451000
O	2.282330000	0.002304000	0.000241000
C	3.385116000	-0.905074000	0.000209000
H	3.361244000	-1.537422000	-0.890592000
H	4.276939000	-0.279046000	0.000304000
H	3.361161000	-1.537575000	0.890901000

2-Diazocyclohexane-1,3-dione (1e)

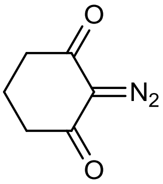

Sum of electronic and zero-point Energies: -491.498577
Sum of electronic and thermal Energies: -491.489890
Sum of electronic and thermal Enthalpies: -491.488946
Sum of electronic and thermal Free Energies: -491.532674

Standard orientation. Coordinates (Angstroms):

C	0.086594000	1.317454000	-0.068146000
C	-0.561373000	-0.000004000	-0.024574000
C	0.086616000	-1.317447000	-0.068251000
N	-1.879763000	-0.000016000	0.053182000
N	-3.000500000	-0.000027000	0.117380000
O	-0.548419000	-2.357284000	-0.035249000
O	-0.548467000	2.357278000	-0.035276000
C	1.594170000	-1.263049000	-0.177937000
H	1.840518000	-1.316654000	-1.249001000
H	1.988110000	-2.173833000	0.282722000
C	2.195550000	0.000024000	0.433457000
H	3.280122000	0.000038000	0.285842000
H	2.025522000	0.000035000	1.517600000
C	1.594136000	1.263067000	-0.177962000
H	1.988104000	2.173876000	0.282622000
H	1.840405000	1.316612000	-1.249048000

2-Diazocyclopentane-1,3-dione (1f)

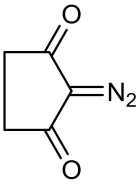

Sum of electronic and zero-point Energies= -452.260019
Sum of electronic and thermal Energies= -452.252197
Sum of electronic and thermal Enthalpies= -452.251253
Sum of electronic and thermal Free Energies= -452.294506

Standard orientation. Coordinates (Angstroms):

C	-0.364170000	1.223053000	-0.000358000
C	0.440997000	0.000086000	0.000144000
C	-0.363818000	-1.223123000	0.000502000
N	1.747937000	0.000203000	0.000050000
N	2.873632000	0.000328000	-0.000177000
O	0.028457000	-2.369702000	-0.000079000
O	0.027754000	2.369754000	0.000187000
C	-1.819524000	-0.765042000	-0.000125000
H	-2.311010000	-1.194604000	0.878833000
H	-2.310185000	-1.194408000	-0.879642000
C	-1.819748000	0.764550000	-0.000027000
H	-2.311006000	1.193875000	-0.879226000
H	-2.310887000	1.193870000	0.879249000

2. Thioketones

Benzene (PCM), 6-31G(d), PBE1PBE

Thiobenzophenone (2a)

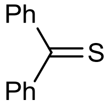

Sum of electronic and zero-point Energies: -898.648303
Sum of electronic and thermal Energies: -898.637300
Sum of electronic and thermal Enthalpies: -898.636356
Sum of electronic and thermal Free Energies: -898.686421

Standard orientation. Coordinates (Angstroms):

S	-0.000003000	2.575363000	-0.000012000
C	-1.265162000	0.162518000	-0.028988000
C	-1.352702000	-1.050528000	-0.732669000
C	-2.417244000	0.659545000	0.600357000
C	-2.559692000	-1.735577000	-0.814434000
H	-0.476545000	-1.438645000	-1.243002000
C	-3.614036000	-0.039459000	0.538365000
H	-2.351689000	1.595653000	1.146272000
C	-3.690797000	-1.236666000	-0.173551000
H	-2.615963000	-2.661027000	-1.380612000
H	-4.492035000	0.349610000	1.046227000
H	-4.631158000	-1.778374000	-0.227930000
C	1.265155000	0.162522000	0.029046000
C	1.352740000	-1.050518000	0.732731000
C	2.417200000	0.659552000	-0.600364000
C	2.559740000	-1.735558000	0.814437000
H	0.476613000	-1.438638000	1.243113000
C	3.614000000	-0.039443000	-0.538431000
H	2.351611000	1.595657000	-1.146281000
C	3.690807000	-1.236644000	0.173490000
H	2.616047000	-2.661003000	1.380619000
H	4.491970000	0.349628000	-1.046341000
H	4.631175000	-1.778345000	0.227824000
C	-0.000005000	0.923535000	0.000061000

Gas phase, 6-31G(d), PBE1PBE

2,2,4,4-Tetramethylcyclobutane-1-one-3-thione (2b)

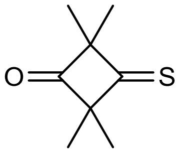

Sum of electronic and zero-point Energies: -784.665265
Sum of electronic and thermal Energies: -784.653265
Sum of electronic and thermal Enthalpies: -784.652320
Sum of electronic and thermal Free Energies: -784.702888

Standard orientation. Coordinates (Angstroms):

C	0.000005000	0.748165000	-0.000099000
C	-1.119758000	-0.292112000	-0.000012000
C	1.119754000	-0.292126000	-0.000019000
C	-0.000009000	-1.351126000	-0.000004000
C	-1.980834000	-0.285391000	1.262782000
H	-1.373159000	-0.301141000	2.173244000
H	-2.599351000	0.618001000	1.281175000
H	-2.632408000	-1.165199000	1.270007000
C	-1.980961000	-0.285444000	-1.262717000
H	-1.373380000	-0.301233000	-2.173241000
H	-2.632533000	-1.165255000	-1.269832000
H	-2.599486000	0.617943000	-1.281092000

C	1.980888000	-0.285408000	1.262731000
H	2.632450000	-1.165226000	1.269935000
H	2.599423000	0.617974000	1.281088000
H	1.373256000	-0.301139000	2.173221000
C	1.980899000	-0.285477000	-1.262768000
H	2.599428000	0.617907000	-1.281179000
H	2.632463000	-1.165293000	-1.269905000
H	1.373276000	-0.301270000	-2.173263000
O	-0.000017000	-2.551544000	-0.000291000
S	0.000015000	2.361237000	0.000176000

3. Transition states for cycloadditions of diazo compounds 1 to thiobenzophenone (2a)

Benzene (PCM), 6-31G(d), PBE1PBE

$TS_{1a+2a \rightarrow 6a}$

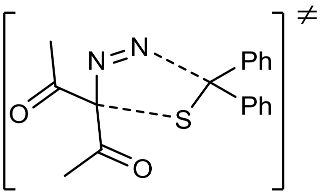

Imaginary Freq.: -389.50 cm^{-1}
Sum of electronic and zero-point Energies:-1352.053781
Sum of electronic and thermal Energies: -1352.032445
Sum of electronic and thermal Enthalpies: -1352.031501
Sum of electronic and thermal Free Energies: -1352.104929

Standard orientation. Coordinates (Angstroms):

N	1.343793000	0.010794000	1.390168000
N	0.202926000	-0.018868000	1.567790000
C	-0.681826000	-0.046329000	-0.394808000
S	0.733369000	-0.106523000	-1.336638000
C	2.266039000	-0.001818000	0.401146000
C	2.961061000	-1.341110000	0.287587000
C	3.969469000	-1.513303000	-0.806192000
C	2.377408000	2.535302000	0.805791000
C	2.936097000	1.319594000	0.120097000
O	3.859496000	1.363953000	-0.664676000
O	2.651301000	-2.229723000	1.056412000
C	-1.392980000	1.256584000	-0.211402000
C	-1.182475000	2.342435000	-1.071301000
C	-1.836657000	3.552701000	-0.864784000
C	-2.713564000	3.705163000	0.206547000
C	-2.933315000	2.633848000	1.069376000
C	-2.283094000	1.422626000	0.862302000
C	-1.464106000	-1.309575000	-0.227956000
C	-2.837081000	-1.334667000	-0.505841000
C	-3.560823000	-2.519906000	-0.409038000
C	-2.930542000	-3.697852000	-0.020445000
C	-1.565587000	-3.683149000	0.262215000
C	-0.838787000	-2.504436000	0.153889000
H	3.569626000	-1.182057000	-1.768995000
H	4.850706000	-0.896309000	-0.606417000
H	4.245884000	-2.568125000	-0.850464000
H	2.439781000	2.434236000	1.895055000
H	2.946676000	3.408889000	0.485530000
H	-0.505309000	2.227244000	-1.912317000
H	-1.665018000	4.377658000	-1.551030000
H	-3.224408000	4.650775000	0.366104000
H	-3.612028000	2.740717000	1.911263000
H	-2.455370000	0.595157000	1.544084000

H	-3.339105000	-0.423723000	-0.817096000
H	-4.622420000	-2.518728000	-0.641567000
H	-3.497066000	-4.621366000	0.062100000
H	-1.061885000	-4.595194000	0.570759000
H	0.225328000	-2.509882000	0.373893000
H	1.321877000	2.671111000	0.542600000

$TS_{1b+2a\rightarrow 6b}$

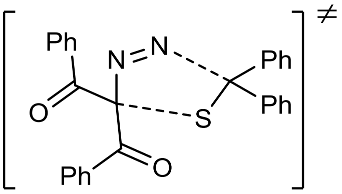

Imaginary Freq.: -378.56 cm^{-1}
Sum of electronic and zero-point Energies:-1734.965996
Sum of electronic and thermal Energies: -1734.938719
Sum of electronic and thermal Enthalpies: -1734.937775
Sum of electronic and thermal Free Energies: -1735.026301

Standard orientation. Coordinates (Angstroms):

N	-0.039754000	0.354530000	-1.460112000
N	0.883213000	-0.335026000	-1.542817000
C	1.118625000	-1.249419000	0.371690000
S	-0.227169000	-0.564231000	1.160108000
C	-1.029674000	0.710333000	-0.602080000
C	-2.313601000	-0.041020000	-0.947307000
C	-1.112443000	2.202001000	-0.311827000
O	-2.194107000	2.679830000	-0.025572000
O	-2.334792000	-0.674602000	-1.989393000
C	2.487186000	-0.711368000	0.650614000
C	2.789351000	-0.054988000	1.850591000
C	4.064270000	0.450840000	2.085471000
C	5.063827000	0.310951000	1.125441000
C	4.779142000	-0.346204000	-0.070081000
C	3.507299000	-0.856901000	-0.303290000
C	1.021331000	-2.667920000	-0.094886000
C	2.030640000	-3.586375000	0.224542000
C	1.928554000	-4.920334000	-0.159683000
C	0.824162000	-5.360673000	-0.882216000
C	-0.183031000	-4.455000000	-1.210414000
C	-0.089034000	-3.126085000	-0.817037000
H	2.013693000	0.055348000	2.602623000
H	4.277271000	0.949652000	3.027116000
H	6.059667000	0.704667000	1.310102000
H	5.550418000	-0.461734000	-0.826788000
H	3.293566000	-1.366922000	-1.237730000
H	2.894876000	-3.258999000	0.793967000
H	2.717986000	-5.616461000	0.111137000
H	0.747606000	-6.400398000	-1.188475000
H	-1.048956000	-4.783243000	-1.778889000
H	-0.885203000	-2.437038000	-1.081726000
C	-3.467916000	-0.044299000	-0.014337000
C	-4.648840000	-0.620879000	-0.500523000
C	-3.430314000	0.434088000	1.300525000
C	-5.774410000	-0.706925000	0.306801000
H	-4.661104000	-0.996400000	-1.518557000
C	-4.554187000	0.334431000	2.110660000
H	-2.530891000	0.886663000	1.701307000
C	-5.727726000	-0.230955000	1.615883000
H	-6.687610000	-1.147627000	-0.082796000
H	-4.513992000	0.702189000	3.131708000
H	-6.606150000	-0.300864000	2.251788000
C	0.091098000	3.062684000	-0.446064000
C	1.386114000	2.640089000	-0.131923000

C	-0.124935000	4.384329000	-0.859367000
C	2.452635000	3.525826000	-0.242740000
H	1.565186000	1.639199000	0.243334000
C	0.944861000	5.258942000	-0.987775000
H	-1.137785000	4.705279000	-1.082310000
C	2.236125000	4.830025000	-0.680187000
H	3.451920000	3.190729000	0.019814000
H	0.773633000	6.277512000	-1.324097000
H	3.072895000	5.516637000	-0.775917000

TS$_{1c+2a\rightarrow 6c}$

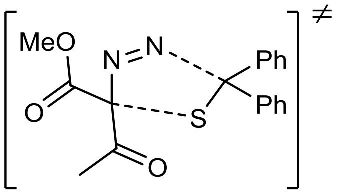

Imaginary Freq.: -391.49 cm^{-1}
Sum of electronic and zero-point Energies:-1427.200980
Sum of electronic and thermal Energies: -1427.178556
Sum of electronic and thermal Enthalpies: -1427.177611
Sum of electronic and thermal Free Energies: -1427.254247

Standard orientation. Coordinates (Angstroms):

N	-1.066883000	-0.601502000	-1.364200000
N	-0.005310000	-0.190045000	-1.549484000
C	0.842864000	0.114579000	0.403436000
S	-0.411304000	-0.522493000	1.359532000
C	-1.893425000	-0.978962000	-0.361885000
C	-2.035466000	-2.478610000	-0.250617000
C	-3.007099000	-0.027561000	-0.061633000
O	-3.870507000	-0.253273000	0.752907000
O	-1.401156000	-3.184650000	-1.008772000
C	0.968105000	1.594927000	0.236499000
C	0.342483000	2.493879000	1.109000000
C	0.454822000	3.866826000	0.919060000
C	1.190297000	4.371974000	-0.150549000
C	1.816409000	3.489797000	-1.027999000
C	1.709649000	2.117107000	-0.835732000
C	2.061626000	-0.729439000	0.206157000
C	3.334782000	-0.210443000	0.474908000
C	4.468949000	-1.008240000	0.350683000
C	4.354131000	-2.333671000	-0.056497000
C	3.092238000	-2.858945000	-0.330373000
C	1.958126000	-2.068508000	-0.194895000
H	-0.228781000	2.105600000	1.946749000
H	-0.029937000	4.545301000	1.616139000
H	1.277478000	5.445085000	-0.297628000
H	2.389286000	3.870338000	-1.869375000
H	2.195464000	1.436895000	-1.528983000
H	3.436803000	0.820297000	0.800325000
H	5.445531000	-0.588235000	0.576570000
H	5.240059000	-2.954214000	-0.160229000
H	2.988808000	-3.891367000	-0.653187000
H	0.980649000	-2.493363000	-0.407399000
O	-2.886303000	1.093064000	-0.768011000
C	-3.851627000	2.108291000	-0.473542000
H	-3.616590000	2.932985000	-1.144551000
H	-4.862173000	1.736296000	-0.656215000
H	-3.760496000	2.421690000	0.568914000
C	-2.919500000	-3.013818000	0.833833000

H	-2.677383000	-2.560584000	1.799789000
H	-3.965100000	-2.766131000	0.626471000
H	-2.787871000	-4.096101000	0.877778000

$TS_{1d+2a\rightarrow 6d}$

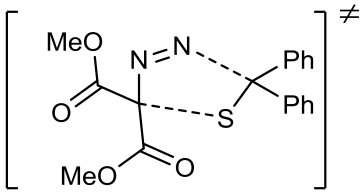

Imaginary Freq.: -388.30 cm^{-1}
Sum of electronic and zero-point Energies:-1502.345369
Sum of electronic and thermal Energies: -1502.321979
Sum of electronic and thermal Enthalpies: -1502.321035
Sum of electronic and thermal Free Energies: -1502.400124

Standard orientation. Coordinates (Angstroms):

N	1.003664000	0.024743000	1.422197000
N	-0.134006000	-0.041899000	1.592743000
C	-0.989916000	-0.164970000	-0.412492000
S	0.446788000	-0.225106000	-1.315728000
C	1.933202000	0.049428000	0.439787000
C	2.729650000	-1.218022000	0.389225000
C	2.509688000	1.403485000	0.143735000
O	3.415491000	1.597138000	-0.625677000
O	2.501817000	-2.157398000	1.119230000
C	-1.736529000	1.123233000	-0.287155000
C	-1.534970000	2.182271000	-1.181203000
C	-2.222730000	3.381626000	-1.032895000
C	-3.123614000	3.551829000	0.015826000
C	-3.333006000	2.508221000	0.914256000
C	-2.650309000	1.306587000	0.763411000
C	-1.730770000	-1.444870000	-0.195929000
C	-3.103292000	-1.526879000	-0.465137000
C	-3.786807000	-2.730967000	-0.318964000
C	-3.116366000	-3.871670000	0.110906000
C	-1.751049000	-3.800888000	0.384174000
C	-1.064478000	-2.604018000	0.226496000
H	-0.836505000	2.053646000	-2.002503000
H	-2.058150000	4.185030000	-1.746138000
H	-3.660626000	4.489532000	0.130525000
H	-4.028990000	2.629365000	1.740024000
H	-2.814346000	0.501539000	1.473558000
H	-3.636672000	-0.645864000	-0.808549000
H	-4.848858000	-2.773764000	-0.545578000
H	-3.651511000	-4.809548000	0.232146000
H	-1.215898000	-4.683551000	0.723902000
H	0.001476000	-2.565948000	0.435327000
O	1.841781000	2.344824000	0.810574000
C	2.230274000	3.689854000	0.515954000
H	1.602399000	4.316756000	1.147296000
H	3.286712000	3.840482000	0.749091000
H	2.054765000	3.908800000	-0.539858000
O	3.634407000	-1.187526000	-0.573823000
C	4.368214000	-2.404200000	-0.753527000
H	5.058370000	-2.204505000	-1.571608000
H	4.912286000	-2.657376000	0.159079000
H	3.688213000	-3.219409000	-1.010883000

TS$_{1e+2a\rightarrow 6e}$

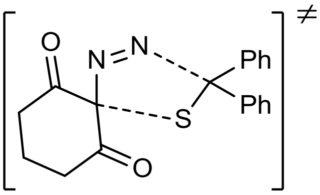

Imaginary Freq.: -393.08 cm^{-1}
Sum of electronic and zero-point Energies:-1352.053781
Sum of electronic and thermal Energies: -1352.032445
Sum of electronic and thermal Enthalpies: -1352.031501
Sum of electronic and thermal Free Energies: -1352.104929

Standard orientation. Coordinates (Angstroms):

N	1.05661800	-0.02051700	1.52114500
N	-0.09340000	-0.03042000	1.62511700
C	-0.87549000	-0.01319200	-0.34996900
S	0.57392100	-0.08702700	-1.24299500
C	2.01557600	-0.01895800	0.56838300
C	2.74749100	-1.31914100	0.40136300
C	3.82607500	-1.27954900	-0.65207700
C	2.71881300	1.29670200	0.37405400
O	2.43053700	2.27203300	1.03071900
O	2.45539500	-2.29994100	1.05295400
C	-1.58302000	1.29417000	-0.19105000
C	-1.32209200	2.38532000	-1.02796800
C	-1.98074800	3.59623700	-0.84575200
C	-2.91082000	3.74419700	0.18013600
C	-3.17888200	2.66766600	1.02233800
C	-2.52503100	1.45504200	0.83788700
C	-1.67683100	-1.27262900	-0.24194400
C	-3.03537500	-1.28007600	-0.58331800
C	-3.77295300	-2.45999800	-0.54043200
C	-3.17170300	-3.65023000	-0.14344400
C	-1.82124700	-3.65316300	0.20204700
C	-1.08010500	-2.47958000	0.14775100
H	3.33125100	-1.35415500	-1.63153200
H	4.44223100	-2.17378500	-0.52691800
H	-0.59867300	2.27582100	-1.83000400
H	-1.76729400	4.42743200	-1.51240600
H	-3.42344500	4.69164800	0.32234300
H	-3.89746700	2.77176400	1.83079800
H	-2.73464300	0.62348100	1.50407500
H	-3.51433000	-0.35902800	-0.90077400
H	-4.82263300	-2.44477800	-0.82155100
H	-3.74942600	-4.56961600	-0.10307800
H	-1.34021700	-4.57508200	0.51741400
H	-0.02649000	-2.49987300	0.41393200
C	3.78025000	1.25896400	-0.69716000
C	4.65303100	0.00631700	-0.58494100
H	4.36702100	2.17734900	-0.61320900
H	3.27203000	1.27424200	-1.67203700
H	5.39049600	0.00475800	-1.39335300
H	5.21722100	0.03350100	0.35557800

TS $_{\mathit{1f+2a\rightarrow 6f}}$

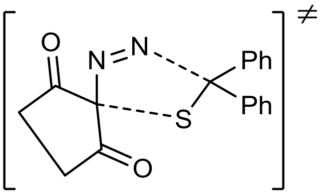

Imaginary Freq.: -423.14 cm^{-1}
Sum of electronic and zero-point Energies= -1350.874420
Sum of electronic and thermal Energies= -1350.855007
Sum of electronic and thermal Enthalpies= -1350.854062
Sum of electronic and thermal Free Energies= -1350.923860

Standard orientation. Coordinates (Angstroms):

N	1.244974000	0.049587000	1.504818000
N	0.089782000	0.005519000	1.607834000
C	-0.688240000	-0.013704000	-0.326784000
S	0.744639000	-0.022031000	-1.257728000
C	2.186655000	0.060014000	0.559373000
C	3.024735000	-1.150186000	0.290457000
C	4.126506000	-0.701059000	-0.657755000
C	2.996376000	1.283298000	0.253988000
O	2.827438000	2.390741000	0.696983000
O	2.852218000	-2.258458000	0.736448000
C	-1.455473000	1.261797000	-0.167466000
C	-1.259923000	2.355890000	-1.017731000
C	-1.972653000	3.535908000	-0.835045000
C	-2.892607000	3.648910000	0.204175000
C	-3.095875000	2.568719000	1.059717000
C	-2.387404000	1.387089000	0.875425000
C	-1.432230000	-1.309735000	-0.217611000
C	-2.798649000	-1.374396000	-0.517576000
C	-3.480566000	-2.587225000	-0.468055000
C	-2.814382000	-3.753609000	-0.105777000
C	-1.454706000	-3.699813000	0.196570000
C	-0.769154000	-2.493278000	0.135259000
H	3.960331000	-1.175625000	-1.630602000
H	5.078028000	-1.082629000	-0.274467000
H	-0.544112000	2.274315000	-1.829757000
H	-1.809132000	4.370095000	-1.511937000
H	-3.447815000	4.572055000	0.346348000
H	-3.806265000	2.645723000	1.878381000
H	-2.546921000	0.552890000	1.552031000
H	-3.328632000	-0.472711000	-0.808003000
H	-4.538057000	-2.616286000	-0.716859000
H	-3.348608000	-4.698693000	-0.060349000
H	-0.922334000	-4.603112000	0.481810000
H	0.293710000	-2.471379000	0.362178000
C	4.074689000	0.831678000	-0.721169000
H	5.016889000	1.312288000	-0.443572000
H	3.805577000	1.199759000	-1.717547000

4. Thiodiazolines 6 obtained from diazo compounds 1 and thiobenzophenone (2a)

Benzene (PCM), 6-31G(d), PBE1PBE

Thiadiazoline 6a

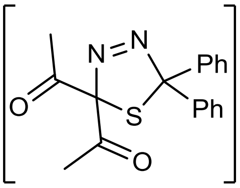

Sum of electronic and zero-point Energies:-1352.088113
Sum of electronic and thermal Energies: -1352.067114
Sum of electronic and thermal Enthalpies: -1352.066169
Sum of electronic and thermal Free Energies: -1352.140015

Standard orientation. Coordinates (Angstroms):

N	-1.341503000	-0.205547000	1.456774000
N	-0.128651000	-0.057076000	1.409516000
C	0.522766000	0.057183000	0.075642000
S	-0.854051000	-0.040250000	-1.164963000
C	-2.081504000	-0.230945000	0.192417000
C	-3.012788000	1.005870000	0.170725000
C	-3.996399000	1.144911000	-0.952652000
C	-2.102132000	-2.769871000	0.699493000
C	-2.772720000	-1.605476000	0.029163000
O	-3.777991000	-1.713907000	-0.635649000
O	-2.883513000	1.843990000	1.035425000
C	1.501918000	-1.112331000	0.010723000
C	1.532199000	-2.029540000	-1.039644000
C	2.461106000	-3.069393000	-1.040299000
C	3.360368000	-3.209058000	0.010985000
C	3.333403000	-2.297022000	1.064687000
C	2.416241000	-1.252983000	1.063484000
C	1.206905000	1.414981000	-0.013417000
C	2.415896000	1.564096000	-0.694248000
C	3.005645000	2.820258000	-0.810602000
C	2.400734000	3.933679000	-0.235548000
C	1.195756000	3.787617000	0.449036000
C	0.596562000	2.537726000	0.555020000
H	-3.552080000	0.875911000	-1.915424000
H	-4.832424000	0.457890000	-0.791391000
H	-4.355908000	2.175434000	-0.973914000
H	-2.148116000	-2.644464000	1.787363000
H	-2.611360000	-3.690391000	0.410331000
H	0.829686000	-1.937270000	-1.862025000
H	2.474906000	-3.771994000	-1.868910000
H	4.080126000	-4.022929000	0.010942000
H	4.030665000	-2.396819000	1.891920000
H	2.400379000	-0.540849000	1.882706000
H	2.899283000	0.697476000	-1.135295000
H	3.943798000	2.924408000	-1.348752000
H	2.865032000	4.912374000	-0.320353000
H	0.716025000	4.651334000	0.900967000
H	-0.352952000	2.438675000	1.074672000
H	-1.042874000	-2.825294000	0.424546000

Thiadiazoline 6b

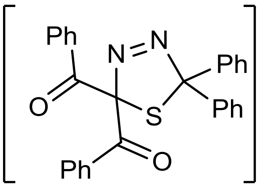

Sum of electronic and zero-point Energies:-1735.004018
Sum of electronic and thermal Energies: -1734.977186
Sum of electronic and thermal Enthalpies: -1734.976242
Sum of electronic and thermal Free Energies: -1735.064214

Standard orientation. Coordinates (Angstroms):

N	0.036069000	-0.068566000	-1.887727000
N	1.187476000	-0.367226000	-1.604125000
C	1.546956000	-0.596051000	-0.184364000
S	-0.011724000	-0.253609000	0.775975000
C	-0.935360000	0.026067000	-0.770739000
C	-1.942519000	-1.146939000	-0.937187000
C	-1.700714000	1.359455000	-0.994036000
O	-2.739771000	1.265563000	-1.618698000
O	-1.728324000	-1.994739000	-1.778531000
C	2.682412000	0.372052000	0.135490000
C	2.734802000	1.103995000	1.322665000
C	3.814856000	1.944303000	1.586254000
C	4.848875000	2.068779000	0.663510000
C	4.802619000	1.339624000	-0.522712000
C	3.732887000	0.490164000	-0.783127000
C	1.969594000	-2.053227000	-0.023067000
C	2.968606000	-2.408224000	0.885202000
C	3.318870000	-3.744481000	1.058928000
C	2.686984000	-4.736644000	0.315532000
C	1.694703000	-4.385664000	-0.597082000
C	1.331846000	-3.053778000	-0.763664000
H	1.926199000	1.029046000	2.043748000
H	3.841417000	2.503618000	2.517418000
H	5.687602000	2.728625000	0.867580000
H	5.604686000	1.428657000	-1.250171000
H	3.704027000	-0.082737000	-1.704364000
H	3.476952000	-1.640035000	1.460122000
H	4.093797000	-4.006559000	1.774116000
H	2.966296000	-5.778636000	0.445481000
H	1.195879000	-5.152213000	-1.183616000
H	0.545163000	-2.797685000	-1.467613000
C	-3.094421000	-1.250003000	0.002603000
C	-3.332410000	-0.353453000	1.050343000
C	-3.967302000	-2.328071000	-0.184348000
C	-4.420815000	-0.534738000	1.895286000
H	-2.674739000	0.493992000	1.225550000
C	-5.058122000	-2.504885000	0.655905000
H	-3.768341000	-3.017060000	-0.998944000
C	-5.286418000	-1.608359000	1.698466000
H	-4.594138000	0.164918000	2.707719000
H	-5.731158000	-3.343120000	0.499553000
H	-6.138834000	-1.746146000	2.358087000
C	-1.211392000	2.668209000	-0.503546000
C	0.138873000	2.956947000	-0.279656000
C	-2.171294000	3.674751000	-0.325166000
C	0.520263000	4.231305000	0.124552000
H	0.901738000	2.204503000	-0.438181000
C	-1.789124000	4.939690000	0.098089000
H	-3.213457000	3.442310000	-0.521797000
C	-0.441271000	5.219219000	0.323626000
H	1.572193000	4.447920000	0.286411000
H	-2.539251000	5.711063000	0.247186000
H	-0.140398000	6.210978000	0.650373000

Thiadiazoline 6c

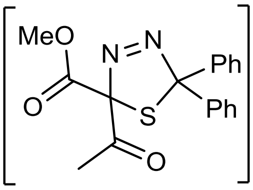

Sum of electronic and zero-point Energies:-1427.237189
Sum of electronic and thermal Energies: -1427.215203
Sum of electronic and thermal Enthalpies: -1427.214259
Sum of electronic and thermal Free Energies: -1427.290572

Standard orientation. Coordinates (Angstroms):

N	1.185129000	-0.389904000	1.309721000
N	0.005869000	-0.061456000	1.340135000
C	-0.719025000	0.084578000	0.052048000
S	0.569511000	-0.071300000	-1.266061000
C	1.794360000	-0.576982000	-0.025481000
C	2.245489000	-2.068626000	-0.131294000
C	3.226045000	-2.526465000	0.909509000
C	3.930190000	2.413052000	0.359791000
C	3.018272000	0.331775000	-0.162443000
O	3.979076000	0.042144000	-0.835425000
O	1.804684000	-2.780559000	-0.997616000
C	-1.342437000	1.468188000	-0.030626000
C	-2.456923000	1.687002000	-0.842913000
C	-2.992032000	2.965331000	-0.972341000
C	-2.428265000	4.033759000	-0.280448000
C	-1.318654000	3.818674000	0.533708000
C	-0.772205000	2.544995000	0.652630000
C	-1.752877000	-1.042951000	0.080917000
C	-2.809087000	-0.934592000	0.994859000
C	-3.748592000	-1.951775000	1.109767000
C	-3.646803000	-3.090659000	0.312614000
C	-2.594113000	-3.206660000	-0.588848000
C	-1.644737000	-2.192207000	-0.700962000
H	2.914344000	-2.196247000	1.905550000
H	3.311326000	-3.613708000	0.873499000
H	4.204829000	-2.080130000	0.700938000
H	3.656882000	3.258246000	0.989679000
H	4.873862000	1.973358000	0.689998000
H	4.017616000	2.722309000	-0.684191000
H	-2.909418000	0.853941000	-1.373519000
H	-3.855954000	3.123183000	-1.612089000
H	-2.851558000	5.030011000	-0.374698000
H	-0.873367000	4.646779000	1.078452000
H	0.103018000	2.388166000	1.276483000
H	-2.890778000	-0.048106000	1.617053000
H	-4.562294000	-1.853955000	1.823146000
H	-4.383911000	-3.884549000	0.397624000
H	-2.502176000	-4.092972000	-1.210587000
H	-0.814073000	-2.299739000	-1.391312000
O	2.863848000	1.467804000	0.505420000

Thiadiazoline 6d

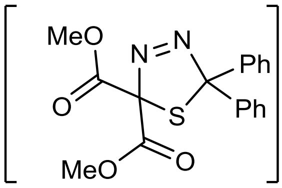

Sum of electronic and zero-point Energies:-1502.384019
Sum of electronic and thermal Energies: -1502.361034
Sum of electronic and thermal Enthalpies: -1502.360090
Sum of electronic and thermal Free Energies: -1502.439379

Standard orientation. Coordinates (Angstroms):

N	0.992591000	0.016611000	1.518073000
N	-0.222908000	-0.081015000	1.439553000
C	-0.852875000	-0.160080000	0.095947000
S	0.553956000	-0.253943000	-1.106524000
C	1.745316000	0.037149000	0.238876000
C	2.753375000	-1.112878000	0.261961000
C	2.465193000	1.395870000	0.213781000
O	3.647254000	1.525673000	0.411627000
O	2.812643000	-1.941865000	1.134635000
C	-1.713208000	1.096507000	-0.011490000
C	-1.581968000	2.027128000	-1.040143000
C	-2.406669000	3.149951000	-1.081484000
C	-3.359132000	3.360410000	-0.089843000
C	-3.488680000	2.437400000	0.946223000
C	-2.675901000	1.310659000	0.983393000
C	-1.660199000	-1.449302000	0.018862000
C	-2.868182000	-1.501682000	-0.677052000
C	-3.569021000	-2.701352000	-0.777976000
C	-3.076889000	-3.853184000	-0.172562000
C	-1.871454000	-3.804653000	0.525591000
C	-1.162225000	-2.612853000	0.614401000
H	-0.829527000	1.878160000	-1.807434000
H	-2.298442000	3.862347000	-1.894950000
H	-3.998172000	4.238645000	-0.122653000
H	-4.226953000	2.592712000	1.728119000
H	-2.783130000	0.589470000	1.788099000
H	-3.264664000	-0.603687000	-1.141189000
H	-4.505675000	-2.730493000	-1.327919000
H	-3.628489000	-4.786562000	-0.244354000
H	-1.478816000	-4.699231000	1.000920000
H	-0.212811000	-2.589750000	1.143338000
C	4.442500000	-2.160635000	-0.955531000
H	4.954255000	-1.993328000	-1.902163000
H	5.149659000	-2.125748000	-0.123924000
H	3.929953000	-3.125418000	-0.959006000
C	2.156351000	3.701455000	0.057287000
H	1.313352000	4.373938000	-0.094496000
H	2.627331000	3.882889000	1.026034000
H	2.897369000	3.824492000	-0.736055000
O	1.602375000	2.382495000	0.015203000
O	3.493442000	-1.095551000	-0.840568000

Thiadiazoline 6e

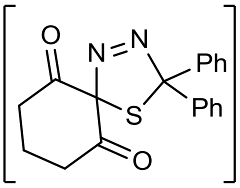

Sum of electronic and zero-point Energies:-1390.149022
Sum of electronic and thermal Energies: -1390.129108
Sum of electronic and thermal Enthalpies: -1390.128164
Sum of electronic and thermal Free Energies: -1390.199295

Standard orientation. Coordinates (Angstroms):

N	1.066821000	-0.030233000	1.574427000
N	-0.148992000	0.018639000	1.468549000
C	-0.749406000	0.030592000	0.107896000
S	0.685044000	0.066686000	-1.074933000
C	-1.560337000	1.311021000	-0.048695000
C	-2.775964000	1.314098000	-0.733448000
C	-3.483738000	2.502055000	-0.902119000
C	-2.990255000	3.691903000	-0.376887000
C	-1.777331000	3.692236000	0.310160000
C	-1.061647000	2.511517000	0.468241000
C	-1.596891000	-1.235238000	0.030115000
C	-2.538716000	-1.446693000	1.045732000
C	-3.345244000	-2.578168000	1.033468000
C	-3.232193000	-3.508915000	0.002490000
C	-2.303415000	-3.300079000	-1.011023000
C	-1.485259000	-2.171867000	-0.995733000
H	-3.173599000	0.387104000	-1.135198000
H	-4.426597000	2.492202000	-1.442111000
H	-3.546731000	4.616734000	-0.502265000
H	-1.383692000	4.616910000	0.723058000
H	-0.106698000	2.525720000	0.987498000
H	-2.631573000	-0.721228000	1.847961000
H	-4.064680000	-2.732202000	1.832955000
H	-3.864614000	-4.392379000	-0.008401000
H	-2.206042000	-4.019023000	-1.819863000
H	-0.754771000	-2.026722000	-1.785051000
C	1.854211000	-0.049798000	0.343335000
C	2.760903000	1.195535000	0.308254000
C	2.617269000	-1.385102000	0.246772000
C	3.906377000	1.131383000	-0.668704000
C	3.748940000	-1.399891000	-0.750148000
C	4.670171000	-0.191267000	-0.560757000
H	4.550797000	1.996194000	-0.490597000
H	3.487198000	1.233104000	-1.681059000
H	4.284854000	-2.345878000	-0.637707000
H	3.315389000	-1.377921000	-1.760646000
H	5.462033000	-0.215696000	-1.315592000
H	5.163787000	-0.253892000	0.416881000
O	2.523654000	2.146185000	1.016328000
O	2.296918000	-2.327282000	0.930488000

Thiadiazoline 6f

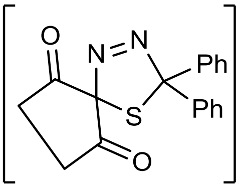

Sum of electronic and zero-point Energies= -1350.910191
Sum of electronic and thermal Energies= -1350.891076
Sum of electronic and thermal Enthalpies= -1350.890131
Sum of electronic and thermal Free Energies= -1350.961102

Standard orientation. Coordinates (Angstroms):

N	1.412802000	0.141231000	1.267210000
N	0.189926000	0.086959000	1.325416000
C	-0.581648000	0.026709000	0.065251000
S	0.664449000	0.314178000	-1.284448000
C	-1.604186000	1.149555000	0.020124000
C	-2.682621000	1.066512000	-0.864359000
C	-3.587853000	2.117415000	-0.969689000
C	-3.433633000	3.256141000	-0.182661000
C	-2.363530000	3.340195000	0.703866000
C	-1.448451000	2.296187000	0.800934000
C	-1.219223000	-1.365169000	0.075923000
C	-2.321114000	-1.578394000	0.913567000
C	-2.899020000	-2.838571000	1.010939000
C	-2.383497000	-3.902767000	0.273624000
C	-1.280141000	-3.698527000	-0.548555000
C	-0.693470000	-2.438182000	-0.642655000
H	-2.814718000	0.174871000	-1.471270000
H	-4.418603000	2.042452000	-1.665973000
H	-4.144565000	4.074140000	-0.259871000
H	-2.235155000	4.224091000	1.322540000
H	-0.610574000	2.376197000	1.486931000
H	-2.726528000	-0.751319000	1.488923000
H	-3.754823000	-2.988322000	1.663451000
H	-2.838271000	-4.887180000	0.344297000
H	-0.865186000	-4.523623000	-1.120942000
H	0.182011000	-2.292383000	-1.267081000
C	1.998806000	0.217518000	-0.103828000
C	2.962344000	1.432223000	-0.032716000
C	2.981327000	-0.975812000	-0.196736000
C	4.314290000	0.956048000	0.465596000
C	4.269226000	-0.575964000	0.491836000
H	4.495719000	1.396413000	1.451829000
H	5.084769000	1.350526000	-0.205108000
H	4.214303000	-0.957317000	1.519240000
H	5.118980000	-1.061705000	0.006088000
O	2.665746000	2.556999000	-0.329715000
O	2.746344000	-2.020964000	-0.741911000

5. Transition states for decompositions of thiadiazolines 6

Benzene (PCM), 6-31G(d), PBE1PBE

$TS_{6a \rightarrow N \equiv N + 7a}$

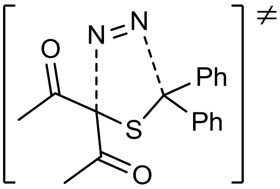

Imaginary Freq.: -378.05 cm^{-1}
Sum of electronic and zero-point Energies:-1352.055128
Sum of electronic and thermal Energies: -1352.033819
Sum of electronic and thermal Enthalpies: -1352.032875
Sum of electronic and thermal Free Energies: -1352.106186

Standard orientation. Coordinates (Angstroms):

S	-0.424438000	-1.111336000	-0.975564000
C	-1.921531000	-0.788616000	-0.148976000
C	-2.813039000	-1.992220000	-0.155132000
O	-2.493341000	-3.018745000	-0.728832000
C	-4.094879000	-1.894390000	0.632191000
H	-3.875479000	-1.697475000	1.688234000
H	-4.728747000	-1.079031000	0.265423000
H	-4.634326000	-2.838894000	0.543667000
C	-2.625892000	0.549146000	-0.309041000
O	-2.800912000	0.992486000	-1.426112000
C	-3.088356000	1.292792000	0.915482000
H	-3.850636000	2.020080000	0.628735000
H	-3.457304000	0.635176000	1.705254000
H	-2.225426000	1.833715000	1.325732000
C	0.707497000	-0.129645000	-0.062139000
N	-1.189191000	-0.710714000	1.724734000
N	-0.063383000	-0.471205000	1.714276000
C	2.068230000	-0.724667000	-0.062614000
C	2.241174000	-2.070036000	0.299734000
C	3.195863000	0.030216000	-0.415877000
C	3.505799000	-2.642719000	0.306147000
H	1.375438000	-2.657862000	0.591103000
C	4.459926000	-0.548459000	-0.414802000
H	3.076902000	1.068933000	-0.707299000
C	4.618812000	-1.884148000	-0.053539000
H	3.624448000	-3.682402000	0.597555000
H	5.323085000	0.045640000	-0.701496000
H	5.608170000	-2.333285000	-0.048991000
C	0.620189000	1.357961000	-0.040623000
C	1.156178000	2.061093000	1.050917000
C	0.067992000	2.079794000	-1.103259000
C	1.113902000	3.448949000	1.084771000
H	1.596781000	1.509312000	1.876088000
C	0.033342000	3.471149000	-1.068101000
H	-0.337769000	1.548856000	-1.958247000
C	0.550716000	4.159458000	0.024839000
H	1.523431000	3.978099000	1.940772000
H	-0.400900000	4.014857000	-1.902232000
H	0.519848000	5.245196000	0.051352000

$\underline{TS_{6b\rightarrow N\equiv N+7b}}$

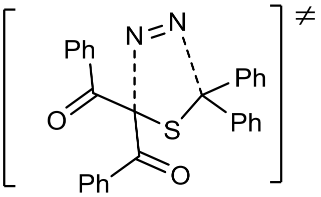

Imaginary Freq.: 384.55 cm^{-1}
Sum of electronic and zero-point Energies: -1734.974896
Sum of electronic and thermal Energies: -1734.947548
Sum of electronic and thermal Enthalpies: -1734.946604
Sum of electronic and thermal Free Energies: -1735.035020

Standard orientation. Coordinates (Angstroms):

N	0.59891000	0.30225900	2.17270400
N	0.27949400	1.36326100	1.86124700
C	-0.31471000	1.59214100	0.06304400
S	0.59076000	0.24973300	-0.64455300
C	0.44641500	-0.96869300	0.58176900
C	-0.90134100	-1.59871800	0.93207900
C	1.54688700	-1.96316600	0.68804700
O	1.27523400	-3.09097000	1.08779100
O	-1.40293400	-1.40337100	2.02045900
C	0.19096000	2.90399900	-0.42258300
C	1.56499900	3.18689300	-0.37043300
C	2.05342700	4.40914200	-0.81165300
C	1.17848900	5.37030300	-1.31566700
C	-0.18684300	5.10206000	-1.37275500
C	-0.67916800	3.88050500	-0.92755200
C	-1.78863100	1.47749000	0.26906600
C	-2.58565200	0.69755000	-0.57391300
C	-3.96500600	0.64326900	-0.38971900
C	-4.56441500	1.36822700	0.63429500
C	-3.77780100	2.15794300	1.47252400
C	-2.40305200	2.21962400	1.28893600
H	2.24896300	2.44324300	0.02823300
H	3.11854700	4.61532000	-0.75754400
H	1.56102000	6.32689500	-1.66078200
H	-0.87276300	5.84535200	-1.76908900
H	-1.74322200	3.67481600	-0.98140300
H	-2.12586300	0.14018200	-1.38430100
H	-4.56720000	0.02728000	-1.05147400
H	-5.64005900	1.32202800	0.77992700
H	-4.23731200	2.72670600	2.27599300
H	-1.79321300	2.83257600	1.94578600
C	-1.58010000	-2.46617000	-0.07878900
C	-0.99331300	-2.85129500	-1.28838700
C	-2.86909900	-2.91770800	0.22986000
C	-1.68677000	-3.66799800	-2.17570900
H	0.00977100	-2.52320700	-1.54116200
C	-3.56182100	-3.73024700	-0.65698000
H	-3.30816200	-2.61268400	1.17443200
C	-2.97140200	-4.10632100	-1.86345200
H	-1.22106900	-3.96577800	-3.11093800
H	-4.56240800	-4.07395300	-0.40927900
H	-3.51166800	-4.74365800	-2.55843100
C	2.95822800	-1.61383300	0.35399800
C	3.53363200	-0.37289300	0.64768200
C	3.75305100	-2.62326900	-0.20204800
C	4.87815400	-0.14302000	0.37377300

H	2.94160500	0.39848800	1.12844000
C	5.08852800	-2.38332400	-0.49862800
H	3.30509600	-3.59332500	-0.39603700
C	5.65335200	-1.14143100	-0.21115900
H	5.32254400	0.81672700	0.62249600
H	5.69340800	-3.16731000	-0.94568000
H	6.70061300	-0.95569900	-0.43385500

$TS_{6c\rightarrow N\equiv N+7c}$

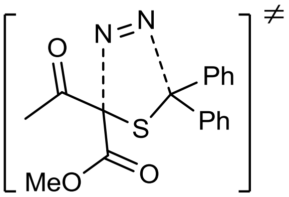

Imaginary Freq.: -379.08 cm^{-1}
Sum of electronic and zero-point Energies:-1427.206306
Sum of electronic and thermal Energies: -1427.183925
Sum of electronic and thermal Enthalpies: -1427.182980
Sum of electronic and thermal Free Energies: -1427.259303

Standard orientation. Coordinates (Angstroms):

S	-0.405572000	-0.872435000	-1.003507000
C	-1.811068000	-0.247946000	-0.200905000
C	-2.913972000	-1.246629000	-0.200505000
O	-2.812323000	-2.412453000	-0.516155000
C	-5.167448000	-1.587775000	0.327035000
H	-5.999701000	-0.984110000	0.687978000
H	-5.397170000	-2.012022000	-0.653268000
H	-4.949642000	-2.398311000	1.026886000
C	-2.243568000	1.199795000	-0.353108000
O	-2.186576000	1.732412000	-1.442893000
C	-2.716615000	1.933485000	0.871272000
H	-3.304751000	2.801432000	0.566186000
H	-3.288980000	1.290150000	1.541154000
H	-1.833370000	2.284664000	1.420642000
C	0.905631000	-0.187743000	-0.049665000
N	-1.082968000	-0.337019000	1.699732000
N	0.067030000	-0.362665000	1.687604000
C	2.092970000	-1.082333000	-0.061203000
C	1.946495000	-2.434594000	0.286225000
C	3.364593000	-0.606843000	-0.410935000
C	3.042951000	-3.286148000	0.284023000
H	0.967073000	-2.807195000	0.572542000
C	4.458744000	-1.464482000	-0.420040000
H	3.490429000	0.434134000	-0.691012000
C	4.301727000	-2.803846000	-0.071721000
H	2.916393000	-4.327832000	0.564623000
H	5.436440000	-1.085403000	-0.703824000
H	5.159158000	-3.471248000	-0.074549000
C	1.171680000	1.279242000	-0.008270000
C	1.860640000	1.816321000	1.091532000
C	0.809206000	2.126956000	-1.058651000
C	2.155170000	3.172399000	1.144478000
H	2.154407000	1.163288000	1.908154000
C	1.112196000	3.484708000	-1.004122000
H	0.283034000	1.722915000	-1.917053000
C	1.781265000	4.012270000	0.095735000
H	2.679638000	3.574807000	2.006698000
H	0.820901000	4.128928000	-1.828790000

H	2.013785000	5.072823000	0.136870000
O	-4.057415000	-0.693868000	0.235858000

__$TS_{6d \rightarrow N \equiv N+7d}$__

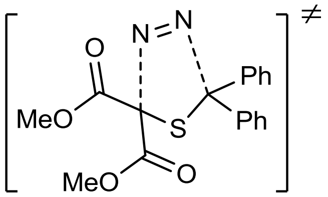

Imaginary Freq.: -379.83 cm^{-1}
Sum of electronic and zero-point Energies:-1502.355163
Sum of electronic and thermal Energies: -1502.331824
Sum of electronic and thermal Enthalpies: -1502.330880
Sum of electronic and thermal Free Energies: -1502.409774

Standard orientation. Coordinates (Angstroms):

S	-0.110266000	-1.100338000	-0.985976000
C	-1.593242000	-0.662745000	-0.209646000
C	-2.577223000	-1.774737000	-0.218531000
O	-2.332109000	-2.929660000	-0.494152000
C	-4.789926000	-2.344793000	0.267928000
H	-5.689868000	-1.828092000	0.600191000
H	-4.953802000	-2.815116000	-0.704807000
H	-4.497926000	-3.109731000	0.991681000
C	-2.190079000	0.710627000	-0.392602000
O	-2.380618000	1.209713000	-1.479020000
C	-3.123827000	2.572518000	0.644601000
H	-2.447552000	3.271932000	0.146802000
H	-4.052796000	2.498532000	0.073891000
H	-3.325028000	2.893987000	1.666004000
C	1.070086000	-0.191694000	-0.049695000
N	-0.878142000	-0.626215000	1.696815000
N	0.259729000	-0.461806000	1.689818000
C	2.387836000	-0.878461000	-0.038776000
C	2.463338000	-2.227352000	0.342683000
C	3.565952000	-0.209528000	-0.399370000
C	3.684498000	-2.887669000	0.361429000
H	1.557347000	-2.747794000	0.640197000
C	4.785883000	-0.876041000	-0.386251000
H	3.520710000	0.830984000	-0.704763000
C	4.848785000	-2.214383000	-0.005478000
H	3.729057000	-3.928803000	0.667871000
H	5.689698000	-0.348836000	-0.678354000
H	5.803973000	-2.732078000	0.008650000
C	1.085497000	1.299353000	-0.054073000
C	1.662121000	1.979390000	1.030643000
C	0.594638000	2.040688000	-1.132768000
C	1.721964000	3.366455000	1.041429000
H	2.051765000	1.410849000	1.869930000
C	0.660750000	3.431980000	-1.119817000
H	0.160349000	1.527880000	-1.984712000
C	1.220261000	4.098796000	-0.034457000
H	2.162160000	3.878289000	1.892691000
H	0.275382000	3.991439000	-1.967600000
H	1.270723000	5.184145000	-0.026512000
O	-3.782852000	-1.337751000	0.172521000
O	-2.507537000	1.288892000	0.762467000

***TS*$_{6e→N≡N+7e}$**

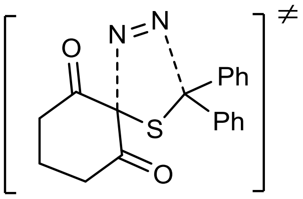

Imaginary Freq.: -382.73
Sum of electronic and zero-point Energies: -1390.125025
Sum of electronic and thermal Energies: -1390.104575
Sum of electronic and thermal Enthalpies: -1390.103631
Sum of electronic and thermal Free Energies: -1390.175581

Standard orientation. Coordinates (Angstroms):

N	-1.16943200	-0.40989800	1.63842600
N	-0.02940100	-0.33828300	1.55065500
C	0.79869900	-0.12766700	-0.00725900
S	-0.41496100	-0.82017200	-1.12468300
C	2.00133800	-1.02101500	0.00909300
C	3.28437400	-0.52473500	-0.25159100
C	4.38174800	-1.37939000	-0.24532900
C	4.21379600	-2.73232600	0.03651000
C	2.94098700	-3.23433700	0.30539300
C	1.84129500	-2.38773700	0.28431600
C	1.10524700	1.34115400	0.02926000
C	1.65866400	1.90167100	1.18750400
C	2.03455300	3.23968200	1.21116900
C	1.88694000	4.02656200	0.07111600
C	1.35679200	3.46787800	-1.08838700
C	0.96218900	2.13430900	-1.11120700
H	3.42016700	0.52824100	-0.47444300
H	5.37044900	-0.98533300	-0.46223400
H	5.07354200	-3.39665400	0.04770200
H	2.80505200	-4.28824900	0.52980800
H	0.84841400	-2.77963400	0.48703200
H	1.78971700	1.28668600	2.07353900
H	2.44900600	3.66495900	2.12091500
H	2.18596800	5.07103500	0.08667400
H	1.23947900	4.07357900	-1.98243400
H	0.53316100	1.70566100	-2.00926700
C	-1.94677100	-0.43043000	-0.40569900
C	-2.86970500	-1.59386900	-0.34547600
C	-2.49475100	0.94112000	-0.39991900
C	-4.26662800	-1.32562300	0.18125000
C	-3.85822500	1.13682400	0.24246000
C	-4.37028300	-0.06354100	1.02548900
H	-4.58455300	-2.22159900	0.72362200
H	-4.92297300	-1.24537100	-0.69883900
H	-3.79700700	2.03982600	0.85948800
H	-4.54879100	1.37702100	-0.57916200
H	-5.41023600	0.10236100	1.32662000
H	-3.78905600	-0.18640000	1.94601100
O	-2.53952900	-2.71627300	-0.69405400
O	-1.90955600	1.89161200	-0.89292400

TS $_{6f\rightarrow N\equiv N+7f}$

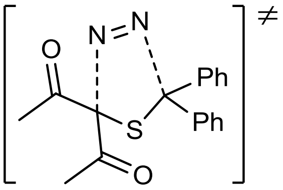

Imaginary Freq.: -386.68 cm^{-1}
Sum of electronic and zero-point Energies= -1350.891054
Sum of electronic and thermal Energies= -1350.871587
Sum of electronic and thermal Enthalpies= -1350.870643
Sum of electronic and thermal Free Energies= -1350.940541

Standard orientation. Coordinates (Angstroms):

N	-1.252219000	-0.492497000	1.685359000
N	-0.118112000	-0.376808000	1.561192000
C	0.645633000	-0.118728000	0.006071000
S	-0.557859000	-0.880509000	-1.090609000
C	1.904604000	-0.929540000	-0.023382000
C	3.130338000	-0.360488000	-0.387710000
C	4.279089000	-1.143614000	-0.435681000
C	4.219027000	-2.494828000	-0.106347000
C	3.002962000	-3.068459000	0.263549000
C	1.851409000	-2.294340000	0.296023000
C	0.839120000	1.368043000	0.031031000
C	1.488896000	1.946405000	1.131100000
C	1.737147000	3.312304000	1.161604000
C	1.359154000	4.114321000	0.085552000
C	0.726905000	3.542340000	-1.013506000
C	0.461169000	2.176487000	-1.041765000
H	3.180557000	0.691552000	-0.648024000
H	5.222827000	-0.695048000	-0.732160000
H	5.119008000	-3.102747000	-0.137572000
H	2.951886000	-4.121512000	0.524169000
H	0.901875000	-2.741791000	0.577033000
H	1.791569000	1.320641000	1.966452000
H	2.229553000	3.750287000	2.025180000
H	1.557787000	5.182316000	0.106025000
H	0.428106000	4.160422000	-1.855116000
H	-0.043861000	1.736301000	-1.893273000
C	-2.064294000	-0.545024000	-0.330370000
C	-3.006293000	-1.667627000	-0.143542000
C	-2.805578000	0.723316000	-0.252051000
C	-4.316029000	-1.096690000	0.384743000
C	-4.153796000	0.424535000	0.396323000
H	-4.510122000	-1.522666000	1.374820000
H	-5.126835000	-1.434403000	-0.269878000
H	-4.134521000	0.832913000	1.413756000
H	-4.938174000	0.959354000	-0.147103000
O	-2.789329000	-2.847295000	-0.342207000
O	-2.449219000	1.825692000	-0.621575000

6. **Thiodiazolines 6 decomposition products**

Benzene (PCM), 6-31G(d), PBE1PBE

Nitrogen (N≡N)

Sum of electronic and zero-point Energies: -109.394762
Sum of electronic and thermal Energies: -109.392401
Sum of electronic and thermal Enthalpies: -109.391457
Sum of electronic and thermal Free Energies: -109.413206

Standard orientation. Coordinates (Angstroms):

N	0.000000000	0.000000000	0.551250000
N	0.000000000	0.000000000	-0.551250000

C=S-ylide 7a

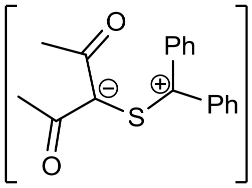

Sum of electronic and zero-point Energies:-1242.717020
Sum of electronic and thermal Energies: -1242.697482
Sum of electronic and thermal Enthalpies: -1242.696538
Sum of electronic and thermal Free Energies: -1242.766744

Standard orientation. Coordinates (Angstroms):

S	0.352646000	-1.464364000	-0.444416000
C	1.923637000	-0.959841000	0.105042000
C	2.926977000	-1.456000000	-0.805630000
O	2.578097000	-1.946672000	-1.890390000
C	4.406475000	-1.423482000	-0.483675000
H	4.936765000	-1.832288000	-1.345694000
H	4.764394000	-0.407556000	-0.292626000
H	4.642017000	-2.028944000	0.397478000
C	1.992738000	-0.319188000	1.402984000
O	0.981589000	0.016764000	2.027304000
C	3.342377000	-0.039353000	2.040167000
H	3.149839000	0.403397000	3.019064000
H	3.943521000	-0.943841000	2.168328000
H	3.925801000	0.668210000	1.441726000
C	-0.713646000	-0.195014000	-0.205263000
C	-2.119795000	-0.554452000	-0.065436000
C	-3.119388000	0.284782000	-0.596979000
C	-2.506711000	-1.755995000	0.557611000
C	-4.454810000	-0.081693000	-0.526138000
H	-2.833442000	1.207269000	-1.091981000
C	-3.845829000	-2.114065000	0.627099000
H	-1.751055000	-2.380038000	1.024222000
C	-4.822246000	-1.281180000	0.084392000
H	-5.213860000	0.567401000	-0.952933000
H	-4.130298000	-3.037353000	1.123143000
H	-5.870328000	-1.560545000	0.145613000
C	-0.309732000	1.209826000	-0.239259000
C	-0.876329000	2.129778000	0.655404000
C	0.615295000	1.661444000	-1.194101000
C	-0.523979000	3.472704000	0.596651000
H	-1.570540000	1.777671000	1.411748000
C	0.949128000	3.006250000	-1.259533000
H	1.048885000	0.954296000	-1.895396000
C	0.385070000	3.913858000	-0.361695000
H	-0.958682000	4.174973000	1.302031000
H	1.647922000	3.350732000	-2.016477000
H	0.653855000	4.965415000	-0.412760000

C=S-ylide 7b

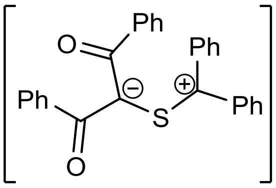

Sum of electronic and zero-point Energies: -1625.631866
Sum of electronic and thermal Energies: -1625.606155
Sum of electronic and thermal Enthalpies: -1625.605211
Sum of electronic and thermal Free Energies: -1625.692064

Standard orientation. Coordinates (Angstroms):

C	1.503769000	0.339118000	0.849971000
S	0.207062000	-0.564458000	1.411996000
C	-1.177548000	-0.194504000	0.436524000
C	-2.330086000	-0.128931000	1.305388000
C	-1.047386000	-0.107473000	-1.014368000
O	-1.731023000	0.640773000	-1.712570000
O	-2.135772000	-0.191795000	2.534952000
C	1.388752000	1.603631000	0.121197000
C	0.412145000	2.555227000	0.463844000
C	0.367743000	3.775949000	-0.190273000
C	1.276371000	4.061365000	-1.211131000
C	2.240284000	3.122513000	-1.567410000
C	2.303731000	1.903792000	-0.901416000
C	2.820069000	-0.187298000	1.200269000
C	3.862590000	0.695834000	1.546713000
C	5.093565000	0.203884000	1.951719000
C	5.316590000	-1.172822000	2.006242000
C	4.300981000	-2.057045000	1.650793000
C	3.062892000	-1.572683000	1.249291000
H	-0.295850000	2.336604000	1.257078000
H	-0.379892000	4.510052000	0.094880000
H	1.229669000	5.016191000	-1.727195000
H	2.945897000	3.337942000	-2.364551000
H	3.049773000	1.166663000	-1.182029000
H	3.683656000	1.766134000	1.525497000
H	5.882855000	0.895223000	2.232171000
H	6.285433000	-1.554020000	2.316464000
H	4.478377000	-3.128304000	1.670108000
H	2.289961000	-2.261081000	0.921980000
C	-3.753140000	-0.150475000	0.833597000
C	-4.248198000	0.560077000	-0.263828000
C	-4.646248000	-0.886059000	1.625900000
C	-5.607464000	0.516221000	-0.568113000
H	-3.567154000	1.140847000	-0.873566000
C	-5.995843000	-0.944620000	1.306634000
H	-4.258168000	-1.406403000	2.495891000
C	-6.482150000	-0.239764000	0.205771000
H	-5.982013000	1.080419000	-1.418378000
H	-6.672100000	-1.533509000	1.920935000
H	-7.540021000	-0.276102000	-0.041986000
C	-0.050668000	-0.991677000	-1.714698000
C	0.177578000	-2.315391000	-1.324656000
C	0.571286000	-0.504642000	-2.868067000
C	1.030506000	-3.130296000	-2.063972000
H	-0.341413000	-2.716217000	-0.457729000
C	1.438511000	-1.311703000	-3.595798000
H	0.361138000	0.513969000	-3.180015000

C	1.670744000	-2.626410000	-3.194260000
H	1.186611000	-4.163088000	-1.763796000
H	1.927666000	-0.919637000	-4.483623000
H	2.340686000	-3.261065000	-3.768316000

C=S-ylide 7c

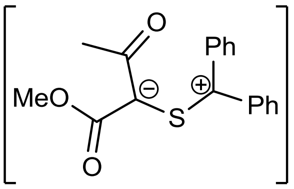

Sum of electronic and zero-point Energies:-1317.867614
Sum of electronic and thermal Energies: -1317.847062
Sum of electronic and thermal Enthalpies: -1317.846118
Sum of electronic and thermal Free Energies: -1317.918795

Standard orientation. Coordinates (Angstroms):

S	0.263714000	-1.366459000	-0.311383000
C	1.734270000	-0.669080000	0.262209000
C	2.854867000	-1.128102000	-0.528397000
O	2.738282000	-1.781199000	-1.560178000
C	5.170955000	-1.244670000	-0.814465000
H	6.060155000	-0.871776000	-0.304576000
H	5.189614000	-2.336947000	-0.859555000
H	5.124195000	-0.851860000	-1.833741000
O	4.067459000	-0.786136000	-0.041429000
C	1.698036000	0.070717000	1.508678000
O	0.635122000	0.347852000	2.072329000
C	2.998714000	0.506434000	2.153574000
H	3.649728000	-0.345237000	2.367647000
H	3.558992000	1.173257000	1.491543000
H	2.746707000	1.027795000	3.079110000
C	-0.945692000	-0.209277000	-0.178698000
C	-2.304925000	-0.723687000	-0.076864000
C	-3.368544000	-0.024234000	-0.682899000
C	-2.581777000	-1.941179000	0.573986000
C	-4.654136000	-0.542294000	-0.658949000
H	-3.167156000	0.909217000	-1.198484000
C	-3.872154000	-2.452332000	0.594032000
H	-1.784800000	-2.455757000	1.101462000
C	-4.910285000	-1.757807000	-0.023237000
H	-5.461175000	0.000147000	-1.142833000
H	-4.071802000	-3.386301000	1.111071000
H	-5.920692000	-2.156223000	0.000370000
C	-0.701483000	1.229614000	-0.258884000
C	-1.395998000	2.111747000	0.582046000
C	0.191220000	1.748440000	-1.209929000
C	-1.201656000	3.483331000	0.473275000
H	-2.064785000	1.710067000	1.336531000
C	0.368057000	3.119365000	-1.325643000
H	0.724453000	1.070733000	-1.869967000
C	-0.323142000	3.989555000	-0.481402000
H	-1.734937000	4.157341000	1.137375000
H	1.043856000	3.513031000	-2.079444000
H	-0.176645000	5.062480000	-0.570754000

C=S-ylide 7d

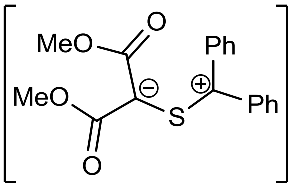

Sum of electronic and zero-point Energies:-1393.011609
Sum of electronic and thermal Energies: -1392.989973
Sum of electronic and thermal Enthalpies: -1392.989029
Sum of electronic and thermal Free Energies: -1393.064416

Standard orientation. Coordinates (Angstroms):

S	0.058242000	-1.448883000	-0.159528000
C	1.606508000	-0.763730000	0.121472000
C	2.614370000	-1.504122000	-0.609582000
O	2.333462000	-2.366023000	-1.438311000
C	4.862106000	-1.957432000	-1.010246000
H	5.826831000	-1.577058000	-0.672230000
H	4.774543000	-3.023699000	-0.783288000
H	4.755892000	-1.819436000	-2.089920000
O	3.887070000	-1.202973000	-0.300445000
C	1.746310000	0.258173000	1.142501000
O	0.833813000	0.670767000	1.845340000
O	3.002218000	0.733747000	1.257405000
C	3.170374000	1.712143000	2.274947000
H	4.222567000	1.996895000	2.234740000
H	2.532786000	2.581334000	2.089825000
H	2.926989000	1.302554000	3.259534000
C	-1.111257000	-0.249298000	-0.183102000
C	-2.475540000	-0.714396000	0.041975000
C	-3.544403000	-0.101678000	-0.643317000
C	-2.754796000	-1.801699000	0.891328000
C	-4.837390000	-0.581141000	-0.501644000
H	-3.342384000	0.730672000	-1.309864000
C	-4.052807000	-2.276033000	1.026871000
H	-1.953540000	-2.240908000	1.477463000
C	-5.096434000	-1.670667000	0.330906000
H	-5.648418000	-0.107569000	-1.047329000
H	-4.252574000	-3.108558000	1.695101000
H	-6.112287000	-2.038348000	0.444960000
C	-0.847052000	1.162584000	-0.455267000
C	-1.515837000	2.154233000	0.277567000
C	0.034320000	1.545472000	-1.478922000
C	-1.304378000	3.498842000	-0.003833000
H	-2.182485000	1.862020000	1.082770000
C	0.227421000	2.887957000	-1.767499000
H	0.551280000	0.784280000	-2.055316000
C	-0.435853000	3.868283000	-1.027138000
H	-1.818274000	4.258280000	0.578351000
H	0.895708000	3.173451000	-2.574739000
H	-0.274970000	4.919124000	-1.251623000

C=S-ylide 7e

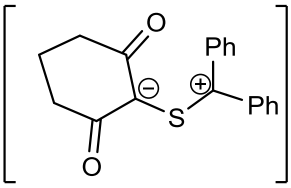

Sum of electronic and zero-point Energies: -1280.791710
Sum of electronic and thermal Energies: -1280.773091
Sum of electronic and thermal Enthalpies: -1280.772147
Sum of electronic and thermal Free Energies: -1280.840121

Standard orientation. Coordinates (Angstroms):

C	-0.91413200	-0.19433400	0.24342500
S	0.19497000	-1.40781800	0.58946800
C	-2.29113200	-0.62513800	0.04181100
C	-3.35468800	0.19126100	0.47777800
C	-4.66659400	-0.24023300	0.35677700
C	-4.94742700	-1.48364600	-0.21043100
C	-3.90734400	-2.29497400	-0.65968000
C	-2.59105800	-1.87197100	-0.53958800
C	-0.57399300	1.22675200	0.23866800
C	-1.12740600	2.08092400	-0.72718800
C	-0.84321800	3.44079700	-0.70652300
C	-0.01493300	3.96502100	0.28261200
C	0.53644200	3.12356400	1.25020900
C	0.27041600	1.76259400	1.22455600
H	-3.13704900	1.14825800	0.94086900
H	-5.47528600	0.39235800	0.71091500
H	-5.97724900	-1.81458400	-0.31109500
H	-4.12350700	-3.25326400	-1.12265200
H	-1.78543600	-2.48217600	-0.93565100
H	-1.75610900	1.66396500	-1.50733700
H	-1.26681800	4.09101900	-1.46637700
H	0.20114200	5.02965100	0.30294900
H	1.17157700	3.53266900	2.03065100
H	0.69332300	1.10871800	1.98162900
C	1.74937300	-0.84116300	0.09255800
C	2.81724000	-1.25294900	0.96254500
C	1.90568700	-0.20317900	-1.19092500
C	4.23336200	-0.99412400	0.47982300
C	3.34320200	0.06876100	-1.61107700
C	4.34211900	-0.92860900	-1.03839100
H	4.87502300	-1.76915100	0.91083000
H	4.55526400	-0.03815300	0.92007700
H	3.36021000	0.08934200	-2.70508900
H	3.59056700	1.08557000	-1.26969400
H	5.36124000	-0.65437800	-1.33403300
H	4.14627600	-1.92254600	-1.46142700
O	2.61037900	-1.78024400	2.06042900
O	0.97109900	0.12295200	-1.92515300

C=S-ylide 7f

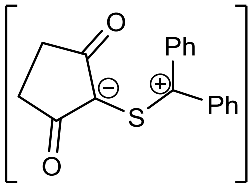

Sum of electronic and zero-point Energies= -1241.554463
Sum of electronic and thermal Energies= -1241.536699
Sum of electronic and thermal Enthalpies= -1241.535755
Sum of electronic and thermal Free Energies= -1241.602373

Standard orientation. Coordinates (Angstroms):

C	0.740512000	-0.209806000	-0.218500000
S	-0.307652000	-1.500838000	-0.486588000
C	2.142869000	-0.566832000	-0.051669000
C	3.148547000	0.292835000	-0.540255000
C	4.484321000	-0.066491000	-0.451168000
C	4.848207000	-1.280103000	0.133516000
C	3.866885000	-2.133784000	0.633098000
C	2.527042000	-1.782217000	0.547764000
C	0.324338000	1.189801000	-0.225515000
C	0.876903000	2.092223000	0.696830000
C	0.526445000	3.435999000	0.660801000
C	-0.367570000	3.897703000	-0.301595000
C	-0.918068000	3.008933000	-1.226788000
C	-0.586055000	1.663585000	-1.184517000
H	2.866496000	1.225234000	-1.018431000
H	5.247009000	0.598941000	-0.844930000
H	5.896713000	-1.554139000	0.208460000
H	4.147526000	-3.068134000	1.110102000
H	1.769544000	-2.424286000	0.986391000
H	1.559793000	1.725320000	1.456527000
H	0.949714000	4.122397000	1.388232000
H	-0.636839000	4.949766000	-0.333790000
H	-1.605104000	3.369392000	-1.986937000
H	-1.010578000	0.975198000	-1.909035000
C	-1.871219000	-1.018567000	-0.009194000
C	-3.041606000	-1.538242000	-0.660261000
C	-2.175761000	-0.270335000	1.180179000
C	-4.251243000	-1.089934000	0.161056000
C	-3.699994000	-0.248872000	1.316311000
H	-4.789384000	-1.980643000	0.504118000
H	-4.939148000	-0.538923000	-0.489265000
H	-3.972828000	-0.634213000	2.304420000
H	-4.033371000	0.794729000	1.281732000
O	-3.100169000	-2.220916000	-1.678133000
O	-1.396637000	0.245591000	1.974039000

7. Transition states for 1,5-electrocyclizations of C=S-ylides 7 to oxathioles 3

Benzene (PCM), 6-31G(d), PBE1PBE

TS$_{7a\rightarrow3a}$

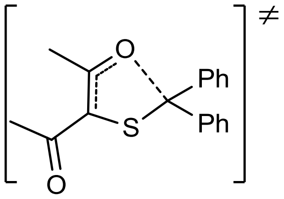

Imaginary Freq.: -144.42 cm^{-1}
Sum of electronic and zero-point Energies:-1242.714664
Sum of electronic and thermal Energies: -1242.696025
Sum of electronic and thermal Enthalpies: -1242.695081
Sum of electronic and thermal Free Energies: -1242.762247

Standard orientation. Coordinates (Angstroms):

S	0.444119000	-1.286143000	-0.977106000
C	1.880058000	-0.959493000	-0.019331000
C	3.110730000	-1.206204000	-0.727630000
O	3.082337000	-1.516641000	-1.924013000
C	4.456328000	-1.080549000	-0.041496000
H	4.529775000	-0.191281000	0.590139000
H	4.657918000	-1.956341000	0.586408000
H	5.220023000	-1.039302000	-0.820772000
C	1.570170000	-0.716763000	1.346321000
O	0.403336000	-0.437723000	1.719911000
C	2.615467000	-0.801099000	2.438240000
H	3.301206000	-1.640797000	2.313448000
H	3.205482000	0.122318000	2.463146000
H	2.093463000	-0.892750000	3.392573000
C	-0.602705000	-0.137006000	-0.268731000
C	-2.002352000	-0.542634000	-0.140594000
C	-3.042935000	0.347509000	-0.464196000
C	-2.328561000	-1.851798000	0.255600000
C	-4.365630000	-0.069681000	-0.409747000
H	-2.804696000	1.354681000	-0.790153000
C	-3.653377000	-2.259254000	0.318309000
H	-1.530559000	-2.525006000	0.551276000
C	-4.674404000	-1.371389000	-0.017495000
H	-5.158991000	0.621432000	-0.679213000
H	-3.891930000	-3.268581000	0.640681000
H	-5.711364000	-1.691967000	0.032104000
C	-0.240617000	1.286595000	-0.183939000
C	-0.784882000	2.089703000	0.830553000
C	0.620039000	1.867305000	-1.125459000
C	-0.479147000	3.442650000	0.895085000
H	-1.422329000	1.634118000	1.581443000
C	0.917899000	3.222956000	-1.060557000
H	1.038559000	1.252565000	-1.917043000
C	0.370669000	4.013182000	-0.051610000
H	-0.899983000	4.052849000	1.689241000
H	1.576012000	3.664104000	-1.803660000
H	0.605626000	5.072985000	-0.003049000

TS $_{7b\rightarrow 3b}$

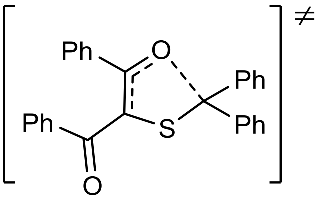

Imaginary Freq.: -32.95 cm^{-1}
Sum of electronic and zero-point Energies= -1625.624116
Sum of electronic and thermal Energies= -1625.598988
Sum of electronic and thermal Enthalpies= -1625.598044
Sum of electronic and thermal Free Energies= -1625.682288

Standard orientation. Coordinates (Angstroms):

C	1.579562000	-0.738980000	-0.474398000
S	0.457434000	0.196366000	-1.314053000
C	-1.095589000	0.292592000	-0.501998000
C	-2.049348000	-0.601684000	-1.079939000
C	-1.395090000	1.434737000	0.339546000
O	-2.536394000	1.715193000	0.704259000
O	-1.702459000	-1.520386000	-1.846736000
C	1.260981000	-1.666378000	0.596868000
C	0.102588000	-2.463177000	0.552167000
C	-0.163240000	-3.348277000	1.587622000
C	0.692704000	-3.427468000	2.685745000
C	1.840316000	-2.636438000	2.741705000
C	2.135820000	-1.773022000	1.697338000
C	2.964177000	-0.570421000	-0.905202000
C	3.820197000	-1.688481000	-0.950783000
C	5.121258000	-1.553855000	-1.411296000
C	5.595318000	-0.304627000	-1.811995000
C	4.763699000	0.812811000	-1.753928000
C	3.455657000	0.685595000	-1.309183000
H	-0.542819000	-2.417288000	-0.321679000
H	-1.043879000	-3.981918000	1.537243000
H	0.468560000	-4.112337000	3.498859000
H	2.505777000	-2.697442000	3.597581000
H	3.025741000	-1.151918000	1.737485000
H	3.441800000	-2.662696000	-0.657837000
H	5.767764000	-2.424804000	-1.461686000
H	6.618141000	-0.200948000	-2.163019000
H	5.140002000	1.788783000	-2.045199000
H	2.819555000	1.561184000	-1.224655000
C	-3.514628000	-0.478886000	-0.769713000
C	-4.335519000	0.341931000	-1.544262000
C	-4.085099000	-1.290347000	0.211004000
C	-5.710014000	0.357367000	-1.332378000
H	-3.894467000	0.972744000	-2.311654000
C	-5.459972000	-1.268502000	0.427506000
H	-3.448590000	-1.935507000	0.811787000
C	-6.276218000	-0.445684000	-0.344244000
H	-6.341969000	1.000265000	-1.939833000
H	-5.894985000	-1.898636000	1.199196000
H	-7.350068000	-0.430326000	-0.177346000
C	-0.281155000	2.358423000	0.765175000
C	-0.410089000	3.720139000	0.475376000
C	0.814613000	1.925307000	1.516098000
C	0.562104000	4.625056000	0.888048000
H	-1.282544000	4.060776000	-0.075230000
C	1.776999000	2.834112000	1.949670000

H	0.897885000	0.876571000	1.789207000
C	1.659368000	4.183816000	1.625999000
H	0.458717000	5.678936000	0.643318000
H	2.616903000	2.488186000	2.546978000
H	2.413940000	4.892112000	1.957861000

$TS_{7c\rightarrow 3c}$

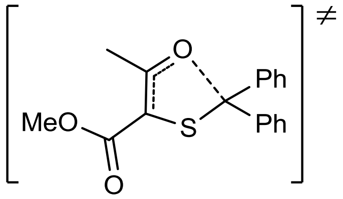

Imaginary Freq.: -152.44 cm^{-1}
Sum of electronic and zero-point Energies:-1317.865117
Sum of electronic and thermal Energies: -1317.845366
Sum of electronic and thermal Enthalpies: -1317.844422
Sum of electronic and thermal Free Energies: -1317.914479

Standard orientation. Coordinates (Angstroms):

S	0.301291000	-1.212951000	-0.932079000
C	1.670993000	-0.757761000	0.053552000
C	2.954765000	-0.901833000	-0.583679000
O	3.105810000	-1.201342000	-1.760864000
C	1.317604000	-0.524803000	1.407639000
O	0.121267000	-0.344456000	1.749121000
C	2.358440000	-0.498761000	2.503217000
H	3.047997000	-1.342220000	2.435302000
H	2.956905000	0.413965000	2.416862000
H	1.843116000	-0.499775000	3.465460000
C	-0.853078000	-0.144917000	-0.258173000
C	-2.215428000	-0.668014000	-0.158920000
C	-3.318925000	0.127312000	-0.519208000
C	-2.439883000	-1.996471000	0.243815000
C	-4.602214000	-0.400792000	-0.494978000
H	-3.158442000	1.148450000	-0.849175000
C	-3.726470000	-2.515229000	0.275102000
H	-1.596344000	-2.595844000	0.570380000
C	-4.809781000	-1.720903000	-0.097403000
H	-5.443903000	0.217996000	-0.792259000
H	-3.886936000	-3.538242000	0.602630000
H	-5.816708000	-2.128272000	-0.071568000
C	-0.614235000	1.304586000	-0.183366000
C	-1.245572000	2.067514000	0.811346000
C	0.211120000	1.948027000	-1.115517000
C	-1.059851000	3.442639000	0.864990000
H	-1.856034000	1.566053000	1.555369000
C	0.388987000	3.324882000	-1.061503000
H	0.697399000	1.364093000	-1.891485000
C	-0.244297000	4.074942000	-0.072375000
H	-1.547448000	4.022116000	1.643812000
H	1.021247000	3.813832000	-1.797086000
H	-0.102605000	5.151515000	-0.031863000
O	4.006634000	-0.665975000	0.235916000
C	5.281642000	-0.816922000	-0.377515000
H	5.400525000	-0.115531000	-1.207918000
H	6.011005000	-0.603745000	0.405081000
H	5.416979000	-1.833963000	-0.755815000

$TS_{7d\rightarrow 3d}$

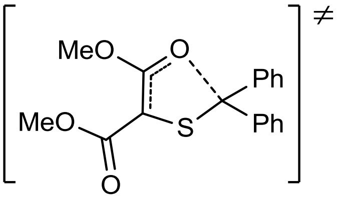

Imaginary Freq.: -281.44 cm^{-1}
Sum of electronic and zero-point Energies:-1392.999272
Sum of electronic and thermal Energies: -1392.978471
Sum of electronic and thermal Enthalpies: -1392.977527
Sum of electronic and thermal Free Energies: -1393.050262

Standard orientation. Coordinates (Angstroms):

S	0.208263000	-1.107543000	-1.250937000
C	1.602237000	-0.730314000	-0.255895000
C	2.887266000	-0.807250000	-0.892467000
O	3.035171000	-1.018739000	-2.090552000
C	1.226825000	-0.618660000	1.098942000
O	0.016510000	-0.493350000	1.451867000
C	1.752472000	-0.459766000	3.374724000
H	2.668567000	-0.429591000	3.965149000
H	1.205685000	0.481900000	3.470797000
H	1.114896000	-1.281711000	3.710920000
C	-0.892153000	-0.118125000	-0.353207000
C	-2.252093000	-0.659237000	-0.231971000
C	-3.373796000	0.147356000	-0.488629000
C	-2.446434000	-2.015941000	0.077691000
C	-4.651474000	-0.395984000	-0.450587000
H	-3.236886000	1.191593000	-0.749049000
C	-3.726294000	-2.549633000	0.127654000
H	-1.583483000	-2.629987000	0.313045000
C	-4.831125000	-1.742451000	-0.140113000
H	-5.509592000	0.233216000	-0.668302000
H	-3.864018000	-3.596291000	0.382850000
H	-5.832558000	-2.162365000	-0.103083000
C	-0.679608000	1.340404000	-0.239323000
C	-1.257738000	2.046669000	0.826577000
C	0.058761000	2.039865000	-1.201160000
C	-1.104180000	3.423469000	0.921263000
H	-1.804023000	1.501280000	1.589662000
C	0.206022000	3.418872000	-1.104790000
H	0.504986000	1.498016000	-2.029649000
C	-0.373738000	4.113283000	-0.045762000
H	-1.551321000	3.959805000	1.753501000
H	0.774737000	3.951515000	-1.861500000
H	-0.256623000	5.191066000	0.027290000
O	3.933402000	-0.617751000	-0.058775000
C	5.207028000	-0.707212000	-0.683433000
H	5.317747000	0.052881000	-1.462313000
H	5.935545000	-0.541482000	0.111533000
H	5.356680000	-1.692681000	-1.134114000
O	2.179364000	-0.664979000	2.030107000

8. Oxathioles 3

Benzene (PCM), 6-31G(d), PBE1PBE

Oxathiole 3a

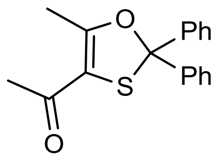

Sum of electronic and zero-point Energies:-1242.748529
Sum of electronic and thermal Energies: -1242.729640
Sum of electronic and thermal Enthalpies: -1242.728696
Sum of electronic and thermal Free Energies: -1242.797886

Standard orientation. Coordinates (Angstroms):

S	0.789187000	-0.818529000	-1.265265000
C	2.131602000	-0.664961000	-0.112012000
C	3.471817000	-0.841794000	-0.668375000
O	3.577386000	-1.103198000	-1.860573000
C	4.703986000	-0.710875000	0.195466000
H	5.578072000	-0.745794000	-0.457392000
H	4.709231000	0.226405000	0.760623000
H	4.769174000	-1.539946000	0.909450000
C	1.668972000	-0.508993000	1.152289000
O	0.323297000	-0.447893000	1.281143000
C	2.355487000	-0.424936000	2.466888000
H	1.885028000	-1.131654000	3.159607000
H	3.417657000	-0.649703000	2.404058000
H	2.226548000	0.577923000	2.890896000
C	-0.341131000	-0.150335000	0.033315000
C	-1.661350000	-0.893217000	-0.007675000
C	-2.661864000	-0.476809000	-0.889188000
C	-1.877590000	-2.018574000	0.789356000
C	-3.858852000	-1.180832000	-0.976584000
H	-2.506146000	0.404700000	-1.505210000
C	-3.082047000	-2.711147000	0.711246000
H	-1.102092000	-2.345812000	1.473654000
C	-4.074581000	-2.297860000	-0.173799000
H	-4.627440000	-0.848967000	-1.669207000
H	-3.242833000	-3.580158000	1.343631000
H	-5.012605000	-2.842697000	-0.236265000
C	-0.526945000	1.365707000	-0.014526000
C	-0.076124000	2.166281000	-1.060429000
C	-1.191532000	1.962905000	1.064102000
C	-0.286783000	3.545175000	-1.032770000
H	0.444179000	1.710086000	-1.897074000
C	-1.395444000	3.336298000	1.093848000
H	-1.548148000	1.342460000	1.881936000
C	-0.943683000	4.133355000	0.041751000
H	0.070462000	4.157481000	-1.856375000
H	-1.911533000	3.786820000	1.937470000
H	-1.105637000	5.207709000	0.062468000

Oxathiole 3b

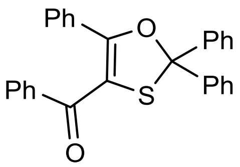

Sum of electronic and zero-point Energies= -1625.669899
Sum of electronic and thermal Energies= -1625.644892
Sum of electronic and thermal Enthalpies= -1625.643948
Sum of electronic and thermal Free Energies= -1625.728201

Standard orientation. Coordinates (Angstroms):

C	-1.807347000	0.066591000	-0.295724000
S	-1.024610000	0.452065000	-1.920194000
C	0.539101000	0.027079000	-1.194559000
C	1.737585000	0.446934000	-1.931616000
C	0.355296000	-0.757106000	-0.098362000
O	-0.937916000	-0.934918000	0.276355000
O	1.646914000	0.636892000	-3.140786000
C	-1.841155000	1.237019000	0.684911000
C	-1.508463000	2.545130000	0.343277000
C	-1.558248000	3.557400000	1.302293000
C	-1.936178000	3.269148000	2.608531000
C	-2.270325000	1.960320000	2.956950000
C	-2.225965000	0.952981000	2.001741000
C	-3.173453000	-0.546096000	-0.521907000
C	-4.289606000	0.282017000	-0.660849000
C	-5.541298000	-0.265437000	-0.925721000
C	-5.691586000	-1.644866000	-1.039993000
C	-4.581219000	-2.473237000	-0.895856000
C	-3.325487000	-1.928321000	-0.646726000
H	-1.209116000	2.773567000	-0.675155000
H	-1.295581000	4.573660000	1.021211000
H	-1.973324000	4.058773000	3.354121000
H	-2.571075000	1.725983000	3.974592000
H	-2.494805000	-0.064941000	2.271947000
H	-4.179939000	1.358010000	-0.556225000
H	-6.401422000	0.389285000	-1.035461000
H	-6.670255000	-2.072986000	-1.239006000
H	-4.690319000	-3.551014000	-0.981516000
H	-2.459013000	-2.572753000	-0.543211000
C	3.021960000	0.717427000	-1.224083000
C	3.066336000	1.153991000	0.103443000
C	4.209016000	0.615584000	-1.957754000
C	4.286256000	1.466594000	0.693803000
H	2.143754000	1.265997000	0.665054000
C	5.427698000	0.909861000	-1.360560000
H	4.154587000	0.303435000	-2.996432000
C	5.467479000	1.336122000	-0.033021000
H	4.314397000	1.814731000	1.722506000
H	6.348098000	0.815023000	-1.930201000
H	6.420292000	1.573943000	0.432486000
C	1.304180000	-1.528162000	0.707812000
C	2.402096000	-2.171492000	0.122931000
C	1.084875000	-1.659819000	2.085983000
C	3.281289000	-2.905942000	0.908540000
H	2.552973000	-2.110270000	-0.950171000
C	1.968316000	-2.394183000	2.867207000
H	0.226563000	-1.171007000	2.536565000
C	3.069472000	-3.015929000	2.281448000

H	4.128386000	-3.403534000	0.445267000
H	1.796690000	-2.482819000	3.936311000
H	3.757132000	-3.593556000	2.893042000

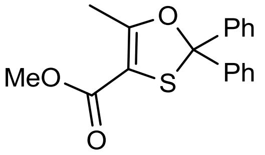

__Oxathiole 3c__

Sum of electronic and zero-point Energies:-1317.899537
Sum of electronic and thermal Energies: -1317.879574
Sum of electronic and thermal Enthalpies: -1317.878630
Sum of electronic and thermal Free Energies: -1317.950571

Standard orientation. Coordinates (Angstroms):

S	0.563162000	-0.736796000	-1.254183000
C	1.876781000	-0.498138000	-0.088262000
C	3.240648000	-0.576390000	-0.600712000
O	3.493132000	-0.828813000	-1.762700000
C	5.528828000	-0.440405000	-0.155548000
H	6.163146000	-0.225117000	0.704260000
H	5.731903000	-1.443407000	-0.539280000
H	5.703181000	0.287814000	-0.951454000
C	1.403266000	-0.385829000	1.173307000
O	0.053619000	-0.426244000	1.291802000
C	2.100078000	-0.268394000	2.477382000
H	1.725844000	-1.041710000	3.158042000
H	3.176619000	-0.371656000	2.359095000
H	1.874261000	0.702979000	2.932856000
C	-0.617830000	-0.150722000	0.046267000
C	-1.890926000	-0.973194000	-0.008731000
C	-2.964587000	-0.559747000	-0.799663000
C	-1.983978000	-2.176754000	0.693993000
C	-4.112990000	-1.341750000	-0.890016000
H	-2.906724000	0.379567000	-1.342061000
C	-3.138246000	-2.948228000	0.614032000
H	-1.150638000	-2.501989000	1.307737000
C	-4.205303000	-2.535701000	-0.181014000
H	-4.940168000	-1.009716000	-1.511420000
H	-3.202186000	-3.877795000	1.173234000
H	-5.104954000	-3.141624000	-0.245645000
C	-0.886195000	1.352241000	-0.001741000
C	-0.658983000	2.134477000	-1.131809000
C	-1.425511000	1.950279000	1.143446000
C	-0.963905000	3.495216000	-1.119765000
H	-0.231607000	1.681132000	-2.021130000
C	-1.721927000	3.307646000	1.156711000
H	-1.607351000	1.345125000	2.026971000
C	-1.492929000	4.085757000	0.022440000
H	-0.778252000	4.092912000	-2.008093000
H	-2.135353000	3.759847000	2.054283000
H	-1.725794000	5.147131000	0.032000000
O	4.188215000	-0.347058000	0.325350000

Oxathiole 3d

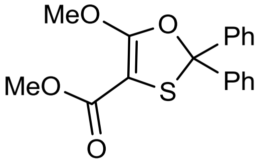

Sum of electronic and zero-point Energies:-1393.022424
Sum of electronic and thermal Energies: -1393.001318
Sum of electronic and thermal Enthalpies: -1393.000374
Sum of electronic and thermal Free Energies: -1393.075192

Standard orientation. Coordinates (Angstroms):

S	0.396787000	-0.874531000	-1.410208000
C	1.774079000	-0.619085000	-0.319520000
C	3.119320000	-0.690093000	-0.849291000
O	3.364063000	-0.898948000	-2.024640000
C	5.405724000	-0.583340000	-0.405930000
H	6.045751000	-0.422759000	0.462043000
H	5.602776000	-1.563755000	-0.848021000
H	5.587378000	0.187108000	-1.160251000
O	4.070685000	-0.508349000	0.084722000
C	1.330831000	-0.470080000	0.956531000
O	-0.001592000	-0.455697000	1.141612000
O	2.069569000	-0.382694000	2.043062000
C	1.440461000	0.071811000	3.245327000
H	2.248488000	0.155159000	3.971248000
H	0.968864000	1.045772000	3.091186000
H	0.697214000	-0.650084000	3.592011000
C	-0.697012000	-0.165950000	-0.101381000
C	-2.019528000	-0.901561000	-0.097983000
C	-3.051932000	-0.465690000	-0.931664000
C	-2.206432000	-2.042618000	0.684616000
C	-4.254064000	-1.164921000	-0.984157000
H	-2.916584000	0.425722000	-1.537858000
C	-3.415440000	-2.729605000	0.642060000
H	-1.404503000	-2.388340000	1.328210000
C	-4.441264000	-2.295862000	-0.194259000
H	-5.048256000	-0.818223000	-1.639490000
H	-3.553469000	-3.610880000	1.262566000
H	-5.382944000	-2.836750000	-0.229338000
C	-0.861972000	1.350287000	-0.159684000
C	-0.370769000	2.138673000	-1.196378000
C	-1.539175000	1.962518000	0.902913000
C	-0.552755000	3.521561000	-1.174443000
H	0.155970000	1.668772000	-2.021324000
C	-1.715984000	3.339886000	0.925925000
H	-1.931144000	1.350913000	1.711499000
C	-1.222384000	4.124975000	-0.116289000
H	-0.163395000	4.125002000	-1.989828000
H	-2.244181000	3.802483000	1.755330000
H	-1.362585000	5.202417000	-0.101076000

9. Transition states for 1,3-electrocyclizations of C=S-ylides 7 to thiiranes 4

Benzene (PCM), 6-31G(d), PBE1PBE

TS$_{7a\rightarrow4a}$

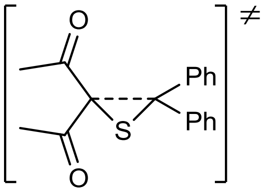

Imaginary Freq.: -109.20 cm^{-1}
Sum of electronic and zero-point Energies:-1242.694654
Sum of electronic and thermal Energies: -1242.675977
Sum of electronic and thermal Enthalpies: -1242.675032
Sum of electronic and thermal Free Energies: -1242.741867

Standard orientation. Coordinates (Angstroms):

S	0.105839000	-1.012795000	-1.744578000
C	0.909064000	-1.566010000	-0.273572000
C	2.366195000	-1.391579000	-0.254585000
O	2.929914000	-0.776988000	-1.156609000
C	3.214705000	-2.002953000	0.841970000
H	2.931533000	-3.032498000	1.075651000
H	4.251738000	-1.979968000	0.501792000
H	3.143541000	-1.416624000	1.764639000
C	0.078500000	-2.307052000	0.686542000
O	-0.988675000	-2.819686000	0.357446000
C	0.498753000	-2.411786000	2.141704000
H	1.066434000	-1.546560000	2.491756000
H	-0.415569000	-2.513626000	2.731252000
H	1.100886000	-3.310666000	2.313476000
C	-0.390066000	0.237237000	-0.627383000
C	-1.797061000	0.364011000	-0.276672000
C	-2.707864000	-0.684799000	-0.528971000
C	-2.309026000	1.583374000	0.229708000
C	-4.061521000	-0.526169000	-0.262544000
H	-2.328292000	-1.629573000	-0.896763000
C	-3.662123000	1.734339000	0.479403000
H	-1.643892000	2.425900000	0.379879000
C	-4.543631000	0.677166000	0.243724000
H	-4.742717000	-1.350630000	-0.451779000
H	-4.036707000	2.684861000	0.848348000
H	-5.604293000	0.798625000	0.446387000
C	0.588532000	1.251165000	-0.201596000
C	1.520772000	1.765169000	-1.114520000
C	0.602290000	1.733468000	1.121196000
C	2.415698000	2.757180000	-0.725221000
H	1.530700000	1.385182000	-2.129666000
C	1.507013000	2.710365000	1.507896000
H	-0.085522000	1.312161000	1.849461000
C	2.411852000	3.233088000	0.581250000
H	3.124455000	3.150721000	-1.448030000
H	1.514496000	3.061666000	2.536005000
H	3.117380000	4.001861000	0.884177000

TS$_{7b\rightarrow 4b}$

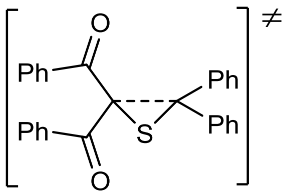

Imaginary Freq.: -61.48 cm^{-1}
Sum of electronic and zero-point Energies= -1625.613332
Sum of electronic and thermal Energies= -1625.588356
Sum of electronic and thermal Enthalpies= -1625.587412
Sum of electronic and thermal Free Energies= -1625.670375

Standard orientation. Coordinates (Angstroms):

C	1.454498000	-0.996657000	-0.420296000
S	0.357653000	-0.788229000	-1.767959000
C	-0.402564000	0.422054000	-0.738572000
C	0.204344000	1.743514000	-0.694437000
C	-1.538040000	0.003657000	0.105516000
O	-1.600689000	0.327452000	1.286824000
O	1.232163000	2.030732000	-1.316217000
C	0.947712000	-1.621519000	0.807677000
C	0.141602000	-2.768745000	0.732836000
C	-0.291842000	-3.400134000	1.891854000
C	0.049281000	-2.884542000	3.140618000
C	0.828220000	-1.731649000	3.227794000
C	1.277788000	-1.105971000	2.073365000
C	2.855450000	-0.677612000	-0.566891000
C	3.811411000	-1.230828000	0.322261000
C	5.163989000	-1.013407000	0.130249000
C	5.604998000	-0.237002000	-0.944169000
C	4.682429000	0.311849000	-1.831557000
C	3.323726000	0.098495000	-1.651383000
H	-0.125437000	-3.167503000	-0.241004000
H	-0.899362000	-4.297678000	1.818682000
H	-0.298786000	-3.375097000	4.045385000
H	1.074652000	-1.311362000	4.198480000
H	1.860252000	-0.191734000	2.139214000
H	3.482277000	-1.870275000	1.133471000
H	5.882426000	-1.460063000	0.811363000
H	6.668168000	-0.064191000	-1.087736000
H	5.022464000	0.921513000	-2.663415000
H	2.598823000	0.571141000	-2.300639000
C	-0.479177000	2.852201000	0.057060000
C	-1.859873000	3.064826000	0.024878000
C	0.335420000	3.781435000	0.710831000
C	-2.415809000	4.174624000	0.653166000
H	-2.506424000	2.373840000	-0.508701000
C	-0.221556000	4.876690000	1.360623000
H	1.410986000	3.631789000	0.697658000
C	-1.600428000	5.076671000	1.332396000
H	-3.489884000	4.334649000	0.611581000
H	0.421303000	5.581667000	1.881344000
H	-2.037582000	5.937571000	1.831471000
C	-2.620471000	-0.838336000	-0.494821000
C	-2.922406000	-0.794130000	-1.859261000
C	-3.401031000	-1.627913000	0.354962000
C	-3.982394000	-1.537477000	-2.368718000
H	-2.340233000	-0.155043000	-2.519740000
C	-4.451599000	-2.380885000	-0.155047000

H	-3.163958000	-1.635561000	1.414692000
C	-4.743512000	-2.336886000	-1.518190000
H	-4.217173000	-1.490366000	-3.428543000
H	-5.049400000	-2.999325000	0.509242000
H	-5.568341000	-2.921709000	-1.916776000

$TS_{7c\rightarrow 4c}$

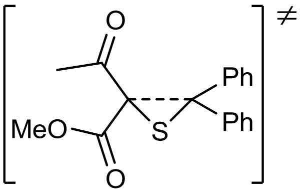

Imaginary Freq.: -105.58 cm^{-1}
Sum of electronic and zero-point Energies: -1317.847295
Sum of electronic and thermal Energies: -1317.827620
Sum of electronic and thermal Enthalpies: -1317.826676
Sum of electronic and thermal Free Energies: -1317.895942

Standard orientation. Coordinates (Angstroms):

S	-0.104167000	0.504167000	-1.989576000
C	-0.923121000	1.310750000	-0.659368000
C	-2.376814000	1.128091000	-0.587860000
O	-2.937415000	0.384274000	-1.389936000
C	-0.110331000	2.231964000	0.137854000
O	0.983552000	2.671834000	-0.175913000
C	0.396141000	-0.531103000	-0.673972000
C	1.802872000	-0.595291000	-0.304790000
C	2.715014000	0.395212000	-0.728070000
C	2.313089000	-1.714450000	0.396121000
C	4.068834000	0.278619000	-0.444274000
H	2.336460000	1.269492000	-1.241834000
C	3.666938000	-1.825785000	0.664474000
H	1.645890000	-2.518114000	0.685548000
C	4.550127000	-0.825915000	0.253412000
H	4.751337000	1.058754000	-0.768608000
H	4.039795000	-2.702545000	1.186159000
H	5.611093000	-0.914638000	0.470831000
C	-0.583984000	-1.450135000	-0.075528000
C	-1.519855000	-2.113881000	-0.882922000
C	-0.595805000	-1.692200000	1.311519000
C	-2.414932000	-3.021036000	-0.324274000
H	-1.532077000	-1.917117000	-1.948938000
C	-1.500831000	-2.584792000	1.864637000
H	0.090808000	-1.147194000	1.953358000
C	-2.407925000	-3.260703000	1.045431000
H	-3.126217000	-3.533614000	-0.965302000
H	-1.508122000	-2.748963000	2.938581000
H	-3.113703000	-3.963261000	1.480011000
O	-0.697659000	2.573637000	1.305491000
C	0.019491000	3.530552000	2.078831000
H	0.182727000	4.449391000	1.509202000
H	-0.604081000	3.729355000	2.951148000
H	0.989168000	3.130770000	2.387081000
C	-3.206112000	1.848006000	0.451845000
H	-3.018044000	2.924778000	0.456915000
H	-4.256537000	1.650559000	0.229251000
H	-2.966415000	1.480294000	1.454262000

TS$_{7d\rightarrow 4d}$

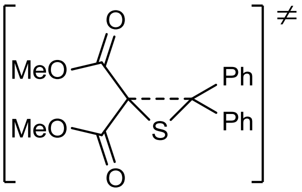

Imaginary Freq.: -94.78 cm^{-1}
Sum of electronic and zero-point Energies:-1392.992441
Sum of electronic and thermal Energies: -1392.971664
Sum of electronic and thermal Enthalpies: -1392.970720
Sum of electronic and thermal Free Energies: -1393.042906

Standard orientation. Coordinates (Angstroms):

S	-0.036016000	-0.455587000	-1.989010000
C	0.934401000	-1.098737000	-0.684714000
C	2.336239000	-0.677549000	-0.664084000
O	2.800400000	0.147083000	-1.435247000
C	0.306175000	-2.112838000	0.167832000
O	-0.685258000	-2.758825000	-0.135089000
C	-0.713877000	0.488131000	-0.683968000
C	-2.106290000	0.298808000	-0.312809000
C	-2.821007000	-0.849673000	-0.717068000
C	-2.812034000	1.316940000	0.373157000
C	-4.174443000	-0.975809000	-0.435111000
H	-2.287031000	-1.652138000	-1.210088000
C	-4.164263000	1.185091000	0.638713000
H	-2.300631000	2.231218000	0.651861000
C	-4.850179000	0.035076000	0.242855000
H	-4.702694000	-1.873161000	-0.743799000
H	-4.691799000	1.987027000	1.147117000
H	-5.910230000	-0.066748000	0.458946000
C	0.092145000	1.560876000	-0.081734000
C	0.894696000	2.383825000	-0.886263000
C	0.060606000	1.795059000	1.305829000
C	1.617553000	3.430019000	-0.322901000
H	0.940021000	2.198880000	-1.953658000
C	0.794642000	2.830625000	1.863773000
H	-0.518687000	1.133990000	1.944222000
C	1.569443000	3.658046000	1.048647000
H	2.226031000	4.064083000	-0.961210000
H	0.772791000	2.988499000	2.938427000
H	2.140964000	4.471805000	1.486575000
O	0.916687000	-2.267857000	1.356352000
O	3.090527000	-1.299112000	0.258811000
C	0.372260000	-3.296025000	2.175619000
H	0.404071000	-4.262030000	1.663999000
H	0.997325000	-3.319537000	3.068963000
H	-0.664924000	-3.074228000	2.442245000
C	4.459675000	-0.911693000	0.259155000
H	4.924857000	-1.483647000	1.062726000
H	4.932874000	-1.147058000	-0.698456000
H	4.559999000	0.161077000	0.446100000

10. Thiiranes 4

Benzene (PCM), 6-31G(d), PBE1PBE

Thiirane 4a

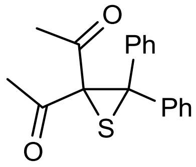

Sum of electronic and zero-point Energies:-1242.742269
Sum of electronic and thermal Energies: -1242.722969
Sum of electronic and thermal Enthalpies: -1242.722025
Sum of electronic and thermal Free Energies: -1242.790594

Standard orientation. Coordinates (Angstroms):

S	-0.027013000	1.080188000	1.909419000
C	-0.083865000	1.454333000	0.144748000
C	1.130291000	2.154802000	-0.478907000
O	1.075112000	2.503358000	-1.642046000
C	2.330093000	2.442705000	0.376138000
H	2.063881000	3.184299000	1.138669000
H	2.671956000	1.551204000	0.908482000
H	3.123133000	2.839073000	-0.260421000
C	-1.414678000	2.023676000	-0.384579000
O	-2.091907000	2.730565000	0.325542000
C	-1.784796000	1.690914000	-1.801479000
H	-1.758161000	0.608286000	-1.963250000
H	-2.783385000	2.077386000	-2.013873000
H	-1.048075000	2.141481000	-2.473372000
C	0.002933000	-0.035461000	0.458063000
C	-1.231072000	-0.887026000	0.225809000
C	-2.444858000	-0.622457000	0.871580000
C	-1.178664000	-1.954237000	-0.679654000
C	-3.574102000	-1.389314000	0.604748000
H	-2.501541000	0.186181000	1.593211000
C	-2.310331000	-2.721406000	-0.943341000
H	-0.247194000	-2.194510000	-1.181325000
C	-3.514227000	-2.441652000	-0.304874000
H	-4.503925000	-1.164599000	1.120079000
H	-2.243409000	-3.545017000	-1.649019000
H	-4.396248000	-3.042718000	-0.507866000
C	1.309992000	-0.738584000	0.189276000
C	1.953245000	-1.442897000	1.209171000
C	1.864598000	-0.745055000	-1.094633000
C	3.135431000	-2.130653000	0.956050000
H	1.527637000	-1.438677000	2.209117000
C	3.050274000	-1.432261000	-1.347093000
H	1.372336000	-0.208023000	-1.901118000
C	3.688519000	-2.125533000	-0.323144000
H	3.627999000	-2.668560000	1.761387000
H	3.474026000	-1.421260000	-2.347446000
H	4.614852000	-2.658294000	-0.519214000

Thiirane 4b

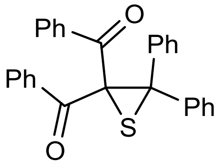

Sum of electronic and zero-point Energies= -1625.656251
Sum of electronic and thermal Energies= -1625.630767
Sum of electronic and thermal Enthalpies= -1625.629823
Sum of electronic and thermal Free Energies= -1625.714669

Standard orientation. Coordinates (Angstroms):

C	1.237667000	-0.274036000	0.335224000
S	0.068822000	-0.236309000	1.747920000
C	-0.230276000	-0.313657000	-0.041678000
C	-0.822401000	-1.650897000	-0.519601000
C	-0.892501000	0.806248000	-0.853104000
O	-1.128950000	0.501043000	-2.012059000
O	-0.154047000	-2.427372000	-1.165985000
C	2.001779000	1.003529000	0.083684000
C	2.764546000	1.569596000	1.109086000
C	3.521023000	2.712507000	0.878588000
C	3.532945000	3.301068000	-0.385461000
C	2.787926000	2.735786000	-1.414630000
C	2.027886000	1.590121000	-1.184263000
C	2.121689000	-1.499805000	0.205672000
C	2.923653000	-1.636680000	-0.933451000
C	3.772944000	-2.726429000	-1.074408000
C	3.850436000	-3.692110000	-0.072635000
C	3.068064000	-3.555490000	1.068465000
C	2.210345000	-2.466597000	1.206392000
H	2.754414000	1.111845000	2.095099000
H	4.102449000	3.145867000	1.687814000
H	4.121665000	4.196364000	-0.565019000
H	2.791450000	3.185414000	-2.403632000
H	1.453173000	1.155722000	-1.997334000
H	2.879863000	-0.888836000	-1.718968000
H	4.379557000	-2.819766000	-1.971160000
H	4.519939000	-4.541127000	-0.180750000
H	3.123320000	-4.296104000	1.861883000
H	1.599570000	-2.369526000	2.098683000
C	-2.241374000	-1.949692000	-0.168660000
C	-3.059465000	-1.075980000	0.555633000
C	-2.765301000	-3.170609000	-0.609742000
C	-4.379272000	-1.418542000	0.830632000
H	-2.678150000	-0.123022000	0.911073000
C	-4.080577000	-3.513982000	-0.330158000
H	-2.117660000	-3.834682000	-1.173532000
C	-4.890603000	-2.637138000	0.390954000
H	-5.007827000	-0.733106000	1.391909000
H	-4.478104000	-4.464265000	-0.675543000
H	-5.921177000	-2.903807000	0.609510000
C	-1.352659000	2.121847000	-0.326100000
C	-0.780960000	2.816933000	0.747589000
C	-2.431373000	2.699664000	-1.014648000
C	-1.284468000	4.058914000	1.120791000
H	0.069506000	2.407555000	1.276349000
C	-2.942019000	3.929902000	-0.626373000
H	-2.859431000	2.159983000	-1.853393000
C	-2.367442000	4.613638000	0.444273000

H	-0.824033000	4.595920000	1.945197000
H	-3.785275000	4.358606000	-1.160517000
H	-2.761704000	5.579890000	0.747536000

Thiirane 4c

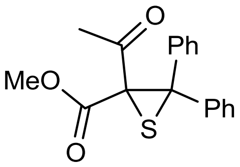

Sum of electronic and zero-point Energies:-1317.888105
Sum of electronic and thermal Energies: -1317.867472
Sum of electronic and thermal Enthalpies: -1317.866528
Sum of electronic and thermal Free Energies: -1317.939730

Standard orientation. Coordinates (Angstroms):

S	0.015897000	-0.695702000	2.104353000
C	0.472399000	-1.166314000	0.423355000
C	-0.217975000	-2.377977000	-0.212134000
O	-0.726217000	-2.280912000	-1.304507000
C	-0.182063000	-3.658197000	0.574041000
H	0.823891000	-3.849937000	0.964704000
H	-0.849163000	-3.573975000	1.439317000
H	-0.506181000	-4.487380000	-0.057006000
C	1.968680000	-1.137175000	0.140420000
O	2.815861000	-0.691708000	0.869941000
O	2.215218000	-1.699331000	-1.049136000
C	3.593448000	-1.726533000	-1.432422000
H	3.617457000	-2.225975000	-2.400142000
H	3.982673000	-0.708706000	-1.512609000
H	4.182039000	-2.279431000	-0.696594000
C	-0.269873000	0.152012000	0.507910000
C	0.499663000	1.426469000	0.267380000
C	0.439575000	2.472773000	1.188507000
C	1.209736000	1.608016000	-0.922526000
C	1.082759000	3.677814000	0.929384000
H	-0.107580000	2.335111000	2.117362000
C	1.853607000	2.815297000	-1.182495000
H	1.263404000	0.800130000	-1.648964000
C	1.791408000	3.853038000	-0.257756000
H	1.033565000	4.481764000	1.658642000
H	2.406407000	2.941284000	-2.109489000
H	2.295746000	4.794233000	-0.458433000
C	-1.704784000	0.231521000	0.025351000
C	-2.787777000	0.017933000	0.877364000
C	-1.956423000	0.574711000	-1.308236000
C	-4.095199000	0.135627000	0.409148000
H	-2.606694000	-0.244679000	1.915186000
C	-3.259140000	0.684774000	-1.776941000
H	-1.127752000	0.750999000	-1.986106000
C	-4.335987000	0.466956000	-0.919166000
H	-4.924841000	-0.030603000	1.091075000
H	-3.433748000	0.941745000	-2.818188000
H	-5.354800000	0.557810000	-1.285913000

Thiirane 4d

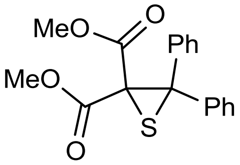

Sum of electronic and zero-point Energies:-1393.037894
Sum of electronic and thermal Energies: -1393.016406
Sum of electronic and thermal Enthalpies: -1393.015462
Sum of electronic and thermal Free Energies: -1393.090512

Standard orientation. Coordinates (Angstroms):

S	-0.391474000	-0.240456000	2.114838000
C	-0.641236000	-0.838171000	0.437941000
C	-2.034396000	-0.725938000	-0.152099000
O	-2.336319000	-0.062659000	-1.111435000
C	-4.211272000	-1.546192000	0.016607000
H	-4.753793000	-2.233509000	0.664733000
H	-4.655641000	-0.548817000	0.056725000
H	-4.219963000	-1.902381000	-1.016551000
O	-2.874718000	-1.515489000	0.521763000
C	-0.007886000	-2.186489000	0.122676000
O	0.715263000	-2.816473000	0.847769000
O	-0.385602000	-2.578033000	-1.101308000
C	0.137647000	-3.841775000	-1.521280000
H	-0.276435000	-4.014594000	-2.513863000
H	1.229059000	-3.804547000	-1.558666000
H	-0.172500000	-4.628835000	-0.830093000
C	0.240215000	0.393769000	0.516925000
C	1.717782000	0.218552000	0.272216000
C	2.644313000	0.712930000	1.191489000
C	2.179357000	-0.361073000	-0.912607000
C	4.007861000	0.621888000	0.937196000
H	2.290610000	1.161925000	2.115870000
C	3.545983000	-0.453148000	-1.167277000
H	1.467823000	-0.742205000	-1.641514000
C	4.463076000	0.037632000	-0.243486000
H	4.717868000	1.004380000	1.665300000
H	3.891182000	-0.911190000	-2.090208000
H	5.529077000	-0.036494000	-0.439948000
C	-0.295381000	1.730573000	0.040234000
C	-1.148391000	2.513350000	0.819257000
C	0.095120000	2.212171000	-1.214596000
C	-1.612079000	3.740770000	0.352894000
H	-1.450729000	2.158577000	1.799686000
C	-0.373109000	3.433993000	-1.682595000
H	0.771830000	1.630351000	-1.832039000
C	-1.229266000	4.205555000	-0.900617000
H	-2.271505000	4.335893000	0.979029000
H	-0.062736000	3.785673000	-2.662844000
H	-1.589599000	5.163908000	-1.264604000

11. Transition states for cycloadditions of diazo compounds 1 to aliphatic thioketone 2b

Gas phase, 6-31G(d), PBE1PBE

***TS**$_{1a+2b\rightarrow 6'a}$*

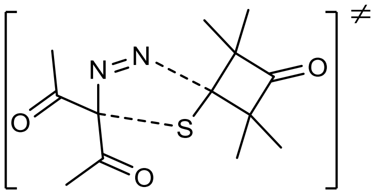

Imaginary Freq.: -361.00 cm^{-1}
Sum of electronic and zero-point Energies= -1238.067221
Sum of electronic and thermal Energies= -1238.045330
Sum of electronic and thermal Enthalpies= -1238.044386
Sum of electronic and thermal Free Energies= -1238.117394

Standard orientation. Coordinates (Angstroms):

S	-0.331688000	0.080242000	1.342851000
C	-2.029705000	0.036185000	-0.393154000
C	-2.839599000	1.242238000	-0.028604000
O	-3.736321000	1.143371000	0.781758000
C	-2.456219000	2.550076000	-0.669858000
H	-1.423483000	2.808657000	-0.409307000
H	-2.521752000	2.490864000	-1.762011000
H	-3.127638000	3.327414000	-0.302639000
C	-2.484923000	-1.402888000	-0.265148000
O	-2.025229000	-2.233560000	-1.019581000
C	-3.461811000	-1.723971000	0.825063000
H	-3.102298000	-1.356252000	1.790395000
H	-4.419088000	-1.227701000	0.640465000
H	-3.590452000	-2.807459000	0.851805000
C	0.981652000	0.041490000	0.311801000
C	1.939745000	-1.147583000	0.030491000
C	2.109984000	1.100294000	0.163726000
C	3.006896000	-0.078430000	-0.251824000
C	2.235649000	-1.974097000	1.285413000
H	2.471459000	-1.348794000	2.151649000
H	1.359857000	-2.581118000	1.538111000
H	3.084792000	-2.639509000	1.097533000
C	1.638726000	-2.079712000	-1.140433000
H	1.580725000	-1.547257000	-2.093325000
H	2.435470000	-2.825850000	-1.225039000
H	0.687628000	-2.598378000	-0.977013000
C	2.526488000	1.693656000	1.514242000
H	3.465039000	2.246840000	1.402021000
H	1.750266000	2.380091000	1.869411000
H	2.669172000	0.925660000	2.280017000
C	1.984672000	2.215407000	-0.867109000
H	1.193507000	2.916793000	-0.574945000
H	2.927037000	2.771108000	-0.914019000
H	1.768581000	1.839388000	-1.869677000
O	4.125199000	-0.132626000	-0.688494000
N	-1.114919000	0.190709000	-1.355704000
N	0.012016000	0.221888000	-1.595173000

$TS_{1b+2b \rightarrow 6'b}$

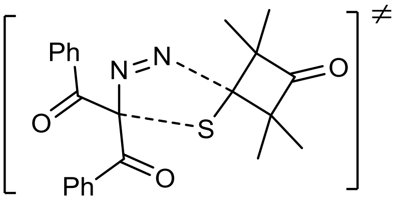

Imaginary Freq.: -333.62 cm^{-1}
Sum of electronic and zero-point Energies= -1238.067221
Sum of electronic and thermal Energies= -1238.045330
Sum of electronic and thermal Enthalpies= -1238.044386
Sum of electronic and thermal Free Energies= -1238.117394

Standard orientation. Coordinates (Angstroms):

S	-0.406569000	-1.027441000	-1.195195000
C	0.826958000	0.367820000	0.373675000
C	1.206922000	1.570758000	-0.443746000
O	1.933618000	1.445785000	-1.410087000
C	1.780294000	-0.622729000	1.022635000
O	1.516078000	-0.974818000	2.160083000
C	-1.828416000	-1.104835000	-0.332122000
C	-2.421494000	-2.309632000	0.444737000
C	-3.225764000	-0.555765000	-0.727198000
C	-3.792205000	-1.666024000	0.175076000
C	-2.227924000	-3.634883000	-0.299393000
H	-2.485308000	-3.560301000	-1.360012000
H	-1.178853000	-3.941733000	-0.230161000
H	-2.854732000	-4.409611000	0.154817000
C	-2.064397000	-2.492318000	1.917284000
H	-2.346140000	-1.629333000	2.525717000
H	-2.589644000	-3.366505000	2.315640000
H	-0.985942000	-2.654644000	2.026917000
C	-3.539482000	-0.792394000	-2.208943000
H	-4.603369000	-0.611656000	-2.396266000
H	-2.948716000	-0.102957000	-2.821690000
H	-3.301701000	-1.811157000	-2.528466000
C	-3.615522000	0.867443000	-0.344893000
H	-2.977713000	1.587329000	-0.871517000
H	-4.654317000	1.052283000	-0.637733000
H	-3.534059000	1.050247000	0.729102000
O	-4.901605000	-1.908485000	0.569088000
N	-0.264311000	0.519190000	1.147506000
N	-1.357569000	0.206634000	1.315620000
C	2.980695000	-1.134451000	0.317192000
C	3.142224000	-1.162023000	-1.071921000
C	3.976259000	-1.683770000	1.139078000
C	4.289490000	-1.722383000	-1.623629000
H	2.384213000	-0.743294000	-1.720095000
C	5.124191000	-2.227673000	0.583578000
H	3.824648000	-1.673491000	2.213708000
C	5.281711000	-2.248673000	-0.801907000
H	4.405940000	-1.746761000	-2.703268000
H	5.895295000	-2.639951000	1.228292000
H	6.178373000	-2.679016000	-1.240257000
C	0.640263000	2.901646000	-0.079811000
C	0.398712000	3.307854000	1.237604000
C	0.418377000	3.799736000	-1.131336000
C	-0.077979000	4.589507000	1.493728000
H	0.609351000	2.640133000	2.067609000
C	-0.076664000	5.069817000	-0.872470000

H	0.637367000	3.477263000	-2.144529000
C	-0.325927000	5.466096000	0.441301000
H	-0.251266000	4.904102000	2.518790000
H	-0.264598000	5.755619000	-1.693713000
H	-0.707214000	6.463107000	0.644560000

TS$_{1c+2b\rightarrow6'c}$

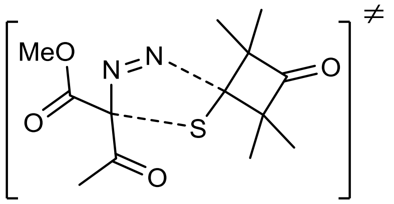

Imaginary Freq.: -364.64 cm^{-1}
Sum of electronic and zero-point Energies= -1313.215254
Sum of electronic and thermal Energies= -1313.192369
Sum of electronic and thermal Enthalpies= -1313.191425
Sum of electronic and thermal Free Energies= -1313.267237

Standard orientation. Coordinates (Angstroms):

S	-0.133062000	-0.226662000	1.357000000
C	-1.814952000	-0.450790000	-0.377296000
C	-2.764738000	0.624389000	0.010946000
O	-3.642913000	0.478452000	0.827110000
C	-3.315320000	2.873554000	-0.250607000
H	-2.984124000	3.704995000	-0.871813000
H	-4.368225000	2.648532000	-0.436254000
H	-3.177367000	3.103349000	0.808688000
O	-2.493713000	1.766224000	-0.627835000
C	-2.093159000	-1.931274000	-0.269087000
O	-1.526230000	-2.690827000	-1.025160000
C	-3.032941000	-2.378128000	0.810225000
H	-2.721904000	-1.985562000	1.782958000
H	-4.040620000	-1.993538000	0.626452000
H	-3.036507000	-3.469277000	0.823251000
C	1.152829000	0.012807000	0.318890000
C	2.323179000	-0.956577000	0.003714000
C	2.044484000	1.277249000	0.184326000
C	3.149804000	0.310228000	-0.275273000
C	2.802007000	-1.718476000	1.243486000
H	2.923489000	-1.067099000	2.114002000
H	2.070145000	-2.491090000	1.502125000
H	3.763857000	-2.198137000	1.033706000
C	2.189718000	-1.921435000	-1.171361000
H	2.023611000	-1.403937000	-2.119530000
H	3.108864000	-2.508178000	-1.269120000
H	1.352642000	-2.608205000	-1.001918000
C	2.356667000	1.913594000	1.543120000
H	3.168366000	2.640665000	1.433518000
H	1.466584000	2.429378000	1.919344000
H	2.655602000	1.173926000	2.291514000
C	1.660380000	2.364260000	-0.813262000
H	0.699831000	2.810398000	-0.529199000
H	2.420939000	3.151875000	-0.808162000
H	1.578933000	1.985487000	-1.834774000
O	4.246164000	0.486659000	-0.734837000
N	-0.939283000	-0.152204000	-1.342067000
N	0.169379000	0.046855000	-1.577637000

TS $_{\mathbf{1d+2b\rightarrow 6'd}}$

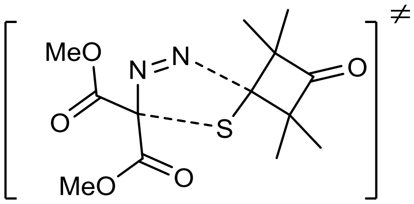

Imaginary Freq.: -363.52 cm^{-1}
Sum of electronic and zero-point Energies= -1388.358216
Sum of electronic and thermal Energies= -1388.334322
Sum of electronic and thermal Enthalpies= -1388.333378
Sum of electronic and thermal Free Energies= -1388.411925

Standard orientation. Coordinates (Angstroms):

S	-0.027489000	-0.171670000	1.315527000
C	-1.681352000	0.051645000	-0.434896000
C	-2.388627000	1.295940000	-0.008884000
O	-3.301713000	1.325472000	0.773463000
C	-2.386951000	3.619578000	-0.177597000
H	-1.848702000	4.376767000	-0.746925000
H	-3.455808000	3.655810000	-0.401435000
H	-2.239569000	3.765151000	0.895366000
O	-1.832700000	2.368449000	-0.588489000
C	-2.275762000	-1.320647000	-0.435892000
O	-1.893734000	-2.191952000	-1.182699000
O	-3.206136000	-1.450448000	0.497037000
C	-3.748871000	-2.766665000	0.618981000
H	-4.490078000	-2.698938000	1.414203000
H	-4.214136000	-3.076484000	-0.319896000
H	-2.961749000	-3.477484000	0.882342000
C	1.311344000	-0.095516000	0.324955000
C	2.300039000	-1.232717000	-0.043133000
C	2.409004000	0.999902000	0.273845000
C	3.337692000	-0.114311000	-0.241162000
C	2.621836000	-2.134805000	1.152947000
H	2.841911000	-1.564349000	2.060092000
H	1.762881000	-2.780931000	1.363400000
H	3.488692000	-2.762437000	0.920541000
C	2.015619000	-2.092880000	-1.271079000
H	1.962882000	-1.503812000	-2.190151000
H	2.813615000	-2.832157000	-1.395254000
H	1.064676000	-2.623499000	-1.145517000
C	2.802443000	1.491388000	1.671123000
H	3.727333000	2.075060000	1.612333000
H	2.006109000	2.127505000	2.072449000
H	2.958590000	0.668328000	2.374491000
C	2.231366000	2.194195000	-0.657224000
H	1.351485000	2.774779000	-0.355614000
H	3.111194000	2.843021000	-0.595614000
H	2.108927000	1.895988000	-1.701190000
O	4.453834000	-0.103449000	-0.686805000
N	-0.749041000	0.198234000	-1.383444000
N	0.378823000	0.220222000	-1.605701000

TS$_{1e+2b \rightarrow 6'e}$

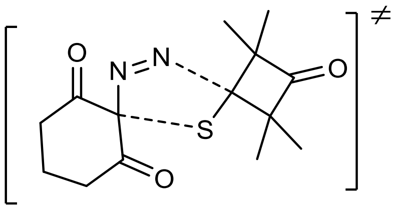

Imaginary Freq.: -363.64 cm^{-1}
Sum of electronic and zero-point Energies= -1276.131079
Sum of electronic and thermal Energies= -1276.110278
Sum of electronic and thermal Enthalpies= -1276.109333
Sum of electronic and thermal Free Energies= -1276.180233

Standard orientation. Coordinates (Angstroms):

S	-0.180689000	0.000000000	1.263018000
C	-1.758994000	0.000000000	-0.599139000
C	-2.424421000	1.318970000	-0.361461000
C	-2.424421000	-1.318970000	-0.361461000
C	1.184026000	0.000000000	0.295838000
C	2.237732000	-1.129807000	0.139891000
C	2.237732000	1.129807000	0.139891000
C	3.234153000	0.000000000	-0.176250000
C	2.543673000	-1.828150000	1.469064000
H	2.693510000	-1.120583000	2.289713000
H	1.707994000	-2.482953000	1.738237000
H	3.449692000	-2.434645000	1.366520000
C	2.048945000	-2.175810000	-0.954526000
H	2.011587000	-1.734744000	-1.953514000
H	2.884108000	-2.883302000	-0.933856000
H	1.119177000	-2.731828000	-0.785554000
C	2.543673000	1.828150000	1.469064000
H	3.449692000	2.434645000	1.366520000
H	1.707994000	2.482953000	1.738237000
H	2.693510000	1.120583000	2.289713000
C	2.048944000	2.175810000	-0.954526000
H	1.119177000	2.731828000	-0.785554000
H	2.884108000	2.883302000	-0.933856000
H	2.011587000	1.734744000	-1.953514000
O	4.367641000	0.000000000	-0.574897000
N	-0.776020000	0.000000000	-1.501511000
N	0.367158000	0.000000000	-1.638854000
O	-2.098691000	-2.315961000	-0.966902000
O	-2.098691000	2.315961000	-0.966902000
C	-4.343966000	0.000000000	0.612913000
C	-3.495127000	-1.269932000	0.702732000
C	-3.495127000	1.269932000	0.702732000
H	-5.084861000	0.000000000	1.418832000
H	-4.907748000	0.000000000	-0.328977000
H	-4.096813000	2.177549000	0.603662000
H	-2.987925000	1.313941000	1.677518000
H	-2.987925000	-1.313941000	1.677518000
H	-4.096813000	-2.177549000	0.603662000

$TS_{1f+2b\rightarrow 6'f}$

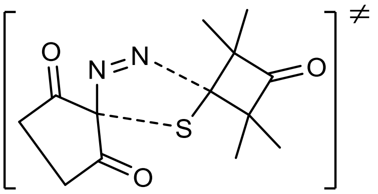

Imaginary Freq.: -384.65 cm^{-1}
Sum of electronic and zero-point Energies= -1236.888999
Sum of electronic and thermal Energies= -1236.869046
Sum of electronic and thermal Enthalpies= -1236.868102
Sum of electronic and thermal Free Energies= -1236.937807

Standard orientation. Coordinates (Angstroms):

S	-0.350334000	-0.098029000	1.272643000
C	-1.947254000	-0.046022000	-0.590847000
C	-2.626106000	1.235714000	-0.242363000
C	-2.808815000	-1.205679000	-0.230389000
C	0.990444000	-0.044975000	0.268438000
C	2.102251000	-1.121891000	0.115724000
C	1.986860000	1.134980000	0.090900000
C	3.033774000	0.053954000	-0.228477000
C	2.460927000	-1.778946000	1.453125000
H	2.583838000	-1.050288000	2.259508000
H	1.664595000	-2.471980000	1.744753000
H	3.396451000	-2.338518000	1.348745000
C	1.968015000	-2.196644000	-0.957249000
H	1.852995000	-1.778040000	-1.959701000
H	2.866082000	-2.822820000	-0.958317000
H	1.103384000	-2.835837000	-0.743347000
C	2.271485000	1.861221000	1.409543000
H	3.144054000	2.511930000	1.289879000
H	1.406482000	2.475185000	1.681962000
H	2.466638000	1.171299000	2.235534000
C	1.732707000	2.160439000	-1.010023000
H	0.780418000	2.674036000	-0.831786000
H	2.534117000	2.906162000	-1.007601000
H	1.702683000	1.708877000	-2.004773000
O	4.158863000	0.105367000	-0.647057000
N	-0.982331000	-0.101603000	-1.486751000
N	0.165753000	-0.105324000	-1.628393000
O	-2.726767000	-2.332110000	-0.649398000
O	-2.357408000	2.334685000	-0.656804000
C	-3.815850000	-0.667320000	0.780555000
C	-3.728806000	0.864315000	0.742924000
H	-4.655379000	1.339812000	0.405826000
H	-3.478256000	1.302406000	1.714465000
H	-3.556749000	-1.078476000	1.762414000
H	-4.805269000	-1.055213000	0.521080000

12. Thiodiazolines 6' obtained from diazo compounds 1 and aliphatic thioketone 2b

Gas phase, 6-31G(d), PBE1PBE

Thiadiazoline 6'a

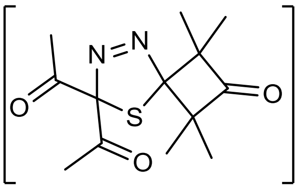

Sum of electronic and zero-point Energies:-1238.115742
Sum of electronic and thermal Energies: -1238.094489
Sum of electronic and thermal Enthalpies: -1238.093545
Sum of electronic and thermal Free Energies: -1238.165227

Standard orientation. Coordinates (Angstroms):

S	0.461915000	-0.336222000	1.191451000
C	1.686044000	-0.107036000	-0.145589000
C	2.858927000	-1.083578000	-0.011502000
O	3.252029000	-1.397839000	1.088993000
C	3.489151000	-1.567713000	-1.286419000
H	2.768343000	-2.171010000	-1.849806000
H	3.752340000	-0.727302000	-1.938358000
H	4.373180000	-2.161343000	-1.047848000
C	2.151352000	1.380586000	-0.064134000
O	1.612123000	2.213861000	-0.751616000
C	3.233719000	1.701683000	0.928218000
H	3.105922000	1.149222000	1.862753000
H	4.206055000	1.407035000	0.513413000
H	3.240087000	2.777771000	1.109346000
C	-0.870457000	-0.178672000	-0.052227000
C	-1.829601000	1.090814000	-0.066110000
C	-2.114770000	-1.135631000	0.117926000
C	-2.996442000	0.101716000	-0.066569000
C	-1.767416000	1.943247000	1.197210000
H	-1.805953000	1.349830000	2.115712000
H	-0.841103000	2.526820000	1.210373000
H	-2.617288000	2.633646000	1.201382000
C	-1.773485000	1.974020000	-1.310118000
H	-1.890349000	1.395057000	-2.229593000
H	-2.585733000	2.706437000	-1.259604000
H	-0.816580000	2.502421000	-1.358327000
C	-2.269642000	-1.722154000	1.520898000
H	-3.269068000	-2.159491000	1.613717000
H	-1.525726000	-2.506906000	1.692461000
H	-2.160650000	-0.971547000	2.311024000
C	-2.290985000	-2.220448000	-0.936781000
H	-1.535027000	-3.004065000	-0.810374000
H	-3.280215000	-2.675022000	-0.820453000
H	-2.208118000	-1.824014000	-1.951149000
O	-4.182161000	0.240571000	-0.203135000
N	0.984402000	-0.293195000	-1.418662000
N	-0.240840000	-0.332767000	-1.360002000

Thiadiazoline 6'b

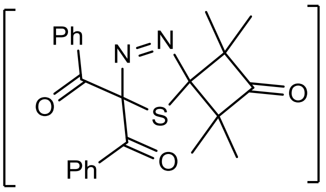

Sum of electronic and zero-point Energies= -1621.036322
Sum of electronic and thermal Energies= -1621.009279
Sum of electronic and thermal Enthalpies= -1621.008335
Sum of electronic and thermal Free Energies= -1621.094657

Standard orientation. Coordinates (Angstroms):

S	-1.045683000	-0.424781000	-1.171083000
C	0.267822000	0.276479000	-0.120127000
C	1.348545000	1.023539000	-0.940645000
O	1.306036000	0.985928000	-2.153970000
C	0.818468000	-0.866899000	0.774683000
O	0.477718000	-0.909079000	1.941263000
C	-2.304408000	0.292185000	-0.059757000
C	-3.206560000	-0.647695000	0.854036000
C	-3.594717000	0.904893000	-0.730476000
C	-4.410677000	0.113019000	0.294355000
C	-3.190852000	-2.117820000	0.446194000
H	-3.317417000	-2.264052000	-0.630640000
H	-2.241420000	-2.578569000	0.738011000
H	-4.007102000	-2.637257000	0.959079000
C	-3.025521000	-0.510306000	2.363148000
H	-3.112275000	0.527627000	2.693711000
H	-3.798645000	-1.098317000	2.868424000
H	-2.041144000	-0.882870000	2.664489000
C	-3.852770000	0.414586000	-2.154926000
H	-4.871900000	0.688809000	-2.446575000
H	-3.149788000	0.882828000	-2.851531000
H	-3.758302000	-0.671705000	-2.255871000
C	-3.749563000	2.418378000	-0.651760000
H	-3.031876000	2.910735000	-1.317842000
H	-4.760593000	2.691998000	-0.970628000
H	-3.590876000	2.794339000	0.361422000
O	-5.576566000	0.119912000	0.585518000
N	-0.387710000	1.260558000	0.777045000
N	-1.612674000	1.261607000	0.775675000
C	1.714541000	-1.903067000	0.192835000
C	1.975529000	-2.041133000	-1.176257000
C	2.310679000	-2.791385000	1.098522000
C	2.823635000	-3.048291000	-1.623737000
H	1.521123000	-1.376409000	-1.902909000
C	3.161035000	-3.790012000	0.647807000
H	2.090338000	-2.674718000	2.154846000
C	3.418995000	-3.920050000	-0.716240000
H	3.017205000	-3.151699000	-2.687487000
H	3.623582000	-4.469503000	1.358144000
H	4.083354000	-4.703195000	-1.071721000
C	2.430754000	1.745090000	-0.221097000
C	2.527763000	1.827375000	1.174695000
C	3.413193000	2.352815000	-1.016626000
C	3.594988000	2.503968000	1.755939000
H	1.767425000	1.387489000	1.809353000
C	4.471965000	3.029440000	-0.431359000
H	3.321273000	2.276404000	-2.095379000

C	4.564919000	3.104474000	0.958520000
H	3.663988000	2.565668000	2.838185000
H	5.227793000	3.498408000	-1.055084000
H	5.394803000	3.633741000	1.419433000

Thiadiazoline 6'c

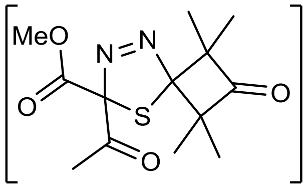

Sum of electronic and zero-point Energies:-1313.261907
Sum of electronic and thermal Energies: -1313.239769
Sum of electronic and thermal Enthalpies: -1313.238825
Sum of electronic and thermal Free Energies: -1313.313587

Standard orientation. Coordinates (Angstroms):

S	-0.338156000	-0.083965000	1.202917000
C	-1.521382000	-0.380378000	-0.167526000
C	-2.734515000	0.531128000	0.002869000
O	-3.773653000	0.212388000	0.528257000
C	-3.534608000	2.691359000	-0.336806000
H	-3.169822000	3.606960000	-0.800884000
H	-4.434869000	2.334368000	-0.842539000
H	-3.758028000	2.855898000	0.720317000
O	-2.474584000	1.743663000	-0.478301000
C	-1.806943000	-1.905892000	-0.195152000
O	-1.156565000	-2.595154000	-0.946798000
C	-2.810036000	-2.476223000	0.764981000
H	-2.714055000	-2.033192000	1.760221000
H	-3.822871000	-2.247163000	0.420292000
H	-2.665314000	-3.557388000	0.809000000
C	1.006130000	0.116837000	-0.029141000
C	2.195799000	-0.934524000	-0.082314000
C	2.031240000	1.294034000	0.198949000
C	3.141482000	0.268329000	-0.050528000
C	2.311935000	-1.815216000	1.158661000
H	2.246641000	-1.251144000	2.094197000
H	1.513859000	-2.564655000	1.160201000
H	3.278332000	-2.329508000	1.139505000
C	2.289868000	-1.783575000	-1.346736000
H	2.309004000	-1.171268000	-2.251416000
H	3.209629000	-2.376320000	-1.311492000
H	1.431575000	-2.460125000	-1.411498000
C	2.072414000	1.825141000	1.631394000
H	2.968988000	2.441260000	1.755344000
H	1.191637000	2.443052000	1.834287000
H	2.109586000	1.028918000	2.382253000
C	1.971353000	2.447287000	-0.794530000
H	1.057229000	3.032306000	-0.641664000
H	2.833682000	3.103136000	-0.636785000
H	1.984550000	2.097871000	-1.829275000
O	4.327429000	0.378711000	-0.207827000
N	-0.828926000	-0.030640000	-1.421880000
N	0.369399000	0.202601000	-1.332994000

Thiadiazoline 6'd

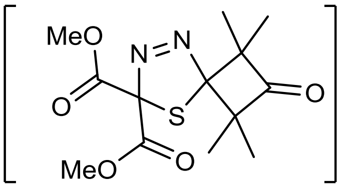

Sum of electronic and zero-point Energies:-1388.409543
Sum of electronic and thermal Energies: -1388.386321
Sum of electronic and thermal Enthalpies: -1388.385376
Sum of electronic and thermal Free Energies: -1388.462856

Standard orientation. Coordinates (Angstroms):

S	-0.226840000	-0.412413000	1.085517000
C	-1.389401000	-0.024083000	-0.261329000
C	-2.080156000	1.342115000	-0.127637000
O	-3.272983000	1.499774000	-0.167571000
C	-1.741552000	3.635555000	0.077483000
H	-0.889339000	4.310936000	0.146385000
H	-2.336501000	3.852132000	-0.812896000
H	-2.375363000	3.722930000	0.963276000
O	-1.184503000	2.321165000	-0.005234000
C	-2.451374000	-1.119507000	-0.384652000
O	-2.650450000	-1.769677000	-1.375904000
O	-3.090220000	-1.261130000	0.775577000
C	-4.118698000	-2.251829000	0.778742000
H	-4.527694000	-2.244839000	1.788514000
H	-4.890419000	-1.996230000	0.048678000
H	-3.703986000	-3.233472000	0.536335000
C	1.178014000	-0.215109000	-0.070477000
C	2.309138000	-1.319127000	-0.048413000
C	2.259811000	0.919916000	0.180976000
C	3.319369000	-0.170291000	0.000570000
C	2.343542000	-2.157049000	1.229132000
H	2.276681000	-1.553251000	2.140089000
H	1.515442000	-2.872869000	1.235791000
H	3.287316000	-2.710944000	1.264416000
C	2.406100000	-2.217149000	-1.275572000
H	2.428206000	-1.642785000	-2.204312000
H	3.322309000	-2.813210000	-1.213131000
H	1.550550000	-2.900839000	-1.316116000
C	2.239292000	1.496188000	1.593828000
H	3.138791000	2.101933000	1.745601000
H	1.356221000	2.129790000	1.724739000
H	2.216562000	0.725615000	2.370496000
C	2.332394000	2.040373000	-0.850700000
H	1.447288000	2.681004000	-0.778198000
H	3.222698000	2.646985000	-0.655673000
H	2.393904000	1.656022000	-1.871510000
O	4.515001000	-0.131706000	-0.111413000
N	-0.605786000	0.027773000	-1.522495000
N	0.609089000	-0.068878000	-1.399803000

Thiadiazoline 6'e

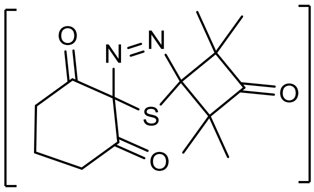

Sum of electronic and zero-point Energies= -1276.174503
Sum of electronic and thermal Energies= -1276.154380
Sum of electronic and thermal Enthalpies= -1276.153436
Sum of electronic and thermal Free Energies= -1276.222106

Standard orientation. Coordinates (Angstroms):

S	0.093305000	-0.601285000	1.281564000
C	1.471018000	-0.206549000	0.200003000
C	2.605382000	-1.240809000	0.251808000
C	2.076394000	1.226007000	0.402850000
C	-1.068605000	-0.165602000	-0.058310000
C	-1.969028000	1.143994000	0.040406000
C	-2.361206000	-1.058056000	-0.214991000
C	-3.155436000	0.246976000	-0.316079000
C	-2.061119000	1.718809000	1.451539000
H	-2.310820000	0.965219000	2.204317000
H	-1.101820000	2.167339000	1.726013000
H	-2.840263000	2.488122000	1.471471000
C	-1.691820000	2.254858000	-0.966334000
H	-1.632419000	1.878583000	-1.990749000
H	-2.504001000	2.987395000	-0.916798000
H	-0.752173000	2.761264000	-0.722365000
C	-2.725163000	-1.854137000	1.038023000
H	-3.743113000	-2.242457000	0.929629000
H	-2.038844000	-2.697947000	1.163017000
H	-2.693092000	-1.252264000	1.951826000
C	-2.432522000	-1.950533000	-1.447835000
H	-1.730521000	-2.787085000	-1.355323000
H	-3.444817000	-2.358295000	-1.535795000
H	-2.198474000	-1.404414000	-2.364419000
O	-4.292948000	0.483735000	-0.623403000
N	0.937996000	-0.154185000	-1.198437000
N	-0.286767000	-0.136020000	-1.284630000
O	1.401390000	2.122917000	0.838083000
O	2.615031000	-2.126314000	1.071018000
C	3.904010000	0.443697000	-1.152244000
C	3.520715000	1.384178000	-0.010647000
C	3.719138000	-1.026302000	-0.751238000
H	4.946173000	0.619009000	-1.436471000
H	3.288661000	0.673548000	-2.027103000
H	3.484551000	-1.636633000	-1.633077000
H	4.624166000	-1.450062000	-0.303680000
H	4.127718000	1.161425000	0.881821000
H	3.689279000	2.437364000	-0.253170000

Thiadiazoline 6'f

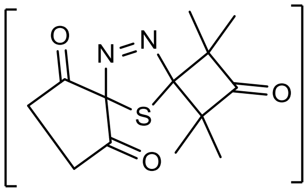

Sum of electronic and zero-point Energies= -1236.937373
Sum of electronic and thermal Energies= -1236.918232
Sum of electronic and thermal Enthalpies= -1236.917288
Sum of electronic and thermal Free Energies= -1236.984430

Standard orientation. Coordinates (Angstroms):

S	0.355489000	-0.501742000	1.234643000
C	1.654733000	-0.165319000	0.059480000
C	2.834266000	-1.157063000	0.012992000
C	2.356436000	1.213660000	0.148508000
C	-0.895018000	-0.158491000	-0.061432000
C	-1.798178000	1.149220000	0.027204000
C	-2.183104000	-1.066459000	-0.091048000
C	-2.997158000	0.226547000	-0.202072000
C	-1.801338000	1.803564000	1.406096000
H	-1.982350000	1.092520000	2.217733000
H	-0.834493000	2.284187000	1.585377000
H	-2.591152000	2.561419000	1.436979000
C	-1.587191000	2.203204000	-1.054386000
H	-1.610248000	1.774997000	-2.059591000
H	-2.382836000	2.951580000	-0.981200000
H	-0.623833000	2.703752000	-0.908694000
C	-2.457509000	-1.802209000	1.220292000
H	-3.476628000	-2.201545000	1.197883000
H	-1.756715000	-2.634718000	1.341111000
H	-2.372505000	-1.155032000	2.099342000
C	-2.319276000	-2.018343000	-1.272747000
H	-1.601730000	-2.841814000	-1.184062000
H	-3.329866000	-2.439431000	-1.278342000
H	-2.148356000	-1.514578000	-2.226894000
O	-4.156136000	0.438652000	-0.437608000
N	1.045460000	-0.170669000	-1.312062000
N	-0.181379000	-0.176270000	-1.324235000
O	1.805188000	2.239876000	0.446218000
O	2.799906000	-2.296831000	0.387805000
C	3.824870000	1.051576000	-0.205261000
C	4.025690000	-0.421386000	-0.572255000
H	3.982204000	-0.561674000	-1.660334000
H	4.958847000	-0.862143000	-0.214562000
H	4.410760000	1.342510000	0.675541000
H	4.087508000	1.751838000	-1.004045000

13. Transition states for decompositions of thiadiazolines 6'

Gas phase, 6-31G(d), PBE1PBE

<u>***$TS_{6'a \rightarrow N \equiv N + 7'a}$***</u>

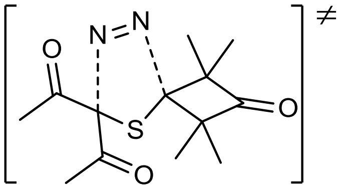

Imaginary Freq.: -349.67 cm^{-1}
Sum of electronic and zero-point Energies:-1238.075254
Sum of electronic and thermal Energies: -1238.053837
Sum of electronic and thermal Enthalpies: -1238.052892
Sum of electronic and thermal Free Energies: -1238.123840

Standard orientation. Coordinates (Angstroms):

S	-0.420529000	-0.912346000	-0.869430000
C	-1.845154000	-0.118897000	-0.270216000
C	-2.965461000	-1.099196000	-0.215136000
O	-2.907872000	-2.126447000	-0.871879000
C	-4.137928000	-0.866897000	0.712191000
H	-3.826046000	-0.462120000	1.679200000
H	-4.863624000	-0.175764000	0.271498000
H	-4.630973000	-1.830950000	0.853289000
C	-1.980982000	1.342004000	-0.351037000
O	-1.115576000	2.025203000	-0.885433000
C	-3.183335000	2.028801000	0.256856000
H	-4.089443000	1.819672000	-0.321958000
H	-3.362049000	1.713739000	1.288710000
H	-2.994932000	3.103543000	0.228996000
C	0.900704000	-0.214664000	0.004937000
C	1.743853000	1.093313000	-0.154359000
C	2.178927000	-1.093581000	0.119733000
C	2.962085000	0.222373000	0.173028000
C	1.814287000	1.556362000	-1.618429000
H	2.015166000	0.733908000	-2.310632000
H	0.865410000	2.018517000	-1.892805000
H	2.627318000	2.283910000	-1.711000000
C	1.531340000	2.281049000	0.772107000
H	1.526561000	1.987133000	1.825381000
H	2.359859000	2.982140000	0.628522000
H	0.593921000	2.787221000	0.527845000
C	2.499695000	-1.945386000	-1.114105000
H	3.527388000	-2.312715000	-1.028722000
H	1.827874000	-2.808830000	-1.165236000
H	2.413521000	-1.387943000	-2.050468000
C	2.294359000	-1.953897000	1.377092000
H	1.535371000	-2.743988000	1.364613000
H	3.283330000	-2.422261000	1.397918000
H	2.174541000	-1.368439000	2.291326000
O	4.106376000	0.480947000	0.428456000
N	-0.977210000	0.075905000	1.814583000
N	0.149713000	-0.039498000	1.686742000

TS $_{6'b \to N \equiv N+7'b}$

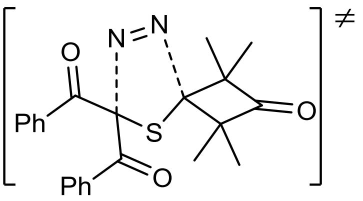

Imaginary Freq.: -355.89 cm^{-1}
Sum of electronic and zero-point Energies= -1620.994246
Sum of electronic and thermal Energies= -1620.966672
Sum of electronic and thermal Enthalpies= -1620.965727
Sum of electronic and thermal Free Energies= -1621.053531

Standard orientation. Coordinates (Angstroms):

S	1.205245000	-0.506789000	-1.213044000
C	-0.262779000	-0.419378000	-0.310197000
C	-1.302682000	-1.285878000	-0.917585000
O	-1.147749000	-1.782591000	-2.029448000
C	-0.630554000	0.819999000	0.450595000
O	-0.009030000	1.170895000	1.444101000
C	2.481916000	-0.328422000	-0.069163000
C	3.061006000	0.907941000	0.700992000
C	3.887852000	-0.752304000	-0.575127000
C	4.432173000	0.460841000	0.179150000
C	2.655800000	2.265352000	0.122191000
H	2.635830000	2.256931000	-0.972396000
H	1.668984000	2.554808000	0.490548000
H	3.389598000	3.011983000	0.444107000
C	3.009567000	0.923809000	2.227862000
H	3.366901000	-0.015168000	2.660430000
H	3.665558000	1.726913000	2.579543000
H	1.990729000	1.107699000	2.572113000
C	4.131248000	-0.679890000	-2.083497000
H	5.205694000	-0.775823000	-2.269863000
H	3.615345000	-1.494989000	-2.601938000
H	3.795449000	0.269958000	-2.511430000
C	4.378314000	-2.093171000	-0.022431000
H	3.804649000	-2.914046000	-0.466213000
H	5.433656000	-2.220230000	-0.283625000
H	4.280036000	-2.154389000	1.063896000
O	5.536275000	0.915550000	0.306929000
N	0.674122000	-1.522781000	1.407377000
N	1.805398000	-1.435366000	1.300854000
C	-1.754972000	1.666831000	-0.049315000
C	-2.226083000	1.604960000	-1.365449000
C	-2.303091000	2.606177000	0.833218000
C	-3.236224000	2.463804000	-1.786054000
H	-1.796963000	0.898836000	-2.069980000
C	-3.320516000	3.451513000	0.415331000
H	-1.913737000	2.652218000	1.845551000
C	-3.788545000	3.381529000	-0.896799000
H	-3.591722000	2.415569000	-2.811405000
H	-3.750002000	4.168672000	1.109447000
H	-4.583023000	4.045922000	-1.226220000
C	-2.531740000	-1.586161000	-0.124263000
C	-2.622270000	-1.397507000	1.259101000
C	-3.625744000	-2.116664000	-0.818163000
C	-3.796680000	-1.721488000	1.931349000
H	-1.769508000	-1.029402000	1.820125000
C	-4.798174000	-2.431488000	-0.146229000

H	-3.527910000	-2.274578000	-1.887778000
C	-4.886172000	-2.232000000	1.231295000
H	-3.857245000	-1.580613000	3.006958000
H	-5.645682000	-2.835140000	-0.693602000
H	-5.803058000	-2.480737000	1.759294000

***TS**$_{6'c \rightarrow N \equiv N+7'c}$*

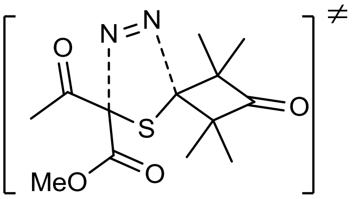

Imaginary Freq.: -352.68 cm^{-1}
Sum of electronic and zero-point Energies:-1313.227218
Sum of electronic and thermal Energies: -1313.204769
Sum of electronic and thermal Enthalpies: -1313.203825
Sum of electronic and thermal Free Energies: -1313.277506

Standard orientation. Coordinates (Angstroms):

S	-0.085198000	-1.114143000	-0.852397000
C	-1.607280000	-0.544680000	-0.261113000
C	-2.585908000	-1.667729000	-0.184681000
O	-2.328531000	-2.731458000	-0.725156000
C	-3.856783000	-1.503157000	0.611985000
H	-3.679192000	-0.988081000	1.559468000
H	-4.583642000	-0.906416000	0.052637000
H	-4.265503000	-2.501075000	0.783681000
C	-1.975434000	0.870348000	-0.369051000
O	-1.280530000	1.740961000	-0.860550000
C	-3.572264000	2.506609000	0.107790000
H	-3.592196000	2.860574000	-0.926018000
H	-4.570446000	2.541725000	0.544887000
H	-2.883173000	3.131146000	0.682614000
C	1.119396000	-0.222441000	0.011844000
C	1.738513000	1.205231000	-0.145019000
C	2.522298000	-0.885362000	0.102594000
C	3.086277000	0.538286000	0.153844000
C	1.708791000	1.685755000	-1.604689000
H	2.023492000	0.910349000	-2.308856000
H	0.694482000	1.996317000	-1.858707000
H	2.395770000	2.532704000	-1.703449000
C	1.354474000	2.334653000	0.799006000
H	1.417322000	2.034008000	1.848406000
H	2.055197000	3.162268000	0.648554000
H	0.342579000	2.683354000	0.577885000
C	2.960440000	-1.671966000	-1.138210000
H	4.033925000	-1.873456000	-1.064687000
H	2.432344000	-2.630200000	-1.187527000
H	2.777970000	-1.131079000	-2.070570000
C	2.786197000	-1.718646000	1.356207000
H	2.163961000	-2.620337000	1.347035000
H	3.837934000	-2.021296000	1.367268000
H	2.580389000	-1.162161000	2.273193000
O	4.179110000	0.975898000	0.389573000
N	-0.781662000	-0.221679000	1.821125000
N	0.350870000	-0.169878000	1.703151000
O	-3.179400000	1.138030000	0.166079000

$TS_{6'd \to N \equiv N + 7'd}$

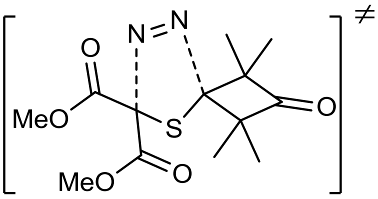

Imaginary Freq.: -350.80 cm^{-1}
Sum of electronic and zero-point Energies:-1388.372669
Sum of electronic and thermal Energies: -1388.349098
Sum of electronic and thermal Enthalpies: -1388.348154
Sum of electronic and thermal Free Energies: -1388.425206

Standard orientation. Coordinates (Angstroms):

S	0.015585000	-0.929244000	-0.920130000
C	-1.427418000	-0.204820000	-0.307987000
C	-2.526643000	-1.203750000	-0.307112000
O	-2.473656000	-2.256653000	-0.911204000
C	-4.615811000	-1.805973000	0.506752000
H	-5.023449000	-1.970265000	-0.494305000
H	-4.263941000	-2.761694000	0.904419000
H	-5.371402000	-1.373057000	1.162559000
C	-1.627024000	1.249050000	-0.361863000
O	-0.771724000	2.042199000	-0.715977000
C	-3.051607000	3.046241000	0.028515000
H	-2.910178000	3.452327000	-0.976639000
H	-4.080929000	3.188260000	0.358212000
H	-2.360082000	3.546936000	0.711806000
C	1.319612000	-0.267735000	0.001871000
C	2.125314000	1.070353000	-0.047328000
C	2.617902000	-1.119027000	0.047211000
C	3.367902000	0.208082000	0.203374000
C	2.180837000	1.655932000	-1.467797000
H	2.388043000	0.896801000	-2.227527000
H	1.224371000	2.127134000	-1.697316000
H	2.983801000	2.399611000	-1.503260000
C	1.882973000	2.169193000	0.976439000
H	1.902799000	1.788483000	2.001191000
H	2.682852000	2.910744000	0.881955000
H	0.923282000	2.655524000	0.785187000
C	2.964622000	-1.869476000	-1.243782000
H	3.999719000	-2.219861000	-1.178732000
H	2.312806000	-2.741351000	-1.364800000
H	2.871549000	-1.244406000	-2.135937000
C	2.747808000	-2.064734000	1.240978000
H	2.012096000	-2.872781000	1.162994000
H	3.750185000	-2.504281000	1.239208000
H	2.600418000	-1.550224000	2.193013000
O	4.507724000	0.475040000	0.469753000
N	-0.605555000	-0.119885000	1.793253000
N	0.525201000	-0.221535000	1.700925000
O	-2.841783000	1.637986000	0.040382000
O	-3.555514000	-0.854830000	0.470194000

TS$_{6'e \rightarrow N\equiv N+7'e}$

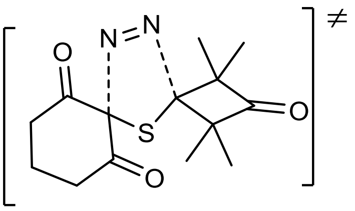

Imaginary Freq.: -350.44 cm^{-1}
Sum of electronic and zero-point Energies= 1276.149027
Sum of electronic and thermal Energies= -1276.128489
Sum of electronic and thermal Enthalpies= -1276.127545
Sum of electronic and thermal Free Energies= -1276.196570

Standard orientation. Coordinates (Angstroms):

S	-0.155098000	-1.019133000	-0.865997000
C	-1.638867000	-0.280149000	-0.380455000
C	-2.744327000	-1.257567000	-0.284426000
C	-1.897274000	1.154769000	-0.457521000
C	1.100139000	-0.213364000	0.026207000
C	1.861194000	1.142380000	-0.183101000
C	2.435744000	-1.002105000	0.169979000
C	3.134601000	0.361130000	0.159292000
C	1.875839000	1.567387000	-1.658514000
H	2.119192000	0.740737000	-2.332087000
H	0.891771000	1.957610000	-1.922622000
H	2.636027000	2.344890000	-1.786367000
C	1.580970000	2.335586000	0.718272000
H	1.624019000	2.071030000	1.778813000
H	2.351439000	3.091654000	0.536436000
H	0.602511000	2.762176000	0.483653000
C	2.805629000	-1.879090000	-1.031615000
H	3.855536000	-2.173875000	-0.936866000
H	2.192145000	-2.786131000	-1.046064000
H	2.681574000	-1.364570000	-1.988210000
C	2.609783000	-1.800425000	1.460580000
H	1.903644000	-2.637750000	1.485703000
H	3.627042000	-2.202439000	1.495120000
H	2.455892000	-1.186188000	2.350874000
O	4.264109000	0.699379000	0.384650000
N	-0.776382000	0.050725000	1.793476000
N	0.347261000	-0.035516000	1.634953000
O	-1.080146000	1.964770000	-0.880038000
O	-2.597745000	-2.438865000	-0.553170000
C	-4.009714000	0.618419000	0.874957000
C	-3.257196000	1.631295000	0.023318000
C	-4.094010000	-0.717825000	0.151599000
H	-5.015057000	0.990580000	1.100875000
H	-3.495955000	0.487233000	1.834397000
H	-4.570886000	-1.495948000	0.755938000
H	-4.702352000	-0.617386000	-0.760040000
H	-3.837247000	1.872012000	-0.880302000
H	-3.098700000	2.581100000	0.544737000

$TS_{6'f \rightarrow N\equiv N+7'f}$

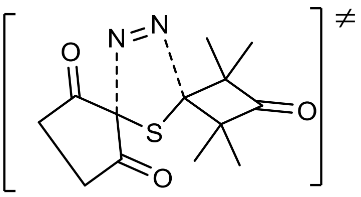

Imaginary Freq.: -355.22 cm^{-1}
Sum of electronic and zero-point Energies= -1236.913936
Sum of electronic and thermal Energies= -1236.894331
Sum of electronic and thermal Enthalpies= -1236.893387
Sum of electronic and thermal Free Energies= -1236.960910

Standard orientation. Coordinates (Angstroms):

S	-0.360748000	-0.995276000	-0.879239000
C	-1.790696000	-0.236423000	-0.329011000
C	-2.985184000	-1.074794000	-0.144231000
C	-2.151625000	1.173667000	-0.290169000
C	0.922107000	-0.246970000	0.027644000
C	1.629851000	1.144013000	-0.129334000
C	2.280711000	-1.001372000	0.057528000
C	2.944165000	0.380435000	0.071021000
C	1.516079000	1.685370000	-1.560513000
H	1.721246000	0.921782000	-2.316607000
H	0.504863000	2.070973000	-1.709783000
H	2.244487000	2.493193000	-1.684794000
C	1.376944000	2.251948000	0.883717000
H	1.509791000	1.909515000	1.914057000
H	2.103056000	3.051229000	0.705102000
H	0.368850000	2.654874000	0.754449000
C	2.604150000	-1.808026000	-1.204660000
H	3.663259000	-2.083161000	-1.180148000
H	2.009615000	-2.727329000	-1.236123000
H	2.420160000	-1.245897000	-2.124501000
C	2.546856000	-1.850106000	1.299615000
H	1.875952000	-2.715973000	1.318312000
H	3.579656000	-2.210409000	1.267806000
H	2.410808000	-1.283822000	2.223973000
O	4.079839000	0.736833000	0.223091000
N	-0.912749000	-0.074429000	1.839251000
N	0.206562000	-0.179880000	1.662625000
O	-1.467503000	2.141222000	-0.585915000
O	-3.069779000	-2.276749000	-0.297452000
C	-3.586889000	1.269812000	0.219681000
C	-4.123233000	-0.160875000	0.300643000
H	-4.422885000	-0.451688000	1.313237000
H	-4.987394000	-0.338445000	-0.347863000
H	-4.153076000	1.912038000	-0.462615000
H	-3.572985000	1.777179000	1.190801000

14. Thiodiazolines 6' decomposition products

Gas phase, 6-31G(d), PBE1PBE

Nitrogen (N≡N)

Sum of electronic and zero-point Energies: -109.394592
Sum of electronic and thermal Energies: -109.392232
Sum of electronic and thermal Enthalpies: -109.391288
Sum of electronic and thermal Free Energies: -109.413037

Standard orientation. Coordinates (Angstroms):

N	0.000000000	0.000000000	0.551300000
N	0.000000000	0.000000000	-0.551300000

C=S-ylide 7'a

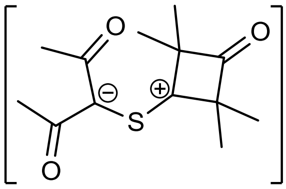

Sum of electronic and zero-point Energies:-1128.723071
Sum of electronic and thermal Energies: -1128.702707
Sum of electronic and thermal Enthalpies: -1128.701763
Sum of electronic and thermal Free Energies: -1128.772659

Standard orientation. Coordinates (Angstroms):

S	-0.418096000	-1.057951000	-0.536825000
C	-1.864912000	-0.169316000	-0.158028000
C	-2.801660000	-1.124724000	0.389091000
O	-2.396057000	-2.258799000	0.677077000
C	-4.262017000	-0.802471000	0.615768000
H	-4.388680000	0.022402000	1.324166000
H	-4.769459000	-0.523096000	-0.312631000
H	-4.732079000	-1.698109000	1.025764000
C	-1.991859000	1.178380000	-0.664370000
O	-1.054117000	1.766097000	-1.208788000
C	-3.320646000	1.903863000	-0.547712000
H	-4.099133000	1.422537000	-1.148381000
H	-3.678174000	1.947448000	0.485474000
H	-3.167565000	2.918497000	-0.919839000
C	0.934947000	-0.218430000	-0.202421000
C	1.390579000	0.910971000	0.712313000
C	2.341503000	-0.736403000	-0.494122000
C	2.783774000	0.260741000	0.592988000
C	1.429043000	2.337930000	0.146364000
H	1.803597000	2.367332000	-0.879573000
H	0.432212000	2.777924000	0.141192000
H	2.100429000	2.925433000	0.781510000
C	0.769842000	0.889245000	2.107313000
H	0.782709000	-0.113679000	2.544921000
H	1.337617000	1.559167000	2.760804000
H	-0.267555000	1.233572000	2.064312000
C	2.859735000	-0.375936000	-1.892765000
H	3.942937000	-0.531207000	-1.927504000
H	2.386340000	-1.022315000	-2.639294000
H	2.649572000	0.662915000	-2.160691000
C	2.615692000	-2.201224000	-0.165067000
H	2.145989000	-2.858901000	-0.904290000
H	3.695846000	-2.377825000	-0.180150000
H	2.236512000	-2.471049000	0.825170000
O	3.821961000	0.439005000	1.165726000

C=S-ylide 7'b

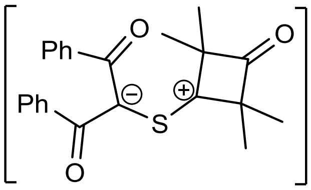

Sum of electronic and zero-point Energies= -1511.636769
Sum of electronic and thermal Energies= -1511.610523
Sum of electronic and thermal Enthalpies= -1511.609579
Sum of electronic and thermal Free Energies= -1511.694898

Standard orientation. Coordinates (Angstroms):

C	1.650778000	-1.082847000	-0.253350000
S	0.445704000	-0.632049000	-1.242459000
C	-0.842330000	0.020345000	-0.236454000
C	-1.988998000	-0.765227000	-0.601465000
C	-0.630435000	1.230820000	0.535640000
O	-1.278334000	1.522643000	1.543035000
O	-1.755052000	-1.717709000	-1.385027000
C	-3.408496000	-0.501866000	-0.230806000
C	-3.816284000	0.170059000	0.925793000
C	-4.379096000	-1.025596000	-1.097143000
C	-5.173441000	0.325209000	1.196313000
H	-3.069523000	0.567220000	1.603714000
C	-5.729511000	-0.855036000	-0.829378000
H	-4.046185000	-1.567002000	-1.976707000
C	-6.131083000	-0.178187000	0.321869000
H	-5.481012000	0.844111000	2.100408000
H	-6.471959000	-1.255064000	-1.514961000
H	-7.188877000	-0.049279000	0.537289000
C	0.426240000	2.209272000	0.089702000
C	0.667162000	2.499866000	-1.256784000
C	1.110604000	2.933922000	1.070830000
C	1.603463000	3.466284000	-1.615202000
H	0.091422000	1.996522000	-2.028951000
C	2.060171000	3.883877000	0.714946000
H	0.875197000	2.742948000	2.113746000
C	2.312621000	4.148731000	-0.630869000
H	1.769693000	3.694000000	-2.664702000
H	2.596540000	4.429698000	1.486690000
H	3.048074000	4.898618000	-0.909923000
C	1.787425000	-1.451811000	1.220166000
C	3.049244000	-1.540163000	-0.643566000
C	1.844358000	-0.332434000	2.259393000
H	2.469943000	0.503395000	1.933973000
H	0.840184000	0.045912000	2.470532000
H	2.268817000	-0.742155000	3.182052000
C	0.807529000	-2.550654000	1.647462000
H	-0.199537000	-2.133545000	1.738211000
H	0.775495000	-3.376110000	0.929991000
H	1.120803000	-2.946680000	2.618773000
C	3.987193000	-0.372401000	-0.977375000
H	5.022464000	-0.728477000	-0.974486000
H	3.749783000	0.024368000	-1.970027000
H	3.899804000	0.447109000	-0.257191000
C	3.178260000	-1.989327000	0.825764000
C	3.146454000	-2.656842000	-1.679320000
H	2.907544000	-2.276968000	-2.678534000
H	4.167783000	-3.050463000	-1.692741000
H	2.462801000	-3.480671000	-1.453022000
O	4.041950000	-2.553766000	1.435519000

C=S-ylide 7'c

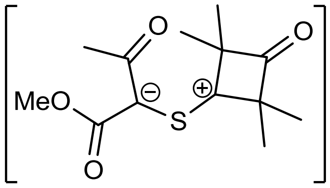

Sum of electronic and zero-point Energies:-1203.873653
Sum of electronic and thermal Energies: -1203.852580
Sum of electronic and thermal Enthalpies: -1203.851636
Sum of electronic and thermal Free Energies: -1203.924193

Standard orientation. Coordinates (Angstroms):

S	0.129693000	-1.201695000	0.125852000
C	1.713423000	-0.566059000	0.010425000
C	2.648170000	-1.692248000	-0.128099000
O	2.217106000	-2.837707000	-0.237814000
C	4.141237000	-1.462245000	-0.129171000
H	4.469426000	-0.941025000	0.774291000
H	4.617130000	-2.442512000	-0.194384000
H	4.444160000	-0.842356000	-0.977847000
C	1.995919000	0.843857000	0.119899000
O	1.159571000	1.718890000	0.289599000
O	3.309144000	1.140958000	0.019341000
C	3.617592000	2.524562000	0.138802000
H	4.702032000	2.590340000	0.043275000
H	3.130061000	3.102338000	-0.651300000
H	3.295769000	2.914582000	1.108242000
C	-1.177569000	-0.228631000	0.075859000
C	-1.724903000	1.177540000	-0.165942000
C	-2.566188000	-0.890954000	0.152223000
C	-3.103730000	0.497987000	-0.219501000
C	-1.673900000	2.137978000	1.032416000
H	-1.963642000	1.641824000	1.964418000
H	-0.665648000	2.535015000	1.144219000
H	-2.383148000	2.952002000	0.847690000
C	-1.324431000	1.866704000	-1.468187000
H	-1.336974000	1.170315000	-2.313340000
H	-2.043705000	2.666157000	-1.676284000
H	-0.323185000	2.289685000	-1.370017000
C	-2.984101000	-1.360956000	1.548148000
H	-4.059376000	-1.567218000	1.558167000
H	-2.448878000	-2.280335000	1.809591000
H	-2.770629000	-0.611108000	2.315738000
C	-2.859652000	-1.958227000	-0.901490000
H	-2.328551000	-2.887127000	-0.667426000
H	-3.934623000	-2.164792000	-0.919203000
H	-2.556449000	-1.632209000	-1.901326000
O	-4.202079000	0.902572000	-0.483678000

C=S-ylide 7'd

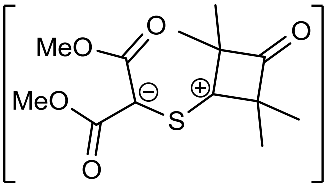

Sum of electronic and zero-point Energies:-1279.018540
Sum of electronic and thermal Energies: -1278.996309
Sum of electronic and thermal Enthalpies: -1278.995365
Sum of electronic and thermal Free Energies: -1279.071049

Standard orientation. Coordinates (Angstroms):

S	0.028284000	-1.062296000	0.097500000
C	1.513166000	-0.230301000	0.024453000
C	2.582991000	-1.232181000	-0.046472000
O	2.372273000	-2.433480000	-0.107015000
C	4.857025000	-1.693846000	-0.098476000
H	5.787998000	-1.126022000	-0.088447000
H	4.812232000	-2.371308000	0.758688000
H	4.778009000	-2.284523000	-1.015239000
O	3.820864000	-0.720583000	-0.030651000
C	1.614590000	1.210286000	0.093950000
O	0.654166000	1.953843000	0.240690000
O	2.864190000	1.685532000	-0.018732000
C	2.968236000	3.100942000	0.063317000
H	4.032392000	3.318666000	-0.033143000
H	2.406536000	3.582582000	-0.742311000
H	2.589315000	3.467297000	1.021594000
C	-1.398748000	-0.277430000	0.053841000
C	-2.140172000	1.049628000	-0.105326000
C	-2.679179000	-1.133763000	0.086787000
C	-3.413639000	0.189745000	-0.167904000
C	-2.175642000	1.958701000	1.130913000
H	-2.361481000	1.389285000	2.047712000
H	-1.226064000	2.484246000	1.229174000
H	-2.993408000	2.676815000	1.006297000
C	-1.885028000	1.837700000	-1.389589000
H	-1.835654000	1.179092000	-2.263356000
H	-2.713266000	2.538830000	-1.539451000
H	-0.946308000	2.387633000	-1.305408000
C	-3.006041000	-1.761690000	1.444040000
H	-4.041606000	-2.117440000	1.443547000
H	-2.343450000	-2.613175000	1.634237000
H	-2.888547000	-1.047040000	2.264436000
C	-2.838922000	-2.146546000	-1.046522000
H	-2.183574000	-3.009560000	-0.886319000
H	-3.875878000	-2.496396000	-1.078517000
H	-2.595129000	-1.706551000	-2.018630000
O	-4.567303000	0.454293000	-0.365909000

C=S-ylide 7'e

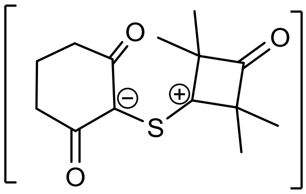

Sum of electronic and zero-point Energies= 1166.797856
Sum of electronic and thermal Energies= -1166.778660
Sum of electronic and thermal Enthalpies= -1166.777716
Sum of electronic and thermal Free Energies= -1166.845554

Standard orientation. Coordinates (Angstroms):

S	-0.196981000	-1.077852000	-0.102845000
C	-1.713244000	-0.282072000	-0.076137000
C	-2.782188000	-1.274399000	0.022000000
O	-2.563986000	-2.477307000	0.118228000
C	-4.203934000	-0.745584000	-0.022841000
C	-1.929268000	1.133231000	-0.219564000
O	-1.021905000	1.954370000	-0.347241000
C	-3.375665000	1.600683000	-0.242126000
C	1.190922000	-0.221852000	-0.057989000
C	1.841889000	1.149818000	0.103197000
C	2.522239000	-0.993231000	-0.063447000
C	3.166356000	0.374691000	0.205774000
C	1.848502000	2.038003000	-1.150134000
H	2.107573000	1.470435000	-2.050161000
H	0.863263000	2.484645000	-1.282931000
H	2.604434000	2.818477000	-1.012260000
C	1.492154000	1.940726000	1.362665000
H	1.458923000	1.296577000	2.247907000
H	2.266308000	2.698798000	1.523054000
H	0.522079000	2.424107000	1.235216000
C	2.910941000	-1.604459000	-1.412024000
H	3.965674000	-1.897290000	-1.389154000
H	2.304184000	-2.494717000	-1.610463000
H	2.767715000	-0.901371000	-2.238148000
C	2.717990000	-1.992595000	1.076452000
H	2.115759000	-2.891809000	0.907563000
H	3.772609000	-2.281876000	1.127108000
H	2.433229000	-1.564767000	2.042808000
O	4.294492000	0.711757000	0.436026000
C	-4.325680000	0.676459000	0.506144000
H	-3.385336000	2.620649000	0.154429000
H	-3.676538000	1.672028000	-1.298234000
H	-4.086846000	0.691919000	1.577628000
H	-5.357236000	1.032563000	0.407804000
H	-4.827342000	-1.456158000	0.528115000
H	-4.531652000	-0.783645000	-1.072698000

C=S-ylide 7'f

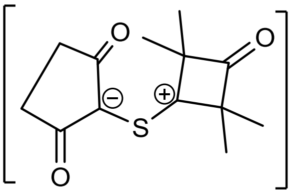

Sum of electronic and zero-point Energies= 1127.563354
Sum of electronic and thermal Energies= -1127.545064
Sum of electronic and thermal Enthalpies= -1127.544120
Sum of electronic and thermal Free Energies= -1127.610007

Standard orientation. Coordinates (Angstroms):

S	0.415252000	-1.098345000	0.000519000
C	1.874811000	-0.241504000	-0.000039000
C	3.058192000	-1.091901000	-0.000542000
O	3.095117000	-2.310969000	-0.000990000
C	4.279615000	-0.176680000	-0.000289000
C	2.214748000	1.156365000	0.000739000
O	1.481832000	2.138400000	0.001632000
C	3.741213000	1.253834000	0.000268000
C	-0.988172000	-0.270177000	0.000184000
C	-1.590419000	1.128404000	-0.000761000
C	-2.335460000	-1.001539000	0.000661000
C	-2.950936000	0.409505000	-0.000157000
C	-1.360046000	1.954361000	1.268928000
H	-1.510197000	1.357075000	2.174697000
H	-0.342340000	2.349829000	1.260172000
H	-2.079370000	2.780030000	1.282284000
C	-1.358706000	1.954569000	-1.269869000
H	-1.507894000	1.357482000	-2.175920000
H	-2.077984000	2.780267000	-1.283842000
H	-0.341060000	2.350149000	-1.259989000
C	-2.645377000	-1.805885000	1.264176000
H	-3.708721000	-2.065988000	1.277714000
H	-2.058778000	-2.730945000	1.280767000
H	-2.419345000	-1.239281000	2.172849000
C	-2.644849000	-1.804253000	-1.264368000
H	-2.056743000	-2.728322000	-1.282665000
H	-3.707813000	-2.065874000	-1.277884000
H	-2.420014000	-1.235782000	-2.172171000
O	-4.084061000	0.801430000	-0.000347000
H	4.047236000	1.833072000	-0.877613000
H	4.047926000	1.832740000	0.878105000
H	4.890144000	-0.410903000	-0.878853000
H	4.890148000	-0.411514000	0.878108000

15. Transition states for 1,5-electrocyclizations of C=S-ylides 7' to oxathioles 3'

Gas phase, 6-31G(d), PBE1PBE

***TS**$_{7'a \rightarrow 3'a}$*

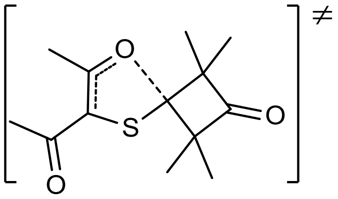

Imaginary Freq.: -93.54 cm^{-1}
Sum of electronic and zero-point Energies:-1128.720845
Sum of electronic and thermal Energies: -1128.701523
Sum of electronic and thermal Enthalpies: -1128.700579
Sum of electronic and thermal Free Energies: -1128.768236

Standard orientation. Coordinates (Angstroms):

S	-0.460289000	-1.212109000	-0.580493000
C	-1.785944000	-0.159590000	-0.156740000
C	-2.944010000	-0.908967000	0.281703000
O	-2.851563000	-2.126669000	0.448686000
C	-4.269354000	-0.229957000	0.560856000
H	-4.156628000	0.706809000	1.113488000
H	-4.799351000	-0.007505000	-0.372419000
H	-4.878469000	-0.926704000	1.139856000
C	-1.595504000	1.170716000	-0.658490000
O	-0.475630000	1.608826000	-0.983267000
C	-2.779003000	2.096319000	-0.867251000
H	-3.617298000	1.608119000	-1.369232000
H	-3.139080000	2.478705000	0.094252000
H	-2.434760000	2.941739000	-1.465207000
C	0.833298000	-0.257497000	-0.194678000
C	1.279664000	0.456210000	1.092802000
C	2.238314000	-0.423842000	-0.743487000
C	2.709970000	0.177428000	0.590890000
C	1.010038000	1.937076000	1.340448000
H	1.190959000	2.547574000	0.455503000
H	-0.026529000	2.089774000	1.653226000
H	1.675323000	2.266109000	2.146147000
C	0.923056000	-0.369490000	2.335884000
H	1.188672000	-1.424992000	2.222857000
H	1.464300000	0.031422000	3.198669000
H	-0.152726000	-0.305801000	2.526731000
C	2.511918000	0.528689000	-1.918991000
H	3.590173000	0.566742000	-2.105057000
H	2.003919000	0.156386000	-2.814383000
H	2.144452000	1.538268000	-1.721299000
C	2.711476000	-1.839834000	-1.052809000
H	2.203587000	-2.231629000	-1.940917000
H	3.788056000	-1.826732000	-1.249676000
H	2.524548000	-2.522853000	-0.218162000
O	3.793689000	0.348435000	1.076167000

$TS_{7'b\rightarrow 3'b}$

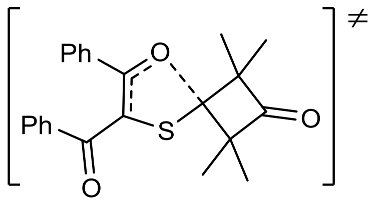

Imaginary Freq.: -29.69 cm^{-1}
Sum of electronic and zero-point Energies= -1511.632695
Sum of electronic and thermal Energies= -1511.607267
Sum of electronic and thermal Enthalpies= -1511.606323
Sum of electronic and thermal Free Energies= -1511.689630

Standard orientation. Coordinates (Angstroms):

C	2.052135000	-0.250926000	-0.200130000
S	0.852930000	0.744225000	-0.725938000
C	-0.579191000	0.112333000	0.032125000
C	-0.740753000	-1.311295000	-0.227022000
C	-1.456529000	1.077411000	0.682413000
O	-2.195061000	0.802476000	1.625574000
O	0.197383000	-1.996725000	-0.664909000
C	-2.087855000	-1.979139000	-0.184322000
C	-3.028606000	-1.839099000	0.839274000
C	-2.348762000	-2.875327000	-1.231464000
C	-4.214806000	-2.569447000	0.798586000
H	-2.831240000	-1.159573000	1.658312000
C	-3.542543000	-3.580585000	-1.278954000
H	-1.596105000	-3.008443000	-2.002009000
C	-4.481801000	-3.430923000	-0.259346000
H	-4.934050000	-2.458696000	1.605868000
H	-3.737258000	-4.257145000	-2.107072000
H	-5.413523000	-3.990102000	-0.288403000
C	-1.423304000	2.505604000	0.210901000
C	-1.403128000	2.839134000	-1.146701000
C	-1.513133000	3.524181000	1.163793000
C	-1.448877000	4.172673000	-1.543989000
H	-1.388620000	2.049368000	-1.894630000
C	-1.539719000	4.855529000	0.768306000
H	-1.568826000	3.250170000	2.213337000
C	-1.506064000	5.182025000	-0.587091000
H	-1.449446000	4.422428000	-2.601559000
H	-1.596200000	5.642374000	1.515775000
H	-1.536021000	6.223394000	-0.896575000
C	2.426327000	-0.899062000	1.134053000
C	3.432946000	-0.374137000	-0.812400000
C	2.194433000	0.046958000	2.318305000
H	2.571334000	1.055451000	2.121099000
H	1.125076000	0.111082000	2.541633000
H	2.716869000	-0.349736000	3.194507000
C	1.983252000	-2.319287000	1.488398000
H	0.956793000	-2.319063000	1.861748000
H	2.028412000	-2.998131000	0.636287000
H	2.651340000	-2.683758000	2.276302000
C	4.101696000	0.897437000	-1.321346000
H	5.155880000	0.691186000	-1.531433000
H	3.627792000	1.242237000	-2.246923000
H	4.051696000	1.705026000	-0.584382000
C	3.866868000	-0.859856000	0.582655000
C	3.499706000	-1.503804000	-1.854526000
H	3.031330000	-1.168266000	-2.785417000

H	4.548432000	-1.751272000	-2.048464000
H	2.975116000	-2.402383000	-1.520851000
O	4.936504000	-1.090267000	1.072931000

$TS_{7'c \rightarrow 3'c}$

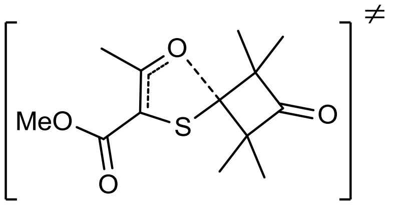

Imaginary Freq.: -82.03 cm^{-1}
Sum of electronic and zero-point Energies:-1203.871773
Sum of electronic and thermal Energies: -1203.851454
Sum of electronic and thermal Enthalpies: -1203.850509
Sum of electronic and thermal Free Energies: -1203.920343

Standard orientation. Coordinates (Angstroms):

S	-0.194181000	-1.118056000	-0.693635000
C	-1.477964000	-0.035036000	-0.265828000
C	-2.708373000	-0.717274000	0.069845000
O	-2.799277000	-1.928647000	0.195997000
C	-4.980591000	-0.557555000	0.573232000
H	-4.878944000	-1.121190000	1.504626000
H	-5.725883000	0.231252000	0.684344000
H	-5.271717000	-1.248750000	-0.222419000
C	-1.224412000	1.314915000	-0.684805000
O	-0.079013000	1.719205000	-0.953459000
C	-2.376897000	2.281020000	-0.859537000
H	-3.170817000	1.861550000	-1.481672000
H	-2.824609000	2.507155000	0.112950000
H	-1.984519000	3.196434000	-1.305385000
C	1.127816000	-0.251879000	-0.206619000
C	1.551242000	0.385025000	1.126491000
C	2.543492000	-0.471526000	-0.705126000
C	2.983725000	0.029544000	0.680663000
C	1.374684000	1.875145000	1.406489000
H	1.635640000	2.495449000	0.548715000
H	0.338823000	2.092937000	1.679501000
H	2.026186000	2.133656000	2.248397000
C	1.076306000	-0.445626000	2.324724000
H	1.273393000	-1.513801000	2.191933000
H	1.600799000	-0.107878000	3.224095000
H	-0.000196000	-0.309573000	2.467124000
C	2.925121000	0.522088000	-1.814718000
H	4.011612000	0.509450000	-1.949454000
H	2.442117000	0.224445000	-2.750966000
H	2.602843000	1.539444000	-1.581286000
C	2.951690000	-1.894950000	-1.068208000
H	2.467881000	-2.211303000	-1.998880000
H	4.035739000	-1.934210000	-1.214008000
H	2.686814000	-2.607578000	-0.280699000
O	4.049072000	0.095164000	1.228309000
O	-3.764945000	0.107785000	0.249355000

TS$_{7'd\rightarrow 3'd}$

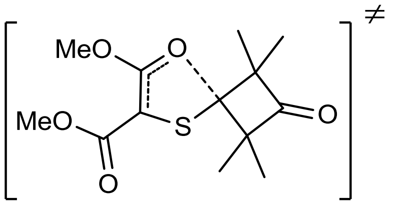

Imaginary Freq.: -201.01 cm^{-1}
Sum of electronic and zero-point Energies:-1279.007178
Sum of electronic and thermal Energies: -1278.985906
Sum of electronic and thermal Enthalpies: -1278.984962
Sum of electronic and thermal Free Energies: -1279.057261

Standard orientation. Coordinates (Angstroms):

S	-0.049271000	-1.177788000	-0.973466000
C	-1.359509000	-0.285426000	-0.251731000
C	-2.594884000	-1.019197000	-0.108388000
O	-2.690021000	-2.214986000	-0.342037000
C	-4.852881000	-0.992858000	0.447664000
H	-5.586298000	-0.264213000	0.795947000
H	-5.160341000	-1.412509000	-0.514490000
H	-4.755579000	-1.810400000	1.167832000
O	-3.632036000	-0.275481000	0.324201000
C	-1.090300000	1.111457000	-0.312340000
O	0.063435000	1.576150000	-0.480669000
O	-2.137591000	1.933409000	-0.225473000
C	-1.837980000	3.324265000	-0.259174000
H	-2.790004000	3.827516000	-0.087376000
H	-1.119847000	3.589242000	0.522192000
H	-1.428256000	3.612138000	-1.231056000
C	1.172335000	-0.276330000	-0.264122000
C	1.571543000	-0.110025000	1.217999000
C	2.596384000	-0.194958000	-0.787830000
C	3.018676000	-0.145709000	0.690374000
C	1.254266000	1.164477000	1.995049000
H	1.472723000	2.066170000	1.421776000
H	0.197826000	1.180835000	2.279426000
H	1.864577000	1.165706000	2.904446000
C	1.211298000	-1.345891000	2.049564000
H	1.508082000	-2.276969000	1.557713000
H	1.722952000	-1.288984000	3.015374000
H	0.130338000	-1.376590000	2.217514000
C	2.907164000	1.117557000	-1.519898000
H	3.989929000	1.199153000	-1.658761000
H	2.416500000	1.115499000	-2.498294000
H	2.549498000	1.988608000	-0.967268000
C	3.096528000	-1.399608000	-1.580669000
H	2.625303000	-1.433918000	-2.569058000
H	4.179135000	-1.315620000	-1.717517000
H	2.890582000	-2.343380000	-1.065695000
O	4.086886000	-0.152116000	1.236949000

16. Oxathioles 3'

Gas phase, 6-31G(d), PBE1PBE

Oxathiole 3'a

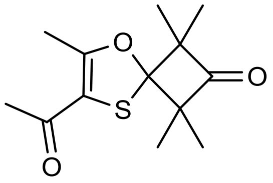

Sum of electronic and zero-point Energies:-1128.773069
Sum of electronic and thermal Energies: -1128.753847
Sum of electronic and thermal Enthalpies: -1128.752902
Sum of electronic and thermal Free Energies: -1128.820023

Standard orientation. Coordinates (Angstroms):

S	-0.640210000	-1.355740000	-0.041829000
C	-1.904949000	-0.113272000	-0.063920000
C	-3.273845000	-0.610946000	0.083342000
O	-3.446636000	-1.815527000	0.197587000
C	-4.455802000	0.332258000	0.094049000
H	-4.354757000	1.102190000	0.866261000
H	-4.570926000	0.833295000	-0.873837000
H	-5.351325000	-0.259686000	0.291203000
C	-1.383996000	1.115257000	-0.284531000
O	-0.040001000	1.170463000	-0.411779000
C	-2.006011000	2.455406000	-0.449011000
H	-3.092910000	2.418461000	-0.437382000
H	-1.664685000	3.125845000	0.348592000
H	-1.677134000	2.891241000	-1.399352000
C	0.638757000	-0.047294000	-0.085961000
C	1.580702000	0.153444000	1.167183000
C	1.882472000	-0.251172000	-1.022629000
C	2.748702000	0.154801000	0.174601000
C	1.406520000	1.453787000	1.939499000
H	1.363776000	2.322496000	1.277912000
H	0.486290000	1.425622000	2.534618000
H	2.252853000	1.584995000	2.621425000
C	1.640575000	-1.036212000	2.122415000
H	1.764685000	-1.992741000	1.605687000
H	2.488269000	-0.909466000	2.803788000
H	0.718503000	-1.097224000	2.709528000
C	1.974998000	0.675700000	-2.226486000
H	2.966628000	0.577708000	-2.679623000
H	1.222406000	0.404711000	-2.975211000
H	1.824359000	1.722666000	-1.952992000
C	2.127859000	-1.700649000	-1.441764000
H	1.383194000	-2.010351000	-2.182316000
H	3.124802000	-1.784427000	-1.886150000
H	2.077007000	-2.401558000	-0.602231000
O	3.914941000	0.426491000	0.284019000

Oxathiole 3'b

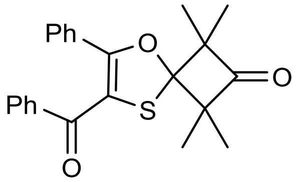

Sum of electronic and zero-point Energies= -1511.691335
Sum of electronic and thermal Energies= -1511.666094
Sum of electronic and thermal Enthalpies= -1511.665150
Sum of electronic and thermal Free Energies= -1511.747565

Standard orientation. Coordinates (Angstroms):

C	-1.519650000	-0.969739000	-0.040828000
S	0.207931000	-1.562171000	0.027275000
C	0.738409000	0.123590000	0.251547000
C	-0.269099000	0.987720000	-0.051116000
C	2.122554000	0.406149000	0.680246000
O	2.387321000	1.311126000	1.460641000
O	-1.426058000	0.383040000	-0.451992000
C	-0.327654000	2.448884000	-0.178008000
C	0.401833000	3.318730000	0.643435000
C	-1.181578000	2.990268000	-1.154324000
C	0.291930000	4.694485000	0.470947000
H	1.043956000	2.907593000	1.412215000
C	-1.279845000	4.364237000	-1.321258000
H	-1.757229000	2.323939000	-1.788137000
C	-0.542392000	5.223303000	-0.508907000
H	0.860299000	5.356923000	1.118082000
H	-1.936605000	4.766233000	-2.087958000
H	-0.624117000	6.299459000	-0.636037000
C	3.222438000	-0.464268000	0.166403000
C	3.176222000	-1.074765000	-1.091189000
C	4.371417000	-0.595884000	0.954297000
C	4.261426000	-1.817026000	-1.546635000
H	2.300984000	-0.951222000	-1.722185000
C	5.445200000	-1.352308000	0.505821000
H	4.399920000	-0.093043000	1.916145000
C	5.391006000	-1.964253000	-0.746248000
H	4.225405000	-2.278685000	-2.529436000
H	6.329032000	-1.462622000	1.128078000
H	6.233895000	-2.552015000	-1.100179000
C	-2.440689000	-1.142855000	1.234455000
C	-2.537631000	-1.760800000	-0.927655000
C	-1.947584000	-2.156375000	2.263625000
H	-1.617933000	-3.096582000	1.810903000
H	-1.102397000	-1.744869000	2.824873000
H	-2.759527000	-2.384467000	2.961910000
C	-2.893430000	0.143850000	1.913081000
H	-2.080249000	0.570854000	2.510620000
H	-3.217391000	0.897059000	1.190741000
H	-3.733298000	-0.077898000	2.579455000
C	-2.098999000	-3.176916000	-1.299977000
H	-2.958599000	-3.729690000	-1.692178000
H	-1.322172000	-3.143667000	-2.071442000
H	-1.705266000	-3.737518000	-0.445439000
C	-3.477340000	-1.751199000	0.284108000
C	-3.067986000	-1.023139000	-2.149791000
H	-2.284654000	-0.927872000	-2.909549000
H	-3.901257000	-1.590331000	-2.576547000

H	-3.427610000	-0.021569000	-1.901266000
O	-4.626691000	-2.074845000	0.423695000

Oxathiole 3'c

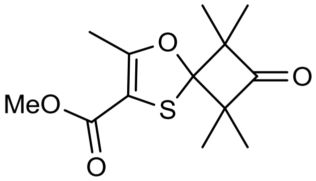

Sum of electronic and zero-point Energies:-1203.924762
Sum of electronic and thermal Energies: -1203.904477
Sum of electronic and thermal Enthalpies: -1203.903533
Sum of electronic and thermal Free Energies: -1203.973315

Standard orientation. Coordinates (Angstroms):

S	-0.330226000	-1.361940000	-0.066341000
C	-1.582209000	-0.111311000	-0.113719000
C	-2.961729000	-0.560997000	0.042192000
O	-3.263235000	-1.728324000	0.178223000
C	-5.220366000	0.003815000	0.165133000
H	-5.497266000	-0.672067000	-0.648118000
H	-5.361210000	-0.514509000	1.117119000
H	-5.823302000	0.911622000	0.130784000
C	-1.061759000	1.107772000	-0.370898000
O	0.283609000	1.149187000	-0.502054000
C	-1.706977000	2.428751000	-0.574357000
H	-2.790994000	2.342378000	-0.540762000
H	-1.372203000	3.132083000	0.197256000
H	-1.398434000	2.838371000	-1.543134000
C	0.950173000	-0.051714000	-0.104466000
C	1.839984000	0.193038000	1.179234000
C	2.228853000	-0.290818000	-0.981322000
C	3.047681000	0.152235000	0.236354000
C	1.639073000	1.523357000	1.891888000
H	1.623333000	2.364688000	1.194751000
H	0.696279000	1.519787000	2.451116000
H	2.458680000	1.681248000	2.600312000
C	1.857176000	-0.958719000	2.181266000
H	1.997706000	-1.934806000	1.707036000
H	2.677939000	-0.807606000	2.890112000
H	0.912676000	-0.994616000	2.733840000
C	2.373353000	0.596063000	-2.209999000
H	3.381605000	0.479592000	-2.620052000
H	1.648944000	0.304146000	-2.978241000
H	2.217062000	1.651797000	-1.976477000
C	2.486746000	-1.753873000	-1.341949000
H	1.776909000	-2.085004000	-2.107136000
H	3.503082000	-1.854837000	-1.735858000
H	2.393846000	-2.427199000	-0.483507000
O	4.211151000	0.416883000	0.384899000
O	-3.869154000	0.436050000	0.023464000

Oxathiole 3'd

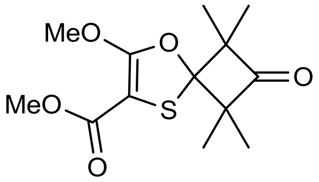

Sum of electronic and zero-point Energies:-1279.044990
Sum of electronic and thermal Energies: -1279.023605
Sum of electronic and thermal Enthalpies: -1279.022661
Sum of electronic and thermal Free Energies: -1279.095193

Standard orientation. Coordinates (Angstroms):

S	-0.216169000	-1.537250000	-0.368578000
C	-1.523256000	-0.357050000	-0.155893000
C	-2.882843000	-0.841513000	-0.025753000
O	-3.174004000	-2.021947000	-0.040859000
C	-5.131875000	-0.303262000	0.229331000
H	-5.735306000	0.599595000	0.329897000
H	-5.432286000	-0.866699000	-0.658519000
H	-5.253318000	-0.944071000	1.107217000
O	-3.787247000	0.146989000	0.112443000
C	-1.038254000	0.903867000	-0.260390000
O	0.292537000	1.035554000	-0.409204000
O	-1.740692000	2.021476000	-0.282282000
C	-1.039179000	3.259889000	-0.211017000
H	-1.815218000	4.024827000	-0.231761000
H	-0.467619000	3.334661000	0.718811000
H	-0.369482000	3.383615000	-1.066115000
C	0.997715000	-0.187742000	-0.137771000
C	1.803112000	-0.087173000	1.219168000
C	2.328580000	-0.252434000	-0.960513000
C	3.063649000	0.028826000	0.354837000
C	1.526914000	1.136168000	2.083491000
H	1.576010000	2.065510000	1.509824000
H	0.537020000	1.063418000	2.548419000
H	2.277091000	1.192329000	2.878737000
C	1.798242000	-1.357190000	2.066562000
H	1.995700000	-2.259409000	1.480544000
H	2.571654000	-1.278151000	2.837495000
H	0.824132000	-1.486428000	2.549242000
C	2.507506000	0.812044000	-2.034406000
H	3.538486000	0.781215000	-2.400624000
H	1.832351000	0.623143000	-2.875983000
H	2.310414000	1.818298000	-1.656110000
C	2.660077000	-1.637657000	-1.516443000
H	2.007219000	-1.871523000	-2.363682000
H	3.700132000	-1.649067000	-1.857405000
H	2.545209000	-2.432880000	-0.772457000
O	4.208680000	0.292549000	0.608963000

17. Transition states for 1,3-electrocyclizations of C=S-ylides 7' to thiiranes 4'

Gas phase, 6-31G(d), PBE1PBE

TS$_{7'a\rightarrow4'a}$

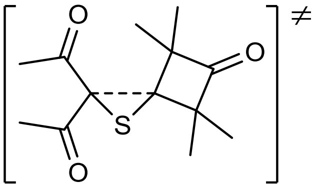

Imaginary Freq.: -180.41 cm^{-1}
Sum of electronic and zero-point Energies:-1128.697492
Sum of electronic and thermal Energies: -1128.677921
Sum of electronic and thermal Enthalpies: -1128.676977
Sum of electronic and thermal Free Energies: -1128.744783

Standard orientation. Coordinates (Angstroms):

S	0.321025000	0.180400000	1.727284000
C	1.514114000	0.259731000	0.428643000
C	2.408178000	-0.898386000	0.368996000
O	2.122321000	-1.933686000	0.966293000
C	3.730449000	-0.839750000	-0.372471000
H	4.309709000	0.054493000	-0.126079000
H	4.296343000	-1.729077000	-0.089540000
H	3.583209000	-0.852552000	-1.457465000
C	1.602834000	1.568826000	-0.246250000
O	0.974446000	2.547538000	0.138862000
C	2.489893000	1.725426000	-1.469730000
H	2.418550000	0.880710000	-2.160479000
H	2.178459000	2.642197000	-1.974370000
H	3.541209000	1.839327000	-1.185176000
C	-0.753483000	-0.014098000	0.445845000
C	-1.020030000	-1.177473000	-0.520237000
C	-2.071009000	0.687445000	0.182898000
C	-2.414005000	-0.538017000	-0.681449000
C	-0.245522000	-1.271084000	-1.832366000
H	-0.116041000	-0.291251000	-2.301635000
H	0.740174000	-1.710475000	-1.654891000
H	-0.796311000	-1.915929000	-2.525604000
C	-1.068125000	-2.533503000	0.192498000
H	-1.694896000	-2.498421000	1.089789000
H	-1.487319000	-3.283163000	-0.486845000
H	-0.055629000	-2.819447000	0.491675000
C	-1.955631000	1.957659000	-0.674225000
H	-2.963862000	2.273680000	-0.960269000
H	-1.459250000	2.748146000	-0.106759000
H	-1.375386000	1.790188000	-1.586462000
C	-2.933483000	0.932792000	1.416839000
H	-2.491319000	1.720994000	2.035967000
H	-3.931982000	1.255829000	1.105383000
H	-3.042385000	0.030061000	2.026774000
O	-3.408611000	-0.886242000	-1.255719000

TS$_{7'b\rightarrow4'b}$

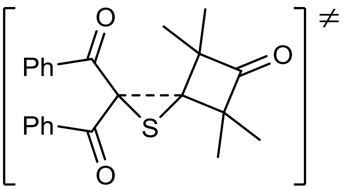

Imaginary Freq.: -145.11 cm^{-1}
Sum of electronic and zero-point Energies= -1511.615047
Sum of electronic and thermal Energies= -1511.589420
Sum of electronic and thermal Enthalpies= -1511.588476
Sum of electronic and thermal Free Energies= -1511.671629

Standard orientation. Coordinates (Angstroms):

C	1.583376000	-1.035883000	-0.409537000
S	0.802019000	-0.302748000	-1.713455000
C	-0.470568000	-0.061697000	-0.526251000
C	-1.717583000	-0.763435000	-0.871178000
C	-0.231617000	0.936557000	0.546796000
O	-0.597193000	0.757035000	1.704560000
O	-1.705739000	-1.523762000	-1.841776000
C	-3.038097000	-0.487038000	-0.215886000
C	-3.240032000	-0.298603000	1.154844000
C	-4.149541000	-0.510636000	-1.071565000
C	-4.529735000	-0.121998000	1.650827000
H	-2.392561000	-0.292331000	1.828415000
C	-5.429190000	-0.310068000	-0.575276000
H	-3.985501000	-0.694017000	-2.128721000
C	-5.623342000	-0.116267000	0.791959000
H	-4.675403000	0.012542000	2.719313000
H	-6.278765000	-0.314325000	-1.252950000
H	-6.625871000	0.032162000	1.184936000
C	0.425311000	2.243827000	0.209539000
C	0.340490000	2.837519000	-1.053855000
C	1.045998000	2.946543000	1.249204000
C	0.888786000	4.096595000	-1.278807000
H	-0.180984000	2.327990000	-1.857899000
C	1.611315000	4.193904000	1.020462000
H	1.066692000	2.495488000	2.236709000
C	1.535654000	4.770887000	-0.247024000
H	0.805696000	4.553078000	-2.261223000
H	2.103088000	4.723744000	1.831792000
H	1.971054000	5.750408000	-0.426084000
C	1.315439000	-2.369912000	0.302870000
C	3.023720000	-0.912970000	0.056362000
C	0.424618000	-2.392570000	1.545033000
H	0.579289000	-1.522908000	2.187535000
H	-0.628151000	-2.402893000	1.247125000
H	0.638838000	-3.301675000	2.117221000
C	0.945801000	-3.500511000	-0.661146000
H	-0.061031000	-3.332898000	-1.055786000
H	1.638336000	-3.557725000	-1.507450000
H	0.976837000	-4.456148000	-0.127490000
C	3.271095000	0.125611000	1.157886000
H	4.252346000	-0.061569000	1.606284000
H	3.256545000	1.134644000	0.734887000
H	2.515450000	0.076036000	1.947733000
C	2.815805000	-2.320684000	0.647384000
C	4.057345000	-0.794845000	-1.059180000
H	3.999025000	0.193070000	-1.529838000
H	5.061771000	-0.919555000	-0.641705000

H	3.913718000	-1.556823000	-1.832180000
O	3.563805000	-3.104309000	1.163953000

TS$_{7'c\rightarrow 4'c}$

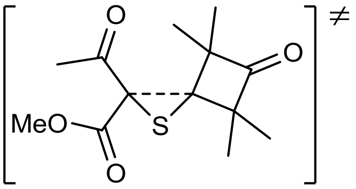

Imaginary Freq.: -191.15 cm^{-1}
Sum of electronic and zero-point Energies:-1203.849823
Sum of electronic and thermal Energies: -1203.829291
Sum of electronic and thermal Enthalpies: -1203.828346
Sum of electronic and thermal Free Energies: -1203.898691

Standard orientation. Coordinates (Angstroms):

S	-0.041610000	0.413899000	1.744044000
C	1.161962000	0.588247000	0.479144000
C	2.229687000	-0.416949000	0.522126000
O	2.312631000	-1.299473000	1.357257000
C	4.178864000	-1.259708000	-0.450927000
H	4.804740000	-1.035047000	-1.315244000
H	4.754091000	-1.165070000	0.473784000
H	3.785999000	-2.277987000	-0.515594000
O	3.119670000	-0.307412000	-0.488114000
C	1.131935000	1.854468000	-0.281384000
O	0.158430000	2.596323000	-0.282648000
C	2.371663000	2.282456000	-1.042030000
H	3.277462000	2.213418000	-0.433962000
H	2.528409000	1.641832000	-1.914890000
H	2.209786000	3.312091000	-1.365872000
C	-0.999642000	0.010064000	0.414284000
C	-1.028354000	-1.242306000	-0.472078000
C	-2.417615000	0.447670000	0.085151000
C	-2.526611000	-0.912919000	-0.626276000
C	-0.300115000	-1.168770000	-1.816711000
H	-0.467706000	-0.213600000	-2.322722000
H	0.777090000	-1.287075000	-1.666924000
H	-0.661734000	-1.972651000	-2.466736000
C	-0.736142000	-2.553251000	0.254435000
H	-1.313426000	-2.637270000	1.180615000
H	-1.004441000	-3.394399000	-0.393242000
H	0.325354000	-2.614701000	0.512006000
C	-2.584570000	1.619713000	-0.890382000
H	-3.626490000	1.639611000	-1.226600000
H	-2.331401000	2.556310000	-0.390330000
H	-1.935441000	1.531845000	-1.764598000
C	-3.308167000	0.631007000	1.311681000
H	-3.028172000	1.544443000	1.847541000
H	-4.351516000	0.721622000	0.993007000
H	-3.237388000	-0.215327000	2.003149000
O	-3.450334000	-1.520534000	-1.094032000

TS$_{7'd\rightarrow4'd}$

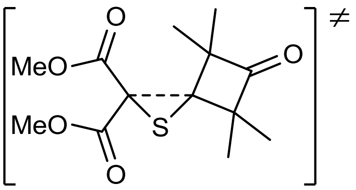

Imaginary Freq.: -164.35 cm^{-1}
Sum of electronic and zero-point Energies:-1278.996056
Sum of electronic and thermal Energies: -1278.974390
Sum of electronic and thermal Enthalpies: -1278.973445
Sum of electronic and thermal Free Energies: -1279.047005

Standard orientation. Coordinates (Angstroms):

S	0.196171000	-0.237272000	-1.833240000
C	-1.078864000	0.117707000	-0.698204000
C	-2.059078000	-0.961232000	-0.526648000
O	-1.963505000	-2.038002000	-1.093344000
C	-4.033977000	-1.719598000	0.437717000
H	-4.771838000	-1.345732000	1.148264000
H	-4.505317000	-1.936366000	-0.524762000
H	-3.569074000	-2.635894000	0.812449000
O	-3.071647000	-0.678577000	0.307784000
C	-1.159824000	1.519815000	-0.256049000
O	-0.437425000	2.421305000	-0.643037000
O	-2.148046000	1.729781000	0.630880000
C	-2.305871000	3.086778000	1.026843000
H	-3.154572000	3.092913000	1.711717000
H	-1.406528000	3.454099000	1.529690000
H	-2.506354000	3.725447000	0.162351000
C	1.147885000	-0.177967000	-0.442080000
C	1.241153000	-1.097478000	0.781071000
C	2.495858000	0.483582000	-0.228280000
C	2.688888000	-0.571231000	0.877006000
C	0.424140000	-0.721026000	2.019779000
H	0.470883000	0.350662000	2.234506000
H	-0.626925000	-0.986335000	1.872857000
H	0.815096000	-1.263688000	2.887090000
C	1.120839000	-2.587025000	0.463024000
H	1.767672000	-2.874551000	-0.371941000
H	1.414349000	-3.171822000	1.340960000
H	0.089275000	-2.829401000	0.190212000
C	2.460770000	1.914574000	0.325309000
H	3.474785000	2.195316000	0.627877000
H	2.095205000	2.600774000	-0.442017000
H	1.800710000	2.009418000	1.191366000
C	3.443568000	0.382461000	-1.421009000
H	3.114592000	1.050905000	-2.224276000
H	4.450145000	0.682026000	-1.111905000
H	3.499389000	-0.637203000	-1.816244000
O	3.632730000	-0.897053000	1.543063000

18. Thiiranes 4'

Gas phase, 6-31G(d), PBE1PBE

Thiirane 4'a

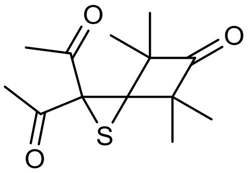

Sum of electronic and zero-point Energies:-1128.760774
Sum of electronic and thermal Energies: -1128.740899
Sum of electronic and thermal Enthalpies: -1128.739954
Sum of electronic and thermal Free Energies: -1128.807905

Standard orientation. Coordinates (Angstroms):

S	-0.488089000	-0.113142000	-1.851974000
C	-1.118807000	0.032931000	-0.158675000
C	-1.989533000	-1.175990000	0.157230000
O	-1.612679000	-2.126432000	0.805053000
C	-3.371935000	-1.144301000	-0.458253000
H	-3.732327000	-0.131584000	-0.662085000
H	-3.329671000	-1.674331000	-1.416695000
H	-4.071946000	-1.677492000	0.189133000
C	-1.694573000	1.378207000	0.279582000
O	-1.623132000	2.370151000	-0.406300000
C	-2.316461000	1.381772000	1.655828000
H	-1.657201000	0.897778000	2.384827000
H	-2.525397000	2.409392000	1.958469000
H	-3.255796000	0.814744000	1.651909000
C	0.372279000	-0.066691000	-0.242367000
C	1.365859000	-1.180457000	0.216008000
C	1.425355000	1.065777000	0.008863000
C	2.429400000	-0.077530000	0.196780000
C	1.210903000	-1.672086000	1.663110000
H	0.951046000	-0.866137000	2.356500000
H	0.431893000	-2.431907000	1.724242000
H	2.168070000	-2.095069000	1.987456000
C	1.600706000	-2.349670000	-0.724071000
H	1.766685000	-2.017677000	-1.753234000
H	2.483927000	-2.908405000	-0.397217000
H	0.737059000	-3.022372000	-0.714250000
C	1.250060000	1.882633000	1.293974000
H	2.215033000	2.322947000	1.566906000
H	0.537735000	2.698891000	1.138965000
H	0.910772000	1.278455000	2.140779000
C	1.755557000	1.989713000	-1.155591000
H	0.908302000	2.649227000	-1.366556000
H	2.630338000	2.595952000	-0.897819000
H	1.988128000	1.429617000	-2.066006000
O	3.628299000	-0.099670000	0.277714000

Thiirane 4'b

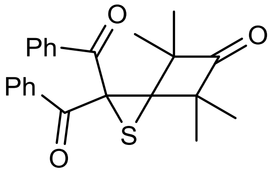

Sum of electronic and zero-point Energies= -1511.686110
Sum of electronic and thermal Energies= -1511.660689
Sum of electronic and thermal Enthalpies= -1511.659745
Sum of electronic and thermal Free Energies= -1511.741078

Standard orientation. Coordinates (Angstroms):

C	-1.680110000	-0.533934000	-0.183157000
S	-0.945816000	-1.745415000	-1.327354000
C	-0.188609000	-0.437768000	-0.320421000
C	0.346557000	0.794486000	-1.054246000
C	0.733798000	-1.108916000	0.690575000
O	0.369350000	-1.371722000	1.822407000
O	0.248393000	0.896780000	-2.261342000
C	0.964098000	1.877737000	-0.238227000
C	0.871639000	1.936571000	1.157591000
C	1.636869000	2.895464000	-0.926729000
C	1.446839000	2.997449000	1.849703000
H	0.347527000	1.164519000	1.712018000
C	2.216551000	3.947099000	-0.232584000
H	1.689613000	2.836781000	-2.009423000
C	2.121448000	3.999713000	1.158070000
H	1.367092000	3.040004000	2.932178000
H	2.742438000	4.729362000	-0.772770000
H	2.573354000	4.824513000	1.702533000
C	2.112160000	-1.454719000	0.236208000
C	2.523512000	-1.405413000	-1.101578000
C	3.024267000	-1.856300000	1.220157000
C	3.828716000	-1.744834000	-1.441517000
H	1.830786000	-1.118742000	-1.886544000
C	4.326823000	-2.189188000	0.877833000
H	2.684521000	-1.898481000	2.250230000
C	4.731537000	-2.133092000	-0.455287000
H	4.138568000	-1.710352000	-2.482070000
H	5.029158000	-2.494126000	1.648643000
H	5.750949000	-2.395638000	-0.725405000
C	-2.756294000	0.454912000	-0.752819000
C	-2.601051000	-0.948499000	1.007958000
C	-2.532953000	1.945977000	-0.477755000
H	-2.107310000	2.143405000	0.509861000
H	-1.867668000	2.381419000	-1.229688000
H	-3.497411000	2.461780000	-0.538152000
C	-3.196653000	0.254984000	-2.197023000
H	-2.388242000	0.532824000	-2.880079000
H	-3.473739000	-0.783741000	-2.399705000
H	-4.070497000	0.883986000	-2.396816000
C	-2.342745000	-0.241547000	2.346934000
H	-3.260609000	-0.288270000	2.943359000
H	-1.531292000	-0.734050000	2.883310000
H	-2.082882000	0.814424000	2.222483000
C	-3.702998000	-0.179153000	0.272298000
C	-2.849477000	-2.430429000	1.226985000
H	-1.957084000	-2.901773000	1.650404000
H	-3.683578000	-2.561269000	1.924357000
H	-3.100519000	-2.943473000	0.293761000

O	-4.893202000	-0.112565000	0.427356000

Thiirane 4'c

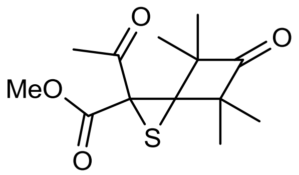

Sum of electronic and zero-point Energies:-1203.913055
Sum of electronic and thermal Energies: -1203.892532
Sum of electronic and thermal Enthalpies: -1203.891588
Sum of electronic and thermal Free Energies: -1203.960781

Standard orientation. Coordinates (Angstroms):

S	0.164193000	0.063935000	1.913154000
C	0.838654000	0.319981000	0.248373000
C	1.904217000	-0.710686000	-0.041048000
O	1.741994000	-1.883125000	-0.277218000
C	4.218852000	-1.033791000	-0.081111000
H	5.110348000	-0.420066000	0.045468000
H	4.180132000	-1.818366000	0.678405000
H	4.204746000	-1.495756000	-1.071552000
O	3.113845000	-0.141959000	0.072522000
C	1.150080000	1.755165000	-0.175897000
O	0.866942000	2.699436000	0.521662000
C	1.768085000	1.928504000	-1.544710000
H	1.492874000	1.123448000	-2.233821000
H	1.461333000	2.894300000	-1.951721000
H	2.858276000	1.923351000	-1.445033000
C	-0.608110000	-0.076345000	0.277098000
C	-1.322301000	-1.366273000	-0.234154000
C	-1.843840000	0.823703000	-0.066465000
C	-2.579310000	-0.495593000	-0.329342000
C	-0.933828000	-1.827267000	-1.645839000
H	-0.773165000	-0.988630000	-2.331607000
H	-0.020929000	-2.423174000	-1.610541000
H	-1.750071000	-2.435053000	-2.051127000
C	-1.409165000	-2.546895000	0.717692000
H	-1.737815000	-2.238982000	1.715014000
H	-2.128650000	-3.275896000	0.330398000
H	-0.429405000	-3.024746000	0.808673000
C	-1.745510000	1.670407000	-1.340236000
H	-2.758263000	1.902437000	-1.686979000
H	-1.231797000	2.614924000	-1.138194000
H	-1.225952000	1.156611000	-2.155138000
C	-2.437312000	1.648916000	1.067053000
H	-1.760945000	2.467818000	1.330940000
H	-3.399435000	2.063084000	0.747839000
H	-2.608440000	1.044877000	1.963081000
O	-3.736907000	-0.753364000	-0.524847000

Thiirane 4'd

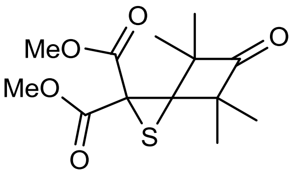

Sum of electronic and zero-point Energies:-1279.064017
Sum of electronic and thermal Energies: -1279.042473
Sum of electronic and thermal Enthalpies: -1279.041529
Sum of electronic and thermal Free Energies: -1279.113489

Standard orientation. Coordinates (Angstroms):

S	-0.092827000	-0.466661000	1.966492000
C	0.720640000	-0.057051000	0.401043000
C	1.619903000	-1.187121000	-0.048851000
O	1.269691000	-2.243056000	-0.515852000
C	3.839633000	-1.902924000	-0.079435000
H	4.809917000	-1.497875000	0.206798000
H	3.618260000	-2.810697000	0.487472000
H	3.818689000	-2.135000000	-1.147241000
O	2.892076000	-0.881026000	0.227822000
C	1.313945000	1.331786000	0.261630000
O	1.278979000	2.209107000	1.085010000
O	1.840787000	1.462255000	-0.963410000
C	2.431259000	2.733637000	-1.232085000
H	2.852986000	2.656107000	-2.233957000
H	1.674974000	3.522385000	-1.194793000
H	3.211593000	2.953728000	-0.499546000
C	-0.768157000	-0.171398000	0.305969000
C	-1.663825000	-1.188993000	-0.465285000
C	-1.787639000	0.995524000	0.087652000
C	-2.729153000	-0.088226000	-0.451300000
C	-1.261961000	-1.458419000	-1.922655000
H	-0.887515000	-0.561814000	-2.426994000
H	-0.486048000	-2.223610000	-1.965660000
H	-2.146971000	-1.804743000	-2.467549000
C	-2.031048000	-2.479642000	0.245657000
H	-2.375865000	-2.295255000	1.267809000
H	-2.835162000	-2.983043000	-0.301137000
H	-1.159655000	-3.139691000	0.287927000
C	-1.425789000	2.021444000	-0.991639000
H	-2.347082000	2.480567000	-1.365856000
H	-0.800790000	2.814898000	-0.569019000
H	-0.896833000	1.577639000	-1.839721000
C	-2.311222000	1.700734000	1.331183000
H	-1.521683000	2.312431000	1.778966000
H	-3.152351000	2.344901000	1.054313000
H	-2.661722000	0.989866000	2.085460000
O	-3.895163000	-0.076071000	-0.742627000

19. Products and transition states for conversion of oxathiole 3'd to alkene 5'd

Disproportionation of two thiirane molecules **4'd** (step ***b2,*** **Table 4,** main text) into thiirane S-sulfide **8'd** and alkene **5'd** followed by decomposition of thiirane S-sulfide **8'd** (step ***b3*** **Table 4,** main text) are characterized by a positive value of the Gibbs free energy change, and therefore are thermodynamically unfavorable. Hence, the driving force of the alkene **5'd** formation from thiirane **4'd** is extrusion of S_2 followed by its tetramerization to the most stable rhombic modification of sulfur S_8 (step ***b4*** **Table 4,** main text), which is characterized by a large negative value of ΔG_{b4},–16.9 kcal·mol^{-1}. The negative value of the total Gibbs free energy change on the way from thiirane **4'd** to alkene **5'd** and rhombic sulfur (–6.6 kcal·mol^{-1}) indicates that this process is thermodynamically favored.

The desulfurization of thiirane **4'd** to give alkene **5'd** is a bimolecular two-step reaction, because the activation energy of the single-step process, atomic sulfur cleavage, is significantly larger in energy (more than 85 kcal·mol^{-1}). According to the postulated mechanism, alkene **5'd** and thiirane S-sulfide **8'd** result from the interaction of two molecules of thiirane **4'd**. Thereupon thiirane S-sulfide **8'd** undergoes decomposition to produce alkene **5'd** and S_2 molecule. An analogous mechanism was proposed before for the spontaneous desulfurization of matrix isolated oxathiiranes.

Gas phase, 6-31G(d), PBE1PBE

TS$_{4'd\rightarrow5'd+8'd}$

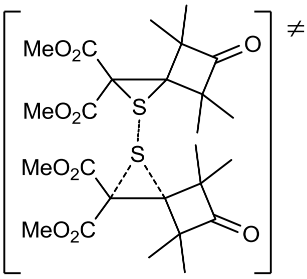

Imaginary Freq.: -361.49 cm^{-1}
Sum of electronic and zero-point Energies:-2558.074509
Sum of electronic and thermal Energies: -2558.029482
Sum of electronic and thermal Enthalpies: -2558.028538
Sum of electronic and thermal Free Energies: -2558.154476

S	1.343264000	-0.654495000	0.923435000
C	2.682156000	0.496091000	0.401821000
C	3.460144000	0.894613000	1.637426000
O	4.172872000	0.171475000	2.289700000
C	3.748257000	2.614647000	3.186767000
H	3.389869000	3.635630000	3.312636000
H	3.431927000	1.987848000	4.023998000
H	4.838298000	2.593605000	3.113525000
O	3.159006000	2.150663000	1.970555000
C	2.356228000	1.634590000	-0.544937000
O	1.284963000	1.880550000	-1.033527000
O	3.483102000	2.304644000	-0.807741000
C	3.329702000	3.415418000	-1.695429000
H	4.315168000	3.875492000	-1.759806000
H	2.999583000	3.073552000	-2.679783000
H	2.596574000	4.120599000	-1.297248000
C	2.799864000	-0.871725000	-0.191498000
C	3.871275000	-1.977503000	0.066905000
C	2.638481000	-1.281419000	-1.694206000
C	3.550844000	-2.462474000	-1.348308000
C	5.326206000	-1.486965000	0.092413000
H	5.504473000	-0.674355000	-0.619814000
H	5.593441000	-1.134667000	1.089169000
H	5.975298000	-2.324571000	-0.184990000
C	3.606234000	-2.976630000	1.181000000
H	2.587217000	-3.374018000	1.137260000
H	4.302135000	-3.816407000	1.085414000
H	3.755124000	-2.500866000	2.154756000

C	3.284802000	-0.347053000	-2.721203000
H	3.479661000	-0.919176000	-3.634322000
H	2.604317000	0.471901000	-2.975676000
H	4.232539000	0.076818000	-2.376425000
C	1.263992000	-1.728870000	-2.175128000
H	0.595363000	-0.875542000	-2.310088000
H	1.387903000	-2.244743000	-3.133257000
H	0.780639000	-2.417184000	-1.476741000
O	3.890608000	-3.430608000	-1.972642000
S	-0.794090000	-0.320609000	0.310790000
C	-3.122043000	-0.513636000	0.375487000
C	-3.235754000	-0.647495000	1.860552000
O	-3.373422000	0.267624000	2.643066000
C	-3.166227000	-2.124055000	3.655528000
H	-3.090638000	-3.201954000	3.801282000
H	-2.327412000	-1.612922000	4.136406000
H	-4.100440000	-1.744228000	4.078099000
O	-3.129906000	-1.924917000	2.248025000
C	-3.498637000	-1.674874000	-0.495507000
O	-2.976704000	-1.980536000	-1.542509000
O	-4.577325000	-2.303459000	-0.004999000
C	-5.025451000	-3.412951000	-0.777601000
H	-5.874084000	-3.826480000	-0.232376000
H	-5.332328000	-3.090069000	-1.776395000
H	-4.231566000	-4.157867000	-0.876667000
C	-2.892905000	0.714160000	-0.222107000
C	-2.956323000	2.166086000	0.298437000
C	-2.987118000	1.144121000	-1.701992000
C	-2.930916000	2.583511000	-1.174606000
C	-4.332992000	2.529927000	0.886015000
H	-5.158764000	2.110444000	0.301576000
H	-4.407931000	2.158335000	1.908687000
H	-4.433350000	3.620645000	0.876512000
C	-1.837476000	2.731178000	1.162691000
H	-0.858978000	2.550633000	0.711998000
H	-1.989293000	3.811658000	1.265143000
H	-1.862188000	2.267991000	2.152257000
C	-4.322952000	0.817969000	-2.383153000
H	-4.455953000	1.494003000	-3.234562000
H	-4.319226000	-0.210929000	-2.753369000
H	-5.177991000	0.945967000	-1.711304000
C	-1.823894000	0.826644000	-2.640404000
H	-1.787679000	-0.249705000	-2.825373000
H	-1.987182000	1.359162000	-3.584321000
H	-0.870618000	1.147755000	-2.214939000
O	-2.883652000	3.651501000	-1.724009000

Thiirane S-sulfide 8'd

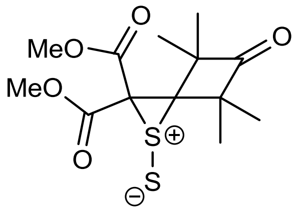

Sum of electronic and zero-point Energies:-1677.063831
Sum of electronic and thermal Energies: -1677.040745
Sum of electronic and thermal Enthalpies: -1677.039801
Sum of electronic and thermal Free Energies: -1677.115545

Standard orientation. Coordinates (Angstroms):

S	0.063383000	0.072358000	1.742231000
C	-0.737051000	0.103076000	-0.043235000
C	-1.579933000	1.344429000	-0.005459000
O	-1.143472000	2.474403000	0.013756000
C	-3.727516000	2.181299000	0.319146000
H	-4.730749000	1.770834000	0.429791000
H	-3.441548000	2.746910000	1.209624000
H	-3.673888000	2.837788000	-0.552948000

O	-2.871726000	1.051825000	0.149409000
C	-1.323304000	-1.191335000	-0.526271000
O	-0.937682000	-2.280297000	-0.173814000
O	-2.271034000	-0.993121000	-1.446160000
C	-2.862302000	-2.187085000	-1.960896000
H	-3.618399000	-1.853907000	-2.671246000
H	-2.110048000	-2.803657000	-2.460043000
H	-3.317951000	-2.765858000	-1.153902000
C	0.754699000	0.184029000	0.004794000
C	1.678233000	1.403045000	-0.338464000
C	1.779496000	-0.836542000	-0.602204000
C	2.727640000	0.359058000	-0.715777000
C	1.300396000	2.189234000	-1.602982000
H	0.888984000	1.547809000	-2.388734000
H	0.565010000	2.958465000	-1.367565000
H	2.209175000	2.657903000	-1.996104000
C	2.075842000	2.344484000	0.787453000
H	2.411360000	1.801775000	1.677302000
H	2.904450000	2.974595000	0.448409000
H	1.233257000	2.986080000	1.061264000
C	1.414722000	-1.375661000	-1.992415000
H	2.342463000	-1.627460000	-2.517210000
H	0.811734000	-2.282383000	-1.903451000
H	0.868350000	-0.649351000	-2.602402000
C	2.351839000	-1.956737000	0.256950000
H	1.623167000	-2.758093000	0.394791000
H	3.233597000	-2.350376000	-0.260512000
H	2.667289000	-1.607589000	1.243286000
O	3.892700000	0.441817000	-0.996256000
S	0.103297000	-1.604124000	2.729664000

Alkene 5'd

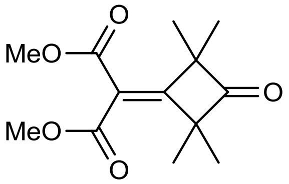

Sum of electronic and zero-point Energies: -881.032540
Sum of electronic and thermal Energies: -881.011970
Sum of electronic and thermal Enthalpies: -881.011026
Sum of electronic and thermal Free Energies: -881.081648

Standard orientation. Coordinates (Angstroms):

C	0.645379000	0.000102000	0.000123000
C	1.407702000	-1.279760000	-0.076616000
O	1.061203000	-2.285262000	-0.657240000
C	3.320696000	-2.397030000	0.653377000
H	4.189641000	-2.170102000	1.271080000
H	2.758110000	-3.234325000	1.075040000
H	3.628403000	-2.655002000	-0.363386000
O	2.530937000	-1.211184000	0.652101000
C	1.407827000	1.279892000	0.076665000
O	1.061319000	2.285656000	0.656826000
O	2.531100000	1.211023000	-0.651986000
C	3.320950000	2.396811000	-0.653586000
H	4.190056000	2.169543000	-1.270936000
H	2.758547000	3.233952000	-1.075805000
H	3.628378000	2.655243000	0.363144000
C	-0.699708000	0.000069000	0.000114000
C	-1.745164000	-1.104231000	-0.184694000
C	-1.745343000	1.104247000	0.184906000
C	-2.796109000	-0.000030000	-0.000233000
C	-1.824693000	-1.652589000	-1.616797000
H	-1.799664000	-0.848797000	-2.360847000
H	-0.983472000	-2.322961000	-1.801682000

H	-2.769999000	-2.192861000	-1.735012000
C	-1.789924000	-2.226108000	0.848710000
H	-1.700374000	-1.840930000	1.869951000
H	-2.748329000	-2.749617000	0.766214000
H	-0.982038000	-2.940819000	0.670379000
C	-1.789737000	2.226174000	-0.848496000
H	-2.748011000	2.749942000	-0.766118000
H	-0.981701000	2.940680000	-0.670003000
H	-1.700133000	1.841023000	-1.869740000
C	-1.825590000	1.652499000	1.616974000
H	-0.984680000	2.323133000	1.802288000
H	-2.771134000	2.192432000	1.734841000
H	-1.800604000	0.848655000	2.360971000
O	-3.997148000	-0.000168000	-0.000085000

$TS_{8'd \rightarrow 5'd+S2}$

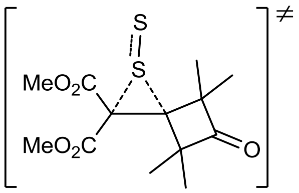

Imaginary Freq.: -62.09 cm^{-1}
Sum of electronic and zero-point Energies:-1677.047173
Sum of electronic and thermal Energies: -1677.023720
Sum of electronic and thermal Enthalpies: -1677.022775
Sum of electronic and thermal Free Energies: -1677.099983

S	-0.475919000	-0.215055000	2.151910000
C	-0.500831000	0.291241000	-0.376270000
C	-1.202737000	1.587231000	-0.122523000
O	-0.646071000	2.631504000	0.142007000
C	-3.261713000	2.630061000	0.167061000
H	-4.312067000	2.356795000	0.068912000
H	-3.039003000	2.937910000	1.192195000
H	-3.010195000	3.447233000	-0.513721000
O	-2.529388000	1.453853000	-0.166740000
C	-1.252951000	-0.872631000	-0.944041000
O	-1.106661000	-2.023648000	-0.592660000
O	-2.058773000	-0.503547000	-1.940656000
C	-2.811750000	-1.563026000	-2.531098000
H	-3.432785000	-1.089995000	-3.291306000
H	-2.145555000	-2.302035000	-2.984297000
H	-3.430919000	-2.056633000	-1.778142000
C	0.861335000	0.177097000	-0.240687000
C	1.987088000	1.202110000	-0.035351000
C	1.826950000	-0.970685000	-0.575458000
C	2.950135000	0.046657000	-0.332161000
C	2.073256000	2.243660000	-1.164388000
H	1.919644000	1.794532000	-2.151615000
H	1.324302000	3.022155000	-1.011996000
H	3.075392000	2.684863000	-1.146772000
C	2.177753000	1.858334000	1.329307000
H	2.149972000	1.122903000	2.137818000
H	3.156674000	2.349178000	1.342375000
H	1.399175000	2.605419000	1.500049000

C	1.771698000	-1.476985000	-2.020802000
H	2.712203000	-1.990743000	-2.245584000
H	0.949329000	-2.186634000	-2.146956000
H	1.649336000	-0.661619000	-2.741483000
C	1.916449000	-2.141892000	0.406255000
H	1.046641000	-2.793561000	0.301795000
H	2.827463000	-2.705883000	0.179752000
H	1.970358000	-1.800397000	1.443550000
O	4.147199000	-0.035506000	-0.365611000
S	-1.178362000	-1.971305000	2.465318000

Disulfur (S=S)

Sum of electronic and zero-point Energies: -796.009635
Sum of electronic and thermal Energies: -796.007174
Sum of electronic and thermal Enthalpies: -796.006230
Sum of electronic and thermal Free Energies: 796.031093
Standard orientation. Coordinates (Angstroms):

S	0.000000000	0.000000000	0.954529000
S	0.000000000	0.000000000	-0.954529000

Orthorombic sulfur (S_8)

Sum of electronic and zero-point Energies:-3184.300566
Sum of electronic and thermal Energies: -3184.289554
Sum of electronic and thermal Enthalpies: -3184.288609
Sum of electronic and thermal Free Energies= -3184.339450
Standard orientation. Coordinates (Angstroms):

S	0.001513000	2.394767000	0.077733000
S	1.727571000	1.429384000	-0.560161000
S	2.152693000	0.061002000	0.919657000
S	1.726255000	-1.852610000	0.099068000
S	-0.000960000	-1.676668000	-0.994917000
S	-1.728584000	-1.850568000	0.098746000
S	-2.152513000	0.063070000	0.919695000
S	-1.725974000	1.431621000	-0.559820000

Calculated at the B3LYP/6-31G(d) level of theory

20. 2-diazo-1,3-dicarbonyl compounds

Gas phase, 6-31G(d), PBE1PBE

3-Diazopentan-2,4-dione (1a)

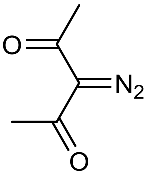

Sum of electronic and zero-point Energies= -453.938696
Sum of electronic and thermal Energies= -453.928960
Sum of electronic and thermal Enthalpies= -453.928016
Sum of electronic and thermal Free Energies= -453.974008

Standard orientation. Coordinates (Angstroms):

C	1.960641000	-1.442407000	-0.000023000
H	1.593925000	-1.988741000	-0.874174000
H	1.593935000	-1.988424000	0.874332000
H	3.051982000	-1.407799000	-0.000036000
C	1.455494000	-0.018516000	-0.000291000
C	-0.025557000	0.196813000	-0.000044000
C	-1.113335000	-0.802817000	0.000325000
C	-2.545121000	-0.289373000	-0.000006000
H	-2.746536000	0.326475000	-0.885044000
H	-3.217809000	-1.148334000	-0.000145000
H	-2.746905000	0.326484000	0.884937000
O	2.203550000	0.946469000	-0.000267000
O	-0.863193000	-1.998892000	-0.000062000
N	-0.361298000	1.476709000	-0.000108000
N	-0.587871000	2.585795000	0.000535000

2-diazo-1,3-diphenylpropane-1,3-dione (1b)

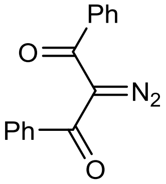

Sum of electronic and zero-point Energies= -837.297402
Sum of electronic and thermal Energies= -837.281786
Sum of electronic and thermal Enthalpies= -837.280841
Sum of electronic and thermal Free Energies= -837.342896

Standard orientation. Coordinates (Angstroms):

C	-0.735328000	-0.478595000	-0.040489000
C	-0.044159000	0.800169000	-0.337312000
C	1.370395000	1.238367000	-0.068881000
O	-0.112096000	-1.525174000	0.084378000
O	1.583228000	2.438743000	0.066133000
N	-0.780257000	1.813718000	-0.772885000
N	-1.334502000	2.707916000	-1.189299000
C	-2.231393000	-0.478143000	0.105246000
C	-2.927335000	-1.597576000	-0.376147000
C	-2.937225000	0.536674000	0.767535000
C	-4.309656000	-1.680378000	-0.235089000
H	-2.367481000	-2.393682000	-0.856449000
C	-4.320068000	0.442075000	0.924921000
H	-2.410282000	1.386386000	1.191924000
C	-5.008900000	-0.659453000	0.414890000
H	-4.842498000	-2.543279000	-0.624781000
H	-4.857056000	1.226395000	1.450851000
H	-6.087153000	-0.727157000	0.531070000
C	2.484859000	0.252980000	0.029091000
C	3.565712000	0.600358000	0.856752000
C	2.555477000	-0.913423000	-0.744719000
C	4.683049000	-0.224448000	0.936469000

H	3.510886000	1.521961000	1.426852000
C	3.688463000	-1.723429000	-0.682012000
H	1.733155000	-1.185977000	-1.393329000
C	4.746673000	-1.387866000	0.164000000
H	5.507484000	0.041846000	1.592115000
H	3.741641000	-2.620779000	-1.292005000
H	5.622183000	-2.029565000	0.219426000

Methyl 2-diazo-3-oxobutanoate (1c)

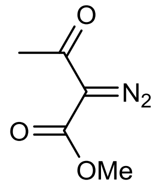

Sum of electronic and zero-point Energies= -529.162761
Sum of electronic and thermal Energies= -529.152055
Sum of electronic and thermal Enthalpies= -529.151111
Sum of electronic and thermal Free Energies= -529.199645

Standard orientation. Coordinates (Angstroms):

C	0.873207000	-0.456419000	-0.000858000
C	-0.418233000	0.241764000	0.000053000
C	-1.787765000	-0.342572000	0.000823000
O	1.006751000	-1.663860000	-0.000147000
O	-2.759599000	0.397639000	0.000294000
N	-0.395198000	1.566058000	0.000197000
N	-0.447639000	2.694099000	-0.000530000
O	1.905986000	0.421043000	-0.000076000
C	3.216201000	-0.172559000	0.000482000
H	3.912246000	0.666311000	0.001094000
H	3.356572000	-0.790400000	0.890967000
H	3.357529000	-0.789909000	-0.890192000
C	-1.908483000	-1.850029000	-0.000261000
H	-1.414990000	-2.284207000	0.874708000
H	-2.971279000	-2.099538000	-0.000493000
H	-1.414886000	-2.283036000	-0.875756000

Dimethyl diazomalonate (1d)

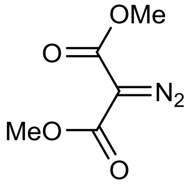

Sum of electronic and zero-point Energies= -604.379983
Sum of electronic and thermal Energies= -604.368204
Sum of electronic and thermal Enthalpies= -604.367260
Sum of electronic and thermal Free Energies= -604.418879

Standard orientation. Coordinates (Angstroms):

C	-1.453869000	0.286587000	0.000274000
C	0.014675000	0.450191000	0.000038000
C	1.067521000	-0.582777000	-0.000112000
O	-2.220375000	1.232333000	0.000051000
O	0.880477000	-1.777670000	0.000076000
N	0.402305000	1.715514000	-0.000044000
N	0.723712000	2.798790000	-0.000061000
O	-1.820805000	-1.001157000	0.000032000
C	-3.241127000	-1.227443000	-0.000148000
H	-3.356148000	-2.311168000	-0.000333000
H	-3.699471000	-0.789385000	0.890221000
H	-3.699297000	-0.789101000	-0.890467000
O	2.295525000	-0.000828000	-0.000067000
C	3.403417000	-0.916753000	0.000022000
H	3.377473000	-1.550235000	-0.890282000

H	4.295663000	-0.290317000	0.000047000
H	3.377384000	-1.550176000	0.890366000

2-Diazocyclohexane-1,3-dione (1e)

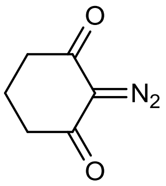

Sum of electronic and zero-point Energies= -492.041071
Sum of electronic and thermal Energies= -492.032362
Sum of electronic and thermal Enthalpies= -492.031418
Sum of electronic and thermal Free Energies= -492.075157

Standard orientation. Coordinates (Angstroms):

C	0.084222000	1.323358000	-0.067158000
C	-0.569148000	-0.000001000	-0.030079000
C	0.084224000	-1.323359000	-0.067158000
N	-1.892458000	-0.000001000	0.049054000
N	-3.018156000	-0.000001000	0.113563000
O	-0.547594000	-2.367236000	-0.027232000
O	-0.547597000	2.367235000	-0.027231000
C	1.602355000	-1.270605000	-0.179327000
H	1.850044000	-1.329407000	-1.250119000
H	1.993568000	-2.180757000	0.285184000
C	2.212695000	0.000001000	0.429430000
H	3.296782000	0.000002000	0.273704000
H	2.051613000	0.000002000	1.515285000
C	1.602354000	1.270606000	-0.179328000
H	1.993566000	2.180760000	0.285181000
H	1.850041000	1.329407000	-1.250121000

2-Diazocyclopentane-1,3-dione (1f)

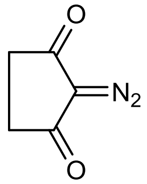

Sum of electronic and zero-point Energies= -452.754114
Sum of electronic and thermal Energies= -452.746334
Sum of electronic and thermal Enthalpies= -452.745389
Sum of electronic and thermal Free Energies= -452.787483

Standard orientation. Coordinates (Angstroms):

C	-0.363765000	-1.229773000	-0.000377000
C	0.445669000	-0.000021000	-0.000123000
C	-0.363681000	1.229789000	-0.000335000
N	1.756096000	-0.000059000	0.000135000
N	2.887497000	-0.000069000	0.000270000
O	0.027699000	2.378414000	-0.000009000
O	0.027532000	-2.378426000	0.000071000
C	-1.830614000	0.769448000	0.000102000
H	-2.322394000	1.199368000	-0.878885000
H	-2.321851000	1.199268000	0.879446000
C	-1.830667000	-0.769328000	0.000039000
H	-2.322001000	-1.199203000	0.879299000
H	-2.322407000	-1.199125000	-0.879032000

Benzene (PCM), 6-31G(d), PBE1PBE

3-Diazopentane-2,4-dione (1a)

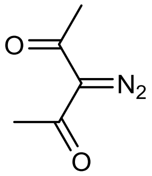

Sum of electronic and zero-point Energies= -453.942517
Sum of electronic and thermal Energies= -453.932838
Sum of electronic and thermal Enthalpies= -453.931894
Sum of electronic and thermal Free Energies= -453.977682

Standard orientation. Coordinates (Angstroms):

C	1.959592000	-1.441500000	-0.000039000
H	1.594221000	-1.987104000	-0.875140000
H	1.594354000	-1.987207000	0.875053000
H	3.050928000	-1.407991000	-0.000120000
C	1.452214000	-0.019126000	0.000086000
C	-0.025068000	0.195883000	0.000114000
C	-1.114134000	-0.802427000	0.000040000
C	-2.543793000	-0.288603000	0.000011000
H	-2.743520000	0.327546000	-0.885003000
H	-3.219443000	-1.145217000	-0.000085000
H	-2.743601000	0.327410000	0.885101000
O	2.203536000	0.946529000	0.000022000
O	-0.862081000	-1.999661000	-0.000063000
N	-0.360731000	1.476879000	0.000127000
N	-0.587476000	2.584872000	-0.000235000

2-Diazo-1,3-diphenylpropane-1,3-dione (1b)

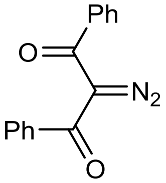

Sum of electronic and zero-point Energies= -837.301893
Sum of electronic and thermal Energies= -837.286275
Sum of electronic and thermal Enthalpies= -837.285331
Sum of electronic and thermal Free Energies= -837.347305

Standard orientation. Coordinates (Angstroms):

C	-0.726560000	-0.464183000	0.003186000
C	-0.043311000	0.812813000	-0.314023000
C	1.370553000	1.249716000	-0.059368000
O	-0.091299000	-1.499130000	0.169504000
O	1.592554000	2.450853000	0.068103000
N	-0.782761000	1.819670000	-0.760475000
N	-1.338690000	2.707817000	-1.185914000
C	-2.223879000	-0.477411000	0.118171000
C	-2.901901000	-1.603822000	-0.373390000
C	-2.949422000	0.534803000	0.763605000
C	-4.286269000	-1.696512000	-0.258127000
H	-2.329919000	-2.396845000	-0.844483000
C	-4.334341000	0.429716000	0.895536000
H	-2.437382000	1.389535000	1.195762000
C	-5.005153000	-0.678943000	0.376121000
H	-4.805141000	-2.564029000	-0.655948000
H	-4.886822000	1.211368000	1.408943000
H	-6.084687000	-0.754659000	0.472426000
C	2.479975000	0.258614000	0.028663000
C	3.559721000	0.578966000	0.868268000
C	2.544344000	-0.890590000	-0.771595000
C	4.671040000	-0.256252000	0.932673000
H	3.510325000	1.485133000	1.463262000
C	3.671570000	-1.709619000	-0.725473000
H	1.723145000	-1.139078000	-1.432117000
C	4.729378000	-1.401217000	0.132389000

H	5.494419000	-0.012166000	1.597992000
H	3.721473000	-2.592016000	-1.357035000
H	5.600224000	-2.049920000	0.175262000

<u>Methyl 2-diazo-3-oxobutanoate (1c)</u>

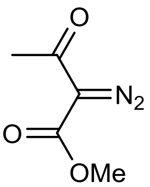

Sum of electronic and zero-point Energies= -529.166311
Sum of electronic and thermal Energies= -529.155616
Sum of electronic and thermal Enthalpies= -529.154671
Sum of electronic and thermal Free Energies= -529.203170

Standard orientation. Coordinates (Angstroms):

C	0.872772000	-0.459107000	-0.000423000
C	-0.419032000	0.240638000	-0.000359000
C	-1.786767000	-0.339342000	-0.000219000
O	1.001984000	-1.668130000	-0.000109000
O	-2.759281000	0.403884000	0.000249000
N	-0.392221000	1.565312000	-0.000256000
N	-0.437759000	2.693164000	0.000482000
O	1.903534000	0.415424000	-0.000065000
C	3.218588000	-0.173184000	0.000301000
H	3.909951000	0.668994000	0.000536000
H	3.360999000	-0.788266000	0.891934000
H	3.361519000	-0.788196000	-0.891296000
C	-1.913826000	-1.845408000	0.000072000
H	-1.422653000	-2.279844000	0.876140000
H	-2.977047000	-2.092915000	0.000448000
H	-1.423207000	-2.280110000	-0.876172000

<u>Dimethyl diazomalonate (1d)</u>

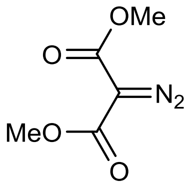

Sum of electronic and zero-point Energies= -604.384373
Sum of electronic and thermal Energies= -604.372565
Sum of electronic and thermal Enthalpies= -604.371620
Sum of electronic and thermal Free Energies= -604.423356

Standard orientation. Coordinates (Angstroms):

C	-1.452913000	0.287509000	-0.000008000
C	0.014836000	0.448671000	0.000088000
C	1.067946000	-0.583980000	0.000311000
O	-2.217887000	1.236464000	-0.000261000
O	0.876954000	-1.780321000	0.000253000
N	0.404751000	1.713693000	-0.000075000
N	0.728444000	2.795775000	-0.000152000
O	-1.824095000	-0.997605000	0.000064000
C	-3.246775000	-1.225295000	-0.000107000
H	-3.360840000	-2.308805000	-0.000018000
H	-3.703791000	-0.788648000	0.891165000
H	-3.703551000	-0.788819000	-0.891586000
O	2.292873000	-0.004807000	0.000048000
C	3.407966000	-0.915662000	-0.000049000
H	3.385445000	-1.547067000	-0.891488000
H	4.295509000	-0.283351000	-0.000258000
H	3.385738000	-1.546891000	0.891521000

2-Diazocyclohexane-1,3-dione (1e)

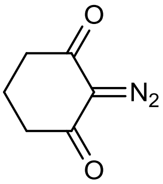

Sum of electronic and zero-point Energies= -492.045501
Sum of electronic and thermal Energies= -492.036775
Sum of electronic and thermal Enthalpies= -492.035831
Sum of electronic and thermal Free Energies= -492.079633

Standard orientation. Coordinates (Angstroms):

C	0.085594000	1.321868000	-0.066727000
C	-0.567983000	0.000000000	-0.028155000
C	0.085584000	-1.321873000	-0.066624000
N	-1.891664000	0.000009000	0.049696000
N	-3.017081000	0.000020000	0.113275000
O	-0.549716000	-2.366201000	-0.028373000
O	-0.549686000	2.366202000	-0.028327000
C	1.601840000	-1.270739000	-0.178853000
H	1.848139000	-1.330769000	-1.249792000
H	1.994658000	-2.179520000	0.286889000
C	2.213023000	-0.000009000	0.428560000
H	3.295942000	-0.000018000	0.268000000
H	2.055330000	-0.000006000	1.514582000
C	1.601860000	1.270727000	-0.178865000
H	1.994662000	2.179502000	0.286903000
H	1.848206000	1.330768000	-1.249791000

2-Diazocyclopentane-1,3-dione (1f)

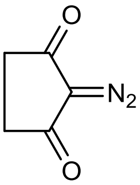

Sum of electronic and zero-point Energies= -452.758675
Sum of electronic and thermal Energies= -452.750822
Sum of electronic and thermal Enthalpies= -452.749878
Sum of electronic and thermal Free Energies= -452.792806

Standard orientation. Coordinates (Angstroms):

C	-0.364933000	-1.227781000	-0.000343000
C	0.444541000	-0.000024000	-0.000114000
C	-0.364838000	1.227799000	-0.000317000
N	1.755760000	-0.000066000	0.000113000
N	2.886447000	-0.000079000	0.000198000
O	0.028978000	2.377968000	-0.000034000
O	0.028789000	-2.377982000	0.000088000
C	-1.829902000	0.769388000	0.000155000
H	-2.322023000	1.197767000	-0.879255000
H	-2.321401000	1.197589000	0.880004000
C	-1.829962000	-0.769253000	0.000032000
H	-2.321643000	-1.197583000	0.879714000
H	-2.321966000	-1.197427000	-0.879545000

21. Thioketones

Benzene (PCM), 6-31G(d), PBE1PBE

Thiobenzophenone (2a)

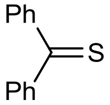

Sum of electronic and zero-point Energies= -899.401368
Sum of electronic and thermal Energies= -899.390329
Sum of electronic and thermal Enthalpies= -899.389385
Sum of electronic and thermal Free Energies= -899.439547

Standard orientation. Coordinates (Angstroms):

S	0.000000000	2.586460000	0.000000000
C	-1.271093000	0.161263000	-0.028650000
C	-1.366163000	-1.049956000	-0.743699000
C	-2.424745000	0.653546000	0.612031000
C	-2.577562000	-1.734234000	-0.826492000
H	-0.494673000	-1.436912000	-1.261102000
C	-3.625791000	-0.044928000	0.548804000
H	-2.358655000	1.583069000	1.167401000
C	-3.708562000	-1.238564000	-0.175356000
H	-2.636882000	-2.654840000	-1.400245000
H	-4.500659000	0.341178000	1.064300000
H	-4.649954000	-1.778327000	-0.230561000
C	1.271093000	0.161263000	0.028649000
C	1.366161000	-1.049956000	0.743697000
C	2.424746000	0.653546000	-0.612030000
C	2.577560000	-1.734235000	0.826491000
H	0.494670000	-1.436912000	1.261099000
C	3.625792000	-0.044929000	-0.548801000
H	2.358658000	1.583070000	-1.167400000
C	3.708561000	-1.238565000	0.175358000
H	2.636879000	-2.654842000	1.400244000
H	4.500661000	0.341178000	-1.064296000
H	4.649954000	-1.778328000	0.230564000
C	0.000000000	0.923800000	-0.000002000

Gas phase, 6-31G(d), PBE1PBE

2,2,4,4-Tetramethylcyclobutane-1-one-3-thione (2b)

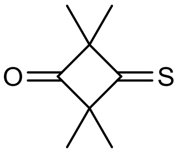

Sum of electronic and zero-point Energies= -785.274959
Sum of electronic and thermal Energies= -785.262933
Sum of electronic and thermal Enthalpies= -785.261988
Sum of electronic and thermal Free Energies= -785.312528

Standard orientation. Coordinates (Angstroms):

C	0.000000000	-0.756543000	-0.000155000
C	1.125962000	0.291285000	-0.000012000
C	-1.125962000	0.291284000	-0.000033000
C	0.000000000	1.356054000	-0.000054000
C	1.994719000	0.290686000	1.269193000
H	1.388779000	0.302228000	2.181060000
H	2.621938000	-0.606772000	1.288385000
H	2.639326000	1.175988000	1.275835000
C	1.994954000	0.290752000	-1.269054000
H	1.389184000	0.302343000	-2.181033000
H	2.639555000	1.176060000	-1.275520000
H	2.622184000	-0.606700000	-1.288184000

C	-1.994770000	0.290681000	1.269135000
H	-2.639378000	1.175983000	1.275754000
H	-2.621990000	-0.606777000	1.288298000
H	-1.388867000	0.302221000	2.181025000
C	-1.994903000	0.290756000	-1.269113000
H	-2.622131000	-0.606697000	-1.288271000
H	-2.639503000	1.176063000	-1.275602000
H	-1.389097000	0.302350000	-2.181067000
O	0.000000000	2.560361000	-0.000364000
S	0.000000000	-2.377432000	0.000174000

22. Transition states for cycloadditions of diazo compounds 1 to thiobenzophenone (2a) Benzene (PCM), 6-31G(d), PBE1PBE

$\underline{\boldsymbol{TS}_{1a+2a\rightarrow 6a}}$

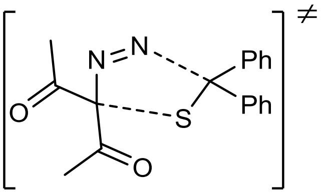

Imaginary Freq.: -375.06 cm^{-1}
Sum of electronic and zero-point Energies= -1353.307368
Sum of electronic and thermal Energies= -1353.285909
Sum of electronic and thermal Enthalpies= -1353.284965
Sum of electronic and thermal Free Energies= -1353.358894

Standard orientation. Coordinates (Angstroms):

N	1.369525000	0.027853000	1.409054000
N	0.215336000	0.000000000	1.565117000
C	-0.686994000	-0.042156000	-0.360542000
S	0.731233000	-0.116127000	-1.336768000
C	2.268245000	-0.005636000	0.375156000
C	2.968567000	-1.356419000	0.280874000
C	3.969480000	-1.563371000	-0.826125000
C	2.502167000	2.535783000	0.830625000
C	2.979945000	1.304326000	0.094691000
O	3.875198000	1.332553000	-0.728929000
O	2.669785000	-2.230139000	1.076353000
C	-1.402162000	1.269123000	-0.195423000
C	-1.193352000	2.351395000	-1.067053000
C	-1.853519000	3.564841000	-0.873344000
C	-2.734738000	3.726704000	0.197381000
C	-2.952918000	2.660484000	1.072531000
C	-2.297568000	1.446083000	0.877987000
C	-1.483630000	-1.307713000	-0.207525000
C	-2.850444000	-1.330426000	-0.533096000
C	-3.585704000	-2.513914000	-0.450911000
C	-2.974478000	-3.694902000	-0.030156000
C	-1.616610000	-3.684073000	0.299742000
C	-0.877979000	-2.507100000	0.206176000
H	3.562897000	-1.255050000	-1.793558000
H	4.858858000	-0.950545000	-0.650768000
H	4.237456000	-2.621480000	-0.846015000
H	2.601446000	2.407229000	1.914354000
H	3.099401000	3.388956000	0.505288000
H	-0.516202000	2.233958000	-1.906414000
H	-1.682452000	4.383010000	-1.567794000
H	-3.248471000	4.672417000	0.346598000
H	-3.633281000	2.773283000	1.912302000
H	-2.470693000	0.627222000	1.568335000

H	-3.338721000	-0.421450000	-0.868112000
H	-4.638767000	-2.508458000	-0.719430000
H	-3.548372000	-4.614835000	0.040458000
H	-1.128261000	-4.595898000	0.632654000
H	0.176649000	-2.519708000	0.462378000
H	1.445037000	2.726926000	0.613491000

$TS_{1b+2a\rightarrow 6b}$

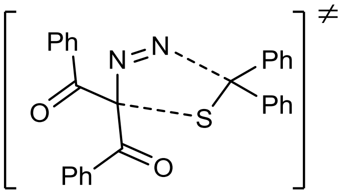

Imaginary Freq.: -362.26 cm^{-1}
Sum of electronic and zero-point Energies= -1736.665545
Sum of electronic and thermal Energies= -1736.638159
Sum of electronic and thermal Enthalpies= -1736.637215
Sum of electronic and thermal Free Energies= -1736.726180

Standard orientation. Coordinates (Angstroms):

N	-0.023805000	0.403024000	-1.451475000
N	0.901861000	-0.298768000	-1.527897000
C	1.150166000	-1.255485000	0.346071000
S	-0.206248000	-0.586065000	1.173534000
C	-1.011387000	0.716753000	-0.546009000
C	-2.312611000	-0.005408000	-0.942663000
C	-1.087061000	2.198298000	-0.173108000
O	-2.143669000	2.636674000	0.255171000
O	-2.325607000	-0.570818000	-2.028820000
C	2.525916000	-0.722767000	0.635430000
C	2.832309000	-0.062432000	1.837172000
C	4.115321000	0.430314000	2.077090000
C	5.120642000	0.276171000	1.120566000
C	4.832729000	-0.382387000	-0.077440000
C	3.553206000	-0.880754000	-0.315613000
C	1.047625000	-2.682244000	-0.119817000
C	2.027847000	-3.619298000	0.250274000
C	1.919154000	-4.957991000	-0.128745000
C	0.836788000	-5.386911000	-0.896720000
C	-0.141540000	-4.464258000	-1.275719000
C	-0.041368000	-3.130376000	-0.887575000
H	2.058200000	0.060330000	2.587409000
H	4.328780000	0.930095000	3.018100000
H	6.119805000	0.659545000	1.308706000
H	5.605700000	-0.508806000	-0.830671000
H	3.340729000	-1.389791000	-1.249833000
H	2.873161000	-3.304023000	0.852502000
H	2.684529000	-5.664700000	0.180992000
H	0.755223000	-6.427723000	-1.197920000
H	-0.988006000	-4.782228000	-1.878331000
H	-0.813794000	-2.432736000	-1.192749000
C	-3.487092000	-0.068046000	-0.027134000
C	-4.667016000	-0.601897000	-0.576158000
C	-3.475881000	0.311795000	1.324599000
C	-5.810852000	-0.741492000	0.202935000
H	-4.665008000	-0.901744000	-1.618203000
C	-4.618633000	0.157617000	2.105721000
H	-2.586144000	0.732148000	1.774274000
C	-5.788141000	-0.364106000	1.548237000
H	-6.717968000	-1.146538000	-0.236412000
H	-4.595538000	0.448987000	3.151660000
H	-6.678877000	-0.476154000	2.160479000
C	0.076919000	3.109074000	-0.388900000
C	1.415989000	2.736486000	-0.200784000

C	-0.226074000	4.436143000	-0.741705000
C	2.433153000	3.674403000	-0.375323000
H	1.672700000	1.736344000	0.123514000
C	0.793534000	5.362586000	-0.935940000
H	-1.265358000	4.721494000	-0.866640000
C	2.126630000	4.982845000	-0.752990000
H	3.464536000	3.377668000	-0.209486000
H	0.550415000	6.381038000	-1.224961000
H	2.923085000	5.707413000	-0.898687000

TS$_{1c+2a\rightarrow 6c}$

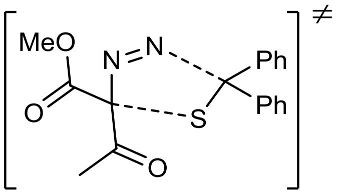

Imaginary Freq.: -376.45 cm^{-1}
Sum of electronic and zero-point Energies= -1428.529897
Sum of electronic and thermal Energies= -1428.507385
Sum of electronic and thermal Enthalpies= -1428.506440
Sum of electronic and thermal Free Energies= -1428.583374

Standard orientation. Coordinates (Angstroms):

N	-1.113068000	-0.563428000	-1.379892000
N	-0.029006000	-0.174988000	-1.546063000
C	0.852187000	0.101018000	0.368665000
S	-0.421238000	-0.501972000	1.360921000
C	-1.920097000	-0.923251000	-0.332199000
C	-2.106723000	-2.430369000	-0.233578000
C	-3.040287000	0.035552000	-0.036487000
O	-3.889013000	-0.172722000	0.804002000
O	-1.506098000	-3.148224000	-1.013608000
C	1.032499000	1.585089000	0.220848000
C	0.466177000	2.501250000	1.122243000
C	0.634593000	3.874486000	0.948385000
C	1.367776000	4.365073000	-0.133743000
C	1.936054000	3.467040000	-1.039684000
C	1.774098000	2.094183000	-0.863339000
C	2.054855000	-0.782067000	0.184009000
C	3.341646000	-0.303092000	0.482753000
C	4.458182000	-1.132953000	0.369137000
C	4.314024000	-2.453193000	-0.056827000
C	3.039750000	-2.939998000	-0.360351000
C	1.922859000	-2.117488000	-0.235627000
H	-0.102179000	2.130306000	1.968509000
H	0.195487000	4.562191000	1.666342000
H	1.497962000	5.435479000	-0.268057000
H	2.506074000	3.834441000	-1.888808000
H	2.216171000	1.406697000	-1.576841000
H	3.469470000	0.719661000	0.821074000
H	5.441597000	-0.742437000	0.617233000
H	5.183929000	-3.097331000	-0.151927000
H	2.912909000	-3.965325000	-0.697134000
H	0.940711000	-2.514934000	-0.471624000
O	-2.944035000	1.143647000	-0.780594000
C	-3.924383000	2.167898000	-0.507658000
H	-3.699637000	2.971012000	-1.207936000
H	-4.932022000	1.779856000	-0.671632000
H	-3.827199000	2.514113000	0.523655000
C	-2.989605000	-2.965067000	0.864466000

H	-2.728439000	-2.528458000	1.832932000
H	-4.034313000	-2.700968000	0.672818000
H	-2.874641000	-4.050101000	0.893869000

$TS_{1d+2a\rightarrow 6d}$

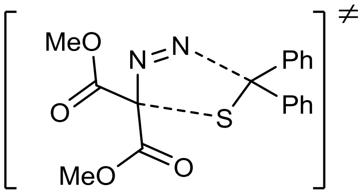

Imaginary Freq.: -371.67 cm^{-1}
Sum of electronic and zero-point Energies= -1503.749790
Sum of electronic and thermal Energies= -1503.726269
Sum of electronic and thermal Enthalpies= -1503.725325
Sum of electronic and thermal Free Energies= -1503.804945

Standard orientation. Coordinates (Angstroms):

N	1.027481000	0.040574000	1.441192000
N	-0.123196000	-0.013621000	1.592889000
C	-1.005557000	-0.147493000	-0.379352000
S	0.435554000	-0.226407000	-1.314394000
C	1.932513000	0.033292000	0.411309000
C	2.720105000	-1.252078000	0.377871000
C	2.566157000	1.372052000	0.116207000
O	3.447984000	1.539054000	-0.692635000
O	2.496125000	-2.174447000	1.136615000
C	-1.752462000	1.150348000	-0.275183000
C	-1.567612000	2.192496000	-1.198862000
C	-2.258930000	3.396351000	-1.068931000
C	-3.147217000	3.590254000	-0.009319000
C	-3.341047000	2.564612000	0.918387000
C	-2.655678000	1.358459000	0.785803000
C	-1.764026000	-1.427190000	-0.171628000
C	-3.136314000	-1.499658000	-0.466261000
C	-3.835923000	-2.699628000	-0.328544000
C	-3.183233000	-3.848421000	0.117804000
C	-1.819184000	-3.788820000	0.416581000
C	-1.116473000	-2.596055000	0.267542000
H	-0.882203000	2.050398000	-2.027621000
H	-2.106112000	4.182713000	-1.803444000
H	-3.685040000	4.529122000	0.090971000
H	-4.025804000	2.702874000	1.750832000
H	-2.810088000	0.571708000	1.516811000
H	-3.657171000	-0.616840000	-0.821199000
H	-4.894111000	-2.732211000	-0.574177000
H	-3.729311000	-4.780874000	0.231879000
H	-1.298360000	-4.675215000	0.768646000
H	-0.055804000	-2.570921000	0.497269000
O	1.975650000	2.338525000	0.834877000
C	2.428759000	3.680990000	0.561939000
H	1.856322000	4.317849000	1.234895000
H	3.499010000	3.767057000	0.762304000
H	2.228717000	3.939824000	-0.480238000
O	3.612307000	-1.257315000	-0.608172000
C	4.341941000	-2.491866000	-0.779584000
H	5.011045000	-2.310966000	-1.619408000
H	4.907518000	-2.724778000	0.125233000
H	3.651096000	-3.308301000	-1.001161000

TS$_{1e+2a\rightarrow 6e}$

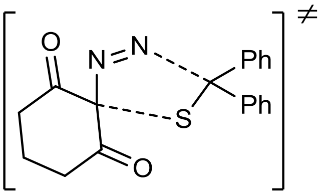

Imaginary Freq.: -380.49 cm^{-1}
Sum of electronic and zero-point Energies= -1391.406968
Sum of electronic and thermal Energies= -1391.386516
Sum of electronic and thermal Enthalpies= -1391.385572
Sum of electronic and thermal Free Energies= -1391.457424

Standard orientation. Coordinates (Angstroms):

N	1.076915000	-0.012195000	1.539670000
N	-0.085237000	-0.025119000	1.621314000
C	-0.881239000	-0.015455000	-0.314483000
S	0.572068000	-0.099431000	-1.243926000
C	2.016845000	-0.018850000	0.545186000
C	2.771392000	-1.321319000	0.403249000
C	3.862345000	-1.297553000	-0.650448000
C	2.742950000	1.295995000	0.352603000
O	2.465936000	2.276215000	1.014127000
O	2.488933000	-2.295050000	1.076279000
C	-1.592787000	1.300392000	-0.172997000
C	-1.339550000	2.385929000	-1.026521000
C	-2.001866000	3.600797000	-0.855094000
C	-2.928429000	3.760673000	0.176687000
C	-3.189973000	2.690821000	1.035105000
C	-2.533128000	1.474279000	0.861337000
C	-1.696531000	-1.277266000	-0.225553000
C	-3.047748000	-1.279463000	-0.610072000
C	-3.796482000	-2.457160000	-0.584533000
C	-3.214768000	-3.652633000	-0.163005000
C	-1.872376000	-3.662201000	0.225111000
C	-1.119898000	-2.490800000	0.188493000
H	3.377379000	-1.388465000	-1.633139000
H	4.479290000	-2.188337000	-0.505879000
H	-0.621967000	2.272336000	-1.831936000
H	-1.793589000	4.423662000	-1.533629000
H	-3.442161000	4.708769000	0.310206000
H	-3.904639000	2.802604000	1.846008000
H	-2.739891000	0.652433000	1.538797000
H	-3.512786000	-0.358553000	-0.945217000
H	-4.836976000	-2.435574000	-0.897602000
H	-3.799466000	-4.568102000	-0.136643000
H	-1.407083000	-4.585688000	0.558955000
H	-0.076458000	-2.519996000	0.486961000
C	3.815843000	1.255228000	-0.719402000
C	4.694464000	-0.003627000	-0.599070000
H	4.403366000	2.172950000	-0.633253000
H	3.316780000	1.268381000	-1.698725000
H	5.428199000	-0.012726000	-1.410979000
H	5.261827000	0.032364000	0.339205000

TS $_{1f+2a\rightarrow 6f}$

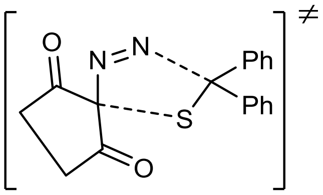

Imaginary Freq.: -409.03 cm^{-1}
Sum of electronic and zero-point Energies= -1352.116585
Sum of electronic and thermal Energies= -1352.097067
Sum of electronic and thermal Enthalpies= -1352.096123
Sum of electronic and thermal Free Energies= -1352.166263

Standard orientation. Coordinates (Angstroms):

N	-1.272659000	-0.045762000	1.513340000
N	-0.105531000	-0.004796000	1.601311000
C	0.693007000	0.015145000	-0.295429000
S	-0.741267000	0.043366000	-1.265857000
C	-2.193002000	-0.046719000	0.527229000
C	-3.049636000	1.169076000	0.285599000
C	-4.180526000	0.729425000	-0.646809000
C	-3.024688000	-1.269970000	0.231625000
O	-2.850925000	-2.383022000	0.669243000
O	-2.870446000	2.275832000	0.742546000
C	1.455029000	-1.274262000	-0.150826000
C	1.263674000	-2.359826000	-1.019739000
C	1.971287000	-3.548705000	-0.845895000
C	2.882191000	-3.681550000	0.203388000
C	3.082283000	-2.610976000	1.077261000
C	2.379778000	-1.420409000	0.901560000
C	1.460171000	1.308570000	-0.201984000
C	2.822825000	1.356925000	-0.538925000
C	3.525448000	2.562603000	-0.503267000
C	2.884805000	3.740265000	-0.119055000
C	1.529571000	3.704143000	0.219710000
C	0.823254000	2.504766000	0.172646000
H	-4.036140000	1.206791000	-1.621883000
H	-5.122446000	1.111077000	-0.240423000
H	0.558003000	-2.267884000	-1.838222000
H	1.810284000	-4.372234000	-1.536319000
H	3.431466000	-4.609281000	0.338457000
H	3.784434000	-2.701860000	1.901590000
H	2.539344000	-0.597986000	1.590880000
H	3.334148000	0.450645000	-0.844799000
H	4.576500000	2.576533000	-0.779221000
H	3.433528000	4.677470000	-0.084784000
H	1.017665000	4.613829000	0.521624000
H	-0.232165000	2.500347000	0.427514000
C	-4.133939000	-0.812398000	-0.719530000
H	-5.069629000	-1.291143000	-0.417373000
H	-3.895621000	-1.175353000	-1.725557000

23. Thiodiazolines 6 obtained from diazo compounds 1 and thiobenzophenone (2a)

Benzene (PCM), 6-31G(d), PBE1PBE

Thiadiazoline 6a

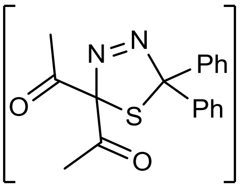

Sum of electronic and zero-point Energies= -1353.332756
Sum of electronic and thermal Energies= -1353.311620
Sum of electronic and thermal Enthalpies= -1353.310675
Sum of electronic and thermal Free Energies= -1353.384853

Standard orientation. Coordinates (Angstroms):

N	-1.348304000	-0.219779000	1.458449000
N	-0.130409000	-0.074687000	1.414818000
C	0.537216000	0.048751000	0.073595000
S	-0.854690000	-0.050648000	-1.186651000
C	-2.098992000	-0.228665000	0.186944000
C	-3.014366000	1.033272000	0.177628000
C	-4.027119000	1.194317000	-0.928643000
C	-2.247713000	-2.772632000	0.761455000
C	-2.822729000	-1.593448000	0.013232000
O	-3.782443000	-1.687381000	-0.724821000
O	-2.851942000	1.874622000	1.038902000
C	1.521692000	-1.127637000	0.010505000
C	1.556201000	-2.050395000	-1.040518000
C	2.487188000	-3.093304000	-1.037159000
C	3.385962000	-3.233027000	0.019109000
C	3.355868000	-2.316900000	1.073731000
C	2.436541000	-1.270082000	1.068360000
C	1.223156000	1.413825000	-0.011621000
C	2.411616000	1.572477000	-0.734796000
C	3.005965000	2.830206000	-0.850285000
C	2.427662000	3.939716000	-0.233006000
C	1.244018000	3.786323000	0.492566000
C	0.639994000	2.534373000	0.598443000
H	-3.606026000	0.933654000	-1.903869000
H	-4.869338000	0.516776000	-0.759437000
H	-4.374843000	2.229486000	-0.928867000
H	-2.333464000	-2.607281000	1.841333000
H	-2.793723000	-3.673755000	0.477150000
H	0.858719000	-1.960953000	-1.866076000
H	2.502494000	-3.796467000	-1.865286000
H	4.105423000	-4.047152000	0.021976000
H	4.050537000	-2.415273000	1.903312000
H	2.421097000	-0.559301000	1.887770000
H	2.875786000	0.714070000	-1.209429000
H	3.924982000	2.938086000	-1.419846000
H	2.894471000	4.917279000	-0.316851000
H	0.784888000	4.643908000	0.976469000
H	-0.290530000	2.434207000	1.148504000
H	-1.182326000	-2.896597000	0.536817000

Thiadiazoline 6b

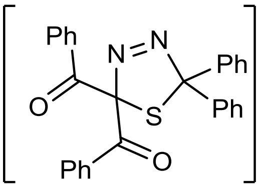

Sum of electronic and zero-point Energies= -1736.694815
Sum of electronic and thermal Energies= -1736.667800
Sum of electronic and thermal Enthalpies= -1736.666855
Sum of electronic and thermal Free Energies= -1736.755289

Standard orientation. Coordinates (Angstroms):

N	0.032777000	-0.008960000	-1.877551000
N	1.179733000	-0.348388000	-1.604170000
C	1.549466000	-0.621408000	-0.182312000
S	-0.011277000	-0.228991000	0.807482000
C	-0.948297000	0.084778000	-0.746571000
C	-1.973975000	-1.082952000	-0.936417000
C	-1.694482000	1.442462000	-0.952288000
O	-2.753937000	1.368175000	-1.554811000
O	-1.763976000	-1.911494000	-1.804465000
C	2.727221000	0.306157000	0.144398000
C	2.818581000	1.020890000	1.344488000
C	3.935360000	1.816656000	1.612956000
C	4.969835000	1.915630000	0.682613000
C	4.885654000	1.205485000	-0.517095000
C	3.778793000	0.400544000	-0.782472000
C	1.919706000	-2.102617000	-0.043291000
C	2.862196000	-2.512512000	0.908637000
C	3.167300000	-3.865026000	1.066339000
C	2.547308000	-4.823585000	0.264166000
C	1.612048000	-4.420312000	-0.690873000
C	1.294135000	-3.071026000	-0.842533000
H	2.016231000	0.966245000	2.072824000
H	3.989758000	2.360000000	2.552297000
H	5.835122000	2.539023000	0.890257000
H	5.684845000	1.274113000	-1.249909000
H	3.723782000	-0.154766000	-1.712381000
H	3.361618000	-1.776591000	1.530265000
H	3.896415000	-4.165530000	1.813896000
H	2.790886000	-5.875970000	0.381554000
H	1.123030000	-5.157146000	-1.322061000
H	0.552052000	-2.779138000	-1.577940000
C	-3.132553000	-1.213592000	0.001557000
C	-3.383529000	-0.343259000	1.073734000
C	-4.002908000	-2.294323000	-0.215261000
C	-4.477784000	-0.552301000	1.910705000
H	-2.732880000	0.502549000	1.271883000
C	-5.098932000	-2.499028000	0.617349000
H	-3.799045000	-2.962831000	-1.044519000
C	-5.338655000	-1.628335000	1.683745000
H	-4.658554000	0.126692000	2.738928000
H	-5.765745000	-3.337368000	0.436674000
H	-6.193086000	-1.787402000	2.335880000
C	-1.169226000	2.751872000	-0.482130000
C	0.191778000	3.019228000	-0.268409000
C	-2.107509000	3.787049000	-0.313451000
C	0.603112000	4.295337000	0.112904000
H	0.937988000	2.250437000	-0.416758000
C	-1.695392000	5.053629000	0.086656000
H	-3.155483000	3.576366000	-0.499067000
C	-0.337345000	5.310160000	0.300329000
H	1.660121000	4.492265000	0.265464000
H	-2.428845000	5.842536000	0.226580000
H	-0.013482000	6.300754000	0.607849000

Thiadiazoline 6c

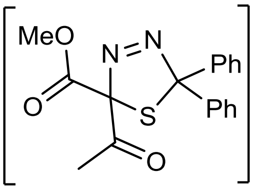

Sum of electronic and zero-point Energies= -1428.557323
Sum of electronic and thermal Energies= -1428.535145
Sum of electronic and thermal Enthalpies= -1428.534201
Sum of electronic and thermal Free Energies= -1428.610976

Standard orientation. Coordinates (Angstroms):

N	1.191669000	-0.396693000	1.316515000
N	0.007389000	-0.069291000	1.347926000
C	-0.731802000	0.083025000	0.053476000
S	0.572197000	-0.080579000	-1.283143000
C	1.811808000	-0.583406000	-0.029291000
C	2.270157000	-2.083950000	-0.131213000
C	3.250669000	-2.548035000	0.919837000
C	3.959316000	2.432468000	0.369309000
C	3.038692000	0.332983000	-0.167143000
O	4.001135000	0.044841000	-0.845907000
O	1.833941000	-2.801764000	-1.000170000
C	-1.349840000	1.476855000	-0.028904000
C	-2.442781000	1.710046000	-0.873704000
C	-2.973845000	2.993570000	-1.004670000
C	-2.428422000	4.056532000	-0.282890000
C	-1.341305000	3.829017000	0.563218000
C	-0.799156000	2.549550000	0.684620000
C	-1.776034000	-1.046523000	0.082770000
C	-2.834701000	-0.936699000	1.000362000
C	-3.780698000	-1.952731000	1.116176000
C	-3.685214000	-3.094535000	0.316491000
C	-2.631610000	-3.213456000	-0.588860000
C	-1.675768000	-2.200216000	-0.701469000
H	2.920746000	-2.240724000	1.917502000
H	3.348816000	-3.633812000	0.866317000
H	4.227280000	-2.086993000	0.734452000
H	3.678038000	3.271564000	1.004012000
H	4.902826000	1.993450000	0.700574000
H	4.046793000	2.746915000	-0.673045000
H	-2.881587000	0.886385000	-1.428583000
H	-3.818271000	3.159282000	-1.668037000
H	-2.847266000	5.054504000	-0.378635000
H	-0.910447000	4.649661000	1.130411000
H	0.055644000	2.386789000	1.333074000
H	-2.914738000	-0.052335000	1.624442000
H	-4.592726000	-1.851351000	1.830987000
H	-4.425444000	-3.885422000	0.402095000
H	-2.543780000	-4.099137000	-1.212075000
H	-0.849272000	-2.313899000	-1.393621000
O	2.887177000	1.474335000	0.508656000

Thiadiazoline 6d

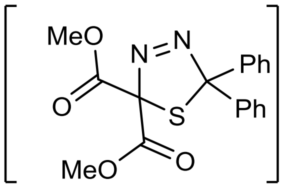

Sum of electronic and zero-point Energies= -1503.779418
Sum of electronic and thermal Energies= -1503.756204
Sum of electronic and thermal Enthalpies= -1503.755260
Sum of electronic and thermal Free Energies= -1503.835248

Standard orientation. Coordinates (Angstroms):

N	0.994719000	0.034056000	1.524618000
N	-0.225001000	-0.065131000	1.447881000
C	-0.871229000	-0.153338000	0.098227000
S	0.552397000	-0.236921000	-1.124686000
C	1.758809000	0.044516000	0.233586000
C	2.757001000	-1.123904000	0.262944000
C	2.501072000	1.399942000	0.212938000
O	3.685890000	1.512048000	0.429754000
O	2.803393000	-1.955141000	1.139803000
C	-1.743596000	1.105160000	-0.009050000
C	-1.626158000	2.035494000	-1.045577000
C	-2.457071000	3.158362000	-1.084956000
C	-3.403425000	3.371987000	-0.083423000
C	-3.520529000	2.450640000	0.960236000
C	-2.701761000	1.323726000	0.995376000
C	-1.670832000	-1.455696000	0.024054000
C	-2.861783000	-1.528468000	-0.707659000
C	-3.555254000	-2.736194000	-0.810543000
C	-3.074281000	-3.879369000	-0.172330000
C	-1.887052000	-3.812262000	0.561236000
C	-1.185254000	-2.611962000	0.653087000
H	-0.882859000	1.888326000	-1.820578000
H	-2.357835000	3.866748000	-1.903040000
H	-4.045244000	4.248238000	-0.114306000
H	-4.251815000	2.606819000	1.748503000
H	-2.801459000	0.608810000	1.805625000
H	-3.250964000	-0.642511000	-1.198584000
H	-4.475374000	-2.777991000	-1.386982000
H	-3.618602000	-4.816847000	-0.245995000
H	-1.502916000	-4.696929000	1.061424000
H	-0.253120000	-2.578413000	1.209181000
C	4.456824000	-2.199175000	-0.960133000
H	4.967266000	-2.031573000	-1.907624000
H	5.164409000	-2.168549000	-0.128784000
H	3.936231000	-3.159561000	-0.964742000
C	2.229398000	3.731518000	0.047838000
H	1.395755000	4.411893000	-0.119904000
H	2.685507000	3.908001000	1.024483000
H	2.984425000	3.841676000	-0.733966000
O	1.654500000	2.408411000	-0.000858000
O	3.507583000	-1.118535000	-0.842739000

Thiadiazoline 6e

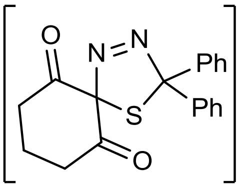

Sum of electronic and zero-point Energies= -1391.430913
Sum of electronic and thermal Energies= -1391.410831
Sum of electronic and thermal Enthalpies= -1391.409887
Sum of electronic and thermal Free Energies= -1391.481376

Standard orientation. Coordinates (Angstroms):

N	1.075424000	-0.041529000	1.572449000
N	-0.145077000	0.011886000	1.472571000
C	-0.766305000	0.030549000	0.107251000
S	0.683499000	0.059336000	-1.099765000
C	-1.574210000	1.321458000	-0.045314000
C	-2.780452000	1.336977000	-0.755431000
C	-3.486994000	2.529968000	-0.922017000
C	-3.003207000	3.715977000	-0.370218000
C	-1.800560000	3.705845000	0.341193000
C	-1.086182000	2.519779000	0.497896000
C	-1.621556000	-1.240279000	0.032221000
C	-2.543269000	-1.468688000	1.068372000
C	-3.351613000	-2.603441000	1.059490000
C	-3.262381000	-3.522877000	0.011589000
C	-2.355637000	-3.297998000	-1.023089000
C	-1.535627000	-2.166638000	-1.011387000
H	-3.172051000	0.418193000	-1.179091000
H	-4.419253000	2.526677000	-1.480217000
H	-3.557240000	4.642511000	-0.493737000
H	-1.414103000	4.624490000	0.773848000
H	-0.142254000	2.529875000	1.034368000
H	-2.619710000	-0.757045000	1.883533000
H	-4.051960000	-2.768197000	1.873656000
H	-3.894292000	-4.406718000	0.003592000
H	-2.276696000	-4.005056000	-1.844336000
H	-0.825666000	-2.012987000	-1.816407000
C	1.870277000	-0.055483000	0.330520000
C	2.778655000	1.199700000	0.304037000
C	2.642952000	-1.395095000	0.234776000
C	3.953331000	1.140032000	-0.651824000
C	3.803831000	-1.405639000	-0.741219000
C	4.723914000	-0.187906000	-0.535250000
H	4.592320000	2.004663000	-0.453849000
H	3.559079000	1.247135000	-1.673439000
H	4.340824000	-2.349334000	-0.614688000
H	3.394349000	-1.388136000	-1.761714000
H	5.526516000	-0.208293000	-1.279006000
H	5.202935000	-0.251178000	0.449621000
O	2.524810000	2.157330000	1.003913000
O	2.310063000	-2.348071000	0.904758000

Thiadiazoline 6f

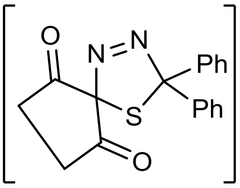

Sum of electronic and zero-point Energies= -1352.143972
Sum of electronic and thermal Energies= -1352.124719
Sum of electronic and thermal Enthalpies= -1352.123775
Sum of electronic and thermal Free Energies= -1352.194700

Standard orientation. Coordinates (Angstroms):

N	-1.412647000	-0.082430000	1.292814000
N	-0.184530000	-0.050811000	1.339849000
C	0.595065000	-0.016616000	0.070666000
S	-0.687840000	-0.258740000	-1.291447000
C	1.575941000	-1.185054000	0.017600000
C	2.638798000	-1.156494000	-0.895789000
C	3.501926000	-2.245698000	-1.010371000
C	3.322606000	-3.372071000	-0.204683000
C	2.269195000	-3.403588000	0.710091000
C	1.396101000	-2.320355000	0.817146000
C	1.284380000	1.359814000	0.076875000
C	2.415030000	1.532208000	0.892424000
C	3.037774000	2.775095000	0.988062000
C	2.539727000	3.866022000	0.271894000
C	1.408227000	3.704888000	-0.527255000
C	0.777064000	2.462140000	-0.619394000
H	2.791854000	-0.280023000	-1.518491000
H	4.317414000	-2.210504000	-1.727539000
H	3.998770000	-4.218292000	-0.289490000
H	2.120459000	-4.274741000	1.342126000
H	0.573358000	-2.362913000	1.523128000
H	2.808231000	0.689998000	1.452431000
H	3.912824000	2.889469000	1.621962000
H	3.027906000	4.834374000	0.341190000
H	1.006296000	4.548484000	-1.081663000
H	-0.116613000	2.355984000	-1.223492000
C	-2.023324000	-0.163361000	-0.087134000
C	-2.982313000	-1.392852000	-0.010574000
C	-3.008174000	1.037265000	-0.182442000
C	-4.387536000	-0.915788000	0.348131000
C	-4.330173000	0.620140000	0.447613000
H	-4.706367000	-1.402282000	1.275488000
H	-5.068334000	-1.258303000	-0.439948000
H	-4.304247000	0.947716000	1.495101000
H	-5.159992000	1.138028000	-0.039286000
O	-2.646342000	-2.533543000	-0.204022000
O	-2.753378000	2.103711000	-0.685249000

24. Transition states for decompositions of thiadiazolines 6

Benzene (PCM), 6-31G(d), PBE1PBE

TS$_{6a\rightarrow N\equiv N+7a}$

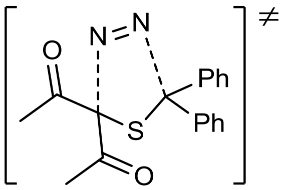

Imaginary Freq.: -376.88 cm^{-1}
Sum of electronic and zero-point Energies= -1353.307428
Sum of electronic and thermal Energies= -1353.286010
Sum of electronic and thermal Enthalpies= -1353.285066
Sum of electronic and thermal Free Energies= -1353.358724

Standard orientation. Coordinates (Angstroms):

S	-0.462534000	-1.053019000	-1.026919000
C	-1.960473000	-0.743150000	-0.144693000
C	-2.863756000	-1.952100000	-0.161949000
O	-2.580087000	-2.946623000	-0.812710000
C	-4.110886000	-1.903716000	0.698209000
H	-3.847989000	-1.715606000	1.745669000
H	-4.786351000	-1.103183000	0.374711000
H	-4.627167000	-2.861973000	0.617021000
C	-2.669295000	0.602059000	-0.300565000
O	-2.804519000	1.077525000	-1.414016000
C	-3.179574000	1.324385000	0.929733000
H	-3.942659000	2.046001000	0.629933000
H	-3.565259000	0.652208000	1.698285000
H	-2.338072000	1.871296000	1.374548000
C	0.702948000	-0.122051000	-0.048490000
N	-1.220395000	-0.680758000	1.693190000
N	-0.082675000	-0.454028000	1.684430000
C	2.047003000	-0.777479000	-0.047248000
C	2.169444000	-2.126448000	0.336795000
C	3.205697000	-0.078642000	-0.429036000
C	3.412089000	-2.753235000	0.343149000
H	1.284736000	-2.675866000	0.642173000
C	4.447667000	-0.712237000	-0.431585000
H	3.131377000	0.957825000	-0.739045000
C	4.555598000	-2.048792000	-0.043800000
H	3.488888000	-3.791791000	0.652070000
H	5.330908000	-0.160236000	-0.740409000
H	5.524916000	-2.539610000	-0.041277000
C	0.683417000	1.377457000	-0.031641000
C	1.257552000	2.058779000	1.059941000
C	0.152031000	2.129107000	-1.089687000
C	1.278584000	3.450627000	1.095760000
H	1.678528000	1.490080000	1.882876000
C	0.182481000	3.524280000	-1.053481000
H	-0.287161000	1.621712000	-1.940991000
C	0.741049000	4.189736000	0.037722000
H	1.717193000	3.958874000	1.950028000
H	-0.233283000	4.087684000	-1.883959000
H	0.760810000	5.275695000	0.064727000

TS$_{6b \rightarrow N \equiv N+7b}$

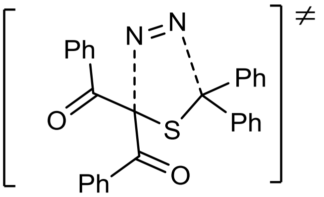

Imaginary Freq.: 374.62 cm^{-1}
Sum of electronic and zero-point Energies= -1736.673001
Sum of electronic and thermal Energies= -1736.645510
Sum of electronic and thermal Enthalpies= -1736.644566
Sum of electronic and thermal Free Energies= -1736.733537

Standard orientation. Coordinates (Angstroms):

N	0.565949000	0.354502000	2.142119000
N	0.142142000	1.387080000	1.828100000
C	-0.475859000	1.577795000	0.077531000
S	0.522820000	0.289117000	-0.688476000
C	0.527522000	-0.932218000	0.573237000
C	-0.757817000	-1.701433000	0.923635000
C	1.731664000	-1.812107000	0.690726000
O	1.566092000	-2.961081000	1.103139000
O	-1.287344000	-1.532585000	2.008755000
C	-0.070321000	2.934016000	-0.412914000
C	1.277127000	3.334909000	-0.340501000
C	1.670154000	4.594465000	-0.783262000
C	0.725939000	5.477061000	-1.314938000
C	-0.612933000	5.091472000	-1.396738000
C	-1.010101000	3.833158000	-0.946463000
C	-1.949956000	1.355148000	0.271641000
C	-2.693071000	0.534614000	-0.588753000
C	-4.069789000	0.383495000	-0.411603000
C	-4.723166000	1.048086000	0.625167000
C	-3.992312000	1.875017000	1.482922000
C	-2.620803000	2.034923000	1.304850000
H	2.015923000	2.656225000	0.072507000
H	2.713116000	4.889292000	-0.711009000
H	1.033206000	6.459677000	-1.662005000
H	-1.351930000	5.769632000	-1.813882000
H	-2.051845000	3.542265000	-1.018981000
H	-2.196222000	0.021319000	-1.404863000
H	-4.626645000	-0.258618000	-1.088036000
H	-5.793613000	0.926038000	0.764926000
H	-4.491116000	2.396391000	2.295063000
H	-2.059974000	2.677350000	1.975777000
C	-1.345882000	-2.653358000	-0.076008000
C	-0.727197000	-2.990366000	-1.289234000
C	-2.579093000	-3.244096000	0.246712000
C	-1.331897000	-3.893586000	-2.162778000
H	0.230153000	-2.557277000	-1.556367000
C	-3.183178000	-4.143598000	-0.626306000
H	-3.045705000	-2.981340000	1.189867000
C	-2.560712000	-4.470071000	-1.835075000
H	-0.841050000	-4.149374000	-3.097570000
H	-4.137620000	-4.592849000	-0.365902000
H	-3.030597000	-5.173391000	-2.517370000
C	3.112628000	-1.338093000	0.358590000
C	3.574348000	-0.041085000	0.629375000
C	4.005699000	-2.284946000	-0.169984000
C	4.899277000	0.303697000	0.362390000

H	2.912644000	0.685252000	1.085798000
C	5.320693000	-1.931369000	-0.459895000
H	3.651350000	-3.295205000	-0.348292000
C	5.770541000	-0.634886000	-0.193842000
H	5.251979000	1.305044000	0.593104000
H	5.997878000	-2.667492000	-0.884027000
H	6.799320000	-0.360414000	-0.410707000

$TS_{6c \rightarrow N\equiv N+7c}$

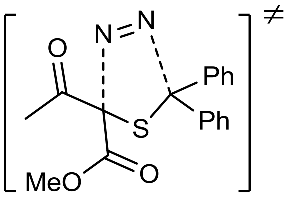

Imaginary Freq.: -376.62 cm^{-1}
Sum of electronic and zero-point Energies= -1428.534243
Sum of electronic and thermal Energies= -1428.511697
Sum of electronic and thermal Enthalpies= -1428.510752
Sum of electronic and thermal Free Energies= -1428.587771

Standard orientation. Coordinates (Angstroms):

S	-0.423287000	-0.808381000	-1.054716000
C	-1.836281000	-0.205445000	-0.202258000
C	-2.940348000	-1.217297000	-0.196150000
O	-2.830472000	-2.388441000	-0.501824000
C	-5.216330000	-1.567902000	0.331967000
H	-6.047886000	-0.958396000	0.684964000
H	-5.442701000	-2.001132000	-0.645087000
H	-4.997920000	-2.369859000	1.041297000
C	-2.276570000	1.248833000	-0.347378000
O	-2.187878000	1.803338000	-1.428607000
C	-2.788153000	1.973990000	0.878756000
H	-3.382901000	2.834085000	0.562896000
H	-3.366524000	1.322402000	1.534504000
H	-1.923264000	2.339197000	1.448008000
C	0.906660000	-0.178372000	-0.030350000
N	-1.093942000	-0.308045000	1.671849000
N	0.064636000	-0.342692000	1.652688000
C	2.067848000	-1.123626000	-0.045373000
C	1.876601000	-2.469632000	0.319956000
C	3.353922000	-0.703784000	-0.426435000
C	2.942188000	-3.364909000	0.311479000
H	0.889455000	-2.805301000	0.621468000
C	4.416844000	-1.605981000	-0.445369000
H	3.518365000	0.326344000	-0.722345000
C	4.215559000	-2.936386000	-0.074088000
H	2.779505000	-4.397528000	0.606543000
H	5.402257000	-1.267955000	-0.753286000
H	5.046147000	-3.636791000	-0.083808000
C	1.235989000	1.286232000	0.003484000
C	1.955886000	1.798134000	1.100932000
C	0.890901000	2.155271000	-1.040789000
C	2.301892000	3.145692000	1.155203000
H	2.234066000	1.135501000	1.914311000
C	1.246988000	3.503981000	-0.985821000
H	0.338263000	1.778165000	-1.893315000
C	1.949338000	4.004713000	0.110148000
H	2.848949000	3.525008000	2.013847000
H	0.970385000	4.161008000	-1.805430000

H	2.222282000	5.055551000	0.151438000
O	-4.097695000	-0.666409000	0.232958000

$TS_{6d\rightarrow N\equiv N+7d}$

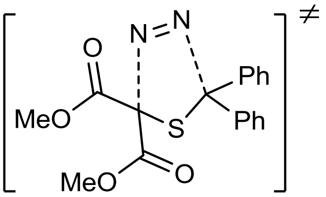

Imaginary Freq.: -375.63 cm^{-1}
Sum of electronic and zero-point Energies= -1503.758122
Sum of electronic and thermal Energies= -1503.734630
Sum of electronic and thermal Enthalpies= -1503.733686
Sum of electronic and thermal Free Energies= -1503.813042

Standard orientation. Coordinates (Angstroms):

S	-0.124754000	-1.046592000	-1.041006000
C	-1.615310000	-0.635685000	-0.217990000
C	-2.592530000	-1.766284000	-0.212762000
O	-2.330804000	-2.926107000	-0.466485000
C	-4.823244000	-2.357490000	0.276420000
H	-5.727149000	-1.837367000	0.592696000
H	-4.977724000	-2.848455000	-0.687557000
H	-4.528649000	-3.103372000	1.018669000
C	-2.229494000	0.737520000	-0.403224000
O	-2.383390000	1.259931000	-1.488812000
C	-3.243954000	2.577237000	0.651017000
H	-2.570446000	3.300653000	0.185414000
H	-4.157324000	2.495486000	0.056736000
H	-3.475365000	2.869183000	1.675048000
C	1.075556000	-0.177886000	-0.032012000
N	-0.888629000	-0.606273000	1.664119000
N	0.258749000	-0.446439000	1.653451000
C	2.381366000	-0.908760000	-0.022579000
C	2.421141000	-2.259757000	0.371242000
C	3.579472000	-0.281687000	-0.405963000
C	3.624663000	-2.958721000	0.386310000
H	1.504174000	-2.753043000	0.677642000
C	4.781692000	-0.987741000	-0.399988000
H	3.566894000	0.755335000	-0.722631000
C	4.808636000	-2.325474000	-0.002027000
H	3.639330000	-3.997697000	0.702749000
H	5.697190000	-0.491607000	-0.709565000
H	5.747249000	-2.872674000	0.007261000
C	1.142745000	1.321842000	-0.040374000
C	1.740592000	1.984735000	1.049066000
C	0.669907000	2.085758000	-1.116628000
C	1.842279000	3.373022000	1.065017000
H	2.114248000	1.405424000	1.887307000
C	0.779771000	3.477895000	-1.099409000
H	0.215554000	1.592742000	-1.968264000
C	1.362122000	4.126245000	-0.010548000
H	2.297926000	3.867673000	1.918306000
H	0.409512000	4.051286000	-1.944554000
H	1.445493000	5.209490000	0.000894000
O	-3.814869000	-1.336822000	0.163880000
O	-2.599458000	1.293346000	0.757612000

***TS*$_{6e→N≡N+7e}$**

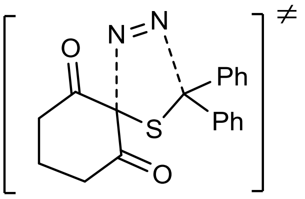

Imaginary Freq.: -348.35
Sum of electronic and zero-point Energies= -1391.414466
Sum of electronic and thermal Energies= -1391.393941
Sum of electronic and thermal Enthalpies= -1391.392996
Sum of electronic and thermal Free Energies= -1391.464924

Standard orientation. Coordinates (Angstroms):

N	-1.164619000	-0.384257000	1.618474000
N	-0.018404000	-0.314002000	1.515792000
C	0.808356000	-0.113020000	0.012295000
S	-0.426710000	-0.757853000	-1.176153000
C	1.992807000	-1.047869000	0.018804000
C	3.288365000	-0.593543000	-0.272445000
C	4.362995000	-1.483254000	-0.280983000
C	4.160926000	-2.831145000	0.016714000
C	2.875066000	-3.292768000	0.313808000
C	1.798123000	-2.411844000	0.305294000
C	1.157796000	1.357683000	0.044006000
C	1.737032000	1.902678000	1.202705000
C	2.134211000	3.237959000	1.234120000
C	1.980577000	4.041304000	0.101971000
C	1.421822000	3.500591000	-1.056477000
C	1.006444000	2.169242000	-1.086926000
H	3.455073000	0.451592000	-0.505878000
H	5.358072000	-1.119563000	-0.520543000
H	5.000239000	-3.521102000	0.016760000
H	2.710789000	-4.340687000	0.547077000
H	0.799030000	-2.776989000	0.522061000
H	1.871481000	1.280130000	2.082095000
H	2.568278000	3.647287000	2.142023000
H	2.294992000	5.081105000	0.123429000
H	1.297663000	4.116919000	-1.942287000
H	0.555309000	1.760706000	-1.982199000
C	-1.967023000	-0.407388000	-0.418040000
C	-2.871797000	-1.591871000	-0.315701000
C	-2.539255000	0.963053000	-0.416898000
C	-4.282961000	-1.338184000	0.207196000
C	-3.921122000	1.147674000	0.210417000
C	-4.419710000	-0.049968000	1.021689000
H	-4.581784000	-2.225072000	0.774982000
H	-4.941589000	-1.296456000	-0.674173000
H	-3.886141000	2.065846000	0.806835000
H	-4.609428000	1.352160000	-0.623167000
H	-5.466071000	0.102597000	1.308423000
H	-3.845833000	-0.137829000	1.950954000
O	-2.519647000	-2.723209000	-0.624587000
O	-1.956288000	1.925314000	-0.900052000

***TS**$_{6f\rightarrow N\equiv N+7f}$*

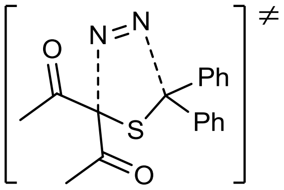

Imaginary Freq.: -340.57 cm^{-1}
Sum of electronic and zero-point Energies= -1352.132179
Sum of electronic and thermal Energies= -1352.112584
Sum of electronic and thermal Enthalpies= -1352.111640
Sum of electronic and thermal Free Energies= -1352.181943

Standard orientation. Coordinates (Angstroms):

N	-1.252981000	-0.457866000	1.677232000
N	-0.112963000	-0.347995000	1.535461000
C	0.650005000	-0.107188000	0.030758000
S	-0.586553000	-0.823546000	-1.128930000
C	1.889055000	-0.966694000	-0.010046000
C	3.123502000	-0.449212000	-0.430402000
C	4.247444000	-1.273495000	-0.496088000
C	4.155604000	-2.616010000	-0.128346000
C	2.930375000	-3.139578000	0.295640000
C	1.803197000	-2.325286000	0.343753000
C	0.898461000	1.383484000	0.045833000
C	1.579464000	1.943428000	1.142079000
C	1.863945000	3.305927000	1.175882000
C	1.490028000	4.125978000	0.107681000
C	0.824611000	3.574055000	-0.986378000
C	0.522975000	2.211742000	-1.017830000
H	3.203255000	0.592705000	-0.718504000
H	5.194306000	-0.862855000	-0.834553000
H	5.033612000	-3.254375000	-0.172580000
H	2.852294000	-4.183868000	0.583362000
H	0.850579000	-2.738946000	0.660339000
H	1.878642000	1.309039000	1.971201000
H	2.380151000	3.725691000	2.034594000
H	1.716110000	5.188409000	0.130651000
H	0.527782000	4.203593000	-1.820154000
H	-0.009073000	1.793661000	-1.862546000
C	-2.094796000	-0.515892000	-0.328455000
C	-3.034358000	-1.645959000	-0.121096000
C	-2.846721000	0.754923000	-0.263227000
C	-4.361146000	-1.073317000	0.389254000
C	-4.211244000	0.458683000	0.372129000
H	-4.560140000	-1.478922000	1.387152000
H	-5.165166000	-1.430315000	-0.263839000
H	-4.218249000	0.888678000	1.380868000
H	-4.990562000	0.972825000	-0.198537000
O	-2.807726000	-2.831673000	-0.294086000
O	-2.483459000	1.859037000	-0.634699000

25. Thiodiazolines 6 decomposition products

Benzene (PCM), 6-31G(d), PBE1PBE

<u>Nitrogen (N≡N)</u>

Sum of electronic and zero-point Energies: -109.394762
Sum of electronic and thermal Energies: -109.392401
Sum of electronic and thermal Enthalpies: -109.391457
Sum of electronic and thermal Free Energies: -109.413206

Standard orientation. Coordinates (Angstroms):

N	0.000000000	0.000000000	0.552700000
N	0.000000000	0.000000000	-0.552700000

<u>C=S-ylide 7a</u>

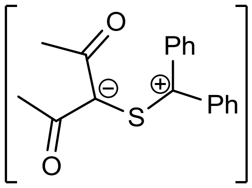

Sum of electronic and zero-point Energies= -1243.853423
Sum of electronic and thermal Energies= -1243.833776
Sum of electronic and thermal Enthalpies= -1243.832832
Sum of electronic and thermal Free Energies= -1243.903392

Standard orientation. Coordinates (Angstroms):

S	0.362711000	-1.457672000	-0.456360000
C	1.952732000	-0.958005000	0.100454000
C	2.956142000	-1.437840000	-0.824708000
O	2.607557000	-1.910495000	-1.923413000
C	4.446160000	-1.412157000	-0.506696000
H	4.970791000	-1.825256000	-1.370336000
H	4.809605000	-0.396521000	-0.321838000
H	4.685050000	-2.014698000	0.375762000
C	2.029536000	-0.333693000	1.410197000
O	1.018368000	-0.001753000	2.046786000
C	3.387878000	-0.063011000	2.055434000
H	3.195716000	0.382155000	3.033489000
H	3.978378000	-0.974411000	2.188273000
H	3.983828000	0.636570000	1.459693000
C	-0.723816000	-0.189380000	-0.210375000
C	-2.129996000	-0.566918000	-0.066577000
C	-3.145364000	0.259609000	-0.601422000
C	-2.506914000	-1.774250000	0.562810000
C	-4.479687000	-0.122445000	-0.527636000
H	-2.873555000	1.182807000	-1.101196000
C	-3.845229000	-2.147339000	0.635968000
H	-1.746214000	-2.390294000	1.029745000
C	-4.834781000	-1.326229000	0.089875000
H	-5.245909000	0.516608000	-0.956705000
H	-4.118120000	-3.072002000	1.135892000
H	-5.879497000	-1.617144000	0.153304000
C	-0.333271000	1.224829000	-0.238217000
C	-0.896599000	2.135918000	0.674818000
C	0.577642000	1.698787000	-1.202741000
C	-0.556337000	3.485797000	0.624846000
H	-1.577578000	1.773209000	1.437161000
C	0.898740000	3.050637000	-1.259727000
H	1.009097000	1.005689000	-1.917841000
C	0.337456000	3.946994000	-0.343587000
H	-0.988129000	4.176386000	1.343388000
H	1.584313000	3.408382000	-2.022539000
H	0.595706000	5.001421000	-0.387771000

C=S-ylide 7b

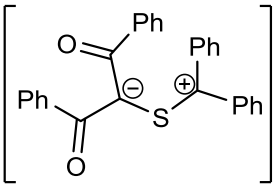

Sum of electronic and zero-point Energies= -1627.213679
Sum of electronic and thermal Energies= -1627.187830
Sum of electronic and thermal Enthalpies= -1627.186886
Sum of electronic and thermal Free Energies= -1627.273698

Standard orientation. Coordinates (Angstroms):

C	-1.528639000	0.641528000	-0.663350000
S	-0.252736000	-0.083607000	-1.503613000
C	1.161738000	-0.098929000	-0.464420000
C	2.301785000	0.325781000	-1.248718000
C	1.056488000	-0.582737000	0.910625000
O	1.772944000	-0.170532000	1.830902000
O	2.089510000	0.752924000	-2.407505000
C	-1.355603000	1.604243000	0.432970000
C	-0.354650000	2.596628000	0.378406000
C	-0.245087000	3.538487000	1.393511000
C	-1.111337000	3.496947000	2.492242000
C	-2.098905000	2.513543000	2.563970000
C	-2.228393000	1.578081000	1.538914000
C	-2.869980000	0.305499000	-1.145922000
C	-3.881685000	1.293841000	-1.168885000
C	-5.136567000	1.007697000	-1.692477000
C	-5.418406000	-0.269377000	-2.189755000
C	-4.435848000	-1.261311000	-2.158873000
C	-3.173910000	-0.981449000	-1.642582000
H	0.317309000	2.634992000	-0.472322000
H	0.518082000	4.308352000	1.330148000
H	-1.014442000	4.230149000	3.287879000
H	-2.770511000	2.473529000	3.416612000
H	-2.991524000	0.809008000	1.598809000
H	-3.661379000	2.291124000	-0.804181000
H	-5.897155000	1.782504000	-1.718368000
H	-6.403349000	-0.491247000	-2.590547000
H	-4.657113000	-2.260124000	-2.523229000
H	-2.431184000	-1.768729000	-1.573901000
C	3.741540000	0.153642000	-0.839782000
C	4.254275000	0.431442000	0.435092000
C	4.631210000	-0.227749000	-1.860663000
C	5.624329000	0.311187000	0.682225000
H	3.580338000	0.734849000	1.224532000
C	5.991801000	-0.368588000	-1.604350000
H	4.235505000	-0.407479000	-2.854731000
C	6.494750000	-0.096234000	-0.328322000
H	6.009216000	0.539016000	1.673022000
H	6.662260000	-0.681489000	-2.400720000
H	7.558550000	-0.195835000	-0.127644000
C	0.054084000	-1.663776000	1.247612000
C	-0.183030000	-2.756387000	0.400107000
C	-0.562579000	-1.634514000	2.506773000
C	-1.035419000	-3.788444000	0.796638000
H	0.326337000	-2.812831000	-0.557476000
C	-1.430090000	-2.654192000	2.894879000
H	-0.349124000	-0.804228000	3.172408000

C	-1.668887000	-3.734171000	2.040213000
H	-1.195909000	-4.638932000	0.139289000
H	-1.912876000	-2.613376000	3.867777000
H	-2.336805000	-4.534784000	2.346834000

<u>C=S-ylide 7c</u>

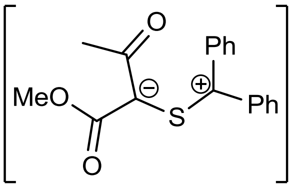

Sum of electronic and zero-point Energies= -1319.079158
Sum of electronic and thermal Energies= -1319.058471
Sum of electronic and thermal Enthalpies= -1319.057527
Sum of electronic and thermal Free Energies= -1319.130534

Standard orientation. Coordinates (Angstroms):

S	0.272705000	-1.359075000	-0.318160000
C	1.761070000	-0.663918000	0.262321000
C	2.879633000	-1.119139000	-0.541045000
O	2.760262000	-1.764799000	-1.582474000
C	5.218909000	-1.237738000	-0.832207000
H	6.104965000	-0.865620000	-0.315892000
H	5.239031000	-2.329780000	-0.883391000
H	5.173109000	-0.837260000	-1.848500000
O	4.104645000	-0.782642000	-0.052225000
C	1.734899000	0.070094000	1.517136000
O	0.672164000	0.348787000	2.091409000
C	3.044530000	0.503391000	2.167556000
H	3.694435000	-0.350202000	2.378239000
H	3.606907000	1.173904000	1.510749000
H	2.792764000	1.019657000	3.096370000
C	-0.958213000	-0.204751000	-0.184814000
C	-2.315176000	-0.737940000	-0.078344000
C	-3.392877000	-0.056651000	-0.691935000
C	-2.581507000	-1.956816000	0.585101000
C	-4.675462000	-0.590468000	-0.662413000
H	-3.205712000	0.873376000	-1.217224000
C	-3.869329000	-2.483097000	0.611582000
H	-1.780456000	-2.459925000	1.115559000
C	-4.918974000	-1.805416000	-0.012922000
H	-5.488388000	-0.061733000	-1.151557000
H	-4.057297000	-3.414418000	1.137648000
H	-5.924703000	-2.215001000	0.015245000
C	-0.730052000	1.242129000	-0.261754000
C	-1.426383000	2.117612000	0.592637000
C	0.149814000	1.780293000	-1.220987000
C	-1.246188000	3.495004000	0.490240000
H	-2.084229000	1.708019000	1.351504000
C	0.311997000	3.157002000	-1.330693000
H	0.683032000	1.114935000	-1.892199000
C	-0.380661000	4.017955000	-0.472426000
H	-1.779734000	4.159118000	1.164005000
H	0.976182000	3.561336000	-2.089115000
H	-0.245928000	5.092757000	-0.556765000

C=S-ylide 7d

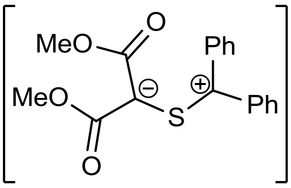

Sum of electronic and zero-point Energies= -1394.298038
Sum of electronic and thermal Energies= -1394.276259
Sum of electronic and thermal Enthalpies= -1394.275315
Sum of electronic and thermal Free Energies= -1394.351040

Standard orientation. Coordinates (Angstroms):

S	0.060221000	-1.447624000	-0.173782000
C	1.624608000	-0.763592000	0.118621000
C	2.633239000	-1.487353000	-0.636165000
O	2.352423000	-2.330131000	-1.491095000
C	4.904110000	-1.931214000	-1.051304000
H	5.865000000	-1.556997000	-0.695213000
H	4.817819000	-3.003917000	-0.856159000
H	4.802868000	-1.759735000	-2.126689000
O	3.916308000	-1.195067000	-0.318236000
C	1.767632000	0.240594000	1.162140000
O	0.853278000	0.646703000	1.874547000
O	3.033943000	0.713733000	1.289663000
C	3.217593000	1.672410000	2.338108000
H	4.272783000	1.946629000	2.296948000
H	2.587729000	2.552021000	2.177758000
H	2.976291000	1.239621000	3.313346000
C	-1.126300000	-0.244764000	-0.190433000
C	-2.490558000	-0.720957000	0.042075000
C	-3.571852000	-0.119286000	-0.643983000
C	-2.763040000	-1.810165000	0.899596000
C	-4.863980000	-0.608444000	-0.495509000
H	-3.380957000	0.710310000	-1.315908000
C	-4.060541000	-2.293385000	1.042743000
H	-1.958635000	-2.243358000	1.484206000
C	-5.114002000	-1.697985000	0.345786000
H	-5.679703000	-0.143291000	-1.041390000
H	-4.251649000	-3.124114000	1.715686000
H	-6.126765000	-2.072281000	0.465051000
C	-0.869661000	1.174264000	-0.460855000
C	-1.531184000	2.166006000	0.287420000
C	-0.000959000	1.568573000	-1.497503000
C	-1.326600000	3.515829000	0.008946000
H	-2.185560000	1.871165000	1.100816000
C	0.185090000	2.916243000	-1.783086000
H	0.509247000	0.813924000	-2.086954000
C	-0.471843000	3.894235000	-1.027430000
H	-1.834193000	4.270624000	0.602556000
H	0.841211000	3.206889000	-2.598388000
H	-0.317153000	4.946520000	-1.249190000

C=S-ylide 7e

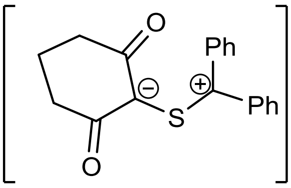

Sum of electronic and zero-point Energies= -1281.965105
Sum of electronic and thermal Energies= -1281.946373
Sum of electronic and thermal Enthalpies= -1281.945429
Sum of electronic and thermal Free Energies= -1282.013728

Standard orientation. Coordinates (Angstroms):

C	0.925812000	-0.187479000	-0.246293000
S	-0.207908000	-1.395309000	-0.598558000
C	2.299723000	-0.643418000	-0.041706000
C	3.382964000	0.151273000	-0.485570000
C	4.690419000	-0.302834000	-0.361324000
C	4.951632000	-1.549026000	0.217911000
C	3.894568000	-2.339847000	0.675065000
C	2.582387000	-1.894684000	0.550959000
C	0.606659000	1.243716000	-0.237129000
C	1.169338000	2.089957000	0.737460000
C	0.904333000	3.457211000	0.723155000
C	0.086260000	4.001196000	-0.268852000
C	-0.474061000	3.170403000	-1.245574000
C	-0.227551000	1.802204000	-1.225993000
H	3.184944000	1.107426000	-0.957114000
H	5.509260000	0.313023000	-0.721544000
H	5.975571000	-1.896810000	0.320911000
H	4.093718000	-3.298045000	1.145723000
H	1.768892000	-2.490051000	0.950947000
H	1.789316000	1.663105000	1.518356000
H	1.334389000	4.096466000	1.488635000
H	-0.114165000	5.068889000	-0.284147000
H	-1.099919000	3.592388000	-2.026607000
H	-0.657363000	1.161804000	-1.989509000
C	-1.779291000	-0.828797000	-0.096409000
C	-2.845371000	-1.239031000	-0.975502000
C	-1.946066000	-0.199977000	1.194412000
C	-4.274597000	-0.987130000	-0.498552000
C	-3.393861000	0.067638000	1.616905000
C	-4.397793000	-0.927257000	1.027061000
H	-4.909688000	-1.764363000	-0.935713000
H	-4.600138000	-0.031449000	-0.937526000
H	-3.414091000	0.073049000	2.711203000
H	-3.643171000	1.088888000	1.289633000
H	-5.418547000	-0.648690000	1.314174000
H	-4.211604000	-1.924253000	1.447695000
O	-2.635024000	-1.762422000	-2.080331000
O	-1.014214000	0.126028000	1.940541000

C=S-ylide 7f

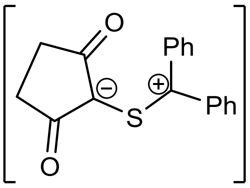

Sum of electronic and zero-point Energies= -1242.679576
Sum of electronic and thermal Energies= -1242.661696
Sum of electronic and thermal Enthalpies= -1242.660752
Sum of electronic and thermal Free Energies= -1242.727794

Standard orientation. Coordinates (Angstroms):

C	0.748458000	-0.203734000	-0.224353000
S	-0.322001000	-1.493274000	-0.503009000
C	2.150106000	-0.579003000	-0.052215000
C	3.173047000	0.269880000	-0.538771000
C	4.507505000	-0.105312000	-0.445208000
C	4.857651000	-1.325434000	0.142945000
C	3.861908000	-2.169257000	0.640802000
C	2.523038000	-1.802354000	0.550233000
C	0.344965000	1.205200000	-0.226841000
C	0.890176000	2.099794000	0.714597000
C	0.551968000	3.450334000	0.684107000
C	-0.323047000	3.930752000	-0.291721000
C	-0.867052000	3.052171000	-1.235616000
C	-0.547461000	1.700065000	-1.199013000
H	2.906347000	1.204849000	-1.018771000
H	5.278412000	0.551534000	-0.837303000
H	5.902677000	-1.611219000	0.221292000
H	4.129857000	-3.106712000	1.118955000
H	1.759241000	-2.437754000	0.985354000
H	1.556418000	1.722969000	1.483226000
H	0.969395000	4.125992000	1.424817000
H	-0.581644000	4.985532000	-0.319580000
H	-1.537780000	3.424891000	-2.004321000
H	-0.965994000	1.024535000	-1.937895000
C	-1.896805000	-1.012697000	-0.010583000
C	-3.067208000	-1.525832000	-0.675132000
C	-2.206419000	-0.277904000	1.190124000
C	-4.289750000	-1.091962000	0.151437000
C	-3.739903000	-0.265304000	1.329741000
H	-4.830119000	-1.987894000	0.477948000
H	-4.975227000	-0.528600000	-0.491474000
H	-4.010847000	-0.673305000	2.309661000
H	-4.081551000	0.776371000	1.316578000
O	-3.120505000	-2.195584000	-1.707672000
O	-1.428773000	0.236182000	1.994226000

26. Transition states for cycloadditions of diazo compounds 1 to aliphatic thioketone 2b

Gas phase, 6-31G(d), PBE1PBE

TS$_{1a+2b\rightarrow6'a}$

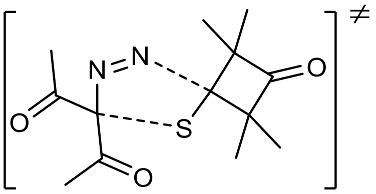

Imaginary Freq.: -359.00 cm^{-1}
Sum of electronic and zero-point Energies= -1239.178436
Sum of electronic and thermal Energies= -1239.156448
Sum of electronic and thermal Enthalpies= -1239.155504
Sum of electronic and thermal Free Energies= -1239.228798

Standard orientation. Coordinates (Angstroms):

S	-0.333158000	0.086131000	1.351424000
C	-2.026482000	0.038568000	-0.375427000
C	-2.866010000	1.236926000	-0.019805000
O	-3.744653000	1.131503000	0.815531000
C	-2.541154000	2.550030000	-0.701556000
H	-1.508802000	2.847815000	-0.484932000
H	-2.642460000	2.465552000	-1.789625000
H	-3.224518000	3.313568000	-0.326659000
C	-2.493064000	-1.408596000	-0.263991000
O	-2.034648000	-2.234914000	-1.030119000
C	-3.479368000	-1.749767000	0.824060000
H	-3.130775000	-1.391798000	1.796801000
H	-4.440471000	-1.261136000	0.637768000
H	-3.600339000	-2.834760000	0.836715000
C	0.981843000	0.048184000	0.292443000
C	1.950870000	-1.151044000	0.019457000
C	2.128872000	1.108667000	0.165950000
C	3.031696000	-0.079013000	-0.239432000
C	2.234663000	-1.995833000	1.276393000
H	2.457762000	-1.380009000	2.152852000
H	1.361069000	-2.612382000	1.512894000
H	3.090261000	-2.654186000	1.091612000
C	1.667437000	-2.078050000	-1.171957000
H	1.610838000	-1.534356000	-2.118322000
H	2.472809000	-2.814983000	-1.258845000
H	0.720659000	-2.609074000	-1.024472000
C	2.537440000	1.709111000	1.526181000
H	3.484475000	2.249555000	1.421339000
H	1.768220000	2.409003000	1.869984000
H	2.663379000	0.944528000	2.298399000
C	2.025531000	2.227426000	-0.877218000
H	1.241486000	2.941723000	-0.596396000
H	2.975972000	2.770044000	-0.920068000
H	1.810708000	1.849300000	-1.878985000
O	4.162567000	-0.136789000	-0.653677000
N	-1.125705000	0.204764000	-1.369917000
N	0.017201000	0.238871000	-1.576845000

$TS_{1b+2b→6'b}$

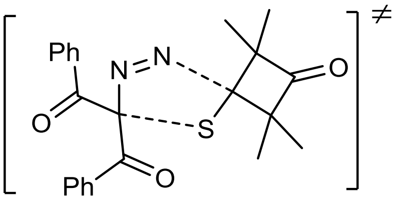

Imaginary Freq.: -329.45 cm^{-1}
Sum of electronic and zero-point Energies= -1622.539527
Sum of electronic and thermal Energies= -1622.511587
Sum of electronic and thermal Enthalpies= -1622.510643
Sum of electronic and thermal Free Energies= -1622.600119

Standard orientation. Coordinates (Angstroms):

S	-0.731945000	-0.905732000	-1.212674000
C	0.819041000	0.182488000	0.292644000
C	1.522023000	1.230164000	-0.545348000
O	2.096863000	0.897640000	-1.569321000
C	1.516454000	-0.989638000	0.990277000
O	1.185999000	-1.216170000	2.147002000
C	-2.123145000	-0.618301000	-0.312002000
C	-2.963279000	-1.644191000	0.517121000
C	-3.387656000	0.201901000	-0.734388000
C	-4.175563000	-0.737736000	0.207190000
C	-3.061294000	-3.025132000	-0.160169000
H	-3.298321000	-2.951881000	-1.225788000
H	-2.105670000	-3.551598000	-0.065139000
H	-3.841896000	-3.620456000	0.325833000
C	-2.654815000	-1.824374000	2.010711000
H	-2.727948000	-0.886916000	2.567351000
H	-3.368718000	-2.529799000	2.449075000
H	-1.643354000	-2.224666000	2.144702000
C	-3.762847000	-0.007807000	-2.215483000
H	-4.768835000	0.384082000	-2.401440000
H	-3.052094000	0.525340000	-2.856011000
H	-3.747413000	-1.062901000	-2.504331000
C	-3.476636000	1.693634000	-0.390531000
H	-2.731364000	2.260409000	-0.961354000
H	-4.469859000	2.069874000	-0.658307000
H	-3.320483000	1.888178000	0.672790000
O	-5.317274000	-0.733317000	0.595907000
N	-0.180833000	0.656384000	1.087291000
N	-1.327359000	0.613660000	1.246907000
C	2.553153000	-1.814167000	0.308880000
C	2.709861000	-1.923339000	-1.081533000
C	3.381984000	-2.573525000	1.156749000
C	3.685358000	-2.770204000	-1.607098000
H	2.085619000	-1.347564000	-1.749613000
C	4.360432000	-3.405961000	0.626490000
H	3.238775000	-2.494805000	2.228945000
C	4.513199000	-3.507080000	-0.759729000
H	3.795665000	-2.852796000	-2.684539000
H	5.001347000	-3.979056000	1.290664000
H	5.274672000	-4.161063000	-1.176605000
C	1.475085000	2.665242000	-0.123987000
C	1.411254000	3.082606000	1.215382000
C	1.579190000	3.628858000	-1.141963000
C	1.432262000	4.442353000	1.525397000
H	1.372218000	2.353728000	2.017743000
C	1.580424000	4.983725000	-0.828790000

H	1.652900000	3.292052000	-2.170662000
C	1.506758000	5.392939000	0.506564000
H	1.394401000	4.757076000	2.564271000
H	1.642763000	5.722650000	-1.622651000
H	1.513900000	6.451571000	0.751467000

$TS_{1c+2b\rightarrow 6'c}$

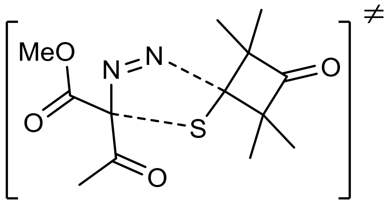

Imaginary Freq.: -361.10 cm^{-1}
Sum of electronic and zero-point Energies= -1314.401568
Sum of electronic and thermal Energies= -1314.378606
Sum of electronic and thermal Enthalpies= -1314.377662
Sum of electronic and thermal Free Energies= -1314.453557

Standard orientation. Coordinates (Angstroms):

S	-0.131549000	-0.217033000	1.365750000
C	-1.810493000	-0.445096000	-0.357055000
C	-2.789199000	0.617285000	0.024334000
O	-3.661672000	0.460339000	0.850734000
C	-3.400639000	2.870451000	-0.270732000
H	-3.083858000	3.696245000	-0.907039000
H	-4.447882000	2.619264000	-0.454530000
H	-3.267441000	3.120047000	0.784615000
O	-2.548801000	1.765376000	-0.632350000
C	-2.098612000	-1.934917000	-0.268695000
O	-1.533204000	-2.688505000	-1.038037000
C	-3.044480000	-2.405626000	0.807579000
H	-2.746491000	-2.021417000	1.787529000
H	-4.056254000	-2.033341000	0.619733000
H	-3.035495000	-3.497186000	0.807921000
C	1.155131000	0.021117000	0.298252000
C	2.333791000	-0.961829000	-0.009067000
C	2.072008000	1.285966000	0.187333000
C	3.181029000	0.305882000	-0.260556000
C	2.797788000	-1.750677000	1.230864000
H	2.911886000	-1.114291000	2.113541000
H	2.064363000	-2.527949000	1.470152000
H	3.761788000	-2.227847000	1.024111000
C	2.213825000	-1.912896000	-1.208779000
H	2.047990000	-1.379782000	-2.148073000
H	3.138759000	-2.490141000	-1.311451000
H	1.381839000	-2.610139000	-1.058500000
C	2.376675000	1.924177000	1.557332000
H	3.202146000	2.637235000	1.456645000
H	1.492577000	2.457470000	1.922996000
H	2.655264000	1.181986000	2.311218000
C	1.720891000	2.384910000	-0.823360000
H	0.776098000	2.866815000	-0.544640000
H	2.508015000	3.146423000	-0.823181000
H	1.625986000	2.002037000	-1.841866000
O	4.293103000	0.476064000	-0.694508000
N	-0.952079000	-0.134760000	-1.353909000
N	0.171192000	0.070251000	-1.559319000

TS$_{1d+2b\rightarrow6'd}$

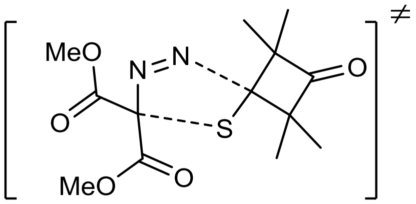

Imaginary Freq.: -358.58 cm^{-1}
Sum of electronic and zero-point Energies= -1389.619918
Sum of electronic and thermal Energies= -1389.595910
Sum of electronic and thermal Enthalpies= -1389.594966
Sum of electronic and thermal Free Energies= -1389.673792

Standard orientation. Coordinates (Angstroms):

S	-0.024414000	-0.167856000	1.321702000
C	-1.677038000	0.047818000	-0.415809000
C	-2.416468000	1.285433000	0.004423000
O	-3.324702000	1.301965000	0.798870000
C	-2.472977000	3.628869000	-0.196760000
H	-1.951656000	4.385490000	-0.782620000
H	-3.543155000	3.638098000	-0.416759000
H	-2.322372000	3.794062000	0.872853000
O	-1.889972000	2.372934000	-0.594027000
C	-2.279346000	-1.330229000	-0.437496000
O	-1.904451000	-2.192414000	-1.204662000
O	-3.206401000	-1.478130000	0.507593000
C	-3.762163000	-2.802722000	0.618673000
H	-4.490216000	-2.738034000	1.426416000
H	-4.244303000	-3.092670000	-0.317983000
H	-2.976263000	-3.522672000	0.859666000
C	1.316963000	-0.084120000	0.305041000
C	2.318036000	-1.230438000	-0.054970000
C	2.431632000	1.012984000	0.276986000
C	3.369160000	-0.109947000	-0.225472000
C	2.625442000	-2.154348000	1.140124000
H	2.831398000	-1.596465000	2.058492000
H	1.768284000	-2.808954000	1.330523000
H	3.498767000	-2.774822000	0.911662000
C	2.055658000	-2.080083000	-1.306538000
H	2.001033000	-1.476672000	-2.215867000
H	2.866476000	-2.804902000	-1.435277000
H	1.112221000	-2.627996000	-1.200361000
C	2.813948000	1.510232000	1.685440000
H	3.746475000	2.082857000	1.635006000
H	2.022437000	2.158610000	2.076458000
H	2.954004000	0.689058000	2.394571000
C	2.281285000	2.212564000	-0.667156000
H	1.416913000	2.818956000	-0.371252000
H	3.177594000	2.839348000	-0.609857000
H	2.150169000	1.910557000	-1.708677000
O	4.499855000	-0.102974000	-0.644512000
N	-0.760205000	0.213045000	-1.397798000
N	0.382119000	0.239056000	-1.590667000

***TS**$_{1e+2b\rightarrow6'e}$*

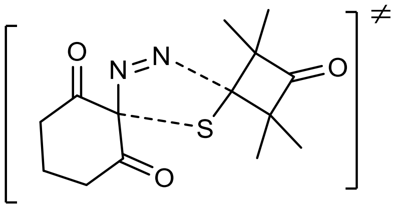

Imaginary Freq.: -360.80 cm^{-1}
Sum of electronic and zero-point Energies= -1277.278729
Sum of electronic and thermal Energies= -1277.257819
Sum of electronic and thermal Enthalpies= -1277.256875
Sum of electronic and thermal Free Energies= -1277.328224

Standard orientation. Coordinates (Angstroms):

S	-1.271473000	0.177736000	0.000000000
C	0.577642000	1.757259000	0.000000000
C	0.358682000	2.447392000	1.319611000
C	0.358682000	2.447392000	-1.319611000
C	-0.272424000	-1.188032000	0.000000000
C	-0.135027000	-2.257293000	-1.136348000
C	-0.135027000	-2.257293000	1.136348000
C	0.159134000	-3.264123000	0.000000000
C	-1.470219000	-2.549129000	-1.849162000
H	-2.299443000	-2.683076000	-1.148252000
H	-1.723702000	-1.717149000	-2.514745000
H	-1.375325000	-3.461926000	-2.447081000
C	0.978227000	-2.095991000	-2.180525000
H	1.971892000	-2.050344000	-1.728987000
H	0.962109000	-2.948710000	-2.867350000
H	0.820932000	-1.180089000	-2.762362000
C	-1.470219000	-2.549129000	1.849162000
H	-1.375325000	-3.461926000	2.447081000
H	-1.723702000	-1.717149000	2.514745000
H	-2.299443000	-2.683076000	1.148252000
C	0.978227000	-2.095991000	2.180525000
H	0.820932000	-1.180089000	2.762362000
H	0.962109000	-2.948710000	2.867350000
H	1.971892000	-2.050344000	1.728987000
O	0.529412000	-4.411416000	0.000000000
N	1.513558000	0.786118000	0.000000000
N	1.621134000	-0.369947000	0.000000000
O	0.977581000	2.130916000	-2.316106000
O	0.977581000	2.130916000	2.316106000
C	-0.613291000	4.387464000	0.000000000
C	-0.702847000	3.533337000	-1.277102000
C	-0.702847000	3.533337000	1.277102000
H	-1.419948000	5.127782000	0.000000000
H	0.328822000	4.950895000	0.000000000
H	-0.591265000	4.135333000	2.183080000
H	-1.683649000	3.038873000	1.327304000
H	-1.683649000	3.038873000	-1.327304000
H	-0.591265000	4.135333000	-2.183080000

$TS_{1f+2b\rightarrow 6'f}$

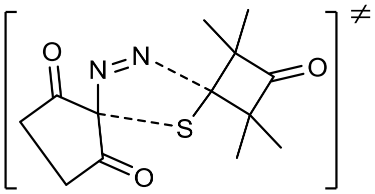

Imaginary Freq.: -380.01 cm^{-1}
Sum of electronic and zero-point Energies= -1237.988516
Sum of electronic and thermal Energies= -1237.968493
Sum of electronic and thermal Enthalpies= -1237.967549
Sum of electronic and thermal Free Energies= -1238.037213

Standard orientation. Coordinates (Angstroms):

S	-0.344780000	-0.129027000	1.286687000
C	-1.948605000	-0.059187000	-0.561082000
C	-2.629659000	1.238867000	-0.238347000
C	-2.853583000	-1.203549000	-0.224126000
C	0.993108000	-0.058132000	0.247517000
C	2.136388000	-1.124469000	0.111462000
C	1.987279000	1.143719000	0.083409000
C	3.060230000	0.072130000	-0.214135000
C	2.494384000	-1.790552000	1.455052000
H	2.593308000	-1.066967000	2.269446000
H	1.712966000	-2.505608000	1.733305000
H	3.443941000	-2.327518000	1.356471000
C	2.043997000	-2.197946000	-0.980065000
H	1.906299000	-1.772941000	-1.976606000
H	2.969597000	-2.783172000	-0.990751000
H	1.209992000	-2.878935000	-0.773478000
C	2.249563000	1.890344000	1.405862000
H	3.120309000	2.544619000	1.290423000
H	1.379617000	2.503738000	1.663012000
H	2.439764000	1.210611000	2.241574000
C	1.741475000	2.162438000	-1.038507000
H	0.789649000	2.681735000	-0.875475000
H	2.545660000	2.905570000	-1.040054000
H	1.715850000	1.697846000	-2.027144000
O	4.198178000	0.140134000	-0.606373000
N	-0.998662000	-0.128022000	-1.490202000
N	0.162237000	-0.131962000	-1.607670000
O	-2.787408000	-2.333233000	-0.648499000
O	-2.335382000	2.334459000	-0.655737000
C	-3.883879000	-0.647152000	0.766865000
C	-3.773531000	0.892033000	0.722365000
H	-4.682805000	1.376692000	0.352246000
H	-3.550006000	1.332788000	1.699346000
H	-3.656650000	-1.054232000	1.758414000
H	-4.872149000	-1.022043000	0.484637000

27. Thiodiazolines 6' obtained from diazo compounds 1 and aliphatic thioketone 2b

Gas phase, 6-31G(d), PBE1PBE

Thiadiazoline 6'a

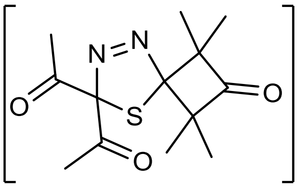

Sum of electronic and zero-point Energies= -1239.216459
Sum of electronic and thermal Energies= -1239.195065
Sum of electronic and thermal Enthalpies= -1239.194121
Sum of electronic and thermal Free Energies= -1239.265988

Standard orientation. Coordinates (Angstroms):

S	0.468126000	-0.309787000	1.212922000
C	1.707955000	-0.099843000	-0.141931000
C	2.870930000	-1.101104000	-0.003616000
O	3.287903000	-1.385752000	1.100583000
C	3.466263000	-1.653310000	-1.278114000
H	2.720549000	-2.257419000	-1.807481000
H	3.744669000	-0.845623000	-1.964696000
H	4.337428000	-2.262714000	-1.030693000
C	2.196700000	1.392303000	-0.079883000
O	1.648735000	2.229115000	-0.762789000
C	3.312974000	1.718409000	0.885846000
H	3.197041000	1.190160000	1.835652000
H	4.272098000	1.399213000	0.457749000
H	3.338994000	2.798404000	1.042077000
C	-0.879340000	-0.171322000	-0.046754000
C	-1.857412000	1.100502000	-0.067386000
C	-2.129681000	-1.142286000	0.122120000
C	-3.021510000	0.096783000	-0.066848000
C	-1.814662000	1.963360000	1.201307000
H	-1.848743000	1.372187000	2.121324000
H	-0.899755000	2.564112000	1.219365000
H	-2.676765000	2.638837000	1.199507000
C	-1.802199000	1.988641000	-1.319081000
H	-1.912570000	1.409222000	-2.238915000
H	-2.618208000	2.717575000	-1.272817000
H	-0.848377000	2.522833000	-1.364817000
C	-2.290910000	-1.741671000	1.528987000
H	-3.291440000	-2.178266000	1.615789000
H	-1.549668000	-2.528990000	1.699410000
H	-2.183571000	-0.996570000	2.324213000
C	-2.305163000	-2.230154000	-0.943567000
H	-1.549570000	-3.015031000	-0.820792000
H	-3.294518000	-2.685841000	-0.829953000
H	-2.222216000	-1.831086000	-1.956666000
O	-4.212123000	0.227963000	-0.203677000
N	0.991811000	-0.291008000	-1.419662000
N	-0.238265000	-0.329973000	-1.359541000

Thiadiazoline 6'b

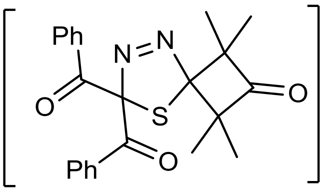

Sum of electronic and zero-point Energies= -1622.583846
Sum of electronic and thermal Energies= -1622.556591
Sum of electronic and thermal Enthalpies= -1622.555647
Sum of electronic and thermal Free Energies= -1622.642445

Standard orientation. Coordinates (Angstroms):

S	1.046309000	0.450364000	-1.179925000
C	-0.283634000	-0.252816000	-0.115460000
C	-1.364705000	-1.019174000	-0.936514000
O	-1.333730000	-0.965726000	-2.154319000
C	-0.848228000	0.899450000	0.777738000
O	-0.507836000	0.944624000	1.949012000
C	2.318941000	-0.279464000	-0.059971000
C	3.245898000	0.665901000	0.846563000
C	3.611145000	-0.919556000	-0.732033000
C	4.442882000	-0.120176000	0.285956000
C	3.256784000	2.141406000	0.421976000
H	3.377717000	2.274226000	-0.657142000
H	2.321538000	2.627845000	0.717209000
H	4.089156000	2.648293000	0.921927000
C	3.067638000	0.549855000	2.366947000
H	3.141229000	-0.484441000	2.711291000
H	3.849648000	1.134029000	2.863545000
H	2.089495000	0.939514000	2.667133000
C	3.877029000	-0.455617000	-2.173830000
H	4.894057000	-0.744734000	-2.459197000
H	3.171438000	-0.929152000	-2.863950000
H	3.793259000	0.629316000	-2.295005000
C	3.756210000	-2.442237000	-0.628374000
H	3.035834000	-2.942015000	-1.286365000
H	4.765621000	-2.728322000	-0.942491000
H	3.595486000	-2.801940000	0.390184000
O	5.614107000	-0.136209000	0.571949000
N	0.384222000	-1.235610000	0.796346000
N	1.613868000	-1.240923000	0.790849000
C	-1.754345000	1.935568000	0.195615000
C	-2.024371000	2.073025000	-1.176440000
C	-2.351122000	2.828844000	1.103690000
C	-2.877833000	3.081155000	-1.623096000
H	-1.575347000	1.407654000	-1.903772000
C	-3.206521000	3.828132000	0.653560000
H	-2.128269000	2.717241000	2.159276000
C	-3.471752000	3.956726000	-0.713027000
H	-3.076247000	3.181436000	-2.686287000
H	-3.666363000	4.508134000	1.365228000
H	-4.138442000	4.738653000	-1.066803000
C	-2.431609000	-1.777012000	-0.218314000
C	-2.535606000	-1.863996000	1.181284000
C	-3.395131000	-2.418558000	-1.019118000
C	-3.586631000	-2.575497000	1.759019000
H	-1.796071000	-1.399447000	1.820575000
C	-4.436952000	-3.130456000	-0.437193000
H	-3.303580000	-2.341286000	-2.097086000

C	-4.535573000	-3.208976000	0.955779000
H	-3.658859000	-2.637869000	2.841046000
H	-5.173839000	-3.623442000	-1.065132000
H	-5.350454000	-3.764457000	1.412561000

Thiadiazoline 6'c

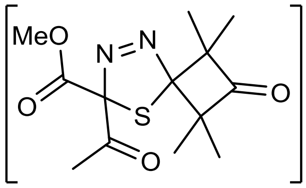

Sum of electronic and zero-point Energies= -1314.438090
Sum of electronic and thermal Energies= -1314.415753
Sum of electronic and thermal Enthalpies= -1314.414809
Sum of electronic and thermal Free Energies= -1314.490064

Standard orientation. Coordinates (Angstroms):

S	-0.340625000	-0.093127000	1.222301000
C	-1.541174000	-0.382000000	-0.161798000
C	-2.752296000	0.543693000	0.011132000
O	-3.792083000	0.233773000	0.549738000
C	-3.548825000	2.727247000	-0.345517000
H	-3.173510000	3.633238000	-0.820335000
H	-4.454075000	2.374351000	-0.845060000
H	-3.764290000	2.903206000	0.711359000
O	-2.490670000	1.759318000	-0.484480000
C	-1.843027000	-1.914288000	-0.198242000
O	-1.182533000	-2.609290000	-0.942010000
C	-2.875718000	-2.489669000	0.740933000
H	-2.797494000	-2.058384000	1.742868000
H	-3.880638000	-2.252823000	0.378377000
H	-2.737929000	-3.572434000	0.776332000
C	1.016162000	0.113188000	-0.028099000
C	2.216820000	-0.946051000	-0.083013000
C	2.053219000	1.298019000	0.195911000
C	3.166694000	0.262786000	-0.047269000
C	2.341790000	-1.835621000	1.162451000
H	2.276375000	-1.273990000	2.099375000
H	1.550039000	-2.591450000	1.166248000
H	3.311971000	-2.343111000	1.139621000
C	2.313842000	-1.798055000	-1.356457000
H	2.329900000	-1.184964000	-2.260529000
H	3.236030000	-2.387713000	-1.323582000
H	1.458711000	-2.478397000	-1.422095000
C	2.098409000	1.850310000	1.630149000
H	3.000220000	2.460557000	1.746855000
H	1.223593000	2.478323000	1.827265000
H	2.129331000	1.063038000	2.390431000
C	2.005437000	2.449070000	-0.815491000
H	1.099100000	3.047979000	-0.669335000
H	2.876042000	3.096118000	-0.664833000
H	2.014163000	2.090336000	-1.846884000
O	4.357987000	0.371126000	-0.196104000
N	-0.837361000	-0.031439000	-1.424760000
N	0.366339000	0.199290000	-1.336410000

Thiadiazoline 6'd

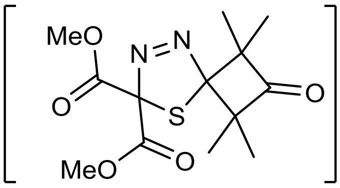

Sum of electronic and zero-point Energies= -1389.661028
Sum of electronic and thermal Energies= -1389.637579
Sum of electronic and thermal Enthalpies= -1389.636634
Sum of electronic and thermal Free Energies= -1389.714711

Standard orientation. Coordinates (Angstroms):

S	-0.226123000	-0.394335000	1.105582000
C	-1.405245000	-0.024085000	-0.256162000
C	-2.123367000	1.338519000	-0.141497000
O	-3.319573000	1.477703000	-0.234986000
C	-1.826385000	3.658569000	0.080037000
H	-0.984264000	4.340352000	0.195137000
H	-2.375716000	3.870814000	-0.840144000
H	-2.505560000	3.734403000	0.932522000
O	-1.246288000	2.339733000	0.021671000
C	-2.448597000	-1.147497000	-0.378501000
O	-2.609589000	-1.830186000	-1.359551000
O	-3.118003000	-1.275081000	0.776041000
C	-4.135119000	-2.294012000	0.789981000
H	-4.561804000	-2.262139000	1.792158000
H	-4.897061000	-2.074868000	0.038164000
H	-3.695475000	-3.273594000	0.586426000
C	1.191947000	-0.203483000	-0.068539000
C	2.332599000	-1.317101000	-0.053128000
C	2.287580000	0.936343000	0.182594000
C	3.348263000	-0.162941000	0.000034000
C	2.372898000	-2.173193000	1.223278000
H	2.304788000	-1.578688000	2.140037000
H	1.549091000	-2.893794000	1.225508000
H	3.319564000	-2.722955000	1.251850000
C	2.434386000	-2.210781000	-1.294945000
H	2.455534000	-1.630166000	-2.219596000
H	3.353285000	-2.803664000	-1.236480000
H	1.582221000	-2.898434000	-1.342871000
C	2.279989000	1.518472000	1.603517000
H	3.186916000	2.114907000	1.749559000
H	1.405579000	2.162135000	1.741326000
H	2.255168000	0.748641000	2.380754000
C	2.361527000	2.064035000	-0.854724000
H	1.478215000	2.707409000	-0.780761000
H	3.253184000	2.669810000	-0.661748000
H	2.420218000	1.680963000	-1.875989000
O	4.548297000	-0.126978000	-0.108739000
N	-0.609050000	0.033486000	-1.527300000
N	0.610834000	-0.056399000	-1.403031000

Thiadiazoline 6'e

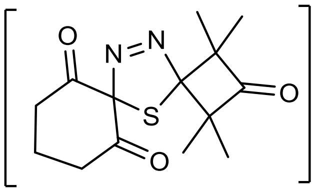

Sum of electronic and zero-point Energies= -1277.311953
Sum of electronic and thermal Energies= -1277.291625
Sum of electronic and thermal Enthalpies= -1277.290680
Sum of electronic and thermal Free Energies= -1277.359812

Standard orientation. Coordinates (Angstroms):

S	-0.098880000	-0.590102000	-1.298278000
C	-1.489327000	-0.203683000	-0.203022000
C	-2.622292000	-1.251880000	-0.249077000
C	-2.104217000	1.236644000	-0.397494000
C	1.079626000	-0.163535000	0.058663000
C	1.995533000	1.152288000	-0.036652000
C	2.380445000	-1.067109000	0.213101000
C	3.185005000	0.241251000	0.305885000
C	2.087463000	1.747479000	-1.449474000
H	2.325996000	0.999971000	-2.211886000
H	1.134012000	2.211855000	-1.716319000
H	2.876027000	2.507633000	-1.463862000
C	1.731436000	2.261545000	0.989436000
H	1.679233000	1.876346000	2.010802000
H	2.546334000	2.991487000	0.940263000
H	0.791691000	2.774775000	0.760341000
C	2.743635000	-1.878251000	-1.041849000
H	3.762593000	-2.264460000	-0.933141000
H	2.059099000	-2.724226000	-1.160408000
H	2.709258000	-1.282921000	-1.959765000
C	2.459344000	-1.956823000	1.459288000
H	1.758450000	-2.795715000	1.377691000
H	3.472856000	-2.362780000	1.545154000
H	2.229641000	-1.405889000	2.373907000
O	4.332292000	0.471532000	0.596940000
N	-0.941029000	-0.155739000	1.209057000
N	0.288560000	-0.137944000	1.290002000
O	-1.424855000	2.149360000	-0.803151000
O	-2.623582000	-2.150473000	-1.059930000
C	-3.963658000	0.441194000	1.136970000
C	-3.565331000	1.385459000	-0.008192000
C	-3.752827000	-1.038521000	0.749434000
H	-5.013804000	0.606402000	1.397671000
H	-3.370302000	0.684565000	2.023191000
H	-3.519121000	-1.635438000	1.640741000
H	-4.649176000	-1.477738000	0.299389000
H	-4.158007000	1.156242000	-0.908689000
H	-3.745267000	2.437604000	0.230973000

Thiadiazoline 6'f

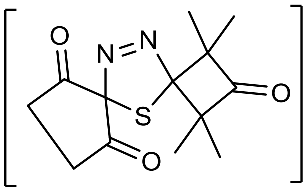

Sum of electronic and zero-point Energies= -1238.027126
Sum of electronic and thermal Energies= -1238.007762
Sum of electronic and thermal Enthalpies= -1238.006818
Sum of electronic and thermal Free Energies= -1238.074656

Standard orientation. Coordinates (Angstroms):

S	0.371069000	-0.480117000	1.245743000
C	1.676972000	-0.158666000	0.048941000
C	2.851678000	-1.168191000	0.004759000
C	2.390058000	1.223547000	0.133973000
C	-0.903883000	-0.153900000	-0.062703000
C	-1.826023000	1.157217000	0.023357000
C	-2.197324000	-1.076759000	-0.081684000
C	-3.025024000	0.217417000	-0.185154000
C	-1.827485000	1.834707000	1.401450000
H	-1.991551000	1.131641000	2.223542000
H	-0.868692000	2.334681000	1.568775000
H	-2.629652000	2.579964000	1.428925000
C	-1.635807000	2.206819000	-1.079417000
H	-1.667227000	1.767304000	-2.079378000
H	-2.436438000	2.950251000	-1.006060000
H	-0.674717000	2.716687000	-0.952710000
C	-2.463703000	-1.824496000	1.235348000
H	-3.483195000	-2.223742000	1.217795000
H	-1.763014000	-2.657857000	1.349178000
H	-2.373517000	-1.181296000	2.116689000
C	-2.343325000	-2.030550000	-1.273006000
H	-1.624702000	-2.854396000	-1.195075000
H	-3.353745000	-2.452844000	-1.271467000
H	-2.181815000	-1.524166000	-2.227233000
O	-4.193576000	0.420091000	-0.401126000
N	1.044921000	-0.172209000	-1.332058000
N	-0.186258000	-0.177656000	-1.332703000
O	1.834683000	2.265225000	0.382887000
O	2.793483000	-2.322510000	0.343535000
C	3.879801000	1.045394000	-0.155574000
C	4.079146000	-0.434596000	-0.529763000
H	4.081909000	-0.564245000	-1.620205000
H	4.992700000	-0.888147000	-0.138237000
H	4.428868000	1.317338000	0.755034000
H	4.189827000	1.749636000	-0.933669000

28. Transition states for decompositions of thiadiazolines 6'

Gas phase, 6-31G(d), PBE1PBE

TS$_{6'a\rightarrow N\equiv N+7'a}$

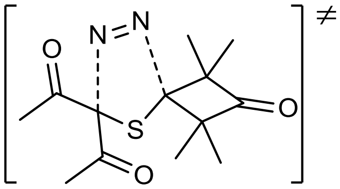

Imaginary Freq.: -341.28 cm^{-1}
Sum of electronic and zero-point Energies= -1239.184697
Sum of electronic and thermal Energies= -1239.163160
Sum of electronic and thermal Enthalpies= -1239.162216
Sum of electronic and thermal Free Energies= -1239.233369

Standard orientation. Coordinates (Angstroms):

S	-0.422519000	-0.885124000	-0.918437000
C	-1.859286000	-0.114704000	-0.265090000
C	-2.980932000	-1.105683000	-0.212020000
O	-2.945095000	-2.107444000	-0.914907000
C	-4.130367000	-0.917462000	0.767857000
H	-3.797848000	-0.510639000	1.726930000
H	-4.893579000	-0.246913000	0.358739000
H	-4.588827000	-1.897540000	0.917416000
C	-2.021285000	1.352293000	-0.338047000
O	-1.161739000	2.058359000	-0.861263000
C	-3.247912000	2.019100000	0.265556000
H	-4.150852000	1.773912000	-0.304656000
H	-3.413612000	1.715329000	1.303298000
H	-3.090165000	3.098319000	0.221584000
C	0.907030000	-0.213955000	0.018120000
C	1.763898000	1.103445000	-0.141743000
C	2.198347000	-1.099189000	0.119318000
C	2.987548000	0.222075000	0.172292000
C	1.819735000	1.599842000	-1.604497000
H	2.015203000	0.792287000	-2.315559000
H	0.869328000	2.067357000	-1.862351000
H	2.631168000	2.330248000	-1.690939000
C	1.556769000	2.281826000	0.813514000
H	1.561688000	1.970026000	1.861638000
H	2.380805000	2.989510000	0.675180000
H	0.615351000	2.789611000	0.589188000
C	2.518235000	-1.941728000	-1.133200000
H	3.547876000	-2.305897000	-1.056119000
H	1.849822000	-2.806951000	-1.193307000
H	2.428061000	-1.373818000	-2.062881000
C	2.331847000	-1.976499000	1.373710000
H	1.586913000	-2.779962000	1.357706000
H	3.328635000	-2.428842000	1.387198000
H	2.205540000	-1.403096000	2.294647000
O	4.138740000	0.476613000	0.419672000
N	-0.969498000	0.033854000	1.808646000
N	0.163469000	-0.074904000	1.666303000

$TS_{6'b \rightarrow N \equiv N+7'b}$

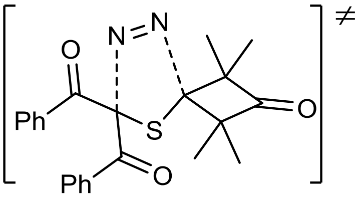

Imaginary Freq.: -346.99 cm^{-1}
Sum of electronic and zero-point Energies= -1622.550325
Sum of electronic and thermal Energies= -1622.522605
Sum of electronic and thermal Enthalpies= -1622.521660
Sum of electronic and thermal Free Energies= -1622.609449

Standard orientation. Coordinates (Angstroms):

S	1.181338000	-0.415227000	-1.252835000
C	-0.289554000	-0.407386000	-0.313846000
C	-1.339242000	-1.256548000	-0.946141000
O	-1.198752000	-1.693551000	-2.089699000
C	-0.660847000	0.816948000	0.491003000
O	-0.066672000	1.103519000	1.526116000
C	2.477132000	-0.341529000	-0.079748000
C	3.077968000	0.859215000	0.755386000
C	3.890988000	-0.753215000	-0.610534000
C	4.452639000	0.408679000	0.223858000
C	2.694030000	2.257917000	0.239509000
H	2.694924000	2.310736000	-0.853793000
H	1.702584000	2.536972000	0.603392000
H	3.425719000	2.981025000	0.615802000
C	3.007511000	0.806137000	2.290306000
H	3.344945000	-0.156826000	2.683841000
H	3.670006000	1.582128000	2.688315000
H	1.987619000	0.988305000	2.631495000
C	4.144498000	-0.576611000	-2.119296000
H	5.219232000	-0.669061000	-2.306823000
H	3.624456000	-1.348528000	-2.696071000
H	3.820487000	0.403371000	-2.483347000
C	4.372697000	-2.142544000	-0.153906000
H	3.798563000	-2.926446000	-0.659973000
H	5.429167000	-2.257277000	-0.417270000
H	4.267754000	-2.283562000	0.924141000
O	5.569206000	0.827661000	0.395930000
N	0.661563000	-1.607391000	1.315858000
N	1.799104000	-1.514987000	1.209011000
C	-1.745182000	1.722671000	-0.012447000
C	-2.202106000	1.707446000	-1.339882000
C	-2.275279000	2.666799000	0.883807000
C	-3.175713000	2.614701000	-1.757076000
H	-1.794098000	1.000361000	-2.054012000
C	-3.255343000	3.561450000	0.468149000
H	-1.903319000	2.677956000	1.902876000
C	-3.707372000	3.537499000	-0.855118000
H	-3.517785000	2.599758000	-2.788022000
H	-3.667317000	4.279706000	1.171715000
H	-4.470809000	4.238676000	-1.181636000
C	-2.559138000	-1.611535000	-0.149457000
C	-2.643454000	-1.477938000	1.245377000
C	-3.655118000	-2.136489000	-0.854394000
C	-3.808917000	-1.850117000	1.915970000
H	-1.796323000	-1.114014000	1.814908000
C	-4.817917000	-2.500159000	-0.183470000

H	-3.568197000	-2.250937000	-1.929722000
C	-4.898020000	-2.355804000	1.205108000
H	-3.861727000	-1.750522000	2.996627000
H	-5.662495000	-2.898076000	-0.739583000
H	-5.805293000	-2.642028000	1.730722000

<u>***$TS_{6'c \rightarrow N \equiv N+7'c}$***</u>

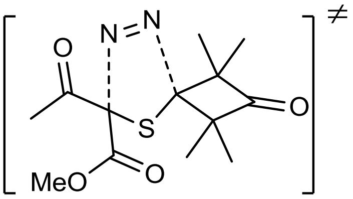

Imaginary Freq.: -344.70 cm^{-1}
Sum of electronic and zero-point Energies= -1314.411999
Sum of electronic and thermal Energies= -1314.389383
Sum of electronic and thermal Enthalpies= -1314.388439
Sum of electronic and thermal Free Energies= -1314.462503

Standard orientation. Coordinates (Angstroms):

S	-0.088306000	-1.087454000	-0.901144000
C	-1.618914000	-0.540204000	-0.258544000
C	-2.597850000	-1.673603000	-0.181410000
O	-2.361134000	-2.721132000	-0.769222000
C	-3.844773000	-1.543564000	0.674651000
H	-3.644403000	-1.025693000	1.615953000
H	-4.611190000	-0.968922000	0.145357000
H	-4.219198000	-2.552752000	0.860815000
C	-2.007900000	0.877370000	-0.362116000
O	-1.315145000	1.766385000	-0.833042000
C	-3.656411000	2.496404000	0.112362000
H	-3.673173000	2.862601000	-0.917169000
H	-4.659486000	2.502643000	0.539814000
H	-2.984846000	3.124760000	0.703341000
C	1.129370000	-0.219744000	0.023931000
C	1.764768000	1.217167000	-0.135029000
C	2.543645000	-0.890580000	0.106183000
C	3.116873000	0.537656000	0.154486000
C	1.719640000	1.726610000	-1.593499000
H	2.023617000	0.962882000	-2.314949000
H	0.705183000	2.046749000	-1.832788000
H	2.408883000	2.572690000	-1.686191000
C	1.389898000	2.340418000	0.835567000
H	1.464576000	2.025476000	1.879993000
H	2.086708000	3.172319000	0.689233000
H	0.374739000	2.690957000	0.633753000
C	2.979741000	-1.672066000	-1.150759000
H	4.053925000	-1.872683000	-1.082945000
H	2.452795000	-2.630231000	-1.208015000
H	2.794981000	-1.123035000	-2.077918000
C	2.823022000	-1.736192000	1.358770000
H	2.213274000	-2.646489000	1.346826000
H	3.878698000	-2.025865000	1.364632000
H	2.613686000	-1.189896000	2.281028000
O	4.217876000	0.969774000	0.382914000
N	-0.766942000	-0.254261000	1.814296000
N	0.370698000	-0.196560000	1.682823000
O	-3.233951000	1.123906000	0.155616000

TS$_{6'd \rightarrow N\equiv N+7'd}$

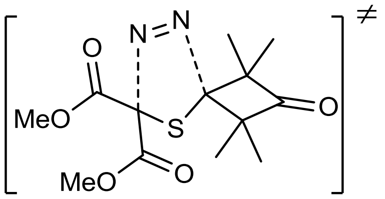

Imaginary Freq.: -344.01 cm^{-1}
Sum of electronic and zero-point Energies= -1389.632780
Sum of electronic and thermal Energies= -1389.609030
Sum of electronic and thermal Enthalpies= -1389.608086
Sum of electronic and thermal Free Energies= -1389.685345

Standard orientation. Coordinates (Angstroms):

S	0.024023000	-0.872384000	-0.983395000
C	-1.431991000	-0.190555000	-0.306459000
C	-2.532440000	-1.199620000	-0.328697000
O	-2.534345000	-2.183057000	-1.047564000
C	-4.572206000	-1.889355000	0.602104000
H	-5.071557000	-1.931169000	-0.369605000
H	-4.203145000	-2.887042000	0.854451000
H	-5.255344000	-1.526380000	1.370449000
C	-1.669921000	1.265300000	-0.334958000
O	-0.811734000	2.099027000	-0.586545000
C	-3.207693000	3.013973000	-0.015324000
H	-2.999438000	3.464634000	-0.989374000
H	-4.266365000	3.100806000	0.230454000
H	-2.596105000	3.509420000	0.743589000
C	1.337076000	-0.261518000	0.009339000
C	2.152839000	1.088207000	-0.006362000
C	2.648384000	-1.116341000	0.020939000
C	3.403836000	0.213245000	0.201987000
C	2.184313000	1.748118000	-1.403834000
H	2.376174000	1.027493000	-2.204178000
H	1.226743000	2.233527000	-1.594354000
H	2.988338000	2.491702000	-1.417890000
C	1.921764000	2.143382000	1.079240000
H	1.963620000	1.715596000	2.084588000
H	2.713384000	2.896351000	1.005137000
H	0.954528000	2.629381000	0.931948000
C	2.993306000	-1.827451000	-1.304220000
H	4.030526000	-2.174735000	-1.255766000
H	2.345125000	-2.697438000	-1.453269000
H	2.895004000	-1.172782000	-2.174465000
C	2.799379000	-2.103106000	1.189949000
H	2.078810000	-2.922605000	1.091049000
H	3.809561000	-2.524678000	1.171975000
H	2.646232000	-1.621815000	2.158248000
O	4.552362000	0.472737000	0.456452000
N	-0.594919000	-0.192654000	1.782651000
N	0.542252000	-0.287601000	1.682712000
O	-2.940007000	1.603901000	-0.040204000
O	-3.492736000	-0.942109000	0.576806000

$TS_{6'e \rightarrow N \equiv N + 7'e}$

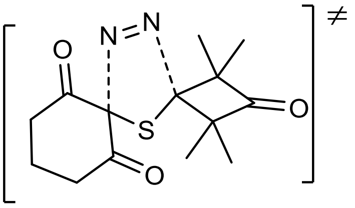

Imaginary Freq.: -332.74 cm^{-1}
Sum of electronic and zero-point Energies= -1277.295039
Sum of electronic and thermal Energies= -1277.274376
Sum of electronic and thermal Enthalpies= -1277.273432
Sum of electronic and thermal Free Energies= -1277.342720

Standard orientation. Coordinates (Angstroms):

S	-0.156757000	-0.987563000	-0.928708000
C	-1.654589000	-0.274949000	-0.389721000
C	-2.755596000	-1.265871000	-0.275456000
C	-1.931265000	1.164886000	-0.457391000
C	1.106336000	-0.211762000	0.035628000
C	1.883215000	1.155016000	-0.161592000
C	2.454566000	-1.008670000	0.166750000
C	3.159489000	0.360218000	0.172019000
C	1.895218000	1.618344000	-1.634625000
H	2.138720000	0.808794000	-2.328495000
H	0.912527000	2.015536000	-1.891014000
H	2.655264000	2.398944000	-1.745219000
C	1.601309000	2.336718000	0.770144000
H	1.643351000	2.050843000	1.825187000
H	2.369647000	3.098972000	0.604755000
H	0.622167000	2.767415000	0.546619000
C	2.828936000	-1.869075000	-1.057646000
H	3.880201000	-2.161228000	-0.968331000
H	2.219242000	-2.777940000	-1.089242000
H	2.704274000	-1.338552000	-2.005308000
C	2.639148000	-1.832748000	1.450017000
H	1.945740000	-2.681055000	1.461970000
H	3.662125000	-2.221089000	1.479180000
H	2.476161000	-1.236879000	2.351111000
O	4.294098000	0.692556000	0.401935000
N	-0.771864000	-0.004206000	1.783550000
N	0.357105000	-0.079692000	1.609700000
O	-1.118014000	1.988737000	-0.872647000
O	-2.604947000	-2.450643000	-0.544216000
C	-4.047660000	0.613780000	0.890933000
C	-3.303843000	1.632804000	0.024289000
C	-4.114878000	-0.739538000	0.180375000
H	-5.058615000	0.975299000	1.110582000
H	-3.534542000	0.501466000	1.853103000
H	-4.568655000	-1.517656000	0.802603000
H	-4.740767000	-0.661407000	-0.721815000
H	-3.889736000	1.857953000	-0.879989000
H	-3.157094000	2.588755000	0.537959000

$TS_{6'f \rightarrow N \equiv N + 7'f}$

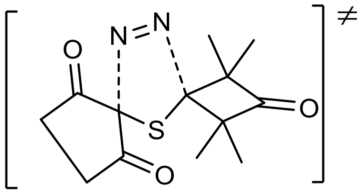

Imaginary Freq.: -337.02 cm^{-1}
Sum of electronic and zero-point Energies= -1238.011761
Sum of electronic and thermal Energies= -1237.992041
Sum of electronic and thermal Enthalpies= -1237.991097
Sum of electronic and thermal Free Energies= -1238.058775

Standard orientation. Coordinates (Angstroms):

S	-0.371192000	-0.965966000	-0.927852000
C	-1.811398000	-0.232494000	-0.324951000
C	-3.004549000	-1.082149000	-0.132940000
C	-2.185350000	1.181027000	-0.282566000
C	0.925591000	-0.244348000	0.038632000
C	1.653208000	1.154631000	-0.116631000
C	2.294607000	-1.009116000	0.056771000
C	2.968388000	0.376190000	0.077331000
C	1.538212000	1.724203000	-1.546189000
H	1.740744000	0.973703000	-2.315803000
H	0.529781000	2.116690000	-1.691371000
H	2.269592000	2.531474000	-1.657923000
C	1.405997000	2.256710000	0.918445000
H	1.536525000	1.899532000	1.944064000
H	2.134858000	3.056311000	0.751716000
H	0.399776000	2.666397000	0.798109000
C	2.616978000	-1.807192000	-1.222860000
H	3.676701000	-2.080936000	-1.204018000
H	2.023820000	-2.726755000	-1.263886000
H	2.431378000	-1.235947000	-2.136629000
C	2.571473000	-1.876589000	1.294378000
H	1.909341000	-2.749480000	1.305462000
H	3.607571000	-2.227488000	1.257076000
H	2.432382000	-1.323421000	2.226194000
O	4.110507000	0.725012000	0.230592000
N	-0.902893000	-0.115902000	1.836095000
N	0.220662000	-0.210137000	1.638038000
O	-1.502381000	2.155188000	-0.576387000
O	-3.083435000	-2.288273000	-0.287511000
C	-3.630565000	1.271201000	0.226460000
C	-4.155428000	-0.172976000	0.319956000
H	-4.443473000	-0.458639000	1.337723000
H	-5.024343000	-0.361500000	-0.319359000
H	-4.202250000	1.898689000	-0.465488000
H	-3.628229000	1.789089000	1.192270000

29. Thiodiazolines 6' decomposition products

Gas phase, 6-31G(d), PBE1PBE

Nitrogen (N≡N)

Sum of electronic and zero-point Energies= -109.518530
Sum of electronic and thermal Energies= -109.516170
Sum of electronic and thermal Enthalpies= -109.515225
Sum of electronic and thermal Free Energies= -109.536980

Standard orientation. Coordinates (Angstroms):

N	0.000000000	0.000000000	0.552751000
N	0.000000000	0.000000000	-0.552751000

C=S-ylide 7'a

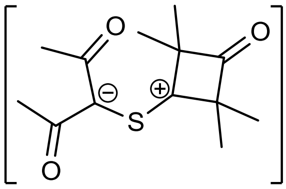

Sum of electronic and zero-point Energies= -1129.715756
Sum of electronic and thermal Energies= -1129.695322
Sum of electronic and thermal Enthalpies= -1129.694377
Sum of electronic and thermal Free Energies= -1129.765058

Standard orientation. Coordinates (Angstroms):

S	-0.416444000	-1.055080000	-0.563170000
C	-1.884903000	-0.169782000	-0.166029000
C	-2.807341000	-1.132359000	0.398657000
O	-2.388714000	-2.267265000	0.689384000
C	-4.276836000	-0.823962000	0.644330000
H	-4.405872000	0.014462000	1.336585000
H	-4.802482000	-0.571923000	-0.282384000
H	-4.728587000	-1.717354000	1.079474000
C	-2.028808000	1.177352000	-0.680377000
O	-1.098431000	1.772897000	-1.240115000
C	-3.367465000	1.902201000	-0.551672000
H	-4.156280000	1.406196000	-1.126915000
H	-3.704755000	1.963850000	0.487934000
H	-3.226915000	2.910856000	-0.944816000
C	0.944078000	-0.226349000	-0.193683000
C	1.399976000	0.901231000	0.739411000
C	2.363020000	-0.726863000	-0.503796000
C	2.812404000	0.282319000	0.581129000
C	1.386540000	2.347812000	0.197494000
H	1.745486000	2.406792000	-0.832963000
H	0.377877000	2.758786000	0.213576000
H	2.048641000	2.945320000	0.833435000
C	0.809278000	0.838277000	2.157234000
H	0.855913000	-0.171918000	2.575909000
H	1.376977000	1.508571000	2.810780000
H	-0.236636000	1.158464000	2.147066000
C	2.864426000	-0.363699000	-1.918209000
H	3.949270000	-0.505529000	-1.964789000
H	2.391621000	-1.016971000	-2.659369000
H	2.639315000	0.672419000	-2.185653000
C	2.664106000	-2.195913000	-0.170714000
H	2.196482000	-2.863663000	-0.902299000
H	3.746776000	-2.356880000	-0.196336000
H	2.300067000	-2.468770000	0.824547000
O	3.865315000	0.487742000	1.125017000

C=S-ylide 7'b

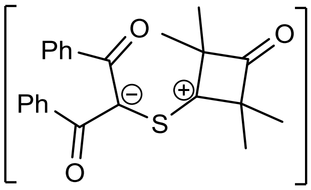

Sum of electronic and zero-point Energies= -1513.075188
Sum of electronic and thermal Energies= -1513.048732
Sum of electronic and thermal Enthalpies= -1513.047788
Sum of electronic and thermal Free Energies= -1513.133729

Standard orientation. Coordinates (Angstroms):

C	1.710549000	-1.075177000	-0.262141000
S	0.501441000	-0.603392000	-1.251499000
C	-0.826188000	0.010955000	-0.236913000
C	-1.953048000	-0.803039000	-0.609895000
C	-0.661084000	1.226343000	0.547741000
O	-1.316059000	1.467071000	1.570455000
O	-1.699265000	-1.746492000	-1.408342000
C	-3.384951000	-0.579014000	-0.233874000
C	-3.810802000	0.014275000	0.963635000
C	-4.348651000	-1.064490000	-1.136793000
C	-5.174842000	0.130970000	1.238367000
H	-3.076090000	0.381867000	1.668169000
C	-5.705935000	-0.930869000	-0.864330000
H	-4.008419000	-1.546200000	-2.046918000
C	-6.124167000	-0.332346000	0.328019000
H	-5.493098000	0.587132000	2.172200000
H	-6.439192000	-1.298813000	-1.577371000
H	-7.184690000	-0.233711000	0.546162000
C	0.335221000	2.275332000	0.103443000
C	0.550500000	2.594162000	-1.245403000
C	0.980958000	3.037075000	1.089278000
C	1.418454000	3.628469000	-1.601038000
H	0.009156000	2.059052000	-2.019847000
C	1.861918000	4.056166000	0.735608000
H	0.770482000	2.821294000	2.131980000
C	2.085830000	4.352570000	-0.612540000
H	1.563257000	3.874426000	-2.649640000
H	2.366121000	4.628952000	1.509520000
H	2.765565000	5.154282000	-0.888874000
C	1.856145000	-1.449348000	1.218972000
C	3.114365000	-1.544498000	-0.661961000
C	1.914023000	-0.313483000	2.255745000
H	2.527283000	0.526253000	1.916227000
H	0.909134000	0.057200000	2.474602000
H	2.353553000	-0.709442000	3.177569000
C	0.883009000	-2.557718000	1.670109000
H	-0.125659000	-2.148834000	1.775223000
H	0.845733000	-3.387903000	0.958258000
H	1.213768000	-2.947182000	2.638568000
C	4.077439000	-0.401208000	-1.042417000
H	5.104009000	-0.782183000	-1.049938000
H	3.835273000	-0.019829000	-2.040142000
H	4.025618000	0.434069000	-0.337309000
C	3.257420000	-1.979004000	0.819292000
C	3.182323000	-2.696575000	-1.677565000
H	2.937076000	-2.338415000	-2.683363000
H	4.197477000	-3.106604000	-1.692585000
H	2.489165000	-3.504450000	-1.423738000
O	4.135387000	-2.526077000	1.432038000

C=S-ylide 7'c

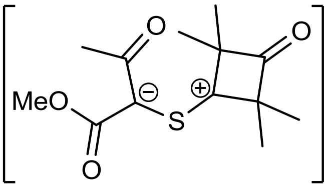

Sum of electronic and zero-point Energies= -1204.941451
Sum of electronic and thermal Energies= -1204.920234
Sum of electronic and thermal Enthalpies= -1204.919290
Sum of electronic and thermal Free Energies= -1204.992041

Standard orientation. Coordinates (Angstroms):

S	0.131397000	-1.206369000	0.099181000
C	1.733498000	-0.567917000	0.009517000
C	2.675274000	-1.699234000	-0.099477000
O	2.247888000	-2.852536000	-0.189253000
C	4.177575000	-1.469760000	-0.096657000
H	4.502909000	-0.937662000	0.801751000
H	4.652032000	-2.451982000	-0.145309000
H	4.487537000	-0.862762000	-0.952322000
C	2.014437000	0.848033000	0.093126000
O	1.171865000	1.730395000	0.223101000
O	3.338540000	1.147784000	0.014413000
C	3.654744000	2.543401000	0.104842000
H	4.741495000	2.598114000	0.028667000
H	3.185920000	3.101987000	-0.709917000
H	3.316684000	2.958674000	1.058139000
C	-1.188591000	-0.232294000	0.057445000
C	-1.749301000	1.188660000	-0.127533000
C	-2.584210000	-0.905582000	0.115876000
C	-3.136046000	0.506666000	-0.168100000
C	-1.655805000	2.124964000	1.098521000
H	-1.915954000	1.604956000	2.026720000
H	-0.644545000	2.519042000	1.188034000
H	-2.369216000	2.944733000	0.959467000
C	-1.390725000	1.917239000	-1.433168000
H	-1.441246000	1.245586000	-2.297233000
H	-2.111718000	2.726829000	-1.591657000
H	-0.383588000	2.330307000	-1.363760000
C	-2.989738000	-1.459841000	1.494657000
H	-4.065403000	-1.665528000	1.504217000
H	-2.453951000	-2.393152000	1.700169000
H	-2.768706000	-0.754358000	2.301575000
C	-2.893016000	-1.917929000	-1.000268000
H	-2.358198000	-2.858077000	-0.827483000
H	-3.968131000	-2.125343000	-1.015654000
H	-2.604498000	-1.535667000	-1.984584000
O	-4.247337000	0.924077000	-0.368311000

C=S-ylide 7'd

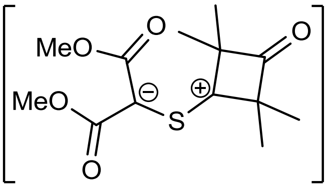

Sum of electronic and zero-point Energies= -1280.161208
Sum of electronic and thermal Energies= -1280.138814
Sum of electronic and thermal Enthalpies= -1280.137869
Sum of electronic and thermal Free Energies= -1280.213917

Standard orientation. Coordinates (Angstroms):

S	0.025170000	-1.068444000	0.082769000
C	1.527008000	-0.232800000	0.021534000
C	2.598707000	-1.241217000	-0.040773000
O	2.386673000	-2.446500000	-0.104529000
C	4.895984000	-1.706125000	-0.072218000
H	5.822690000	-1.131278000	-0.050060000
H	4.844801000	-2.385756000	0.782884000
H	4.829476000	-2.292949000	-0.992457000
O	3.846536000	-0.730192000	-0.011968000
C	1.632085000	1.212151000	0.080309000
O	0.669447000	1.963299000	0.212959000
O	2.891614000	1.688238000	-0.028639000
C	3.008645000	3.114536000	0.040741000
H	4.075998000	3.318839000	-0.052138000
H	2.455912000	3.592347000	-0.773306000
H	2.628314000	3.492526000	0.993956000
C	-1.411999000	-0.279708000	0.045788000
C	-2.159491000	1.058797000	-0.084355000
C	-2.702712000	-1.138996000	0.069333000
C	-3.442761000	0.198667000	-0.138178000
C	-2.166940000	1.964229000	1.166631000
H	-2.336260000	1.387782000	2.082622000
H	-1.215097000	2.487049000	1.251291000
H	-2.985017000	2.686144000	1.065858000
C	-1.925442000	1.864657000	-1.373761000
H	-1.900501000	1.215673000	-2.256146000
H	-2.751480000	2.573642000	-1.498857000
H	-0.981461000	2.406812000	-1.306233000
C	-3.021362000	-1.813029000	1.417118000
H	-4.059601000	-2.161710000	1.416328000
H	-2.363693000	-2.675014000	1.574707000
H	-2.891456000	-1.125640000	2.258843000
C	-2.881428000	-2.123954000	-1.098510000
H	-2.230025000	-2.995424000	-0.971060000
H	-3.920935000	-2.467124000	-1.129754000
H	-2.645981000	-1.658306000	-2.060817000
O	-4.604293000	0.470685000	-0.300563000

C=S-ylide 7'e

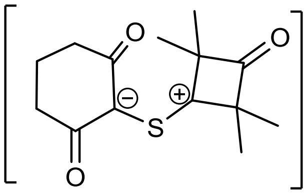

Sum of electronic and zero-point Energies= -1167.827619
Sum of electronic and thermal Energies= -1167.808277
Sum of electronic and thermal Enthalpies= -1167.807333
Sum of electronic and thermal Free Energies= -1167.875673

Standard orientation. Coordinates (Angstroms):

S	0.198042000	-1.081731000	0.080448000
C	1.731147000	-0.281710000	0.073720000
C	2.804771000	-1.278974000	0.004077000
O	2.589199000	-2.488172000	-0.074879000
C	4.236188000	-0.750493000	0.056926000
C	1.949173000	1.140243000	0.180385000
O	1.038715000	1.970829000	0.268066000
C	3.402939000	1.614338000	0.213100000
C	-1.201955000	-0.225195000	0.042376000
C	-1.863944000	1.157546000	-0.071714000
C	-2.540882000	-1.003112000	0.035870000
C	-3.197873000	0.380450000	-0.154656000
C	-1.823008000	2.036116000	1.198318000
H	-2.046405000	1.456202000	2.100499000
H	-0.835380000	2.484563000	1.300304000
H	-2.585153000	2.817082000	1.101419000
C	-1.557550000	1.971041000	-1.341361000
H	-1.563876000	1.339983000	-2.237027000
H	-2.333172000	2.735576000	-1.460834000
H	-0.580619000	2.446753000	-1.247485000
C	-2.910331000	-1.684305000	1.367115000
H	-3.965783000	-1.975650000	1.347191000
H	-2.301851000	-2.583303000	1.514474000
H	-2.754427000	-1.021197000	2.223728000
C	-2.759269000	-1.954378000	-1.153324000
H	-2.156920000	-2.861792000	-1.036391000
H	-3.815476000	-2.239519000	-1.200258000
H	-2.489828000	-1.483907000	-2.104295000
O	-4.338533000	0.725430000	-0.323660000
C	4.373557000	0.668900000	-0.498142000
H	3.417723000	2.622842000	-0.211909000
H	3.688275000	1.715786000	1.271390000
H	4.160957000	0.664915000	-1.575424000
H	5.403517000	1.025070000	-0.381431000
H	4.864153000	-1.472046000	-0.474511000
H	4.552759000	-0.771172000	1.111037000

C=S-ylide 7'f

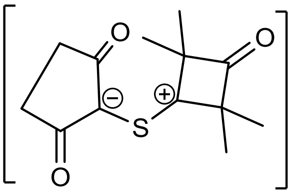

Sum of electronic and zero-point Energies= -1128.544649
Sum of electronic and thermal Energies= -1128.526262
Sum of electronic and thermal Enthalpies= -1128.525317
Sum of electronic and thermal Free Energies= -1128.591274

Standard orientation. Coordinates (Angstroms):

S	-0.418883000	-1.104659000	-0.000122000
C	-1.892376000	-0.243964000	-0.000065000
C	-3.080176000	-1.096322000	0.000408000
O	-3.119019000	-2.319754000	0.001024000
C	-4.309357000	-0.176431000	0.000129000
C	-2.231136000	1.158074000	-0.000749000
O	-1.492417000	2.141929000	-0.001403000
C	-3.766133000	1.262111000	-0.000435000
C	0.993900000	-0.271757000	0.000069000
C	1.602208000	1.135070000	0.000289000
C	2.350505000	-1.006493000	-0.000066000
C	2.969659000	0.411856000	0.000532000
C	1.372072000	1.968666000	-1.276389000
H	1.524527000	1.372955000	-2.183131000
H	0.355093000	2.364263000	-1.271202000
H	2.091216000	2.794884000	-1.288450000
C	1.370976000	1.969419000	1.276151000
H	1.522479000	1.374228000	2.183394000
H	2.090214000	2.795553000	1.288428000
H	0.354086000	2.365231000	1.269802000
C	2.666777000	-1.816229000	-1.270479000
H	3.731295000	-2.072655000	-1.282726000
H	2.084249000	-2.743968000	-1.288310000
H	2.440624000	-1.251644000	-2.180502000
C	2.666362000	-1.816126000	1.270627000
H	2.083553000	-2.743687000	1.288483000
H	3.730814000	-2.072809000	1.283101000
H	2.440189000	-1.251328000	2.180513000
O	4.106711000	0.804314000	0.000757000
H	-4.072931000	1.841025000	0.877774000
H	-4.073361000	1.840548000	-0.878802000
H	-4.920900000	-0.408281000	0.879023000
H	-4.920905000	-0.408915000	-0.878597000
